# Critical analysis of withdrawn drug applications in marketing authorization procedures of the European Medicines Agency (EMA) 2006–2024

**DOI:** 10.1007/s00210-026-05443-1

**Published:** 2026-05-22

**Authors:** Florian Hitsch, Roland Seifert

**Affiliations:** Institute of Pharmacology, Hannover Medical School, Carl-Neuberg-Str. 1, D-30625 Hannover, Germany

**Keywords:** EMA, Withdrawn drug applications, Marketing authorization procedures

## Abstract

Withdrawn drug applications (WDAs) are drug approval applications withdrawn by the applicant before the European Medicines Agency (EMA) has made a final decision. To better understand the main reasons for the emergence of WDAs, this study critically analyzes all WDAs in the period from 2006 to 2024. The data analysis is based on the 330 WDAs published by the EMA during the period mentioned above. Various parameters were analyzed, such as active substances, therapeutic subgroups and objections raised by the EMA. The most common therapeutic subgroups are antineoplastic agents (*N* = 80), followed by immunosuppressants (*N* = 23). Most drug approval applications are for initial approval (*N* = 244; 73.9%), supported by a single key clinical trial. In 77.3% (*N* = 255) of cases, the key clinical trials included a control arm (reference drug and/or placebo). In 69.7% (*N* = 230) of cases, no initial marketing authorization recommendation was made by the EMA’s Committee for Medicinal Products for Human Use (CHMP). The most common reason for this was concerns about clinical efficacy (33.46%; *N* = 348), partly due to inadequate clinical trial design. A major reason for the accumulation of WDAs in the antineoplastic agents group is presumably the high demand for supply and the resulting economic potential of this drug group. The present analysis may contribute to the identification of obstacles and the resulting potential for optimization in the EMA’s drug approval procedures.

**Supplementary Information:**

The online version contains supplementary material available at 10.1007/s00210-026-05443-1.

## Introduction

The European Medicines Agency (EMA) is an institution of the European Union (EU), responsible for the authorization and supervision of medicinal products within the EU. The EMA was founded in 1993 as the European Agency for the Evaluation of Medicinal Products (EMEA) before being renamed the European Medicines Agency (EMA) in 2009. EMA began its work as central authority for the regulation of drug approval processes in the EU back in 1995. The EMA’s core tasks include conducting centralized drug approval procedures, pharmacovigilance, public communication, and cooperation with other authorities at the national and international level (European Agency for the Evaluation of Medicinal Products, Strachan Heppell 1996; European Medicines Agency 2015; European Medicines Agency 2025a). During the EMA centralized drug approval procedures, various other scenarios may arise in addition to the primary goal of drug approval. For example, at the end of the drug approval process, a negative opinion (no recommendation for approval) from the EMA’s Committee for Medicinal Products for Human Use (CHMP) may result in a refusal of drug approval application by the EMA. These refusals, usually due to insufficient evidence of a positive benefit-risk ratio, become legally binding after the decision of the European Commission. However, if the negative CHMP opinion is already foreseeable before the EMA’s final statement, the applicant may withdraw the drug approval application on its own before the end of the drug approval process (withdrawal of drug application (WDA)). This process is shown in Table 1. In addition, even after a positive recommendation of the CHMP and subsequent legal marketing authorization by the European Commission, the marketing authorization holder may withdraw the existing marketing authorization for various reasons. Typical reasons in these cases are adverse drug reactions and economic interests. Drug approvals, refusals and the withdrawal of existing approvals (usually by the manufacturer) are the most prominent scenarios of drug approval procedures within scientific literature. In contrast, the withdrawal of drug approval applications in uncompleted drug approval procedures (WDAs) is discussed less frequently in the scientific literature. Studies on WDAs are largely confined to short time periods (e.g., 1995–1999, 2003–2010) (Tafuri et al. 2012; The European Agency for the Evaluation of Medicinal Products Human Medicines Evaluation Unit 1999; The European Agency for the Evaluation of Medicinal Products 2000) or specific therapeutic areas (e.g., oncology) (Cramer et al. 2025) (Table S1), although these cases can provide important information and insights for process optimization with regard to the further development of drug approval.

**Table 1:** EMA’s central drug approval procedure and the withdrawal of application procedure (European Medicines Agency 2020)

Research and development	• At the beginning research is carried out by pharmaceutical and biotechnology companies in the laboratory and after a national application for clinical trial, in compliance with good clinical practice (GCP), clinical
	• Applicants can voluntarily request scientific advice from the EMA (Scientific Advice Working Party (SAWP)) a few months before submission of marketing authorization application (no authorization guarantee)
Assessment	• EMA marketing authorization applications must comply with EU legislation, including data on manufacture, mechanism of action, efficacy, benefits, side effects and risk management
	• The central principle of the EMA’s CHMP assessment is the benefit-risk evaluation in peer reviews
	• The evaluation up to the first clock stop (time to answer outstanding questions from the CHMP) takes up to 120 days, followed by a second clock stop after 180 days at the latest
	• By day 210 at latest, after clarifying open questions, CHMP issues updated assessment/recommendation before final opinion (within 60 days)
Withdrawal of application procedure	Withdrawal of marketing authorization application:
	• Possible anytime before European Commission final decision and only by the applicant
	• The applicant must justify the withdrawal of marketing authorization in a withdrawal letter. In the course of the process, the following documents will be published with information on the application for admission and the reasons for the withdrawal (procedure is laid down in article 11 of regulation (EC) No 726/2004)
	• Q&A document (day after next CHMP plenary)
	• Withdrawal assessment report (≤ 3 months after withdrawal letter)
	• Procedural steps document for extension of indications (≤ 3 months after withdrawal letter)
	• Post-opinion withdrawals (reported separately in the aforementioned documents)

The aim of this study is to critically analyze the reasons and characteristics of WDAs, analyzing active substances, active substance groups, indication areas, clinical trials, time factors, and EMA assessment. The analysis covers all 330 WDAs published by the EMA between 2006 and 2024. The results of the analysis of the WDAs are intended to shed light on the topic of WDAs in EMA centralized procedures. In particular, the analysis aims to identify areas and topics with the greatest hurdles and thus the greatest potential for optimization during both application and drug approval process.

## Materials and methods

The critical analysis of WDAs in EMA centralized procedures between 2006 and 2024 was performed as a retrospective data analysis based on the data published and regularly updated by the EMA on drug approval procedures. The data analysis covers all 330 WDAs in the period from January 19, 2006, when the EMA began publishing data, until December 31, 2024 (Table S2). The dataset published by the EMA contains various information relevant to the drug approval process as well as some information on the specific drugs themselves (European Medicines Agency 2025b).

**Fig. 1:**
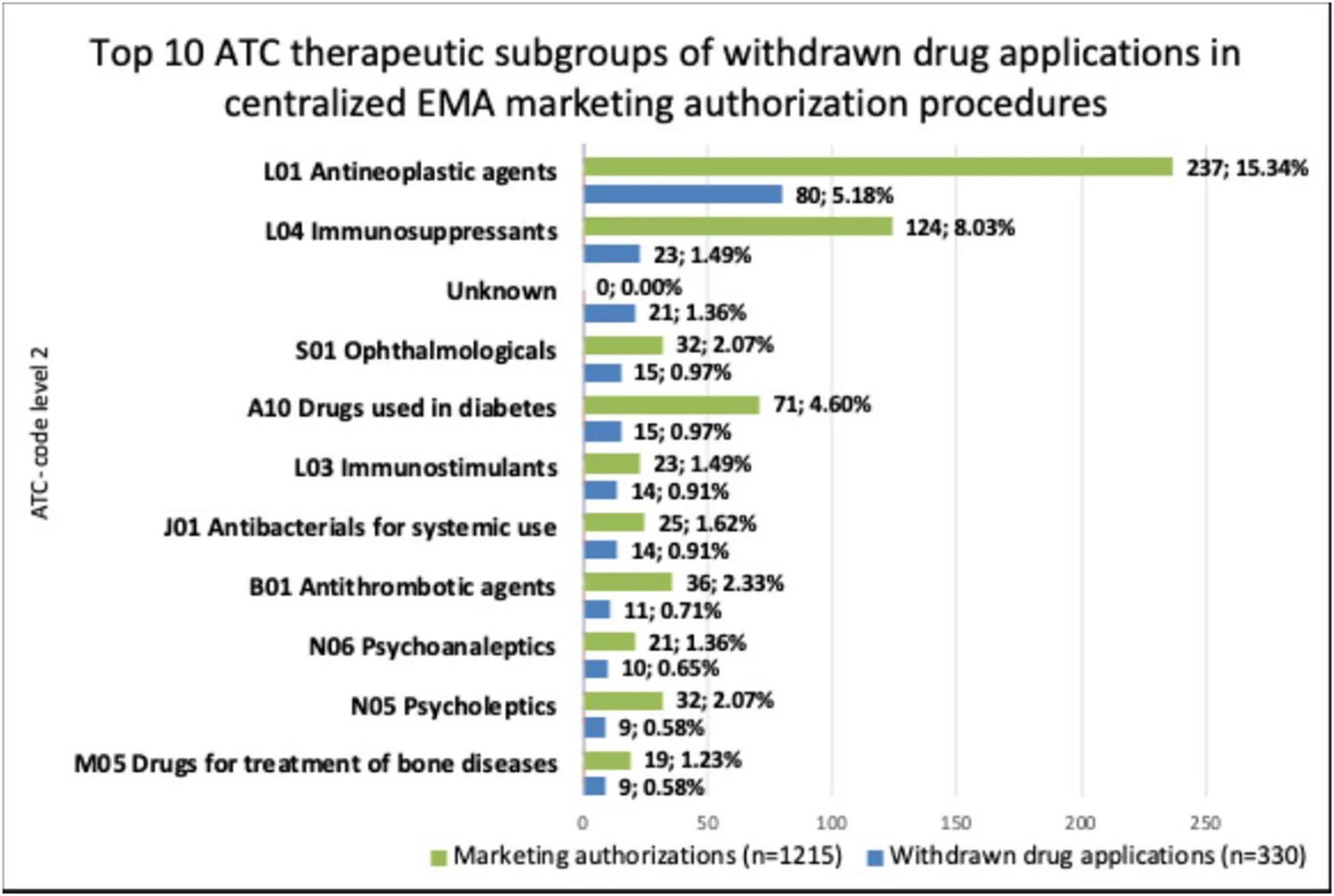
Top 10 ATC therapeutic subgroups of withdrawn drug applications in centralized EMA marketing authorization procedures. Percentages are based on total sample size of *N* = 1545 (WDAs and authorizations altogether)

**Fig. 2:**
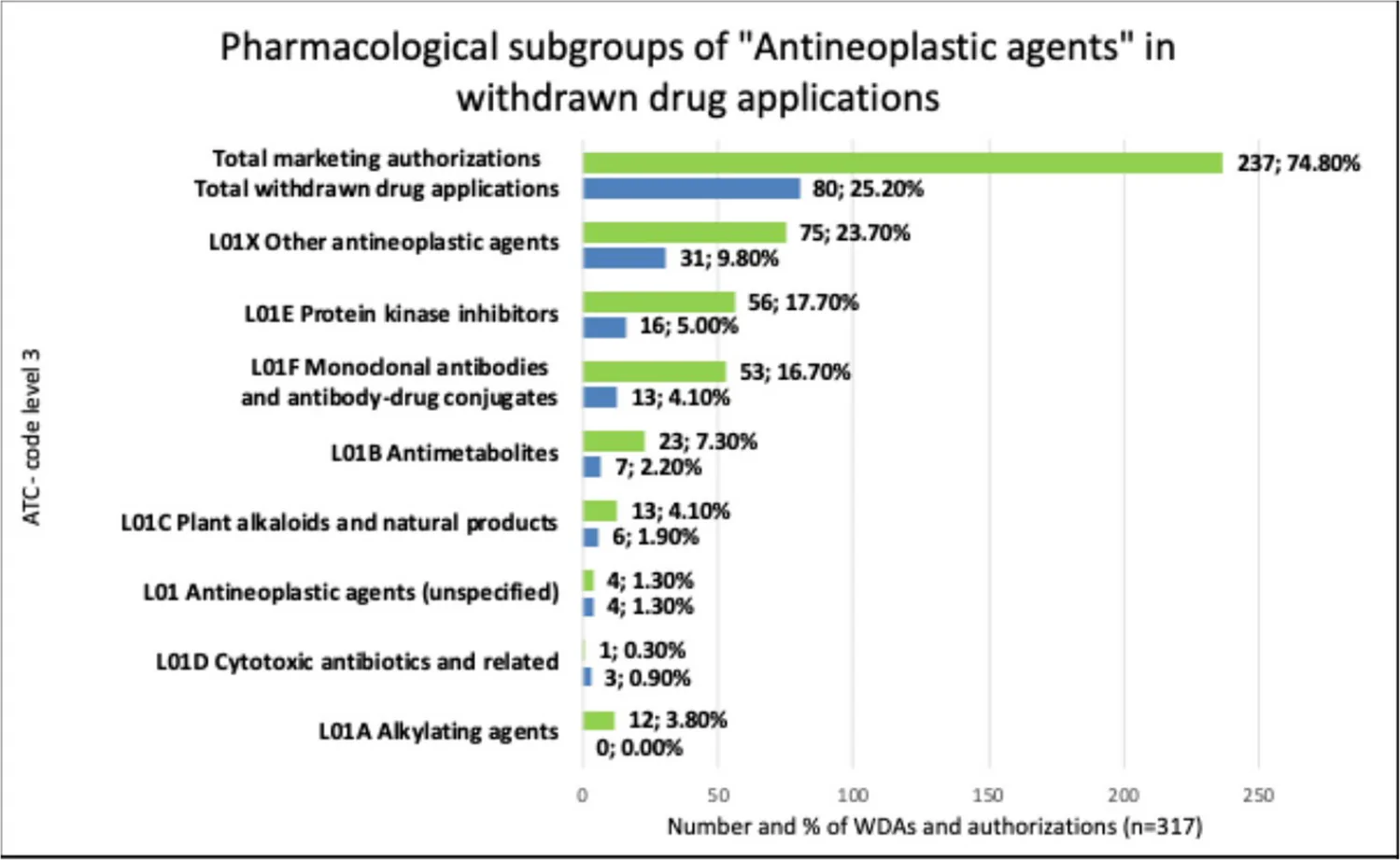
Pharmacological subgroups of “Antineoplastic agents” (ATC) in withdrawn drug applications. Percentages are based on total sample size of *N* = 317 (WDAs and authorizations in subgroup “Antineoplastic agents” altogether)

Based on these data published by EMA, the available information was supplemented with additional information on manufacturers, active substances, indications and the timing of the WDAs. Additional analysis criteria were also added, such as missing assignment of WDAs to the classification of active substances using the Anatomical Therapeutic Chemical Classification System (ATC codes, WHO Collaborating Centre for Drug Statistics 2024). Each WDA was categorized and evaluated based on its respective ATC level 2 (therapeutic subgroup) or level 3 (therapeutic/pharmacological subgroup) classification. A comparison of therapeutic subgroups (ATC level 2) or therapeutic/pharmacological subgroups (ATC level 3) for WDAs (2006–2024) versus completed marketing authorizations within the same period was conducted using published data (European Medicines Agency 2025b). Marketing authorizations comprised all 1215 EMA-approved medicinal products (2006–2024) with active status as of April 2026. The reported percentages comparing WDAs and marketing authorizations (Fig. 1) are based on a total sample size of *N* = 1545 (100%), comprising all withdrawn drug applications (WDAs; *N* = 330; 21.4%) and all marketing authorizations (*N* = 1215; 78.6%) (Fig. 1). The reported percentages comparing WDAs and markerting authorizations in the ATC subgroup “antineoplastic agents” (Fig. 2) are based on a total sample size of *N* = 317 (100%) in this ATC subgroup, comprising all withdrawn drug applications (WDAs; *N* = 80; 25.2%) and all marketing authorizations (*N* = 237; 74.8%) in EMA marketing authorization procedures within the specified period.

In addition, other important analysis criteria, such as concerns and recommendations of the EMA, date of withdrawal (initial authorization or extensions), reasons for withdrawal given by manufacturers, types and scope of documentation in marketing authorization applications and types of marketing authorization procedures (such as generics and biosimilars), were added in the current analysis. Information on the respective WDAs, such as indications, clinical studies, EMA statements and statements of the manufacturer was taken from the summary EMA information (questions and answers on the withdrawal of application) or the respective “withdrawal letter” published in the context of the drug approval procedures. Key clinical trials were defined as the clinical studies designated in the published EMA documents during the marketing authorization process as the pivotal trials guiding the regulatory decision. Specific analysis criteria were coded to enable data collection and evaluation of the WDAs using Microsoft Excel. The descriptive statistical data analysis was followed by a literature review and a comparison with other studies and findings from the scientific literature. The methodological approach is depicted in Table 2.

**Table 2:** Methodological approach

Data basis	• Data on withdrawn drug applications in the EMA’s drug approval procedures are publicly available and regularly updated by the EMA
	• The data set contains all 330 withdrawn drug applications between 2006 and 2024 (initial marketing authorizations or marketing authorization extensions) published by the EMA
Determination of the analysis criteria	• Defining the focus of data analysis
	• Analysis criteria (selection): Active substances, active substance groups, target structures, types of approval procedures, clinical studies in marketing authorization applications, pharmaceutical companies, EMA opinion and recommendations
Revision and supplementation of existing data	• Addition and revision of the active substance groups, ICD-11 indication areas, approval procedure types and drug target structures
	• Supplementation of clinical data in marketing authorization applications, opinions and recommendations of the EMA as well as in statements for reasons of revocation by pharmaceutical companies
Evaluation and analysis of the dataset	• Evaluation and analysis of the data with Microsoft Excel
	• Graphical representation of the results
Interpretation of the data	• Interpretation of the results and addition of further analysis criteria such as: – Orphan drugs – ATC codes
Literature research and writing	• Comparison of the results with data from the literature
	• Research, especially on PubMed and in drug prescription reports (2010–2024), EMA reports and guidelines

## Results

### Withdrawn drug applications: therapeutic subgroups, pharmacological subgroups and active substances

The WDAs in EMA centralized procedures were classified according to the Anatomical Therapeutic Chemical Classification System (ATC codes) and assigned to their respective therapeutic subgroups (WHO Collaborating Centre for Drug Statistics 2024) (Fig. 1). This analysis shows that antineoplastic agents (*N* = 80; 5.2%), immunosuppressants (*N* = 23; 1.5%) and ophthalmologicals (*N* = 15; 1.0%) are the most common active substance groups in ATC level 2 (therapeutic subgroups). However, some active substances could not be assigned to a clear ATC code (unknown, *N* = 21; 1.4%). In accordance, antineoplastic agents (*N* = 237; 15.3%) and immunosuppressants (*N* = 124; 8.0%) were also the most frequent therapeutic subgroups amongst EMA marketing authorizations within the same period (2006–2024) (Fig. 1). In contrast, differences between the frequency of a particular therapeutic subgroup within WDAs and total marketing authorizations were observed for drugs used in diabetes (*N* = 71; 4.6%), antivirals for systemic use (*N* = 61; 3.9%), and vaccines (*N* = 47; 3.0%). In WDAs, these therapeutic subgroups were either absent or ranked differently with regard to frequency when compared to total marketing authorizations.

Analysis of WDAs within the therapeutic subgroup “Antineoplastic agents” (ATC level 2) reveals that “Other antineoplastic agents” (not elsewhere classified) (*N* = 31; 9.8%), protein kinase inhibitors (*N* = 16; 5.0%), and monoclonal antibodies and antibody–drug conjugates (*N* = 13; 4.1%) are the most frequent pharmacological subgroups (ATC level 3; Fig. 2). These subgroups also represent the most frequent categories among EMA marketing authorizations during the same period (Fig. 2).

**Fig. 3:**
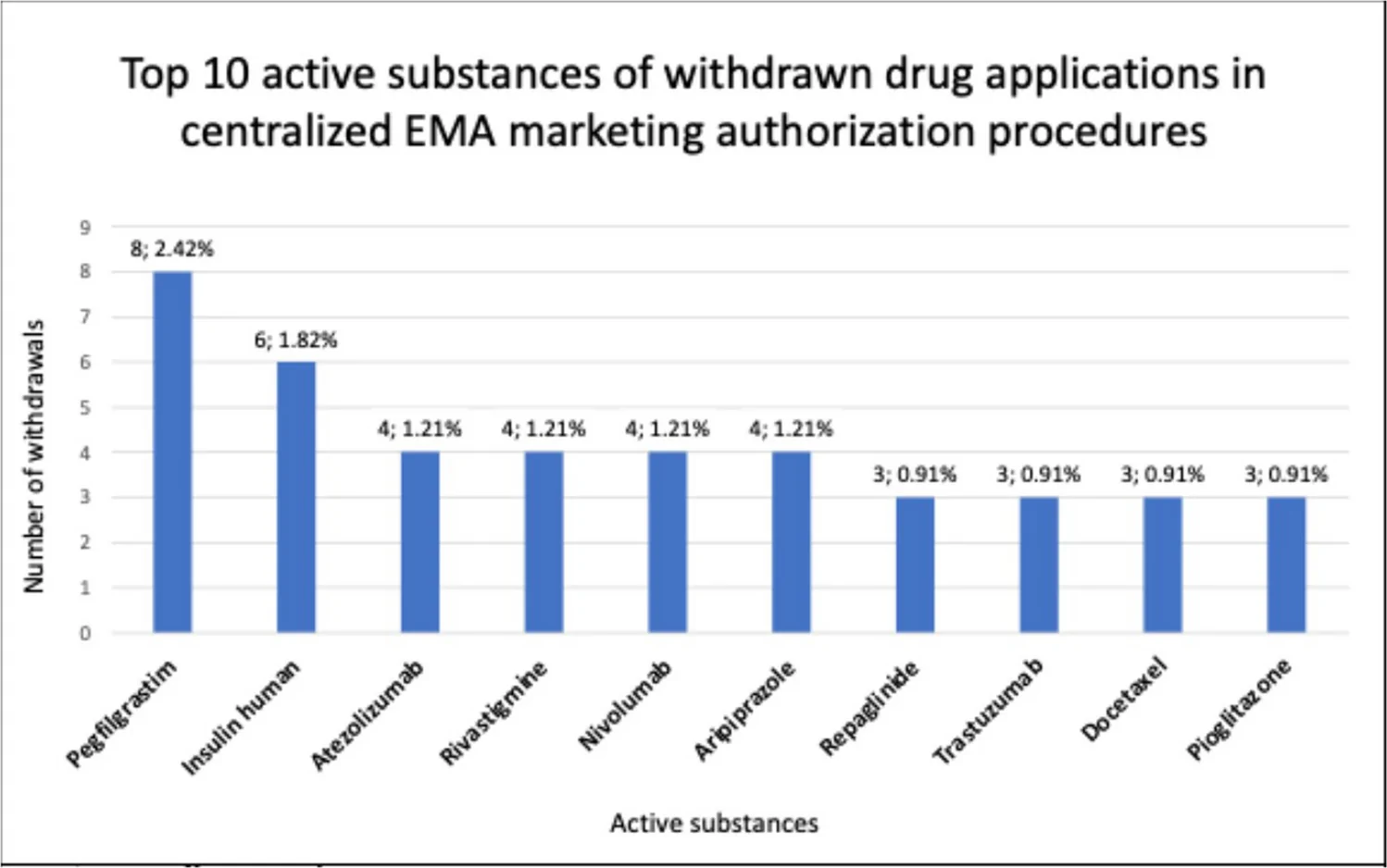
Top 10 active substances of withdrawn drug applications in centralized EMA marketing authorization procedures (% of total WDAs, *N* = 330)

Regarding the 330 WDAs in central drug approval procedures by the EMA between 2006 and 2024 (Fig. 3), the most common active substances are pegfilgrastim (*N* = 8; 2.4%) and human insulin (*N* = 6; 1.8%), followed by atezolizumab (*N* = 4; 1.2%), rivastigmine (*N* = 4; 1.2%), nivolumab (*N* = 4; 1.2%), and aripiprazole (*N* = 4; 1.2%). Both WDAs in initial marketing authorization applications and applications for extension of authorization (for already approved indications) were considered.

### Types of marketing authorizations and clinical studies of withdrawn drug applications

**Fig. 4:**
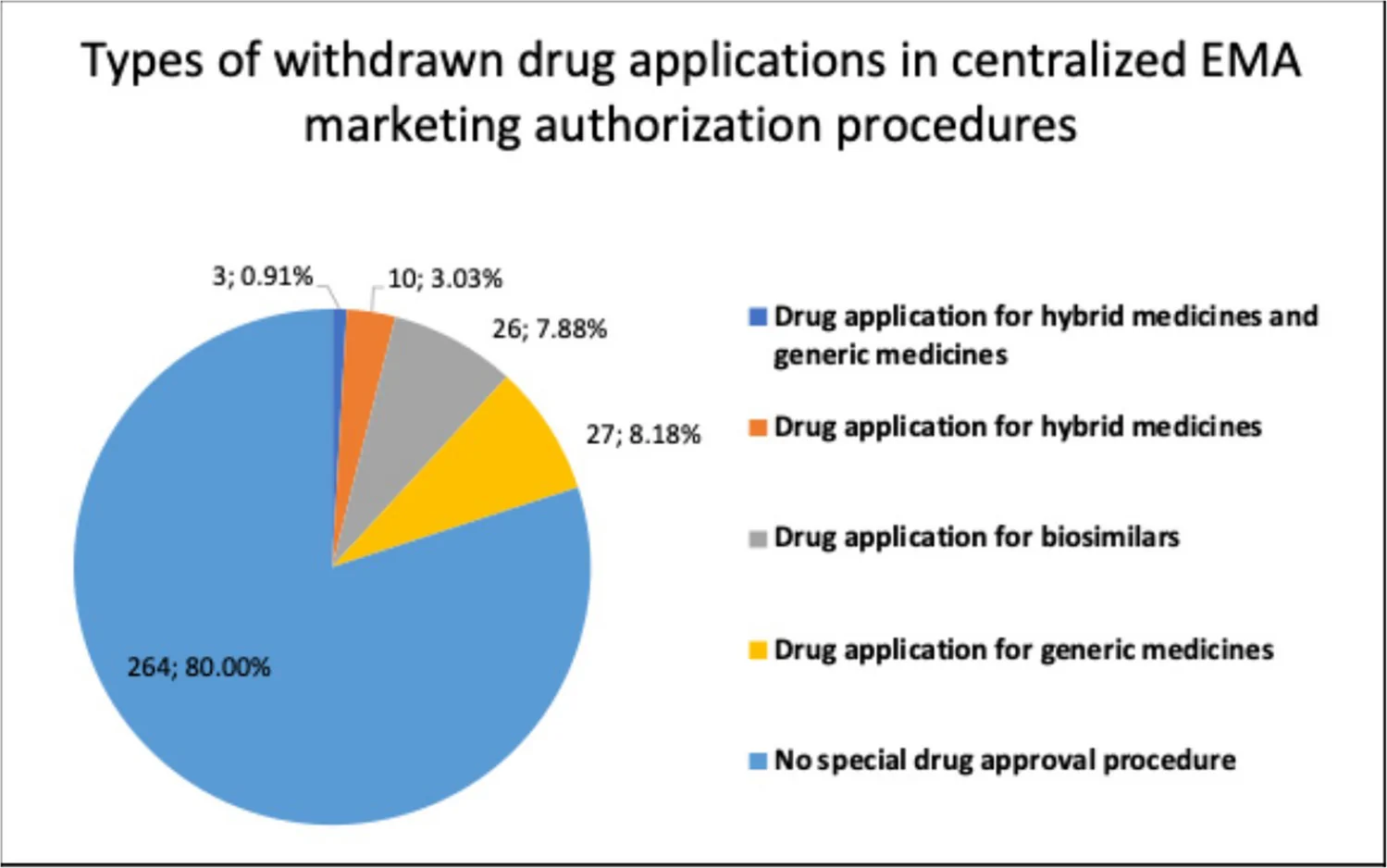
Types of withdrawn drug applications in centralized EMA marketing authorization procedures

**Fig. 5:**
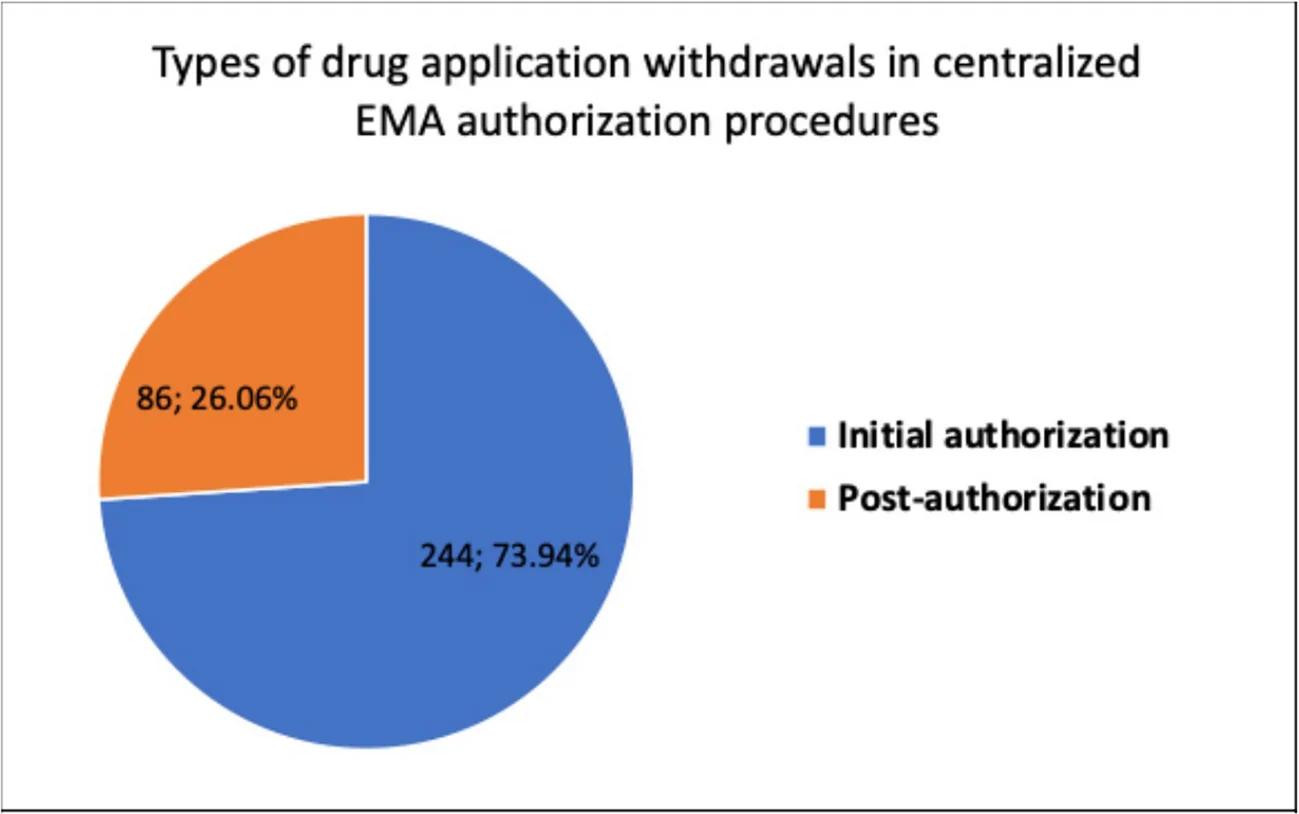
Types of drug application withdrawals in centralized EMA authorization procedures

There are various types of drug approval applications in the EMA centralized procedures. Drug approval applications are divided into applications for new active substances, generics, biosimilars, and hybrid drugs. Generic drugs are defined as drugs that are approved as imitation products after the patent protection for the original preparations has expired (Federal Ministry of Health 2025). On the other hand, so-called biosimilars (e.g., generic antibodies) are imitation products of complex biological drugs (e.g., hormones or monoclonal antibodies) that are not identical to the original preparation in terms of purity, structure etc., but are merely similar and thus equivalent (Federal Agency for Safety in Health Care (BASG) 2025). Another type of drug approval is the application for approval of hybrid drugs. Hybrid drugs are similar to an already approved drug in terms of the active substance, but differ from the original preparation in terms of indication, drug strength, or dosage form (European Medicines Agency 2025c). Due to the differences mentioned above, the drug approval procedures for new active substances, generics, biosimilars, and hybrid drugs differ in terms of their approval requirements and the approval process. An analysis of the WDA drug approval procedures shows that special approval procedures (for biosimilars, generics, and hybrid drugs) account for a total of 20.0% (*N* = 66) of the WDA approval procedures (Fig. 4). Among these special drug approval procedures, generics (*N* = 27; 8.2%) are the most common ones, followed by biosimilars (*N* = 26; 7.9%). In contrast, 80.0% (*N* = 264) of the 330 WDAs did not involve any of the special drug approval procedures mentioned above. The 330 WDAs between 2006 and 2024 can also be analyzed in more detail regarding the timing of the drug approval applications. In 73.9% (*N* = 244) of the WDAs, the applications were submitted for initial marketing authorization, while in 26.1% (*N* = 86) of the WDAs, the applications were made for an extension or change to an existing marketing authorization (post-authorization) (Fig. 5).

**Fig. 6:**
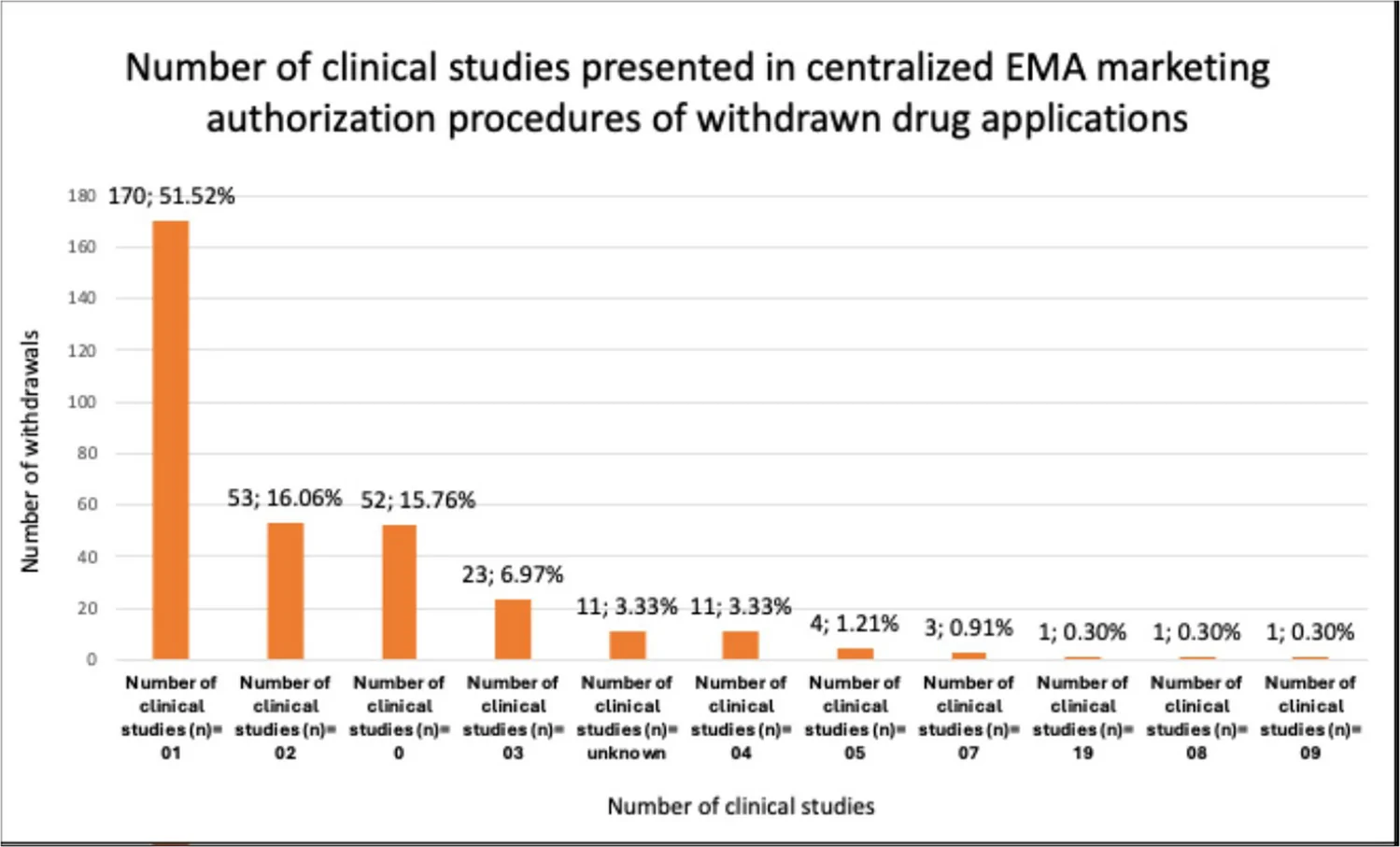
Number of clinical studies presented in centralized EMA marketing authorization procedures of withdrawn drug applications

**Fig. 7:**
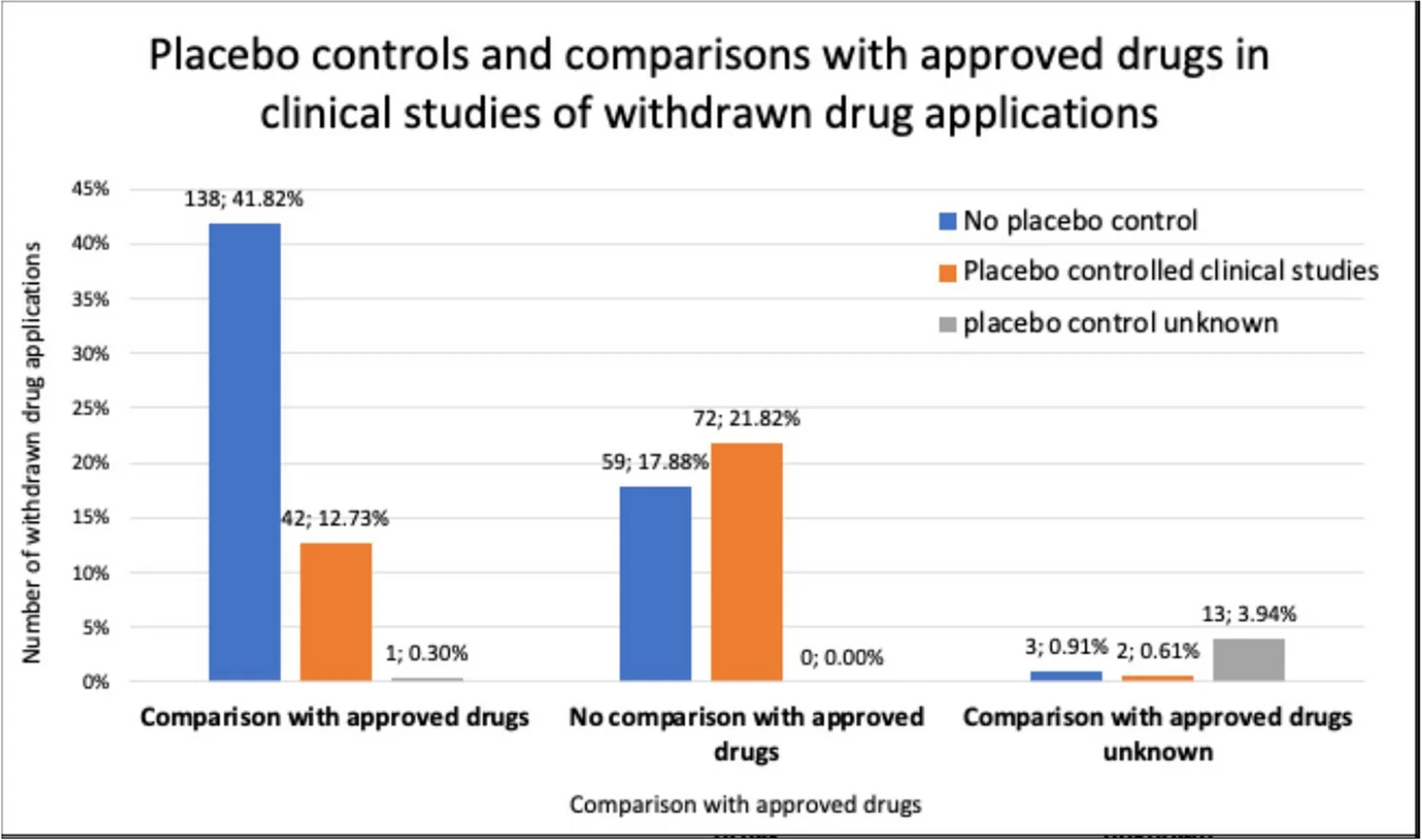
Placebo controls and comparisons with approved drugs in clinical studies of withdrawn drug applications

One key clinical trial was submitted in 51.5% (*N* = 170) of the drug approval applications (Fig. 6). Two key clinical trials were submitted in 16.1% (*N* = 53) of the 330 drug approval applications. No clinical trials were submitted for 15.8% (*N* = 52) of the drug approval applications, while more than two clinical trials were submitted for the remaining 13.3% (*N* = 44) of the drug approval applications. The number of marketing authorization applications without clinical trials also includes marketing authorization procedures for generics and biosimilars, which typically require only equivalence or similarity testing with the reference medicinal product rather than additional clinical trials. The evaluation of clinical trials includes all key clinical trials mentioned in the summary information published by the EMA. Supportive clinical studies and data, quality studies, and bioequivalence studies were not considered; 54.8% (*N* = 181) of the 330 drug approval applications described a comparison with already approved drugs (Fig. 7). In 42 (12.7% of all 330 WDAs) of these 181 drug approval procedures, a comparison with placebo treatment was also described in the application documents. In contrast, no comparison with reference drugs was described in 39.7% (*N* = 131) of the drug approval procedures, although a comparison with placebo treatment was made in 72 (21.8% of all 330 WDAs) of these 131 approval procedures. In 17.9% (*N* = 59) of the drug approval procedures, there was neither a comparison with reference drugs nor placebo treatment in the respective EMA information (Fig. 7). The complete absence of clinical comparisons in some applications reflects, among other issues, the inclusion of generics and biosimilars, which typically require only equivalence/similarity testing rather than key clinical trials.

### Analysis of EMA recommendations and manufacturer statements in withdrawn drug applications

**Fig. 8:**
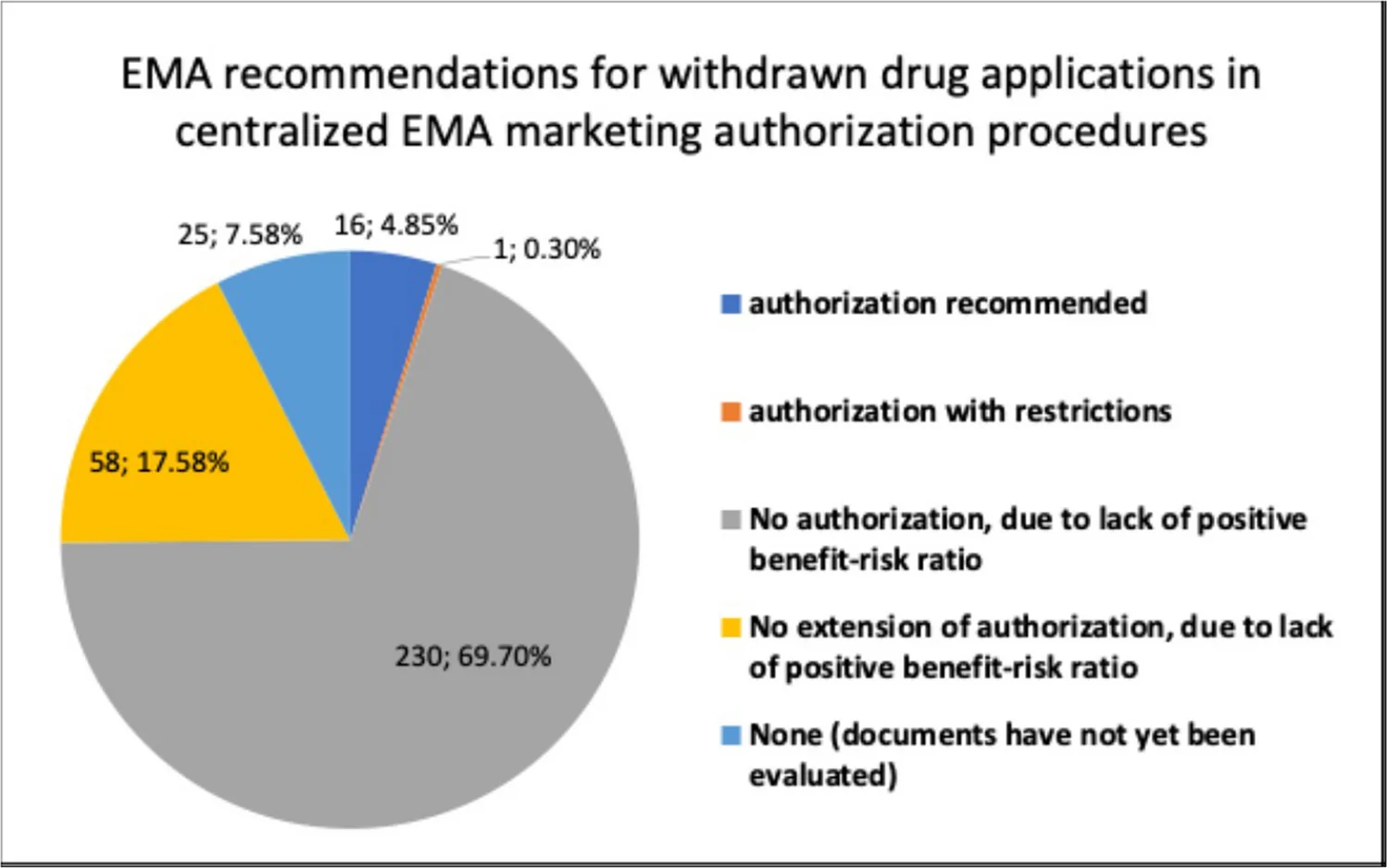
EMA recommendations for withdrawn drug applications in centralized EMA marketing authorization procedures

**Fig. 9:**
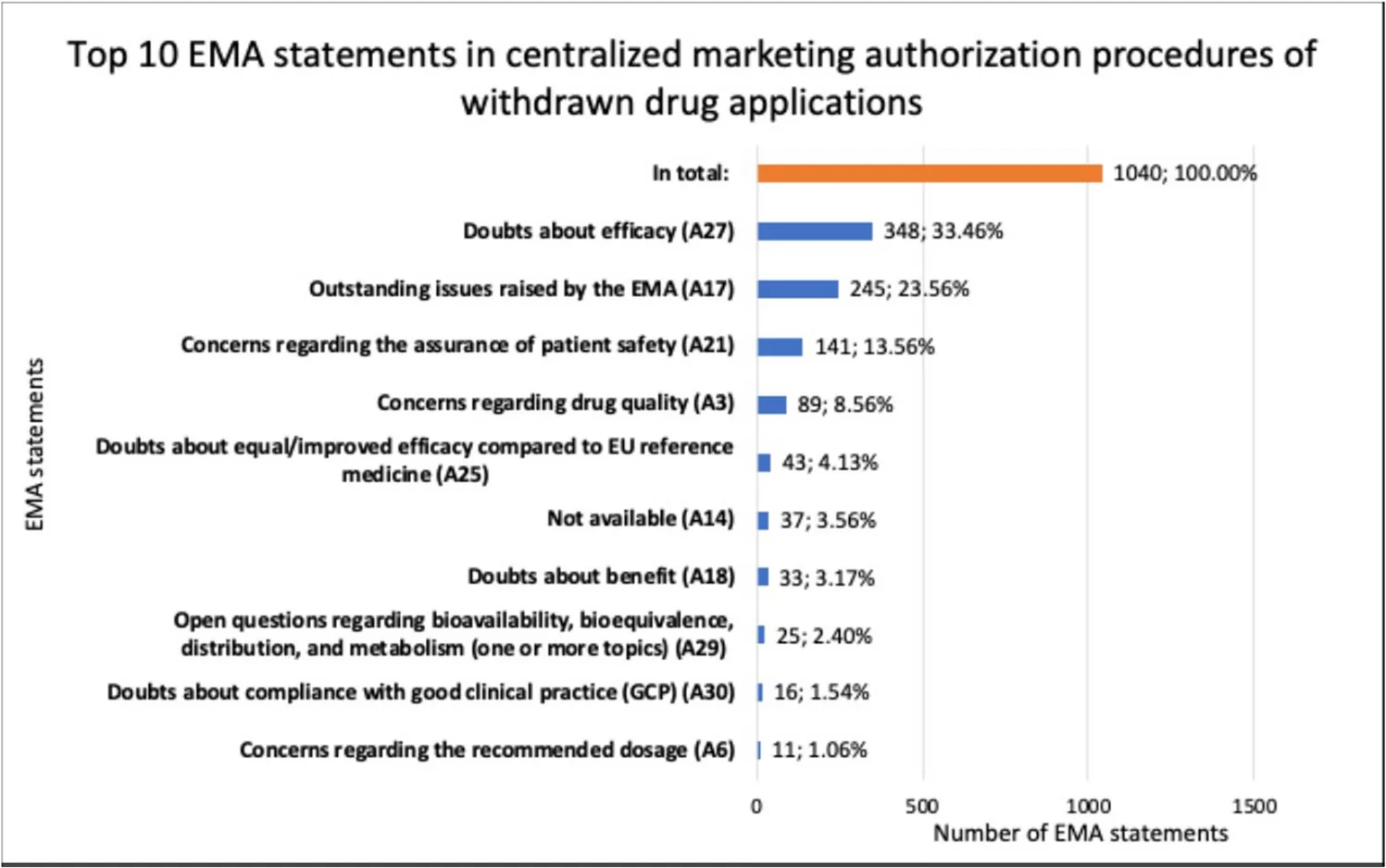
Top 10 EMA statements in centralized marketing authorization procedures of withdrawn drug applications

In 87.3% (*N* = 288) of the 330 WDA procedures, the EMA CHMP did not recommend marketing authorisation or extension of authorisation since a positive benefit-risk ratio had not been convincingly demonstrated (Fig. 8). In 17.6% (*N* = 58) of the WDAs with a negative opinion, this recommendation concerned applications for marketing authorization extensions or changes to existing drug approvals. However, in 4.9% (*N* = 16) of the drug approval procedures the applicants withdrew their applications (mostly for strategic or economic reasons) although the CHMP had previously issued a positive opinion for approval. In addition, the EMA’s CHMP was unable to make a recommendation in 7.6% (*N* = 25) of the WDA drug approval applications because the EMA had not yet completed its review of the application documents at the time the approval application was withdrawn. The EMA’s recommendations regarding the WDAs described above are based on various objections or feedback to the applicants resulting from the assessment of the application documents. Analysis of EMA statements on the respective marketing authorization applications with WDAs yielded a total of 1040 coded objections or feedback items for 330 WDAs in total (Fig. 9). The most frequent objections made were “doubts regarding efficacy (A27)” (*N* = 348; 33.5%), “Outstanding issues raised by the EMA (A17)” (*N* = 245; 23.6%) and “Concerns regarding the assurance of patient safety (A21)” (*N* = 141; 13.6%) (Fig. 9).

**Fig. 10:**
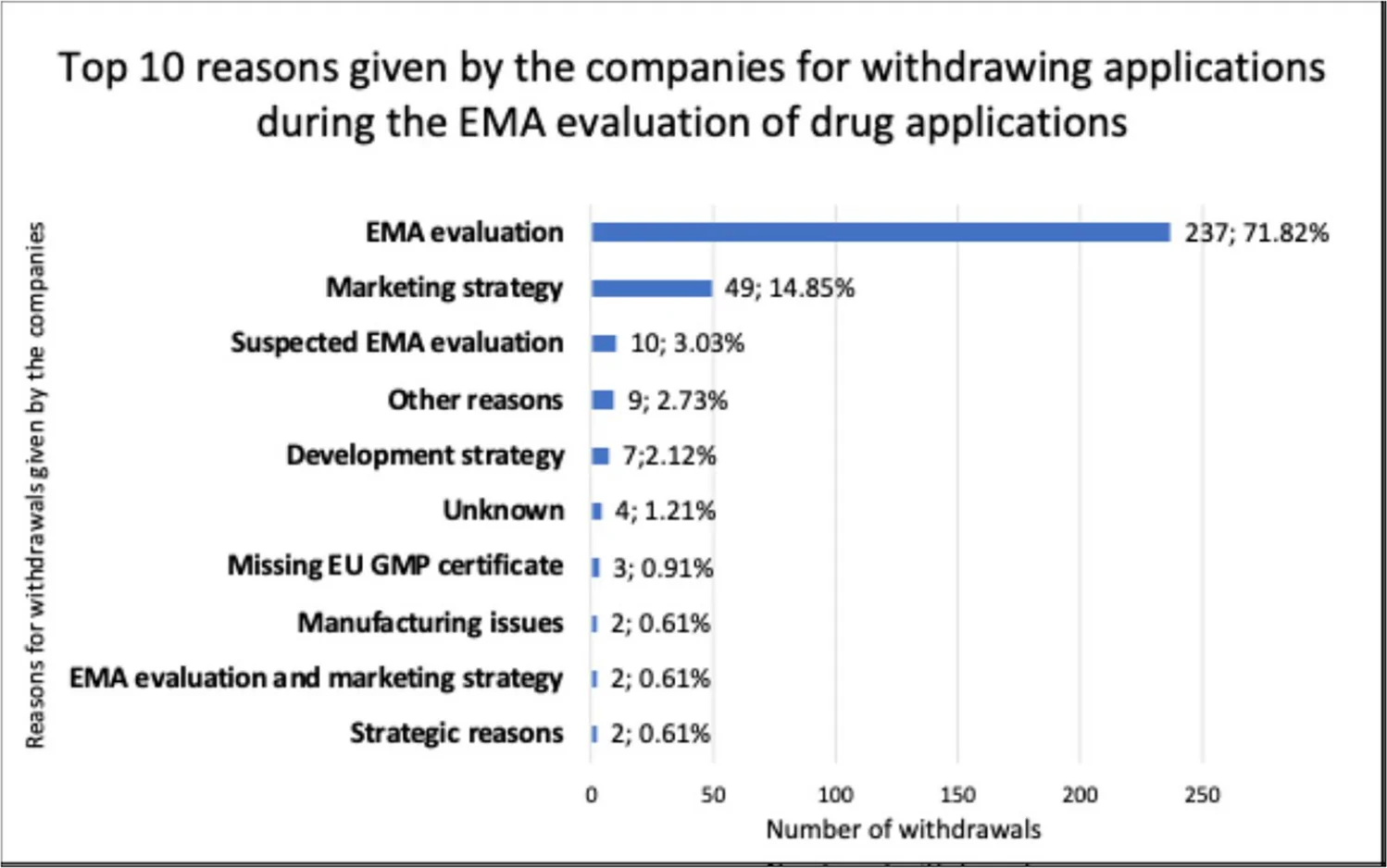
Top 10 reasons given by the companies for withdrawing applications during the EMA evaluation of drug applications

In the drug approval procedures for the 330 WDAs, the applicant’s reasons for withdrawing their drug approval applications were also published in the respective “withdrawal letter”. The most common reasons within 330 withdrawn drug approval applications were the “EMA evaluation” (*N* = 237; 71.8%), “marketing strategies” (*N* = 49; 14.9%) and the “suspected EMA evaluation” (not yet published at that time) (*N* = 10; 3.0%) (Fig. 10). However, in 1.2% (*N* = 4) of the WDAs, no reason could be determined for the applicant’s withdrawal of the marketing authorization application.

## Discussion

The aim of this study was to critically analyze the reasons and characteristics of withdrawn drug applications. Our results show clear differences in the frequency of WDAs between the therapeutic subgroups as well as different types and reasons for withdrawal.

### Therapeutic subgroups and active substances

Considering the most common therapeutic subgroups among WDAs (Fig. 1, ATC classification), multiple factors may account for the observed differences in the frequency amongst WDAs. One important factor leading to a high percentage of antineoplastic agents, immunosuppressants, and antidiabetic drugs among all WDAs may be the high market potential of these drugs. This view is supported by the fact that these drugs caused the highest net costs on the German statutory health insurance (SHI) drug market in 2023 (Ludwig et al. 2025). The aspect of medical need within the aforementioned groups of therapeutic subgroups (especially antineoplastic agents) is also of interest in this context, as malignant neoplasms (27.10%) ranked second behind cardiovascular diseases (27.80%) as causes of death in Western Europe in 2021 (according to the Global Burden of Disease Study 2021, see Mubarik et al. 2024). A look at the development of the number of newly introduced oncological drugs worldwide (11 new drugs in 2012, 30 new drugs in 2021) (Pharma Fakten e.V 2022) also shows the expanding research and development potential in the field of these therapeutic subgroups and indications. This is also reflected by the high number of clinical trials in oncological and immunological indications in Germany in 2023 (Verband der forschenden Pharma-Unternehmen in Deutschland 2024), since these clinical trials are performed with drugs also belonging to the most common therapeutic subgroups in WDAs (Fig. 1). In line with this observation, a closer look at the most common pharmacological subgroups (ATC level 3) listed in “Antineoplastic agents”; Fig. 2) shows that protein kinase inhibitors, monoclonal antibodies, and antimetabolites are (besides “Other antineoplastic agents”, not elsewhere classified), the most frequent pharmacological subgroups among WDAs in the therapeutic subgroup of “Antineoplastic agents”.

Interestingly, despite high treatment costs, kinase inhibitors for oncologic indications are frequently assessed by the Federal Joint Committee as having no proven additional therapeutic benefit (Obst and Seifert 2023, 2025). Furthermore, an analysis of monoclonal antibodies approved by the Food and Drug Administration (FDA), particularly for the treatment of malignant diseases (solid tumors), describes issues with a limited and highly variable efficacy accompanied by high costs. For example, two-arm clinical trials of monoclonal antibodies have demonstrated a 10.3% efficacy improvement and an increase in progression-free survival of 3 months at median annual therapy costs of $142,833 (Bordeau and Balthasar 2021). The structural and physiological characteristics of monoclonal antibodies are pointed out as reasons for the partially limited efficacy, as these factors significantly influence the penetration and distribution of the active substance, especially in solid tumors (Cruz and Kayser 2019). Thus, in the case of antineoplastic agents, there is presumably a correlation between the high number of drug approval applications resulting from increased research and high market potential due to high medical care needs. However, limited efficacy in some indications may contribute to the high number of WDAs amongst these drugs.

There are several reasons for WDAs of the most common active substances in EMA centralized procedures (Fig. 3). It is striking that WDAs contain many biologicals such as pegfilgrastim, human insulin, atezolizumab, and nivolumab. Possible reasons could include the substantially lower development costs of biosimilars compared with the original biological products, estimated at approximately 10%–20% of the development costs of the original reference biologicals. In addition, biosimilars generally have shorter development timelines, with an average of about 8 years until phase 3 compared with approximately 12 years for original reference biologicals (Agbogbo et al. 2019). However, despite the generally shorter development time, the scientific literature reports on the increased complexity of the drug approval process for biosimilars, mainly resulting from intermolecular variations between the original preparations (biologicals) and the biosimilars. Pharmacodynamic and pharmacokinetic studies, as well as studies comparing efficacy and immunogenicity, sometimes describe ambiguous or insufficient study results for biosimilars (Wolff-Holz et al. 2019). In particular, pharmacokinetic studies have proven to be extremely important in the approval process for biosimilars (Wolff-Holz et al. 2019). In addition to regulatory and pharmacological explanations, economic reasons (amongst many other influencing factors) could also have an important impact on WDAs of the most common active substances.

### Drug approval procedures and clinical data

Most WDAs (approximately 75%) occurred after application for initial approval (Fig. 5). Possible reasons for the high number of applications for initial marketing authorization among WDAs include the regulatory framework for drug approval procedures by the EMA. In this context, it is important to distinguish between decentralized, country-specific drug approval procedures and centralized drug approval procedures by the EMA. While decentralized drug approval only leads to approval in the country where the application is submitted, approval in the centralized EMA drug approval procedure ensures EU-wide drug approval through a single application (European Medicines Agency 2023). For certain medicinal products, such as gene therapy medicinal products, orphan drugs, medicinal products for the treatment of viral diseases, neurodegenerative diseases, diabetes mellitus and malignant diseases, a centralized EMA marketing authorization procedure is mandatory (European Medicines Agency 2024). However, other medicinal products, as well as generic medicinal products, can also be approved on a decentralized basis by the respective country-specific medicinal product approval authority. Consequently, failed applications from these decentralized procedures do not appear in EMA-published WDAs. It can therefore be assumed that the EMA-centralized drug approval procedures involve a higher number of applications for initial approval for complex active substances (e.g., antineoplastic agents) or complex diseases (e.g., malignant diseases). In addition, insufficient evidence of clinical efficacy and relevance is one of the most common reasons for withdrawals and refusals of drug approval applications at the EMA (Tafuri et al. 2012). Therefore, the high proportion of applications for initial approval among WDAs (Fig. 5) can mainly be explained by the regulatory framework of the EMA and the complexity of particular drugs or indications, possibly resulting in concerns regarding efficacy and clinical relevance. Concerns such as inadequate efficacy or clinical relevance play a minor role in the case of generic drugs and biosimilars, since the original product is already licensed. In line with this, none of the top 10 most frequently prescribed active substances in Germany in 2022 (all available as generics) are found among the most common active substances in WDAs (Schröder and Seifert 2025, Fig. 3).

Most of the WDAs had either one or two key clinical trials or none (Fig. 6). The relatively high number of marketing authorization applications without clinical trials can be explained by the fact that most of them are marketing authorization applications for generic drugs and biosimilars, for which clinical trials on efficacy are not mandatory due to the already approved original product. An analysis of drug marketing authorization applications submitted to the EMA between 2016 and 2018 shows similar results (Byrne et al. 2023). In this analysis, 142 drug approval applications were examined regardless of their approval status (131 (92.2%) drugs were approved, 11 (7.8%) drugs were withdrawn). Among the 119 finally included applications, 46.2% (*N* = 55) relied on one pivotal trial and 26.9% (*N* = 32) on two pivotal trials (Byrne et al. 2023). Analysis of the study design of the key clinical trials based on the number of key clinical trials in the drug approval applications of WDAs reveals that among 330 WDAs, 181 (54.8%) described comparisons with authorized drugs, while 131 (39.7%) did not. Of these 131 (39.7%) WDAs, 72 (21.8% of all 330 WDAs) used placebo controls (Fig. 7). In this context, the EMA’s regulatory recommendations state that randomized controlled trials (RCTs) are the standard for determining the efficacy of new drugs (Committee for Medicinal Products for Human Use (CHMP) 2023, European Medicines Agency 2001). The recommendation to use a reference drug or placebo as control was mostly followed in WDA approval procedures, as only 17.9% (*N* = 59) of WDAs lacked clinical control arms (neither placebo nor reference drug). In the literature there are varying results concerning the use of clinical control arms, depending on the indication area or the group of active substances. For example, an analysis of FDA drug approvals for the treatment of nine life-threatening diseases between 2006 and 2011 shows that 201/508 trials (39.6%) included a control arm with placebo, while 307 (60.4%) included an active control arm (reference drug). In addition, the proportion of placebo-only controlled trials depended on the clinical indication (75.5% for chronic obstructive pulmonary disease (COPD) and 0.0% for osteoporosis) (Sorscher et al. 2018). Other evaluations of non-oncological drugs approved by the EMA and/or the FDA (2011–2016) based on a single key clinical trial showed that of 27 key clinical trials for 27 indications, 13 (48%) key clinical trials included a placebo control. Ten (37%) of the key clinical trials also included an active control arm (reference drug), while 4 (15%) were conducted without a control arm (Morant et al. 2019). The number of clinical trials with active controls (reference drug), placebo controls and no control arm thus varies depending on the indication, type of marketing authorization application and drug class. This seems reasonable since the most appropriate control arm within clinical trials is determined by standard therapeutic algorithms which may differ substantially between different indications.

“Doubts about efficacy”, “Outstanding issues raised by the EMA,”, and “Concerns regarding the assurance of patient safety” are the most common concerns leading to negative EMA recommendations (Fig. 9). A study evaluating the “major objections” in 64 EMA drug approval applications (42 received a recommendation for approval) from small and medium-sized pharmaceutical companies between 2011 and 2015 comes to similar conclusions. It reports that “major objections” regarding clinical efficacy are a leading cause with 80% (*N* = 51), followed by “major objections” regarding quality (73%; *N* = 47) and clinical safety (48%; *N* = 31). In addition, in 20/22 (91%) of marketing authorization applications, the negative EMA opinion was based upon unresolved concerns regarding clinical efficacy (Amaouche et al. 2018). Another study including 86 marketing authorization applications with negative EMA opinions (withdrawal or refusal) between 2003 and 2010 shows similar results. A total of 156 “major objections” were identified, of which 106 (67.9%) were related to clinical efficacy, 27 (17.3%) to drug safety and 23 (21.6%, per source) to drug quality (Tafuri et al. 2012). This again shows that, in addition to clinical safety and drug quality, clinical efficacy is the most important factor in EMA centralized drug approval procedures. In this context, the importance of a safe and effective drug approval procedure is highlighted by an analysis of medicines with innovative principles newly approved by the EMA between 2004 and 2011, excluding kinase inhibitors and monoclonal antibodies. In this analysis, “Rote-Hand-Briefe” (Direct Healthcare Professional Communications) were issued for 30% of these 190 new drugs within the first 10 years after marketing authorization (Manfouo and Seifert 2025).

## Limitations

Limiting factors in the analysis of publicly available data and EMA reports on WDAs were partially incomplete data on the respective drug approval procedures. If possible, these data were added by further research. In addition, there were some differences in the structure and content of the EMA reports (questions and answers on the withdrawal of the marketing authorization application) on the respective drug approval procedures. Therefore, in some cases, understanding of individual recommendations and comparing WDAs was difficult. In particular, there were some differences in the information provided in the EMA reports regarding clinical trials (number of key clinical trials, participants, study design, control groups). Wherever possible, these inaccuracies or missing data were overcome by detailed analysis of withdrawal assessment reports. Another limiting factor in the data analysis was an occasionally inconsistent assignment of active substances to a particular active substance group.

## Future studies

Analysis of EMA centralized drug approval procedures will continue to be an important tool for classifying and interpreting developments and changes in the European drug market. In this context, WDAs, refusals, withdrawals and comparisons between application documents and those of approved drugs are particularly interesting for revealing optimization potential in future approval processes. This enables early identification of challenges for drug approval and associated supply gaps in specific indications, thereby promoting a focus in research, development and approval of drugs with unmet medical need. Future analysis of WDAs is also relevant from an economic and health policy perspective, as the timely identification of weaknesses and deficiencies in marketing authorization applications or in the EMA’s own marketing authorization procedure can promote resource-efficient and innovative drug development and approval.

## Conclusion

Between 2006 and 2024 there are 330 WDAs in EMA centralized drug approval procedures. These WDAs are influenced by various factors. Beyond efficacy and clinical trial design, external non-regulatory influences and market interests play a role. To continue promoting ongoing development and innovation in EMA centralized drug approval procedures and the clinical development of new active substances, analysis of approval procedures by central institutions such as EMA and FDA remains highly relevant. Results from this kind of studies enable drug approval procedures to be continuously adapted and optimized in line with developments in active substances, indications, clinical trials and supply requirements.

## Supplementary Information

Below is the link to the electronic supplementary material.

ESM1 (PDF 1.52 MB)

## Data Availability

All original data for this study is available from the authors upon reasonable request.

## Abbreviations

ATC classification system: Anatomical Therapeutic Chemical Classification
CHMP: Committee for Medicinal Products for Human Use
e.g.: For example
EMA: European Medicines Agency
EMEA: European Agency for the Evaluation of Medicinal Products
EU: European Union
FDA: Food and Drug Administration
Fig.: Figure
ICD-11: International Statistical Classification of Diseases and Related Health Problems, 11th Revision
INN: International Nonproprietary Name
N: Number
SHI: Statutory Health Insurance
Tab.: Table
TDM: Therapeutic drug monitoring
WDA: Withdrawn drug application
