# Correction to: Critical analysis of withdrawn drug applications in marketing authorization procedures of the European Medicines Agency (EMA) 2006–2024

**DOI:** 10.1007/s00210-026-05562-9

**Published:** 2026-06-08

**Authors:** Florian Hitsch, Roland Seifert

**Affiliations:** Institute of Pharmacology, Hannover Medical School, Carl‑Neuberg‑Str. 1, D‑30625 Hannover, Germany

**Correction to** **:** **Naunyn-Schmiedeberg’s Archives of Pharmacology**

10.1007/s00210-026-05443-1

During the proofing and production process of this paper, a text section in the abstract was inadvertently deleted. We apologize to our readers.

The original article has been corrected.

