# Supplementary material for: Critical analysis of withdrawn drug applications in marketing authorization procedures of the European Medicines Agency (EMA) 2006–2024
Source: Naunyn Schmiedebergs Arch Pharmacol. 2026 May 22;399(11):17409–21. doi: 10.1007/s00210-026-05443-1 (PMC13503448; doi:10.1007/s00210-026-05443-1)
Supplement: Supplementary file 1 [file 210_2026_5443_MOESM1_ESM.pdf]

Critical analysis of withdrawn drug applications in marketing authorization procedures of the European Medicines Agency (EMA)

(2006-2024)

Florian Hitsch and Roland Seifert

Department of Pharmacology

Hannover Medical School

Germany

Supplementary material

Withdrawn drug applications in scientific literature

Author and year	Title	Number of WDAs	Key messages
The European Agency for the Evaluation of Medicinal Products Human Medicines Evaluation Unit<br><br>May 20, 1999	**Report on withdrawn centralised applications 1995–1998**	30	Describes regulatory and scientific reasons for the withdrawal of marketing authorization applications (EMA). Considers therapeutic areas, quality, efficacy, and safety concerns. The analysis shows that many applications were withdrawn prior to CHMP opinion/EMA statement due to insufficient data or an unfavorable benefit-risk ratio.
The European Agency for the Evaluation of Medicinal Products<br><br>March 15, 2000	**Applications in Centralised Procedure 1995 to July 1999 –an analysis of outcomes**	35 (135 drug applications, 35 withdrawn drug applications)	Analysis of 135 marketing authorization applications in central procedures between 1995 and July 1999. Results show that 28% (38) of the marketing authorization applications had a negative outcome. Withdrawn drug applications are described as an important part of these negative outcomes, especially in the areas of blood, oncology/immunomodulation and nervous system. Main reasons for withdrawals are concerns about efficacy and safety concerns, thus a negative benefit-risk ratio. The report also shows that some of these applications, which were negative in the EU, were approved by the FDA.
Tafuri G. et al.<br><br>2012	**Disclosure of grounds of European withdrawn and refused applications: a step forward on regulatory transparency**	70 of 86 (16 refusals, 70 withdrawn drug applications)	Systematic evaluation of the reports on withdrawn and refused initial marketing authorization applications published by the EMA (2003–2010). Shows that insufficiently efficacy, safety and quality are the main reasons for the failure of marketing authorization applications. Oncology/ immunology and central nervous system were the most common therapeutic categories.
Cramer A. et al.<br><br>2025	**Cancer drug applications to the EMA and the FDA: A comparison of new drugs and extension of indication in terms of approval decisions and time in review**	142 included drug applications (7 withdrawn or refused drug applications)	Comparison of cancer marketing authorization applications to the EMA and FDA. Analyses approved, rejected, and withdrawn applications. Shows that withdrawn drug applications or refusals often arise due to insufficiently convincing efficacy data. In addition, differences in the approval procedures of the EMA and FDA are pointed out.

Table S1 Withdrawn drug applications in scientific literature (Cramer et al. 2025, Tafuri et al. 2012, The European Agency for the Evaluation of Medicinal Products 2000, The European Agency for the Evaluation of Medicinal Products Human Medicines Evaluation Unit 1999)

Table S2 Dataset and analysis criteria of withdrawn drug applications

Name:	International non-proprietary name (INN) / common name:	Active substance:	Pharmaceutical company:	Pharmaceutical company (parent company):	Active substance group:	ATC-codes:<br>Codes taken from EMA table or from database<br>https://atcddd.fhi.no/atc_ddd_index/	Indication according to ICD-11:<br>Source: https://icd.who.int/browse11/l-m/en#/?view=G12	Indication according to ICD-11 (numbering):	Indication:	Type of target structure:	Numbering of the main groups of the target structures:	Superordinated target structures:<br>Source: https://www.guidetopharmacology.org/GRAC/searchPage.jsp2	Numbering of superordinated target structures:	Initial authorisation/ post-authorisation:	Initial authorisation (if revocation after approval):	Date of withdrawal of application (MM/DD/JJ):
Datopotamab deruxtecan Daiichi Sankyo	datopotamab deruxt	Datopotamab deruxtecan	Daiichi Sankyo Europe GmbH	Daiichi Sankyo Europe GmbH	**Monoclonal antibodies, Cytostatics (Antibody-Drug Conjugates)**	**L01FX35**	**02 Neoplasms**	ICD-11 Chapter 02	Adults with non-squamous non-small cell lung cancer (NSCLC), locally advanced or metastatic<br><br>Datopotamab deruxtecan has received a positive recommendation by EMA for the treatment of breast cancer. This medicine was originally known as Datopotamab deruxtecan Daiichi Sankyo but later renamed as Datroway	**Other proteins (Antagonism/Binding) (N5.A)**<br><br>**Enzyme (Antagonism)(N1.A)**	**/N5.A<br>/N1.A<br>//**	other proteins (Tumor-associated antigens, tumor associated calcium signal transducer 2 (TROP2)) (N5.A)<br><br>Enzyme (Isomerases, Antagonism)(N1.5.A)	**/N5.A<br>/N1.5.A<br>//**	**Initial authorisation**		12/20/24
Ngenla	somatrogon	Somatrogon	Pfizer Europe MA EEIG	Pfizer	**Other medicines**	**H01AC08**	**05 Endocrine diseases, nutritional or metabolic diseases**<br><br>**21 Symptoms, signs or clinical findings not elsewhere classified (short stature)**	ICD-11 Chapter 05<br><br>ICD-11 Chapter 21	Adults with growth hormone deficiency<br><br>**already approved indications:**<br>Growth hormone deficiency for patients from 3 years of age	**Receptors (Agonism) (N4.B)**	**/N4.B<br>//**	Receptors (catalytic receptors, Agonism) (N4.3.B)	**/N4.3.B<br>//**	**Post-authorisation**	2/14/22	12/20/24
Nugalviq	govorestat	Govorestat	Advanz Pharma Limited	ADVANZ PHARMA Limited	**Other medicines**	**A16AX24**	**05 Endocrine diseases, nutritional or metabolic diseases**	ICD-11 Chapter 05	Adults and children aged two years and above with confirmed diagnosis of classic galactosemia	**Enzyme (Antagonism)(N1.A)**	**/N1.A<br>//**	Enzyme (Oxidoreductases, Antagonism)(N1.1.A)	**/N1.1.A<br>//**	**Initial authorisation**		12/10/24
Izelvay	avacincaptad pegol	Avacincaptad pegol	Astellas Pharma Europe B.V.	Astellas Pharma Europe B. V.	**Other medicines**	**S01XA32**	**09 Diseases of the visual system**	ICD-11 Chapter 09	Macular Degeneration;Geographic Atropl	**Ligands (antagonism)(N9.A)**	**/N9.A<br>//**	Ligands (Antagonism) (N9.A)	**/N9.A<br>//**	**Initial authorisation**		10/24/24
Inaqovi	cedazuridine;decitał	Cedazuridine;Decitał	Otsuka Pharmaceutical Netherlands B.V.	Otsuka Pharmaceutical Netherlands B.V.	**Cytostatics**	**L01BC58**	**02 Neoplasms**	ICD-11 Chapter 02	Myelodysplastic syndromes (MDS) or chronic myelomonocytic leukaemia (CMML)<br><br>**Already approved indications:**<br>Acute myeloid leukaemia (AML)	**Enzyme (Antagonism)(N1.A)**	**/N1.A<br>//**	Enzyme (Hydrolases, Antagonism)(N1.3.A)<br><br>Enzyme (Transferases, Antagonism)(N1.2.A)	**/N1.3.A<br>/N1.2.A<br>//**	**Post-authorisation**	9/15/23	11/6/24
Syfovre	pegcetacoplan	Pegcetacoplan	Apellis Netherlands B.V.	Apellis Netherlands B.V.	**Other medicines**	**S01XA31**	**09 Diseases of the visual system**	ICD-11 Chapter 09	Macular Degeneration;Geographic Atropl	**Ligands (antagonism)(N9.A)**	**/N9.A<br>//**	Ligands (Antagonism) (N9.A)	**/N9.A<br>//**	**Initial authorisation**		10/4/24
Epixram	levetiracetam	Levetiracetam	Neuraxpharm Pharmaceuticals S.L.	Neuraxpharm Pharmaceuticals S.L.	**Antiepileptics**	**N03AX14**	**08 Diseases of the nervous system**	ICD-11 Chapter 08	Epilepsy;Generalized Epilepsy;Juvenile M	**Transporter (Antagonism)(N3.A)**	**/N3.A<br>//**	Transporter (SLC-Transporters, Antagonism)(N3.1.A)	**/N3.1.A<br>//**	**Initial authorisation**		9/19/24
Apremilast Viatris	apremilast	Apremilast	Viatris Limited	Viatris Limited	**Immunosuppressa**	**L04AA32**	**15 Diseases of the musculoskeletal system or connective tissue/**<br><br>**14 Skin diseases/**<br><br>**04 Diseases of the immune system**	ICD-11 Chapter 15<br><br>ICD-11 Chapter 14<br><br>ICD-11 Chapter 04	Psoriatic Arthritis; Psoriasis; Behcet Sync	**Enzyme (Antagonism)(N1.A)**	**/N1.A<br>//**	Enzyme (Hydrolases, Antagonism)(N1.3.A)	**/N1.3.A<br>//**	**Initial authorisation**		9/17/24
Tecentriq	atezolizumab	Atezolizumab	Roche Registration GmbH	Roche Registration GmbH	**Monoclonal antibodies**	**L01XC32**	**02 Neoplasms**	ICD-11 Chapter 02	Expansion of use for the treatment of hepatocellular carcinoma after removal with high risk of recurrence. Treatment in combination with Bevacizumab.<br><br>**already approved indications:**<br>urothelial carcinoma,<br>lung carcinoma (small cell, non-small cell);<br>hepatocellular carcinoma; breast cancer (triple-negative)	**Ligands (antagonism)(N9.A)**	**/N9.A<br>//**	Ligands (Antagonism/Binding) (N9.A)	**/N9.A<br>//**	**Post-authorisation**	9/20/17	9/6/24

Name:	EMA (CHMP) opinions:	Numbering of EMA (CHMP) opinions:	Publication of the revocation (MM/DD/JJ):	EMA recommendations:	Procedure of applicant (manufacturer):<br>Reasons for withdrawal of the application:	number of clinical studies:	number of participants in clinical studies:	Clinical comparisons (with placebo):	Additional supportive data (SD):<br>Supportive data generic medicinal products (SDG)<br>Supportive data biosimilars (SDB)<br>Supportive data hybrid medicinal products (SDH)	Clinical comparisons (with approved drugs):	Special drug approval procedures:<br>Biosimilar<br>Generic medicine<br>Hybrid medicine<br>Hybrid and generic medicine<br>no special drug approval procedure	Consequences for patients in ongoing clinical trials or compassionate use programmes	Ongoing clinical trials (at the time the application was withdrawn)	Source:	Source withdrawal letter:	Orphan drugs:	Last update(EMA website, MM/DD/JJ):
Datopotamab deruxtecan Daiichi Sankyo	• open questions from the EMA (a17)<br>• concerns regarding responses/information from the manufacturer (a1)<br>• doubts about efficacy<br>reason: lack of data to sufficiently demonstrate efficacy (a27.3)<br>• doubts about assurance of patient safety<br>reason: adverse events (a21.1)<br>• doubts about efficacy<br>reason: poor study quality (change of study design during the study, inadequate efficacy indicators) (a27.1.1)	**/A17<br>/A1<br>/A27.3<br>/A21.1<br>/A27.1.1<br>//**	1/31/25	**No authorisation, because of no positive benefit-risk ratio**	**EMA evaluation**	**Number of clinical studies 01**	**Participants 0500-0999**	**No placebo control**	**SD**	**Comparison with approved drugs**	**No special drug approval procedure**	**No consequences for patients in ongoing clinical trials or compassionate use programmes**	**Ongoing clinical trials (at the time the application was withdrawn)**	https://www.ema.europa.eu/en/documents/medicine-qa/questions-answers-withdrawal-application-marketing-authorisation-datopotamab-deruxtecan-daiichi-sankyo-datopotamab-deruxtecan_en.pdf	https://www.ema.europa.eu/en/documents/withdrawal-letter/withdrawal-letter-datopotamab-deruxtecan-daiichi-sankyo_en.pdf	**No orphan drugs**	1/31/25
Ngenla	• open questions from the EMA (a17)<br>• concerns regarding responses/information from the manufacturer (a1)<br>• doubts about efficacy<br>reason: lack of data to sufficiently demonstrate efficacy (efficacy versus placebo not sufficient) (a27.3)	**/A17<br>/A1<br>/A27.3<br>//**	4/25/25	**No extension of authorisation, because of no positive benefit-risk ratio**	**Development strategy**	**Number of clinical studies 01**	**Participants 0200-0299**	**Placebo controlled clinical studies**		**No comparison with approved drugs**	**No special drug approval procedure**	**No consequences for patients in ongoing clinical trials or compassionate use programmes**	**Ongoing clinical trials (at the time the application was withdrawn)**	https://www.ema.europa.eu/en/documents/medicine-qa/questions-answers-withdrawal-application-change-marketing-authorisation-ngenla-somatrogon-ii-16_en.pdf	https://www.ema.europa.eu/en/documents/withdrawal-letter/withdrawal-letter-ngenla-ii-16_en.pdf	**Orphan drugs**	4/25/25
Nugalviq	• concerns regarding responses/information from the manufacturer (a1)<br>• doubts about efficacy<br>reason: poor study quality (data analysis) (a27.1.3)<br>• doubts about efficacy<br>reason: poor study quality (change of study design during the study, inadequate efficacy indicators) (a27.1.1)<br>• doubts about assurance of patient safety<br>reason: adverse events (a21.1)<br>• doubts about efficacy<br>reason: poor study quality (conduct) (a27.1.6)<br>• concerns regarding drug quality (a3)<br>• concerns regarding recommended dose (a6)	**/A1<br>/A27.1.3<br>/A27.1.1<br>/A21.1<br>/A27.1.6<br>/A3<br>/A6<br>//**	1/31/25	**No authorisation, because of no positive benefit-risk ratio**	**EMA evaluation**	**Number of clinical studies 02**	**Participants 0075-0099**	**Placebo controlled clinical studies**	**SD**	**No comparison with approved drugs**	**No special drug approval procedure**	**No consequences for patients in ongoing clinical trials or compassionate use programmes**	**Ongoing clinical trials (at the time the application was withdrawn)**	https://www.ema.europa.eu/en/documents/medicine-qa/questions-answers-withdrawal-application-marketing-authorisation-nugalviq-govorestat_en.pdf	https://www.ema.europa.eu/en/documents/withdrawal-letter/withdrawal-letter-nugalviq_en.pdf	**Orphan drugs**	1/31/25
Izelvay	• open questions from the EMA (a17)<br>• concerns regarding responses/information from the manufacturer (a1)<br>• doubts about benefit (benefit too small) (a18)<br>• doubts about assurance of patient safety<br>reason: adverse events (a21.1)	**/A17<br>/A1<br>/A18<br>/A21.1<br>//**	11/15/24	**No authorisation, because of no positive benefit-risk ratio**	**EMA evaluation**	**Number of clinical studies 02**	**Participants 0500-0999**	**Placebo controlled clinical studies**		**No comparison with approved drugs**	**No special drug approval procedure**	**no consequences for patients in ongoing clinical trials or compassionate use programmes**	**Ongoing clinical trials (at the time the application was withdrawn)**	https://www.ema.europa.eu/en/documents/medicine-qa/questions-answers-withdrawal-application-marketing-authorisation-izelvay_en.pdf	https://www.ema.europa.eu/en/documents/withdrawal-letter/withdrawal-letter-izelvay_en.pdf	**No orphan drugs**	2/4/25
Inaqovi	• concerns regarding responses/information from the manufacturer (a1)<br>• doubts about benefit (benefit not sufficiently demonstrated) (a18)	**/A1<br>/A18<br>//**	11/15/24	**No authorisation, because of no positive benefit-risk ratio**	**EMA evaluation**	**Number of clinical studies 02**	**Participants unknown**	**No placebo control**		**Comparison with approved drugs**	**No special drug approval procedure**	**No consequences for patients in ongoing clinical trials or compassionate use programmes**	**Ongoing clinical trials (at the time the application was withdrawn)**	https://www.ema.europa.eu/en/documents/medicine-qa/questions-answers-withdrawal-application-marketing-authorisation-inaqovi-ii-0002_en.pdf	https://www.ema.europa.eu/en/documents/withdrawal-letter/withdrawal-letter-inaqovi-ii-0002-myelodysplastic-syndromes_en.pdf<br><br>https://www.ema.europa.eu/en/documents/withdrawal-letter/withdrawal-letter-inaqovi-ii-0002-cmml_en.pdf	**No orphan drugs**	6/17/25
Syfovre	• doubts about assurance of patient safety<br>reason: adverse events (a21.1)<br>• doubts about benefit (benefit too small) (a18)	**/A21.1<br>/A18<br>//**	8/11/25	**No authorisation, because of no positive benefit-risk ratio**	**EMA evaluation**	**Number of clinical studies 02**	**Participants 1000-1999**	**Placebo controlled clinical studies**		**No comparison with approved drugs**	**No special drug approval procedure**	**No consequences for patients in ongoing clinical trials or compassionate use programmes**	**Ongoing clinical trials (at the time the application was withdrawn)**	https://www.ema.europa.eu/en/documents/medicine-qa/questions-answers-withdrawal-application-marketing-authorisation-syfovre-pegcetacoplan_en.pdf	https://www.ema.europa.eu/en/documents/withdrawal-letter/withdrawal-letter-syfovre_en.pdf	**No orphan drugs**	8/14/25
Epixram	• open questions from the EMA (a17)<br>• concerns regarding responses/information from the manufacturer (a1)<br>• open questions on bioavailability, bioequivalence, distribution and metabolism (one or more topics) (a29)	**/A17<br>/A1<br>/A29<br>//**	10/18/24	**No authorisation, because of no positive benefit-risk ratio**	**Development strategy**	**Number of clinical studies 0**	**Participants 0**	**No placebo control**	**SDH**	**Comparison with approved drugs**	**Drug application for hybrid medicines**	**no consequences for patients in ongoing clinical trials or compassionate use programmes**	**No ongoing clinical trials (at the time the application was withdrawn)**	https://www.ema.europa.eu/en/documents/medicine-qa/questions-answers-withdrawal-application-marketing-authorisation-epixram_en.pdf	https://www.ema.europa.eu/en/documents/withdrawal-letter/withdrawal-letter-epixram_en.pdf	**No orphan drugs**	1/9/25
Apremilast Viatris	• open questions from the EMA (a17)<br>• concerns regarding responses/information from the manufacturer (a1)<br>• open questions on bioavailability, bioequivalence, distribution and metabolism (one or more topics) (a29)	**/A17<br>/A1<br>/A29<br>//**	10/18/24	**No authorisation, because of no positive benefit-risk ratio**	**EMA evaluation**	**Number of clinical studies 0**	**Participants 0**	**No placebo control**	**SDG**	**Comparison with approved drugs**	**Drug application for generic medicines**	**no consequences for patients in ongoing clinical trials or compassionate use programmes**	**Ongoing clinical trials (at the time the application was withdrawn)**	https://www.ema.europa.eu/en/documents/medicine-qa/questions-answers-withdrawal-application-marketing-authorisation-apremilast-viatris-apremilast_en.pdf	https://www.ema.europa.eu/en/documents/withdrawal-letter/withdrawal-letter-apremilast-viatris_en.pdf	**No orphan drugs**	1/6/25
Tecentriq	• open questions from the EMA (a17)<br>• concerns regarding responses/information from the manufacturer (a1)<br>• doubts about efficacy<br>reason: lack of data to sufficiently demonstrate efficacy (related to indication in extension application, missing data on overall survival) (a27.3)<br>• doubts about benefit (clinical relevance) (a18)<br>• doubts about assurance of patient safety (lack of data on deaths) (a21)<br>• concerns regarding requested indication (indication should have related to completely resected tumor disease) (a28)	**/A17<br>/A1<br>/A27.3<br>/A18<br>/A21<br>/A28<br>//**	9/20/24	**No extension of authorisation, because of no positive benefit-risk ratio**	**EMA evaluation**	**Number of clinical studies 01**	**Participants 0500-0999**	**No placebo control**		**No comparison with approved drugs**	**No special drug approval procedure**	**Consequences for patients in ongoing clinical trials or compassionate use programmes**	**Ongoing clinical trials (at the time the application was withdrawn)**	https://www.ema.europa.eu/en/documents/medicine-qa/questions-answers-withdrawal-application-marketing-authorisation-tecentriq-ii-81_en.pdf	https://www.ema.europa.eu/en/documents/withdrawal-letter/withdrawal-letter-tecentriq-ii-81_en.pdf	**No orphan drugs**	9/20/24

Sialanar	glycopyrronium bromide	Glycopyrronium bromide	Proveca Pharma Limited	Proveca Pharma Limited	**Anticholinergics**	**A03AB02**	**13 Diseases of the digestive system**	ICD-11 Chapter 13	Children from 2 years of age with chronic<br>conditions affecting the nervous system.<br><br>**already approved indications:**<br>severe saliva drooling in children and adolescents aged 3 years and<br>above with chronic conditions affecting the nervous system, such as cerebral palsy,<br>epilepsy and neurodegenerative diseases.	**Receptors (Antagonism) (N4.A)**	**/N4.A<br>//**	Receptors<br>(GPCR, Antagonism) (N4.1.A)	**/N4.1.A<br>//**	**Post-authorisation**	9/15/16	9/6/24	• open questions from the EMA (a17)<br>• concerns regarding responses/information from the manufacturer (a1)<br>• doubts about assurance of patient safety (a21)<br>• doubts about efficacy<br>reason: poor study quality (a27.1)<br>• doubts about efficacy<br>reason: inadequate study design (a27.2)<br>• doubts about efficacy<br>reason: patients in the study not compatible with target indication (a27.4)	**/A17<br>/A1<br>/A21<br>/A27.1<br>/A27.2<br>/A27.4<br>//**	9/20/24	**No extension of authorisation, because of no positive benefit-risk ratio**	**EMA evaluation**	**Number of clinical studies 02**	**Participants 0100-0149**	**Placebo controlled clinical studies**	**SD**	**Comparison with approved drugs**	**No special drug approval procedure**	**No consequences for patients in ongoing clinical trials or compassionate use programmes**	**Ongoing clinical trials (at the time the application was withdrawn)**	https://www.ema.europa.eu/en/documents/medicine-qa/questions-answers-withdrawal-application-change-marketing-authorisation-sialanar-glycopyrronium-bromide_en.pdf	https://www.ema.europa.eu/en/documents/withdrawal-letter/withdrawal-letter-sialanar-ii-29_en.pdf	**No orphan drugs**	9/20/24
Durysta	bimatoprost	Bimatoprost	AbbVie Deutschland GmbH & Co. KG	AbbVie Ltd.	**Other medicines**	**S01EE03**	**09 Diseases of the visual system**	ICD-11 Chapter 09	Open Angle Glaucoma; Ocular Hypertens	**Receptors (Agonism) (N4.B)**	**/N4.B<br>//**	Receptors<br>(GPCR, Agonism) (N4.1.B)	**/N4.1.B<br>//**	**Initial authorisation**		9/13/24	• open questions from the EMA (a17)<br>• concerns regarding responses/information from the manufacturer (a1)<br>• concerns regarding drug quality (impurities and/or concerns regarding sterilization) (a3.3)<br>• doubts about assurance of patient safety<br>reason: adverse events (a21.1)	**/A17<br>/A1<br>/A3.3<br>/A21.1<br>//**	9/19/24	**No authorisation, because of no positive benefit-risk ratio**	**EMA evaluation**	**Number of clinical studies 02**	**Participants 0300-0499**	**No placebo control**		**Comparison with approved drugs**	**No special drug approval procedure**	**no consequences for patients in ongoing clinical trials or compassionate use programmes**	**Ongoing clinical trials (at the time the application was withdrawn)**	https://www.ema.europa.eu/en/documents/medicine-qa/questions-answers-withdrawal-application-marketing-authorisation-durysta-bimatoprost-intracameral-implant_en.pdf	https://www.ema.europa.eu/en/documents/withdrawal-letter/withdrawal-letter-durysta_en.pdf	**No orphan drugs**	12/17/24
Dabigatran etexilate	dabigatran etexilate	Dabigatran etexilate	Teva GmbH	Teva Pharma B.V	**Anticoagulants**	**B01AE07**	**11 Diseases of the circulatory system**	ICD-11 Chapter 11	Venous Thromboembolism, Prevention of	**Enzyme (Antagonism)(N1.A)**	**/N1.A<br>//**	Enzyme<br>(Hydrolases, Antagonism)(N1.3.A)	**/N1.3.A<br>//**	**Initial authorisation**		6/13/24	• open questions from the EMA (a17)<br>• concerns regarding responses/information from the manufacturer (a1)<br>• concerns regarding drug quality (impurities and/or concerns regarding sterilization) (a3.3)	**/A17<br>/A1<br>/A3.3<br>//**	6/28/24	**No authorisation, because of no positive benefit-risk ratio**	**Marketing strategy**	**Number of clinical studies 0**	**Participants 0**	**No placebo control**	**SDG**	**Comparison with approved drugs**	**Drug application for generic medicines**	**no consequences for patients in ongoing clinical trials or compassionate use programmes**	**No ongoing clinical trials (at the time the application was withdrawn)**	https://www.ema.europa.eu/en/documents/medicine-qa/questions-answers-withdrawal-application-marketing-authorisation-dabigatran-etexilate-teva_en.pdf	https://www.ema.europa.eu/en/documents/withdrawal-letter/withdrawal-letter-dabigatran-etexilate-teva_en.pdf	**No orphan drugs**	6/28/24
Kinharto	omecamtiv mecarbil	Omecamtiv mecarbil	Cytokinetics (Ireland) Limited	Cytokinetics (Ireland) Limited	**Other medicines**	**C01CX10**	**11 Diseases of the circulatory system**	ICD-11 Chapter 11	Systolic Heart Failure	**other proteins (Agonism) (N5.B)**	**/N5.B<br>//**	other proteins (Agonism) (N5.B)	**/N5.B<br>//**	**Initial authorisation**		5/7/24	• open questions from the EMA (a17)<br>• concerns regarding responses/information from the manufacturer (a1)<br>• doubts about benefit (benefit too small) (a18)<br>• doubts about efficacy<br>reason: lack of data to sufficiently demonstrate efficacy (a27.3)	**/A17<br>/A1<br>/A18<br>/A27.3<br>//**	5/31/24	**No authorisation, because of no positive benefit-risk ratio**	**EMA evaluation**	**Number of clinical studies 01**	**Participants over 2000**	**Placebo controlled clinical studies**		**No comparison with approved drugs**	**No special drug approval procedure**	**no consequences for patients in ongoing clinical trials or compassionate use programmes**	**No ongoing clinical trials (at the time the application was withdrawn)**	https://www.ema.europa.eu/en/documents/medicine-qa/questions-answers-withdrawal-application-marketing-authorisation-kinharto-omecamtiv-mecarbil_en.pdf	https://www.ema.europa.eu/en/documents/withdrawal-letter/withdrawal-letter-kinharto_en.pdf	**No orphan drugs**	9/30/24
Scenesse	afamelanotide	Afamelanotide	Clinuvel Europe Limited	Clinuvel Europe Limited	**Other medicines**	**D02BB02**	**05 Endocrine diseases, nutritional or metabolic diseases**	ICD-11 Chapter 05	Adolescents aged 12 to 17 years with EPP. During the assessment, the company modified the request to adolescents from 15 to 17 years and weighing more than 60 kg.<br><br>**already approved indications:**<br>Erythropoietic protoporphyria	**Receptors (Agonism) (N4.B)**	**/N4.B<br>//**	Receptors<br>(GPCR, Agonism) (N4.1.B)	**/N4.1.B<br>//**	**Post-authorisation**	12/22/14	4/24/24	• open questions from the EMA (a17)<br>• concerns regarding responses/information from the manufacturer (a1)<br>• open questions on bioavailability, bioequivalence, distribution and metabolism (one or more topics) (a29)<br>• concerns regarding recommended dose (a6)<br>• doubts about assurance of patient safety<br>reason: lack of data and/or inadequate long-term observation (a21.3)<br>• doubts about efficacy<br>reason: lack of data to sufficiently demonstrate efficacy (a27.3)	**/A17<br>/A1<br>/A29<br>/A6<br>/A21.3<br>/A27.3<br>//**	5/31/24	**No extension of authorisation, because of no positive benefit-risk ratio**	**EMA evaluation**	**Number of clinical studies 01**	**Participants 0001-0024**	**No placebo control**		**No comparison with approved drugs**	**No special drug approval procedure**	**No consequences for patients in ongoing clinical trials or compassionate use programmes**	**Ongoing clinical trials (at the time the application was withdrawn)**	https://www.ema.europa.eu/en/documents/medicine-qa/questions-answers-withdrawal-application-change-marketing-authorisation-scenesse-afamelanotide_en.pdf	https://www.ema.europa.eu/en/documents/withdrawal-letter/withdrawal-letter-scenesse_en.pdf	**No orphan drugs**	5/31/24
GeGant	germanium (68Ge) c	Gallium (68Ga) Gern	ITM Medical Isotopes GmbH	ITM Medical Isotopes GmbH	**Other medicines**	**V09X**	**X Extension codes (Health Devices, Equipment and Supplies)**	ICD-11 Chapter X	Radionuclide Imaging	**other (N6)**	**/N6.U<br>//**	other (N6)	**/N6.U<br>//**	**Initial authorisation**		4/15/24	• open questions from the EMA (a17)<br>• concerns regarding responses/information from the manufacturer (a1)<br>• concerns regarding drug quality (impurities and/or concerns regarding sterilization) (a3.3)<br>• eu gmp (good manufacturing practice) certificate not available (a8)	**/A17<br>/A1<br>/A3.3<br>/A8<br>//**	4/26/24	**No authorisation, because of no positive benefit-risk ratio**	**EMA evaluation**	**Number of clinical studies 0**	**Participants 0**	**No placebo control**	**SD**	**No comparison with approved drugs**	**No special drug approval procedure**	**No consequences for patients in ongoing clinical trials or compassionate use programmes**	**No ongoing clinical trials (at the time the application was withdrawn)**	https://www.ema.europa.eu/en/documents/medicine-qa/questions-answers-withdrawal-application-marketing-authorisation-gegant-germanium-68ge-chloride-gallium-68ga-chloride_en.pdf	https://www.ema.europa.eu/en/documents/withdrawal-letter/withdrawal-letter-gegant_en.pdf	**No orphan drugs**	2/26/25
Upstelda	ustekinumab	Ustekinumab	Amgen Technology (Ireland) UC	Amgen Europe B.V.	**Monoclonal antibodies**	**L04AC05**	**15 Diseases of the musculoskeletal system or connective tissue/**<br><br>**14 Skin diseases/**<br><br>**13 Diseases of the digestive system**	ICD-11 Chapter 15<br><br>ICD-11 Chapter 14<br><br>ICD-11 Chapter 13	Psoriasis; Psoriatic Arthritis; Crohn Disea	**Ligands (antagonism)(N9.A)**	**/N9.A<br>//**	Ligands<br>(Cytokines, Antagonism)(N9.2.A)	**/N9.2.A<br>//**	**Initial authorisation**		3/22/24	• not available<br>(documents have not yet been evaluated or recommendations not yet published) (a14.1)	**/A14.1<br>//**	7/30/24	**None (documents have not yet been evaluated)**	**Marketing strategy**	**Number of clinical studies unknown**	**Participants unknown**	**placebo control unknown**		**Comparison with approved drugs unknown**	**No special drug approval procedure**	**no consequences for patients in ongoing clinical trials or compassionate use programmes**	**Ongoing clinical trials unknown (at the time the application was withdrawn)**	nicht Verfügbar (Antrag ist Duplikat von Upstelda)	https://www.ema.europa.eu/en/documents/withdrawal-letter/withdrawal-letter-upstelda_en.pdf	**No orphan drugs**	7/30/24
Adcetris	Brentuximab vedotin	Brentuximab vedotin	Takeda Pharma A/S	Takeda	**Monoclonal antibodies, Cytostatics (Antibody-Drug Conjugates)**	**L01XC12**	**02 Neoplasms**	ICD-11 Chapter 02	Adults with CD30-positive peripheral T-cell<br>lymphoma not otherwise specified (PTCL-NOS), when the cancer has not been treated before (use in combination with cyclophosphamide, doxorubicin and prednisone)<br><br>**already approved indications:**<br>Hodgkin's lymphoma, non-Hodgkin's lymphoma (cutaneous T-cell lymphoma (CTCL), systemic anaplastic large cell lymphoma (sALCL))	**Receptors (Antagonism) (N4.A)**	**/N4.A<br>//**	Receptors<br>(catalytic receptors, Antagonism) (N4.3.A)	**/N4.3.A<br>//**	**Post-authorisation**	10/25/12	2/23/24	• concerns regarding responses/information from the manufacturer (a1)<br>• doubts about efficacy<br>reason: poor study quality (selection of participants) (a27.1.7)<br>• doubts about efficacy<br>reason: poor study quality (small participant number) (a27.1.5)<br>• doubts about efficacy<br>reason: lack of data to sufficiently demonstrate efficacy (a27.3)	**/A1<br>/A27.1.7<br>/A27.1.5<br>/A27.3<br>//**	3/22/24	**No extension of authorisation, because of no positive benefit-risk ratio**	**EMA evaluation**	**Number of clinical studies 01**	**Participants 0300-0499**	**No placebo control**		**Comparison with approved drugs**	**No special drug approval procedure**	**no consequences for patients in ongoing clinical trials or compassionate use programmes**	**Ongoing clinical trials (at the time the application was withdrawn)**	https://www.ema.europa.eu/en/documents/medicine-qa/questions-answers-withdrawal-application-change-marketing-authorisation-adcetris-brentuximab-vedotin_en.pdf	https://www.ema.europa.eu/en/documents/withdrawal-letter/withdrawal-letter-adcetris-ii-109_en.pdf	**Orphan drugs**	7/18/24
Orencia	abatacept	Abatacept	Bristol-Myers Squibb Pharma EEIG	Bristol-Myers Squibb Pharma EEIG	**Immunosuppressants (no monoclonal antibodies)**	**L04AA24**	**04 Diseases of the immune system**	ICD-11 Chapter 04	Prevention of acute graft-versus-host disease in adults and children from 2 years of age with cancers that affect blood cells<br><br>**already approved indications:**<br>rheumatoid arthritis, polyarticular juvenile idiopathic arthritis	**other proteins (Antagonism/Binding) (N5.A)**	**/N4.A<br>//**	other proteins<br>(Immune checkpoint proteins/CD-molecules, Antagonism/Binding) (N5.4.A)	**/N5.4.A<br>//**	**Post-authorisation**	5/21/07	2/19/24	• open questions from the EMA (a17)<br>• concerns regarding responses/information from the manufacturer (a1)<br>• doubts about efficacy<br>reason: lack of data to sufficiently demonstrate efficacy (a27.3)<br>• doubts about assurance of patient safety<br>reason: lack of data and/or inadequate long-term observation (a21.3)<br>• doubts about assurance of patient safety<br>reason: adverse events (a21.1)	**/A17<br>/A1<br>/A27.3<br>/A21.3<br>/A21.1<br>//**	3/22/24	**No extension of authorisation, because of no positive benefit-risk ratio**	**EMA evaluation**	**Number of clinical studies 01**	**Participants 0150-0199**	**Placebo controlled clinical studies**		**No comparison with approved drugs**	**No special drug approval procedure**	**no consequences for patients in ongoing clinical trials or compassionate use programmes**	**Ongoing clinical trials (at the time the application was withdrawn)**	https://www.ema.europa.eu/en/documents/medicine-qa/questions-answers-withdrawal-application-change-marketing-authorisation-orencia-abatacept_en.pdf	https://www.ema.europa.eu/en/documents/withdrawal-letter/withdrawal-letter-orencia-ii-152_en.pdf	**No orphan drugs**	4/30/24
Ongentys	opicapone	Opicapone	Bial Portela & Companhia S.A.	Bial Portela & Companhia S.A.	**Parkinson's therapeutics**	**N04**	**08 Diseases of the nervous system**	ICD-11 Chapter 08	Signs and symptoms of Parkinson's disease (in addition to levodopa / DDCI), including patients with early stage Parkinson's disease. It was no longer to be restricted to<br>patients having (motor) fluctuations in the the control of their condition.<br><br>**already approved indications:**<br>Parkinson Disease	**Enzyme (Antagonism)(N1.A)**	**/N1.A<br>//**	Enzyme<br>(Transferases, Antagonism)(N1.2.A)	**/N1.2.A<br>//**	**Post-authorisation**	6/24/16	3/12/24	• open questions from the EMA (a17)<br>• concerns regarding responses/information from the manufacturer (a1)<br>• doubts about efficacy<br>reason: lack of data to sufficiently demonstrate efficacy (a27.3)<br>• doubts about benefit (clinical benefit unclear) (a18)	**/A17<br>/A1<br>/A27.3<br>/A18<br>//**	3/22/24	**No extension of authorisation, because of no positive benefit-risk ratio**	**EMA evaluation**	**Number of clinical studies 01**	**Participants 0300-0499**	**Placebo controlled clinical studies**		**No comparison with approved drugs**	**No special drug approval procedure**	**No consequences for patients in ongoing clinical trials or compassionate use programmes**	**Ongoing clinical trials (at the time the application was withdrawn)**	https://www.ema.europa.eu/en/documents/medicine-qa/questions-answers-withdrawal-application-change-marketing-authorisation-ongentys-opicapone_en.pdf	https://www.ema.europa.eu/en/documents/withdrawal-letter/withdrawal-letter-ongentys-ws2552-60_en.pdf	**No orphan drugs**	9/18/24
Ontilyv	opicapone	Opicapone	Bial Portela & Companhia S.A.	Bial Portela & Companhia S.A.	**Parkinson's therapeutics**	**N04**	**08 Diseases of the nervous system**	ICD-11 Chapter 08	Signs and symptoms of Parkinson's disease (in addition to levodopa / DDCI), including patients with early stage Parkinson's disease. It was no longer to be restricted to<br>patients having (motor) fluctuations in the the control of their condition.<br><br>**already approved indications:**<br>Parkinson Disease	**Enzyme (Antagonism)(N1.A)**	**/N1.A<br>//**	Enzyme<br>(Transferases, Antagonism)(N1.2.A)	**/N1.2.A<br>//**	**Post-authorisation**	2/21/22	3/12/24	• open questions from the EMA (a17)<br>• concerns regarding responses/information from the manufacturer (a1)<br>• doubts about efficacy<br>reason: lack of data to sufficiently demonstrate efficacy (a27.3)<br>• doubts about benefit (clinical benefit unclear) (a18)	**/A17<br>/A1<br>/A27.3<br>/A18<br>//**	3/22/24	**No extension of authorisation, because of no positive benefit-risk ratio**	**EMA evaluation**	**Number of clinical studies 01**	**Participants 0300-0499**	**Placebo controlled clinical studies**		**No comparison with approved drugs**	**No special drug approval procedure**	**No consequences for patients in ongoing clinical trials or compassionate use programmes**	**Ongoing clinical trials (at the time the application was withdrawn)**	https://www.ema.europa.eu/en/documents/medicine-qa/questions-answers-withdrawal-application-change-marketing-authorisation-ontilyv-opicapone_en.pdf	https://www.ema.europa.eu/en/documents/withdrawal-letter/withdrawal-letter-ontilyv-ws-2552_en.pdf	**No orphan drugs**	9/18/24
Bylvay	odevixibat	Odevixibat	Ipsen Pharma	Ipsen Pharma	**Other medicines**	**A05AX**	**14 Skin diseases**	ICD-11 Chapter 14	cholestatic pruritus in Alagille syndrome (ALGS).<br><br>Already approved indications:<br>progressive familial intrahepatic cholestasis (PFIC)	**Transporter (Antagonism)(N3.A)**	**/N3.A<br>//**	Transporter<br>(SLC-Transporters, Antagonism)(N3.1.A)	**/N3.1.A<br>//**	**Post-authorisation**	7/16/21	10/22/23	• not available (marketing authorisation was recommended) (a14.2)	**/A14.2<br>//**	11/20/23	**Authorisation recommended**	**Other reasons**	**Number of clinical studies 01**	**Participants 0050-0074**	**Placebo controlled clinical studies**		**No comparison with approved drugs**	**No special drug approval procedure**	**No consequences for patients in ongoing clinical trials or compassionate use programmes**	Ongoing clinical trials (at the time the application was withdrawn)	https://www.ema.europa.eu/en/documents/medicine-qa/questions-and-answers-withdrawal-application-change-marketing-authorisation-bylvay-odevixibat_en.pdf	https://www.ema.europa.eu/en/documents/withdrawal-letter/withdrawal-letter-bylvay-ii-11_en.pdf-0	**Orphan drugs**	11/23/23
Vijoice	alpelisib	Alpelisib	Novartis Europharm Limited	Novartis	**Kinase inhibitors**	**L01EM03**	**20 Developmental anomalies**	ICD-11 Chapter 20	severe<br>or life-threatening PROS symptoms (PIK3CA-related<br>overgrowth spectrum, PROS) in adults and children from 2 years	**Enzyme (Antagonism)(N1.A)**	**/N1.A<br>//**	Enzyme<br>(Transferases, Antagonism)(N1.2.A)	**/N1.2.A<br>//**	**Initial authorisation**		10/30/23	• open questions from the EMA (a17)<br>• concerns regarding responses/information from the manufacturer (a1)<br>• doubts about efficacy<br>reason: lack of data to sufficiently demonstrate efficacy (a27.3)<br>• doubts about assurance of patient safety<br>reason: lack of data and/or inadequate long-term observation (a21.3)	**/A17<br>/A1<br>/A27.3<br>/A21.3<br>//**	11/10/23	**No authorisation, because of no positive benefit-risk ratio**	**EMA evaluation**	**Number of clinical studies 01**	**Participants 0050-0074**	**placebo control unknown**		**Comparison with approved drugs unknown**	**No special drug approval procedure**	**No consequences for patients in ongoing clinical trials or compassionate use programmes**	Ongoing clinical trials (at the time the application was withdrawn)	https://www.ema.europa.eu/en/documents/medicine-qa/questions-answers-withdrawal-application-marketing-authorisation-vijoice-alpelisib_en.pdf	https://www.ema.europa.eu/en/documents/withdrawal-letter/withdrawal-letter-vijoice_en.pdf	**Orphan drugs**	3/14/24

Sugammadex Lorien	unknown	Sugammadex	LABORATORIOS LORIEN, S.L	LABORATORIOS LORIEN, S.L	**Antidote**	**V03AB35**	**X Extension codes (Antidotes)**	ICD-11 Chapter X	Reversal of the effects of muscle relaxants<br>Rocuronium and Vecuronium	**other (N6)**	**/N6.U<br>//**	other (N6)	**/N6.U<br>//**	**Initial authorisation**		9/28/23	• open questions from the EMA (a17)<br>• concerns regarding drug quality (manufacturing process) (a3.1)	**/A17<br>/A3.1<br>//**	10/13/23	**No authorisation, because of no positive benefit-risk ratio**	**EMA evaluation**	**Number of clinical studies 0**	**Participants 0**	**No placebo control**	**SDG**	**Comparison with approved drugs**	**Drug application for generic medicines**	**No consequences for patients in ongoing clinical trials or compassionate use programmes**	No ongoing clinical trials (at the time the application was withdrawn)	https://www.ema.europa.eu/en/documents/medicine-qa/questions-answers-withdrawal-application-marketing-authorisation-sugammadex-lorien-sugammadex_en.pdf	https://www.ema.europa.eu/en/documents/withdrawal-letter/withdrawal-letter-sugammadex-lorien_en.pdf	**No orphan drugs**	2/8/24
RoActemra	tocilizumab	Tocilizumab	Roche Registration GmbH	Roche Registration GmbH	**Monoclonal antibodies**	**L04AC07**	**12 Respiratory diseases**	ICD-11 Chapter 12	interstitial lung disease associated with systemic sclerosis (in adults)<br><br>Already approved indications:<br>- rheumatoid arthritis (in adults)<br>- systemic juvenile idiopathic arthritis (in children)<br>- COVID-19 (in adults)<br>Note:<br>additional indications in combination with methotrexate	**Ligands (antagonism)(N9.A)**	**/N9.A<br>//**	Ligands<br>(Cytokines, Antagonism)(N9.2.A)	**/N9.2.A<br>//**	**Post-authorisation**	1/16/09	9/13/23	• open questions from the EMA (a17)<br>• concerns regarding responses/information from the manufacturer (a1)<br>• doubts about efficacy<br>reason: lack of data to sufficiently demonstrate efficacy (a27.3)<br>doubts about efficacy<br>reason: other reasons (uncertainties regarding how the target population was defined) (a27)	**/A17<br>/A1<br>/A27.3<br>/A27.<br>//**	10/13/23	**No extension of authorisation, because of no positive benefit-risk ratio**	**EMA evaluation**	**Number of clinical studies 02**	**Participants 0200-0299**	**Placebo controlled clinical studies**		**No comparison with approved drugs**	**No special drug approval procedure**	**No consequences for patients in ongoing clinical trials or compassionate use programmes**	Ongoing clinical trials (at the time the application was withdrawn)	https://www.ema.europa.eu/en/documents/medicine-qa/questions-and-answers-withdrawal-application-change-marketing-authorisation-roactemra-tocilizumab_en.pdf	https://www.ema.europa.eu/en/documents/withdrawal-letter/withdrawal-letter-roactemra-ii-114_en.pdf	**No orphan drugs**	11/7/23
Jivadco	unknown	Trastuzumab/ Duocarmazin	medac Gesellschaft für klinische Spezialpräparate mbH	medac Gesellschaft für klinische Spezialpräparate mbH	**Monoclonal antibodies, Cytostatics (Antibody-Drug Conjugates)**	**L01FD05**	**02 Neoplasms**	ICD-11 Chapte	breast cancer (HER2-positive, if the tumor is locally advanced or metastatic)<br><br>Note:<br>Use if the tumor disease worsened despite at least two treatments targeting HER2, or treatment with Trastuzumab Emtansine.	**Receptors (Antagonism) (N4.A)**<br><br>**other (N6)**	**/N4.A<br>/N6.U<br>//**	Receptors<br>(catalytic receptors, Antagonism) (N4.3.A)<br><br>other (N6)	**/N4.3.A<br>/N6.U<br>//**	**Initial authorisation**		9/12/23	• open questions from the EMA (a17)<br>• concerns regarding responses/information from the manufacturer (a1)<br>doubts about efficacy<br>reason: poor study quality (data analysis) (a27.1.3)<br>• doubts about efficacy<br>reason: inadequate study design (insufficient follow-up of study dropouts) (a27.2)<br>• doubts about efficacy<br>reason: other reasons (doubts about reliability during inspection of the trial site) (a27)	**/A17<br>/A1<br>/A27.1.3<br>/A27.2<br>/A27.<br>//**	10/13/23	**No authorisation, because of no positive benefit-risk ratio**	**EMA evaluation**	**Number of clinical studies 01**	**Participants 0300-0499**	**No placebo control**		**Comparison with approved drugs**	**No special drug approval procedure**	**No consequences for patients in ongoing clinical trials or compassionate use programmes**	No ongoing clinical trials (at the time the application was withdrawn)	https://www.ema.europa.eu/en/documents/medicine-qa/questions-and-answers-withdrawal-application-marketing-authorisation-jivadco-trastuzumab-duocarmazine_en.pdf	https://www.ema.europa.eu/en/documents/withdrawal-letter/withdrawal-letter-jivadco_en.pdf	**No orphan drugs**	11/23/23
Skycovion	GBP510	GBP510	SK Chemicals GmbH	SK Chemicals GmbH	**Other medicines**	**J07BX03**	**X Extension codes (Viral vaccines)**	ICD-11 Chapter X	Prevention of coronavirus disease 2019 (COVID-19)	**other (N6)**	**/N6.U<br>//**	other (N6)	**/N6.U<br>//**	**Initial authorisation**		9/1/23	• open questions from the EMA (a17)<br>• concerns regarding responses/information from the manufacturer (a1)<br>• concerns regarding drug quality (a3)<br>• doubts about efficacy<br>reason: poor study quality (a27.1)<br>• doubts about efficacy<br>reason: lack of data to sufficiently demonstrate efficacy (a27.3)	**/A17<br>/A1<br>/A3.<br>/A27.1<br>/A27.3<br>//**	9/15/23	**No authorisation, because of no positive benefit-risk ratio**	**Marketing strategy**	**Number of clinical studies 01**	**Participants over 2000**	**placebo control unknown**		**Comparison with approved drugs unknown**	**No special drug approval procedure**	**No consequences for patients in ongoing clinical trials or compassionate use programmes**	Ongoing clinical trials (at the time the application was withdrawn)	https://www.ema.europa.eu/en/documents/medicine-qa/questions-and-answers-withdrawal-application-marketing-authorisation-skycovion-covid-19-vaccine-recombinant-adjuvanted_en.pdf	https://www.ema.europa.eu/en/documents/withdrawal-letter/withdrawal-letter-skycovion_en.pdf	**No orphan drugs**	9/15/23
Vivjoa	Oteseconazole	Oteseconazole	Gedeon Richter Plc.	Gedeon Richter Plc.	**Antimycotics**	**J02**	**01 Certain infectious or parasitic diseases**	ICD-11 Chapter 01	Prevention of vaginal candidiasis in women with recurrent infections (not pregnant)	**other (N6)**	**/N6.U<br>//**	other (N6)	**/N6.U<br>//**	**Initial authorisation**		8/24/23	• open questions from the EMA (a17)<br>• concerns regarding responses/information from the manufacturer (a1)<br>• concerns regarding drug quality (manufacturing process) (a3.1)<br>• doubts about efficacy<br>reason: lack of data to sufficiently demonstrate efficacy (a27.3)	**/A17<br>/A1<br>/A3.1<br>/A27.3<br>//**	9/15/23	**No authorisation, because of no positive benefit-risk ratio**	**Marketing strategy**	**Number of clinical studies 03**	**Participants 0500-0999**	**Placebo controlled clinical studies**		**Comparison with approved drugs**	**No special drug approval procedure**	**No consequences for patients in ongoing clinical trials or compassionate use programmes**	No ongoing clinical trials (at the time the application was withdrawn)	https://www.ema.europa.eu/en/documents/medicine-qa/questions-and-answers-withdrawal-application-marketing-authorisation-vivjoa-oteseconazole_en.pdf	https://www.ema.europa.eu/en/documents/withdrawal-letter/withdrawal-letter-vivjoa_en.pdf	**No orphan drugs**	10/23/23
Iclusig	ponatinib	Ponatinib	Incyte Biosciences Distribution B.V.	Incyte Biosciences Distribution B.V.	**Kinase inhibitors**	**L01EA05**	**02 Neoplasms/**<br><br>**X Extension codes (Leukemias)**	ICD-11 Chapter 02<br><br>ICD-11 Chapter X	Acute lymphoblastic leukemia (ALL)<br><br>Note:<br>Treatment of adult patients with newly diagnosed Ph+ ALL, either in combination with a chemotherapy or together with corticosteroids. Use if no chemotherapy and no stem cell transplantation is possible.<br><br>Already approved indications:<br>- chronic myeloid leukemia (CML),<br>- acute lymphoblastic leukemia (ALL) in patients who are Philadelphia chromosome-positive (Ph+)	**Enzyme (Antagonism)(N1.A)**	**/N1.A<br>//**	Enzyme<br>(Transferases, Antagonism)(N1.2.A)	**/N1.2.A<br>//**	**Post-authorisation**	7/1/13	8/11/23	• open questions from the EMA (a17)<br>• concerns regarding responses/information from the manufacturer (a1)<br>• doubts about efficacy<br>reason: poor study quality or inadequate study design (lack of comparison with placebo treatment and/or comparator/reference medicinal products) (a27.1.4)<br>• doubts about efficacy<br>reason: poor study quality (small participant number) (a27.1.5)<br>• doubts about efficacy<br>reason: lack of data to sufficiently demonstrate efficacy (a27.3)<br>• doubts about compliance with good clinical practice (gcp) (a30)	**/A17<br>/A1<br>/A27.1.4<br>/A27.1.5<br>/A27.3<br>/A30<br>//**	9/14/23	**No extension of authorisation, because of no positive benefit-risk ratio**	**EMA evaluation**	**Number of clinical studies 02**	**Participants 0100-0149**	**No placebo control**		**No comparison with approved drugs**	**No special drug approval procedure**	**No consequences for patients in ongoing clinical trials or compassionate use programmes**	No ongoing clinical trials (at the time the application was withdrawn)	https://www.ema.europa.eu/en/documents/medicine-qa/questions-and-answers-withdrawal-application-marketing-authorisation-iclusig_en.pdf	https://www.ema.europa.eu/en/documents/other/withdrawal-letter-iclusig-ii-64_en.pdf	**No orphan drugs**	11/30/23
Lutholaz	unknown	Pegfilgrastim	YES Pharmaceutical Development Services GmbH	YES Pharmaceutical Development Services GmbH	**Immunomodulators**	**L03AA13**	**04 Diseases of the immune system**	ICD-11 Chapter 04	Neutropenia, febrile neutropenia in cancer patients<br><br>Note: not indicated for CML or with myelodysplastic syndromes	**Receptors (Agonism) (N4.B)**	**/N4.B<br>//**	Receptors<br>(catalytic receptors, Agonism) (N4.3.B)	**/N4.3.B<br>//**	**Initial authorisation**		7/19/23	• open questions from the EMA (a17)<br>• concerns regarding responses/information from the manufacturer (a1)<br>• concerns regarding drug quality (a3)<br>• eu gmp (good manufacturing practice) certificate not available (a8)<br>• doubts about efficacy<br>reason: lack of data to sufficiently demonstrate efficacy (a27.3)	**/A17<br>/A1<br>/A3.<br>/A8<br>/A27.3<br>//**	8/18/23	**No authorisation, because of no positive benefit-risk ratio**	**Missing EU GMP certificate**	**Number of clinical studies 0**	**Participants 0**	**No placebo control**	**SDB**	**Comparison with approved drugs**	**Drug application for biosimilars**	**No consequences for patients in ongoing clinical trials or compassionate use programmes**	No ongoing clinical trials (at the time the application was withdrawn)	https://www.ema.europa.eu/en/documents/medicine-qa/questions-and-answers-withdrawal-application-marketing-authorisation-lutholaz-pegfilgrastim_en.pdf	https://www.ema.europa.eu/en/documents/withdrawal-letter/withdrawal-letter-lutholaz-pegfilgrastim_en.pdf	**No orphan drugs**	11/7/23
Gazyvaro,	Obinutuzumab	Obinutuzumab	Roche Registration GmbH	Roche Registration GmbH	**Monoclonal antibodies**	**L01XC15**	**21 Symptoms, signs, or clinical findings not elsewhere classified (Systemic inflammatory response syndrome of non-infectious origin, "cytokine release syndrome" (CRS))/**<br><br>**02 Neoplasms**	ICD-11 Chapter 21<br><br>ICD-11 Chapter 02	Pretreatment before the use of Columvi (Glofitamab, use in B-cell lymphoma) to reduce the risk of a "cytokine release syndrome" (CRS)<br><br>Previous indications:<br>follikular lymphoma, previously untreated chronic lymphocytic leukemia	**other proteins (N5.U)**	**/N5.U<br>//**	other proteins<br>(CD molecules) (N5.1)	**/N5.1.U<br>//**	**Post-authorisation**	7/22/14	7/4/23	• open questions from the EMA (a17)<br>• concerns regarding responses/information from the manufacturer (a1)<br>• doubts about efficacy<br>reason: inadequate study design (related to indication in extension application)(a27.2)<br>• missing justification of dosing regimen (a9)<br>• doubts about same/improved efficacy compared with eu reference medicinal product (no comparison with eu reference medicinal product)(a25)	**/A17<br>/A1<br>/A27.2<br>/A9<br>/A25<br>//**	21/07/2023	**No extension of authorisation, because of no positive benefit-risk ratio**	**EMA evaluation**	**Number of clinical studies 01**	**Participants 0150-0199**	**No placebo control**		**No comparison with approved drugs**	**No special drug approval procedure**	**No consequences for patients in ongoing clinical trials or compassionate use programmes**	Ongoing clinical trials (at the time the application was withdrawn)	Gazyvaro, INN-obinutuzumab (europa.eu)	https://www.ema.europa.eu/en/documents/withdrawal-letter/withdrawal-letter-gazyvaro-ii-0052_en.pdf	**Orphan drugs**	10/4/23
Gefzuris,	Gefapixant	Gefapixant	Merck Sharp & Dohme B.V.	Merck Sharp & Dohme	**Antitussives**	**R05DB29**	**21 Symptoms, signs, or clinical findings not elsewhere classified (Symptoms or signs affecting the respiratory tract, cough)**	**ICD-11 Chapter 21**	cough	**Receptors (Antagonism) (N4.A)**	**/N4.A<br>//**	Receptors<br>(Ligand-gated ion channels, Antagonism) (N4.2.A)	**/N4.2.A<br>//**	**Initial authorisation**		7/14/23	• not available (a14)	**/A14.<br>//**	21/07/2023	**None (documents have not yet been evaluated)**	**Marketing strategy**	**Number of clinical studies unknown**	**Participants unknown**	**placebo control unknown**		**Comparison with approved drugs unknown**	**No special drug approval procedure**	**No consequences for patients in ongoing clinical trials or compassionate use programmes**	No ongoing clinical trials (at the time the application was withdrawn)	Gefzuris | European Medicines Agency (europa.eu)	https://www.ema.europa.eu/en/documents/withdrawal-letter/withdrawal-letter-gefzuris_en.pdf	**No orphan drugs**	11/8/23
Jesduvroq,	Daprodustat	Daprodustat	GlaxoSmithKline Trading Services Limited	GlaxoSmithKline (GSK) Group	**HIF stabilizer**	**B03XA**	**16 Diseases of the urogenital system**<br><br>**03 Diseases of the blood or blood-forming organs**	ICD-11 Chapter 16<br><br>ICD-11 Chapter 03	chronic kidney insufficiency, anemia	**Enzyme (Antagonism)(N1.A)**	**/N1.A<br>//**	Enzyme<br>(Oxidoreductases, Antagonism)(N1.1.A)	**/N1.1.A<br>//**	**Initial authorisation**		7/12/23	• doubts about assurance of patient safety (related to patients without dialysis)<br>reason: insufficient data situation (a21)	**/A21.<br>//**	21/07/2023	**Authorisation with restrictions**	**EMA evaluation**	**Number of clinical studies 05**	**Participants over 2000**	**Placebo controlled clinical studies**		**Comparison with approved drugs**	**No special drug approval procedure**	**No consequences for patients in ongoing clinical trials or compassionate use programmes**	Ongoing clinical trials (at the time the application was withdrawn)	Jesduvroq, INN-Daprodustat (europa.eu)	https://www.ema.europa.eu/en/documents/withdrawal-letter/withdrawal-letter-jesduvroq_en.pdf	**No orphan drugs**	12/13/23
Lagevrio,	Molnupiravir	Molnupiravir	Merck Sharp & Dohme B.V.	Merck Sharp & Dohme	**Antivirals**	**J05A**	**25.1 Codes for special purposes (COVID-19)**	ICD-11 Chapter 25.1	COVID-19 virus infection	**Enzyme (Antagonism)(N1.A)**	**/N1.A<br>//**	Enzyme<br>(Transferases, Antagonism)(N1.2.A)	**/N1.2.A<br>//**	**Initial authorisation**		6/21/23	• doubts about efficacy<br>reason: lack of data to sufficiently demonstrate efficacy (a27.3)<br><br>study data could not show a clear positive effect for patients with covid-19 at increased risk for severe covid-19 course (without oxygen).	**/A27.3<br>//**	27/06/2023	**No authorisation, because of no positive benefit-risk ratio**	**EMA evaluation**	**Number of clinical studies 01**	**Participants 1000-1999**	**Placebo controlled clinical studies**	**SD**	**No comparison with approved drugs**	**No special drug approval procedure**	**No consequences for patients in ongoing clinical trials or compassionate use programmes**	Ongoing clinical trials (at the time the application was withdrawn)	Lagevrio, INN - molnupiravir (europa.eu)	https://www.ema.europa.eu/en/documents/withdrawal-letter/withdrawal-letter-lagevrio_en.pdf	**No orphan drugs**	8/9/23
Zefylti,	Filgrastim	Filgrastim	CuraTeQ Biologics s.r.o	CuraTeQ Biologics s.r.o	**Immunomodulators**	**L03AA02**	**04 Diseases of the immune system**	ICD-11 Chapter 04	neutropenia; chemotherapy-induced febrile neutropenia	**Receptors (Agonism) (N4.B)**	**/N4.B<br>//**	Receptors<br>(catalytic receptors, Agonism) (N4.3.B)	**/N4.3.B<br>//**	**Initial authorisation**		6/8/23	• open questions from the EMA (a17)<br>• concerns regarding responses/information from the manufacturer (a1)<br>• concerns regarding drug quality (a3)<br>• eu gmp (good manufacturing practice) certificate not available (a8)	**/A17<br>/A1<br>/A3.<br>/A8<br>//**	23/06/2023	**No authorisation, because of no positive benefit-risk ratio**	**Missing EU GMP certificate**	**Number of clinical studies 0**	**Participants 0**	**No placebo control**	**SDB**	**Comparison with approved drugs**	**Drug application for biosimilars**	**No consequences for patients in ongoing clinical trials or compassionate use programmes**	Ongoing clinical trials (at the time the application was withdrawn)	Zefylti withdrawal Q&A (europa.eu)	https://www.ema.europa.eu/en/documents/withdrawal-letter/withdrawal-letter-zefylti_en.pdf	**No orphan drugs**	8/30/23
Dyrupeg,	pegfilgrastim	Pegfilgrastim	CuraTeQ Biologics s.r.o	CuraTeQ Biologics s.r.o	**Immunomodulators**	**L03AA13**	**04 Diseases of the immune system**	ICD-11 Chapter 04	neutropenia; chemotherapy-induced febrile neutropenia	**Receptors (Agonism) (N4.B)**	**/N4.B<br>//**	Receptors<br>(catalytic receptors, Agonism) (N4.3.B)	**/N4.3.B<br>//**	**Initial authorisation**		6/8/23	• open questions from the EMA (a17)<br>• concerns regarding responses/information from the manufacturer (a1)<br>• concerns regarding drug quality (a3)<br>• eu gmp (good manufacturing practice) certificate not available (a8)<br>• doubts about same/improved efficacy (bioequivalence) compared with reference medicinal product (neulasta) (a25)	**/A17<br>/A1<br>/A3.<br>/A8<br>/A25<br>//**	23/06/2023	**No authorisation, because of no positive benefit-risk ratio**	**Missing EU GMP certificate**	**Number of clinical studies 0**	**Participants 0**	**No placebo control**	**SDB**	**Comparison with approved drugs**	**Drug application for biosimilars**	**No consequences for patients in ongoing clinical trials or compassionate use programmes**	Ongoing clinical trials (at the time the application was withdrawn)	Dyrupeg_withdrawal Q&A_23 June 2023 (europa.eu)	https://www.ema.europa.eu/en/documents/withdrawal-letter/withdrawal-letter-dyrupeg_en.pdf	**No orphan drugs**	12/14/23

Susvimo,	Ranibizumab	Ranibizumab	Roche Registration GmbH	Roche Registration GmbH	**Monoclonal antibodies**	**S01LA04**	**09 Diseases of the visual system**	**ICD-11 Chapter 09**	wet age-related macular degeneration (AMD)	**Ligands (antagonism)(N9.A)**	**/N9.A //**	Ligands (Vascular endothelial growth factor (VEGF), Antagonism) (N9.1.A)	**/N9.1.A //**	**Initial authorisation**		5/2/23	• doubts about conformity (according to eu standards) (a26)<br>• open questions from the EMA (a17)<br>• concerns regarding requested indication (authorisation recommended only for patients with stable response to vegf-a inhibitor) (a28)	**/A17 /A26 /A28 //**	26/05/2023	**No authorisation, because of no positive benefit-risk ratio**	**EMA evaluation**	**Number of clinical studies 01**	**Participants 0300-0499**	**No placebo control**		**Comparison with approved drugs**	**No special drug approval procedure**	**No consequences for patients in ongoing clinical trials or compassionate use programmes**	Ongoing clinical trials (at the time the application was withdrawn)	Susvimo, INN-Port Delivery System with ranibizumab (europa.eu)	https://www.ema.europa.eu/en/documents/withdrawal-letter/withdrawal-letter-susvimo_en.pdf	**No orphan drugs**	7/18/23
Asimtufii,	aripiprazole	Aripiprazole monohydrate	Otsuka Pharmaceutical Netherlands B.V.	Otsuka Pharmaceutical Netherlands B.V.	**Antipsychotics**	**N05AX12**	**06 Mental, behavioral or neurological developmental disorders**	ICD-11 Chapter 06	schizophrenia	**Receptors (Agonism/Antagonism) (N4.C)**	**/N4.C //**	Receptors (GPCR, Agonism/Antagonism) (N4.1.C)	**/N4.1.C //**	**Initial authorisation**		5/2/23	• open questions from the EMA (a17)<br>• doubts about same/improved efficacy compared with eu reference medicinal product (no comparison with eu reference medicinal product (abilify maintena)) (a25)<br>• concerns regarding responses/information from the manufacturer (a1)	**/A17 /A1 /A25 //**	26/05/2023	**No authorisation, because of no positive benefit-risk ratio**	**Marketing strategy**	**Number of clinical studies 01**	**Participants 0200-0299**	**No placebo control**	**SDH**	**Comparison with approved drugs**	**Drug application for hybrid medicines**	**No consequences for patients in ongoing clinical trials or compassionate use programmes**	Ongoing clinical trials (at the time the application was withdrawn)	Asimtufii, INN-aripiprazole (europa.eu)	https://www.ema.europa.eu/en/documents/withdrawal-letter/withdrawal-letter-asimtufii_en.pdf	**No orphan drugs**	7/7/23
Lumevoq,	Lenadogene Nolparvovec	Lenadogene Nolparvovec	GenSight Biologics S.A.	GenSight Biologics S.A.	**Other medicines**	**S01**	**08 Diseases of the nervous system**	ICD-11 Chapter 08	visual loss due to Leber's hereditary optic neuropathy (LHON)	**Enzyme (Antagonism)(N1.A)**	**/N1.A //**	Enzyme (Translocases, Activation)(N1.7.B)	**/N1.7.B //**	**Initial authorisation**		4/20/23	• doubts about efficacy<br>reason: lack of data to sufficiently demonstrate efficacy (a27.3)<br>• concerns regarding drug quality (a3)<br>• open questions from the EMA (a17) (e.g. regarding submitted laboratory study)<br>• concerns regarding responses/information from the manufacturer (a1)	**/A17 /A1 /A27.3 /A3. //**	26/04/2023	**No authorisation, because of no positive benefit-risk ratio**	**EMA evaluation**	**Number of clinical studies 04**	**Participants 0200-0299**	**Placebo controlled clinical studies**		**No comparison with approved drugs**	**No special drug approval procedure**	**No consequences for patients in ongoing clinical trials or compassionate use programmes**	Ongoing clinical trials (at the time the application was withdrawn)	Lumevoq, INN-lenadogene nolparvovec (europa.eu)	https://www.ema.europa.eu/en/documents/withdrawal-letter/withdrawal-letter-lumevoq_en.pdf	**Orphan drugs**	8/8/23
Tidhesco,	Ivosidenib	Ivosidenib	Les Laboratoires Servier	Les Laboratoires Servier	**Kinase inhibitors**	**L01XX62**	**X Extension codes (Leukemias) /**<br><br>**02 Neoplasms**	ICD-11 Chapter 02<br><br>ICD-11 Chapter X	acute myeloid leukemia	**Enzyme (Antagonism)(N1.A)**	**/N1.A //**	Enzyme (Oxidoreductases, Antagonism)(N1.1.A)	**/N1.1.A //**	**Initial authorisation**		3/27/23	• not available (marketing authorisation was recommended) (a14.2)	**/A14.2 //**	30/05/2023	**Authorisation recommended**	**Marketing strategy**	**Number of clinical studies 01**	**Participants 0100-0149**	**Placebo controlled clinical studies**	**SD**	**Comparison with approved drugs**	**No special drug approval procedure**	**No consequences for patients in ongoing clinical trials or compassionate use programmes**	Ongoing clinical trials unknown (at the time the application was withdrawn)	Tidhesco | European Medicines Agency (europa.eu)	https://www.ema.europa.eu/en/documents/withdrawal-letter/withdrawal-letter-tidhesco_en.pdf	**Orphan drugs**	6/21/23
Buvidal,	Buprenorphin	Buprenorphine	Camurus AB	Camurus AB	**Opioids**	**N07BC01**	**06 Mental, behavioral or neurological developmental disorders**	ICD-11 Chapter 06	Application for extension of marketing authorization for the treatment of chronic pain in opioid addiction<br><br>previous indications:<br>opioid-related disorders/ withdrawal symptoms	**Receptors (Agonism/Antagonism) (N4.C)**	**/N4.C //**	Receptors (GPCR, Agonism/Antagonism) (N4.1.C)	**/N4.1.C //**	**Post-authorisation**	11/20/18	2/13/23	• open questions from the EMA (a17)<br>• concerns regarding the manufacturer's responses/information (a1)<br>• doubts about efficacy<br>reason: inadequate study design (doubts about appropriate patient population and study design) (a27.2)<br>• doubts about efficacy<br>reason: lack of data to adequately demonstrate efficacy (compared with placebo) (a27.3)<br>• doubts about efficacy<br>reason: poor study quality (data analysis) (a27.1.3)<br>• doubts about efficacy<br>reason: poor study quality (conduct) (a27.1.6)	**/A17 /A1 /A27.2 /A27.3 /A27.1.3 /A27.1.6 //**	24/02/2023	**No extension of authorisation, because of no positive benefit-risk ratio**	**EMA evaluation**	**Number of clinical studies 01**	**Participants 0500-0999**	**Placebo controlled clinical studies**		**Comparison with approved drugs**	**Drug application for hybrid medicines**	**No consequences for patients in ongoing clinical trials or compassionate use programmes**	Ongoing clinical trials (at the time the application was withdrawn)	Buvidal_Q-and-A on withdrawal of application (extension of indication) (europa.eu)	https://www.ema.europa.eu/en/documents/withdrawal-letter/withdrawal-letter-buvidal-ii-17_en.pdf	**No orphan drugs**	6/14/23
Feraheme,	Ferumoxytol	Ferumoxytol	Covis Pharma Europe B.V.	Covis	**Other medicines**	**B03AC**	**03 Diseases of the blood or blood-forming organs**	ICD-11 Chapter 03	iron deficiency anemia	**other (N6)**	**/N6.U //**	other (N6)	**/N6.U //**	**Initial authorisation**		3/9/23	• doubts about assurance of patient safety (open questions) (a21)<br>• doubts about efficacy<br>reason: open questions (a27)<br>• open questions from the EMA (a17)<br>• concerns regarding the requested indication (approval for all patients with iron deficiency anemia, without distinction between kidney disease etc.)(a28)	**/A17 /A21. /A28 /A27. //**	31/03/2023	**No authorisation, because of no positive benefit-risk ratio**	**Marketing strategy**	**Number of clinical studies 05**	**Participants 1000-1999**	**Placebo controlled clinical studies**		**Comparison with approved drugs**	**No special drug approval procedure**	**No consequences for patients in ongoing clinical trials or compassionate use programmes**	Ongoing clinical trials (at the time the application was withdrawn)	Feraheme_2023-03_withdrawal Q-and-A (europa.eu)	https://www.ema.europa.eu/en/documents/withdrawal-letter/withdrawal-letter-feraheme_en.pdf	**No orphan drugs**	7/11/23
Raltegravir Viatris,	Raltegravir Potassium	Raltegravir Potassium	Viatris Limited	Viatris Limited	**Antivirals**	**J05AJ01**	**01 Certain infectious or parasitic diseases**	ICD-11 Chapter 01	HIV infections	**Enzyme (Antagonism)(N1.A)**	**/N1.A //**	Enzyme (Transferases, Antagonism)(N1.2.A)	**/N1.2.A //**	**Initial authorisation**		2/22/23	• concerns regarding drug quality (manufacturing process)(a3.1)<br>• concerns regarding the manufacturer's responses/information (a1)<br>• doubts about equivalent/improved efficacy (bioequivalence) compared with the reference medicinal product (isentress)(a25)<br>• open questions from the EMA (a17)	**/A17 /A3.1 /A25 /A1 //**	31/03/2023	**No authorisation, because of no positive benefit-risk ratio**	**EMA evaluation**	**Number of clinical studies 0**	**Participants 0**	**No placebo control**	**SDG**	**Comparison with approved drugs**	**Drug application for generic medicines**	**No consequences for patients in ongoing clinical trials or compassionate use programmes**	Ongoing clinical trials (at the time the application was withdrawn)	Raltegravir Viatris, INN-raltegravir (europa.eu)	https://www.ema.europa.eu/en/documents/withdrawal-letter/withdrawal-letter-raltegravir-viatris_en.pdf	**No orphan drugs**	4/21/23
Garsun,	Artesunate	Artesunate	B And O Pharm	B And O Pharm	**Other medicines**	**P01BE03**	**01 Certain infectious or parasitic diseases**	ICD-11 Chapter 01	malaria	**other (N6)**	**/N6.U //**	other (N6)	**/N6.U //**	**Initial authorisation**		12/8/22	• eu gmp (good manufacturing practice) certificate not available (a8)<br>• open questions from the EMA (a17)<br>• insufficient documentation to justify the application (protection of other medicines for rare diseases from competition by similar medicines) (a13)	**/A17 /A8 /A13 //**	1/03/2023	**No authorisation, because of no positive benefit-risk ratio**	**Marketing strategy**	**Number of clinical studies 02**	**Participants 2000**	**No placebo control**		**Comparison with approved drugs**	**No special drug approval procedure**	**No consequences for patients in ongoing clinical trials or compassionate use programmes**	No ongoing clinical trials (at the time the application was withdrawn)	Garsun, INN-artesunate (europa.eu)	https://www.ema.europa.eu/en/documents/withdrawal-letter/withdrawal-letter-garsun-artesunate_en.pdf	**Orphan drugs**	3/1/23
Ilaris,	canakinumab	Canakinumab	Novartis Europharm Limited	Novartis	**Monoclonal antibodies**	**L04AC08**	**14 Skin diseases**	ICD-11 Chapter 14	treatment of Schnitzler syndrome<br><br>previous indications:<br>cryopyrin-associated periodic syndromes, arthritis, juvenile rheumatoid arthritis, gout	**Ligands (antagonism)(N9.A)**	**/N9.A //**	Ligands (Cytokines, Antagonism)(N9.2.A)	**/N9.2.A //**	**Post-authorisation**	10/23/09	10/26/22	• open questions from the EMA (a17)<br>• concerns regarding the manufacturer's responses/information (a1)<br>• doubts about efficacy<br>reason: poor study quality (conduct, documentation)(a27.1.6)<br><br>note:<br>errors in the randomization procedure	**/A17 /A1 /A27.1.6 //**	11/11/2022	**No authorisation, because of no positive benefit-risk ratio**	**EMA evaluation**	**Number of clinical studies 01**	**Participants 0001-0024**	**Placebo controlled clinical studies**	**SD**	**No comparison with approved drugs**	**No special drug approval procedure**	**No consequences for patients in ongoing clinical trials or compassionate use programmes**	Ongoing clinical trials (at the time the application was withdrawn)	Ilaris, INN-Canakinumab (europa.eu)	https://www.ema.europa.eu/en/documents/withdrawal-letter/withdrawal-letter-ilaris_en.pdf	**No orphan drugs**	2/23/23
Imbarkyd,	Bardoxolonmethyl	Bardoxolone methyl	Reata Ireland Limited	Reata Ireland Limited	**Other medicines**	**Unknown**	**16 Diseases of the urogenital system**	ICD-11 Chapter 16	chronic kidney disease (caused by Alport syndrome)	**other proteins (Agonism) (N5.B)**	**/N5.B //**	other proteins (Agonism) (N5.B)	**/N5.B //**	**Initial authorisation**		11/9/22	• open questions from the EMA (a17)<br>• concerns regarding the manufacturer's responses/information (a1)<br>• doubts about assurance of patient safety (a21)<br>• doubts about efficacy<br>reason: lack of data to adequately demonstrate efficacy (a27.3)	**/A17 /A1 /A21. /A27.3 //**	16/12/2022	**No authorisation, because of no positive benefit-risk ratio**	**EMA evaluation**	**Number of clinical studies 01**	**Participants 0150-0199**	**Placebo controlled clinical studies**		**No comparison with approved drugs**	**No special drug approval procedure**	**No consequences for patients in ongoing clinical trials or compassionate use programmes**	Ongoing clinical trials (at the time the application was withdrawn)	Imbarkyd (bardoxolone) - withdrawal Q&A (europa.eu)	https://www.ema.europa.eu/en/documents/withdrawal-letter/withdrawal-letter-imbarkyd_en.pdf	**Orphan drugs**	2/7/23
Orepaxam,	treprostinil diolamine	Treprostinil Diolamine	Ferrer Internacional S.A.	Ferrer Internacional S.A.	**Prostacyclin analogs**	**B01AC21**	**11 Diseases of the circulatory system**	ICD-11 Chapter 11	pulmonary arterial hypertension (PAH)	**Receptors (Agonism) (N4.B)**	**/N4.B //**	Receptors (GPCR, Agonism) (N4.1.B)	**/N4.1.B //**	**Initial authorisation**		10/11/22	• not available (documents had not yet been evaluated or recommendations not yet published) (a14.1)<br><br>note:<br>documents had not been evaluated at the time of withdrawal of the application.	**/A14.1 //**	11/11/2022	**None (documents have not yet been evaluated)**	**Suspected EMA evaluation**	**Number of clinical studies 04**	**Participants 1000-1999**	**Placebo controlled clinical studies**		**Comparison with approved drugs**	**No special drug approval procedure**	**No consequences for patients in ongoing clinical trials or compassionate use programmes**	No ongoing clinical trials (at the time the application was withdrawn)	OREPAXAM, INN-treprostinil diolamine (europa.eu)	https://www.ema.europa.eu/en/documents/withdrawal-letter/withdrawal-letter-orepaxam_en.pdf	**Orphan drugs**	11/11/22
Febseltiq,	Infigratinib	Infigratinib monophosphate	Helsinn Birex Pharmaceuticals Ltd	Helsinn Birex Pharmaceuticals Ltd	**Kinase inhibitors**	**L01E**	**X Extension codes (Adenomas and adenocarcinomas, cholangiocarcinoma) /**<br>**02 Neoplasms**	ICD-11 Chapter 02<br><br>ICD-11 Chapter X	cholangiocarcinoma	**Receptors (Antagonism) (N4.A)**	**/N4.A //**	Receptors (catalytic receptors, Antagonism) (N4.3.A)	**/N4.3.A //**	**Initial authorisation**		10/11/22	• open questions from the EMA (a17)<br>• doubts about efficacy<br>reason: lack of data to adequately demonstrate efficacy (a27.3)<br>• doubts about assurance of patient safety<br>reason: adverse reactions (serious)(a21.1)<br>• criteria for conditional marketing authorisation not met (a11)<br>• concerns regarding drug quality (manufacturing process)(a3.1)<br>• open questions about bioavailability, distribution and metabolism (one or more topics)(a29)	**/A17 /A3.1 /A11 /A21.1 /A27.3 /A29 //**	11/11/2022	**No authorisation, because of no positive benefit-risk ratio**	**Marketing strategy**	**Number of clinical studies 01**	**Participants 0100-0149**	**No placebo control**		**No comparison with approved drugs**	**No special drug approval procedure**	**Consequences for patients in ongoing clinical trials or compassionate use programmes**	Ongoing clinical trials (at the time the application was withdrawn)	Febseltiq, INN - infigratinib (europa.eu)	https://www.ema.europa.eu/en/documents/withdrawal-letter/withdrawal-letter-febseltiq_en.pdf	**Orphan drugs**	1/30/23
Gavreto,	Pralsetinib	Pralsetinib	Roche Registration GmbH	Roche Registration GmbH	**Kinase inhibitors**	**L01EX23**	**02 Neoplasms**	ICD-11 Chapter 02	thyroid cancer<br><br>already approved indication:<br>non-small cell lung cancer	**Receptors (Antagonism) (N4.A)**	**/N4.A //**	Receptors (catalytic receptors, Antagonism) (N4.3.A)	**/N4.3.A //**	**Post-authorisation**	11/18/21	11/3/22	• open questions from the EMA (a17)<br>• concerns regarding the manufacturer's responses/information (a1)<br>• doubts about efficacy<br>reason: lack of data to adequately demonstrate efficacy (a27.3)	**/A17 /A27.3 /A1 //**	11/11/2022	**No authorisation, because of no positive benefit-risk ratio**	**Marketing strategy**	**Number of clinical studies 01**	**Participants 0150-0199**	**placebo control unknown**		**Comparison with approved drugs unknown**	**No special drug approval procedure**	**No consequences for patients in ongoing clinical trials or compassionate use programmes**	Ongoing clinical trials (at the time the application was withdrawn)	Gavreto, INN - pralsetinib (europa.eu)	https://www.ema.europa.eu/en/documents/withdrawal-letter/withdrawal-letter-gavreto_en.pdf	**No orphan drugs**	2/2/23
Olumiant,	Baricitinib	Baricitinib	Eli Lilly Nederland B.V.	Eli Lilly Nederland B.V.	**Kinase inhibitors**	**L04AA37**	**25.1 Codes for special purposes (COVID-19)**	ICD-11 Chapter 25.1	COVID-19<br>(hospitalized, needing low-flow oxygen therapy or non-invasive ventilation/high-flow oxygen therapy)<br><br>already approved indication:<br>rheumatoid arthritis, atopic dermatitis, alopecia areata	**Enzyme (Antagonism)(N1.A)**	**/N1.A //**	Enzyme (Transferases, Antagonism)(N1.2.A)	**/N1.2.A //**	**Post-authorisation**	2/13/17	12/7/22	• open questions from the EMA (a17)<br>• concerns regarding the manufacturer's responses/information (a1)<br>• doubts about efficacy<br>reason: lack of data to adequately demonstrate efficacy (a27.3)	**/A17 /A1 /A27.3 //**	16/12/2022	**No authorisation, because of no positive benefit-risk ratio**	**EMA evaluation**	**Number of clinical studies 03**	**Participants over 2000**	**Placebo controlled clinical studies**		**Comparison with approved drugs**	**No special drug approval procedure**	**No consequences for patients in ongoing clinical trials or compassionate use programmes**	Ongoing clinical trials (at the time the application was withdrawn)	Olumiant, INN-baricitinib (europa.eu)	https://www.ema.europa.eu/en/documents/withdrawal-letter/withdrawal-letter-olumiant-ii-0028_en.pdf	**No orphan drugs**	2/2/23
Tuznue,	Trastuzumab	Trastuzumab	Prestige Biopharma Belgium	Prestige Biopharma Belgium	**Monoclonal antibodies**	**L01XC03**	**02 Neoplasms**	ICD-11 Chapter 02	breast cancer, gastric cancer	**Receptors (Antagonism) (N4.A)**	**/N4.A //**	Receptors (catalytic receptors, Antagonism) (N4.3.A)	**/N4.3.A //**	**Initial authorisation**		9/14/22	• concerns regarding drug quality (manufacturing process)(a3.1)<br>• concerns regarding the manufacturer's responses/information (a1)<br><br>note:<br>manufacture for clinical studies differed from commercial manufacture)	**/A3.1 /A1 //**	14/10/2022	**No authorisation, because of no positive benefit-risk ratio**	**EMA evaluation**	**Number of clinical studies 01**	**Participants 0500-0999**	**No placebo control**	**SDB**	**Comparison with approved drugs**	**Drug application for biosimilars**	**No consequences for patients in ongoing clinical trials or compassionate use programmes**	No ongoing clinical trials (at the time the application was withdrawn)	Tuznue, INN-trastuzumab (europa.eu)	https://www.ema.europa.eu/en/documents/withdrawal-letter/withdrawal-letter-tuznue_en.pdf	**No orphan drugs**	12/13/22
Imbruvica,	Ibrutinib	Ibrutinib	Janssen-Cilag International NV	Janssen Pharmaceutical Companies of Johnson & Johnson	**Kinase inhibitors**	**L01EL01**	**02 Neoplasms**	ICD-11 Chapter 02	mantle cell lymphoma (MCL) in combination with Bendamustine and Rituximab<br><br>Already approved indications: Mantle cell lymphoma (MCL), chronic lymphocytic leukemia (CLL), lymphoplasmacytic lymphoma	**Enzyme (Antagonism)(N1.A)**	**/N1.A //**	Enzyme (Transferases, Antagonism)(N1.2.A)	**/N1.2.A //**	**Post-authorisation**	10/21/14	12/13/22	• open questions from the EMA (a17)<br>• doubts about assurance of patient safety<br>reason: adverse reactions (serious)(a21.1)<br>• doubts about benefit (a18)<br>• concerns regarding the requested indication (a28)	**/A17 /A28 /A21.1 /A18 //**	27/01/2023	**No authorisation, because of no positive benefit-risk ratio**	**EMA evaluation**	**Number of clinical studies 01**	**Participants 0500-0999**	**Placebo controlled clinical studies**		**Comparison with approved drugs**	**No special drug approval procedure**	**No consequences for patients in ongoing clinical trials or compassionate use programmes**	Ongoing clinical trials (at the time the application was withdrawn)	Imbruvica withdrawal Q&A -EoI II-73 (europa.eu)	https://www.ema.europa.eu/en/documents/withdrawal-letter/withdrawal-letter-imbruvica-ii-73_en.pdf	**No orphan drugs**	1/27/23

Hervelous,	Trastuzumab	Trastuzumab	Prestige Biopharma Belgium	Prestige Biopharma Belgium	**Monoclonal antibodies**	**L01XC03**	**02 Neoplasms**	ICD-11 Chapter 02	breast cancer, gastric cancer	**Receptors (Antagonism) (N4.A)**	**/N4.A //**	Receptors (catalytic receptors, Antagonism) (N4.3.A)	**/N4.3.A //**	**Initial authorisation**		9/14/22	• concerns regarding drug quality (manufacturing process)(a3.1)<br>• concerns regarding the manufacturer's responses/information (a1)<br><br>note:<br>manufacture for clinical studies differed from commercial manufacture)	**/A3.1 /A1 //**	14/10/2022	**No authorisation, because of no positive benefit-risk ratio**	**EMA evaluation**	**Number of clinical studies 01**	**Participants 0500-0999**	**No placebo control**	**SDB**	**Comparison with approved drugs**	**Drug application for biosimilars**	**No consequences for patients in ongoing clinical trials or compassionate use programmes**	No ongoing clinical trials (at the time the application was withdrawn)	Hervelous, INN-trastuzumab (europa.eu)	https://www.ema.europa.eu/en/documents/withdrawal-letter/withdrawal-letter-hervelous_en.pdf	**No orphan drugs**	12/13/22
Sevsury,	Surufatinib	Surufatinib	Hutchmed Europe B.V.	Hutchmed Europe B.V.	**Kinase inhibitors**	**L01EX01**	**02 Neoplasms**	ICD-11 Chapter 02	neuroendocrine tumors	**Receptors (Antagonism) (N4.A)**	**/N4.A //**	Receptors (catalytic receptors, Antagonism) (N4.3.A)	**/N4.3.A //**	**Initial authorisation**		8/1/22	• open questions from the EMA (a17)<br>• doubts about efficacy<br>reason: poor study quality (uncertainties in methodology, good clinical practice etc.)(a27.1)<br>• doubts about efficacy<br>reason: lack of data to adequately demonstrate efficacy(a27.3)<br>• concerns regarding drug quality (a3)	**/A17 /A3. /A27.1 /A27.3 //**	19/08/2022	**No authorisation, because of no positive benefit-risk ratio**	**EMA evaluation**	**Number of clinical studies 02**	**Participants 0300-0499**	**Placebo controlled clinical studies**		**No comparison with approved drugs**	**No special drug approval procedure**	**No consequences for patients in ongoing clinical trials or compassionate use programmes**	Ongoing clinical trials (at the time the application was withdrawn)	Sevsury; INN-surufatinib (europa.eu)	https://www.ema.europa.eu/en/documents/withdrawal-letter/withdrawal-letter-sevsury_en.pdf	**No orphan drugs**	11/16/22
Exkivity,	Mobocertinib	Mobocertinib	Takeda Pharma A/S	Takeda	**Kinase inhibitors**	**L01EB**	**02 Neoplasms**	ICD-11 Chapter 02	non-small cell lung cancer (NSCLC)	**Receptors (Antagonism) (N4.A)**	**/N4.A //**	Receptors (catalytic receptors, Antagonism) (N4.3.A)	**/N4.3.A //**	**Initial authorisation**		7/20/22	• open questions from the EMA (a17)<br>• doubts about efficacy<br>reason: lack of data to adequately demonstrate efficacy(a27.3)<br>• criteria for conditional marketing authorisation not met (a11)<br>• doubts about equivalent/improved efficacy compared with reference medicinal products (a25)	**/A17 /A25 /A11 /A27.3 //**	19/08/2022	**No authorisation, because of no positive benefit-risk ratio**	**EMA evaluation**	**Number of clinical studies 01**	**Participants 0100-0149**	**No placebo control**		**No comparison with approved drugs**	**No special drug approval procedure**	**No consequences for patients in ongoing clinical trials or compassionate use programmes**	Ongoing clinical trials (at the time the application was withdrawn)	Exkivity; INN-mobocertinib (europa.eu)	https://www.ema.europa.eu/en/documents/withdrawal-letter/withdrawal-letter-exkivity_en.pdf	**No orphan drugs**	11/24/22
Parsaclisib Incyte Biosciences Distribution B.V.,	Parsaclisib	Parsaclisib Hydrochloride	Incyte Biosciences Distribution B.V.	Incyte Biosciences Distribution B.V.	**Kinase inhibitors**	**L01EM04**	**X Extension codes (Non-Hodgkin lymphomas, Marginal zone B-cell lymphoma) / 02 Neoplasms**	ICD-11 Chapter 02<br><br>ICD-11 Chapter X	marginal zone lymphoma (MZL) (monotherapy after recurrence or unsuccessful pretreatment)	**Enzyme (Antagonism)(N1.A)**	**/N1.A //**	Enzyme (Transferases, Antagonism)(N1.2.A)	**/N1.2.A //**	**Initial authorisation**		6/27/22	• open questions from the EMA (a17)<br>• concerns regarding drug quality (a3)<br>• doubts about equivalent/improved efficacy (bioequivalence) compared with reference medicinal products (a25)<br>• criteria for conditional marketing authorisation not met (a11)<br>• doubts about efficacy<br>reason: poor study quality or inadequate study design (missing comparison with placebo treatment and/or reference medicinal products) (a27.1.4)<br>• missing justification of the dosage (a9)	**/A17 /A9 /A3. /A25 /A11 /A27.1.4 //**	22/07/2022	**No authorisation, because of no positive benefit-risk ratio**	**EMA evaluation**	**Number of clinical studies 01**	**Participants 0100-0149**	**No placebo control**		**No comparison with approved drugs**	**No special drug approval procedure**	**No consequences for patients in ongoing clinical trials or compassionate use programmes**	Ongoing clinical trials (at the time the application was withdrawn)	Parsaclisib Incyte Biosciences Distribution B.V.; INN-parsaclisib (europa.eu)	https://www.ema.europa.eu/en/documents/withdrawal-letter/withdrawal-letter-parsaclisib-incyte-biosciences-distribution-bv_en.pdf	**Orphan drugs**	11/8/22
HemAryo,	eptacog alfa (activated)	Eptacog alfa (activated)	UGA Biopharma	UGA Biopharma	**Other medicines**	**B02BD08**	**03 Diseases of the blood or blood-forming organs**	ICD-11 Chapter 03	hemophilia A; hemophilia B; factor VII deficiency, Glanzmann-Naegeli thrombastenia	**Enzyme (Antagonism)(N1.A)**	**/N1.A //**	Enzyme (Hydrolases, Agonism)(N1.3.B)	**/N1.3.B //**	**Initial authorisation**		5/6/22	• open questions from the EMA (a17)<br>• concerns regarding drug quality (manufacturing process)(a3.1)<br>• doubts about efficacy<br>reason: poor study quality (conduct)(a27.1.6)<br>• doubts about efficacy<br>reason: poor study quality (a27.1)<br>• concerns regarding the manufacturer's responses/information (a1)	**/A17 /A1 /A3.1 /A27.1.6 /A27.1 //**	20/05/2022	**No authorisation, because of no positive benefit-risk ratio**	**Manufacturing issues**	**Number of clinical studies 0**	**Participants 0**	**No placebo control**	**SDB**	**Comparison with approved drugs**	**Drug application for biosimilars**	**No consequences for patients in ongoing clinical trials or compassionate use programmes**	No ongoing clinical trials (at the time the application was withdrawn)	HemAryo;INN-eptacog alfa (activated) (europa.eu)	https://www.ema.europa.eu/en/documents/withdrawal-letter/withdrawal-letter-hemaryo_en.pdf	**No orphan drugs**	9/27/22
Sitoiganap,	autologous glioma tumor cells, inactivated / autologous glioma tumor cell lysates, inactivated / allogeneic glioma tumor cells, inactivated / allogeneic glioma tumor cell lysates, inactivated	Allogeneic glioma tumor cell lysate (inactivated), Allogeneic glioma tumor cells (inactivated), Autologous glioma tumor cell lysate (inactivated), Autologous glioma tumor cells (inactivated)	Epitopoietic Research Corporation-Belgium (E.R.C.)	Epitopoietic Research Corporation-Belgium (E.R.C.)	**Other medicines**	**L01XX**	**02 Neoplasms /<br><br>X Extension codes (gliosarcoma)**	ICD-11 Chapter 02<br><br>ICD-11 Chapter X	gliosarcoma; glioblastoma (each progressive or recurrent)	**other (N6)**	**/N6.U //**	other (N6)	**/N6.U //**	**Initial authorisation**		5/2/22	• open questions from the EMA (a17)<br>• concerns regarding the manufacturer's responses/information (a1)<br>• concerns regarding drug quality (a3)<br>• doubts about assurance of patient safety (a21)<br>• doubts about efficacy<br>reason: lack of data to adequately demonstrate efficacy(a27.3)	**/A17 /A1 /A21. /A3. /A27.3 //**	20/05/2022	**No authorisation, because of no positive benefit-risk ratio**	**EMA evaluation**	**Number of clinical studies 01**	**Participants 0025-0049**	**Placebo controlled clinical studies**		**Comparison with approved drugs**	**No special drug approval procedure**	**No consequences for patients in ongoing clinical trials or compassionate use programmes**	Ongoing clinical trials (at the time the application was withdrawn)	Sitoiganap, INN-autologous glioma tumor cells (europa.eu)	https://www.ema.europa.eu/en/documents/withdrawal-letter/withdrawal-letter-sitoiganap_en.pdf	**Orphan drugs**	6/17/22
Aduhelm,	Aducanumab	Aducanumab	Biogen Netherlands B.V.	Biogen Netherlands B.V.	**Monoclonal antibodies**	**N07**	**08 Diseases of the nervous system**	ICD-11 Chapter 08	Alzheimer's disease	**Ligands (antagonism)(N9.A)**	**/N9.A //**	Ligands (Antagonism) (N9.A)	**/N9.A //**	**Initial authorisation**		4/20/22	• doubts about assurance of patient safety (adverse reactions) (a21.1)<br>• doubts about efficacy<br>reason: lack of data to adequately demonstrate efficacy(a27.3)<br>• concerns regarding the manufacturer's responses/information (a1)	**/A1 /A21.1 /A27.3 //**	22/04/2022	**No authorisation, because of no positive benefit-risk ratio**	**EMA evaluation**	**Number of clinical studies 02**	**Participants over 2000**	**Placebo controlled clinical studies**		**No comparison with approved drugs**	**No special drug approval procedure**	**No consequences for patients in ongoing clinical trials or compassionate use programmes**	Ongoing clinical trials (at the time the application was withdrawn)	Aduhelm, INN-aducanumab (europa.eu)	https://www.ema.europa.eu/en/documents/withdrawal-letter/withdrawal-letter-aduhelm_en.pdf	**No orphan drugs**	8/1/22
Neffy,	Epinephrin	Epinephrine	Ars Pharmaceuticals Irl Limited	Ars Pharmaceuticals Irl Limited	**Catecholamine**	**C01CA24**	**04 Diseases of the immune system**	ICD-11 Chapter 04	hypersensitivity, allergic reactions/anaphylaxis	**Receptors (Agonism) (N4.B)**	**/N4.B //**	Receptors (GPCR, Agonism) (N4.1.B)	**/N4.1.B //**	**Initial authorisation**		4/4/22	• open questions from the EMA (a17)<br>• concerns regarding the manufacturer's responses/information (a1)<br>• doubts about efficacy<br>reason: poor study quality (missing studies for emergency treatments (allergic reactions, anaphylaxis)). no adequate evidence for comparable absorption in healthy subjects and patients with e.g. severe anaphylaxis (a27.1)<br>• concerns regarding drug quality (preservatives and antioxidants)(a3.2)<br>• concerns regarding the recommended dosage (a6)	**/A17 /A1 /A3.2 /A27.1 /A6 //**	22/04/2022	**No authorisation, because of no positive benefit-risk ratio**	**EMA evaluation**	**Number of clinical studies 04**	**Participants 0500-0999**	**No placebo control**	**SD**	**Comparison with approved drugs**	**No special drug approval procedure**	**No consequences for patients in ongoing clinical trials or compassionate use programmes**	Ongoing clinical trials (at the time the application was withdrawn)	Neffy, INN-Epinephrine (europa.eu)	https://www.ema.europa.eu/en/documents/withdrawal-letter/withdrawal-letter-neffy_en.pdf	**No orphan drugs**	5/20/22
Imcivree,	Setmelanotid	Setmelanotide	Rhythm Pharmaceuticals Netherlands B.V.	Rhythm Pharmaceuticals Netherlands B.V.	**Other medicines**	**A08AA**	**05 Endocrine diseases, nutritional or metabolic diseases**	ICD-11 Chapter 05	obesity, hunger sensation in genetic Alström syndrome<br><br>already approved indications: obesity, Bardet-Biedl syndrome (BBS)	**Receptors (Agonism) (N4.B)**	**/N4.B //**	Receptors (GPCR, Agonism) (N4.1.B)	**/N4.1.B //**	**Post-authorisation**	7/16/21	4/22/22	• open questions from the EMA (a17)<br>• doubts about efficacy<br>reason: poor study quality (small number of participants)(a27.1.5)<br>• doubts about efficacy<br>reason: lack of data to adequately demonstrate efficacy(a27.3)<br>• concerns regarding the manufacturer's responses/information (a1)	**/A17 /A1 /A27.1.5 /A27.3 //**	22/07/2022	**No authorisation, because of no positive benefit-risk ratio**	**EMA evaluation**	**Number of clinical studies 01**	**Participants 0001-0024**	**Placebo controlled clinical studies**		**No comparison with approved drugs**	**No special drug approval procedure**	**No consequences for patients in ongoing clinical trials or compassionate use programmes**	Ongoing clinical trials (at the time the application was withdrawn)	IMCIVREE, INN-setmelanotide (europa.eu)	https://www.ema.europa.eu/en/documents/withdrawal-letter/withdrawal-letter-imcivree_en.pdf	**Orphan drugs**	9/28/22
Miplyffa ,	Arimoclomol	Arimoclomol citrate	Orphazyme A/S	Orphazyme A/S	**Other medicines**	**N07**	**05 Endocrine diseases, nutritional or metabolic diseases**	ICD-11 Chapter 05	Niemann-Pick disease type C	**other proteins (Agonism) (N5.B)**	**/N5.B //**	other proteins (Agonism) (N5.B)	**/N5.B //**	**Initial authorisation**		3/22/22	• doubts about efficacy<br>reason: lack of data to adequately demonstrate efficacy(a27.3)<br>• doubts about efficacy<br>reason: poor study quality (data analysis)(a27.1.3)<br>• concerns regarding the manufacturer's responses/information (a1)<br>• open questions from the EMA (a17)	**/A17 /A27.3 /A1 /A27.1.3 //**	22/04/2022	**No authorisation, because of no positive benefit-risk ratio**	**EMA evaluation**	**Number of clinical studies 01**	**Participants 0050-0074**	**Placebo controlled clinical studies**	**SD**	**Comparison with approved drugs**	**No special drug approval procedure**	**No consequences for patients in ongoing clinical trials or compassionate use programmes**	Ongoing clinical trials (at the time the application was withdrawn)	Miplyffa, INN-arimoclomol (europa.eu)	https://www.ema.europa.eu/en/documents/withdrawal-letter/withdrawal-letter-miplyffa_en.pdf	**Orphan drugs**	2/8/23
Dimherity,	Dimethylfumarat	Dimethyl fumarate	Sandoz GmbH	Sandoz GmbH	**Immunomodulators**	**L04AX07**	**08 Diseases of the nervous system**	ICD-11 Chapter 08	relapsing-remitting multiple sclerosis	**other proteins (Agonism) (N5.B)**	**/N5.B //**	other proteins (Agonism) (N5.B)	**/N5.B //**	**Initial authorisation**		2/22/22	• concerns regarding inconsistent information in application and documentation (a7)<br>• concerns regarding the manufacturer's responses/information (a1)	**/A7 /A1 //**	25/03/2022	**None (documents have not yet been evaluated)**	**Marketing strategy**	**Number of clinical studies 0**	**Participants 0**	**No placebo control**	**SDG**	**Comparison with approved drugs**	**Drug application for generic medicines**	**No consequences for patients in ongoing clinical trials or compassionate use programmes**	No ongoing clinical trials (at the time the application was withdrawn)	Dimherity, INN-dimethyl fumarate (europa.eu)	https://www.ema.europa.eu/en/documents/withdrawal-letter/withdrawal-letter-dimherity_en.pdf	**No orphan drugs**	11/3/22
Aliqopa,	Copanlisib	Copanlisib Dihydrochloride	Bayer AG	Bayer AG	**Kinase inhibitors**	**L01EM02**	**X Extension codes (Non-Hodgkin lymphomas, Marginal zone B-cell lymphoma) / 02 Neoplasms**	ICD-11 Chapter 02<br><br>ICD-11 Chapter X	B-cell lymphomas (marginal zone lymphomas)	**Enzyme (Antagonism)(N1.A)**	**/N1.A //**	Enzyme (Transferases, Antagonism)(N1.2.A)	**/N1.2.A //**	**Initial authorisation**		12/20/21	• concerns regarding the manufacturer's responses/information (a1)<br>• open questions from the EMA (a17)<br>• doubts about efficacy<br>reason: poor study quality (data analysis)(a27.1.3)<br>• doubts about efficacy<br>reason: inadequate study design (monotherapy study)(a27.2)<br>• doubts about efficacy<br>reason: poor study quality (small number of participants)(a27.1.5)<br>• doubts about efficacy<br>reason: lack of data to adequately demonstrate efficacy(a27.3)<br>• doubts about assurance of patient safety<br>reason: lack of data and/or inadequate long-term follow-up (a21.3)<br>• doubts about efficacy<br>reason: poor study quality (conduct) (a27.1.6)	**/A17 /A27.2 /A27.1.3 /A27.1.6 /A1 /A27.1.5 /A27.3 /A21.3 //**	28/01/2022	**No authorisation, because of no positive benefit-risk ratio**	**EMA evaluation**	**Number of clinical studies 02**	**Participants 0100-0149**	**Placebo controlled clinical studies**		**Comparison with approved drugs**	**No special drug approval procedure**	**No consequences for patients in ongoing clinical trials or compassionate use programmes**	Ongoing clinical trials (at the time the application was withdrawn)	Aliqopa; INN-copanlisib (europa.eu)	https://www.ema.europa.eu/en/documents/withdrawal-letter/withdrawal-letter-aliqopa_en.pdf	**Orphan drugs**	2/10/23
Abylqis,	Arachis hypogaea Extrakt	Arachis hypogaea Extract	DBV Technologies	DBV Technologies	**Other medicines**	**V01AA08**	**04 Diseases of the immune system**	**ICD-11 Chapter 04**	peanut allergies	**other (N6)**	**/N6.U //**	other (N6)	**/N6.U //**	**Initial authorisation**		12/17/21	• open questions from the EMA (a17)<br>• doubts about efficacy<br>reason: lack of data to adequately demonstrate efficacy(a27.3)<br>• doubts about assurance of patient safety (adverse reactions)(a21.1)	**/A17 /A21.1 /A27.3 //**	28/01/2022	**No authorisation, because of no positive benefit-risk ratio**	**EMA evaluation**	**Number of clinical studies 01**	**Participants 0300-0499**	**Placebo controlled clinical studies**		**No comparison with approved drugs**	**No special drug approval procedure**	**No consequences for patients in ongoing clinical trials or compassionate use programmes**	Ongoing clinical trials (at the time the application was withdrawn)	Abylqis, INN-arachis hypogaea extract (europa.eu)	https://www.ema.europa.eu/en/documents/withdrawal-letter/withdrawal-letter-abylqis_en.pdf	**No orphan drugs**	11/23/22

Zektayos - Hepjuvo,	Obeticholsäure	Obeticholic acid	ADVANZ PHARMA Limited	ADVANZ PHARMA Limited	**Selective farnesoid X receptor agonists**	**A05AA04**	**13 Diseases of the digestive system**	ICD-11 Chapter 13	non-alcoholic steatohepatitis, fatty liver	**Receptors (Agonism) (N4.B)**	**/N4.B //**	Receptors (nuclear receptors, Agonism) (N4.4.B)	**/N4.4.B //**	**Initial authorisation**		12/9/21	• open questions from the EMA (a17)<br>• concerns regarding the manufacturer's responses/information (a1)<br>• doubts about efficacy<br>reason: lack of data to adequately demonstrate efficacy(a27.3)<br>• doubts about assurance of patient safety<br>reason: adverse reactions (cardiovascular, renal function)(a21.1)	**/A17<br>/A1<br>/A21.1<br>/A27.3<br>//**	17/12/2021	**No authorisation, because of no positive benefit-risk ratio**	**EMA evaluation**	**Number of clinical studies 01**	**Participants 0500-0999**	**Placebo controlled clinical studies**		**No comparison with approved drugs**	**No special drug approval procedure**	**No consequences for patients in ongoing clinical trials or compassionate use programmes**	Ongoing clinical trials (at the time the application was withdrawn)	Zektayos – Hepjuvo (obeticholic acid) (europa.eu)	https://www.ema.europa.eu/en/documents/withdrawal-letter/withdrawal-letter-zektayos-hepjuvo_en.pdf	**No orphan drugs**	4/8/22
Brilique,	Ticagrelor	Ticagrelor	AstraZeneca AB	AstraZeneca	**Platelet aggregation inhibitors**	**B01AC24**	**08 Diseases of the nervous system**	ICD-11 Chapter 08	mild to moderate acute ischemic strokes, high-risk TIA (transient ischemic attack)<br>Use in combination with ASS<br><br>already approved indications: peripheral artery disease; acute coronary syndrome; myocardial infarction (at least one year after event)<br>Use in combination with ASS	**Receptors (Antagonism) (N4.A)**	**/N4.A //**	Receptors (GPCR, Antagonism) (N4.1.A)	**/N4.1.A //**	**Post-authorisation**	12/3/10	12/15/21	• open questions from the EMA (a17)<br>• concerns regarding the manufacturer's responses/information (a1)<br>• doubts about assurance of patient safety<br>reason: adverse reactions (bleeding)(a21.1.1)<br>• doubts about efficacy<br>reason: lack of data to adequately demonstrate efficacy (regarding reduction in number of disabilities)(a27.3)	**/A17<br>/A1<br>/A21.1.1<br>/A27.3<br>//**	28/01/2022	**No authorisation, because of no positive benefit-risk ratio**	**EMA evaluation**	**Number of clinical studies 01**	**Participants over 2000**	**Placebo controlled clinical studies**		**Comparison with approved drugs**	**No special drug approval procedure**	**No consequences for patients in ongoing clinical trials or compassionate use programmes**	Ongoing clinical trials (at the time the application was withdrawn)	Questions and answers on withdrawal of application to change the marketing (europa.eu)	https://www.ema.europa.eu/en/documents/withdrawal-letter/withdrawal-letter-brilique-ii-49_en.pdf	**No orphan drugs**	4/1/22
Tookad,	Padeliporfin	Padeliporfin Di-potassium	STEBA Biotech S.A	STEBA Biotech S.A	**Other medicines**	**L01XD07**	**02 Neoplasms**	ICD-11 Chapter 02	high-grade prostate cancer (ISUP grade 2), change of the evaluation system for the indication of the Gleason scoring system to ISUP grade 1-2, lower restrictive requirements in assessing biopsies for use in more extensive diseases<br><br>already approved indications: prostate neoplasms (low risk, score of 6 or less on the Gleason scoring system)	**other (N6)**	**/N6.U //**	other (N6)	**/N6.U //**	**Post-authorisation**	11/10/17	12/14/21	• open questions from the EMA (a17)<br>• concerns regarding the manufacturer's responses/information (a1)<br>• doubts about efficacy<br>reason: poor study quality (regarding the indication in the extension application)(a27.1.2)	**/A17<br>/A1<br>/A27.1.2<br>//**	28/01/2022	**No authorisation, because of no positive benefit-risk ratio**	**Development strategy**	**Number of clinical studies 0**	**Participants 0**	**No placebo control**	**SD**	**No comparison with approved drugs**	**No special drug approval procedure**	**No consequences for patients in ongoing clinical trials or compassionate use programmes**	Ongoing clinical trials (at the time the application was withdrawn)	Question and answer on withdrawal of application to change the marketing (europa.eu)	https://www.ema.europa.eu/en/documents/withdrawal-letter/withdrawal-letter-tookad-ii-13_en.pdf	**No orphan drugs**	1/28/22
Cervarix,	Impfstoff gegen humane Papillomaviren [Typ 16, 18] (rekombinant, adjuvantiert, adsorbiert)	Human papillomavirus type 16 L1 protein, human papillomavirus type 18 L1 protein	GlaxoSmithKline Biologicals S.A.	GlaxoSmithKline (GSK) Group	**Other medicines**	**J07BM02**	**X Extension codes (Viral vaccines)**	ICD-11 Chapter X	Use from nine years for protection against HPV-associated head and neck cancer<br><br>already approved indications: papillomavirus infections (HPV); cervical dysplasia; anal dysplasia; immunization	**other (N6)**	**/N6.U //**	other (N6)	**/N6.U //**	**Post-authorisation**	9/20/07	10/22/21	• open questions from the EMA (a17)<br>• concerns regarding the manufacturer's responses/information (a1)<br>• doubts about efficacy<br>reason: poor study quality (conduct)(a27.1.6)<br>• doubts about efficacy<br>reason: lack of data to adequately demonstrate efficacy(a27.3)<br>• doubts about efficacy<br>reason: inadequate study design (a27.2)	**/A17<br>/A1<br>/A27.3<br>/A27.1.6<br>/A27.2<br>//**	12/11/2021	**No authorisation, because of no positive benefit-risk ratio**	**EMA evaluation**	**Number of clinical studies 01**	**Participants over 2000**	**No placebo control**		**Comparison with approved drugs**	**No special drug approval procedure**	**No consequences for patients in ongoing clinical trials or compassionate use programmes**	Ongoing clinical trials (at the time the application was withdrawn)	Cervarix, INN-(human papillomavirus vaccine [types 16, 18] (recombinant, adjuvanted, adsorbed)) (europa.eu)	https://www.ema.europa.eu/en/documents/withdrawal-letter/withdrawal-letter-cervarix_en.pdf	**No orphan drugs**	1/10/22
Bamlanivimab und Etesevimab zur Behandlung von COVID-19	Bamlanivimab, Etesevimab	Bamlanivimab, Etesevimab	Eli Lilly Nederland B.V.	Eli Lilly Nederland B.V.	**Monoclonal antibodies**	**Unknown**	**25.1 Codes for special purposes (COVID-19)**	ICD-11 Chapter 25.1	COVID-19 (SARS-CoV-2 virus)	**other proteins (Antagonism/Binding) (N5.A)**	**/N5.A //**	other proteins (accessory proteins, Antagonism/Binding)(N5.A)	**/N5.A //**	**Initial authorisation**		11/2/21	not available<br>(documents had not yet been evaluated or recommendations not yet published)(a14.1)	**/A14.1<br>//**	2/11/2021	**None (documents have not yet been evaluated)**	**Marketing strategy**	**Number of clinical studies unknown**	**Participants unknown**	**placebo control unknown**	**SD**	**Comparison with approved drugs unknown**	**No special drug approval procedure**	**No consequences for patients in ongoing clinical trials or compassionate use programmes**	No ongoing clinical trials (at the time the application was withdrawn)	bamlanivimab / etesevimab_21-11_Q-and-A on RR withdrawal (europa.eu)	https://www.ema.europa.eu/en/documents/withdrawal-letter/withdrawal-letter-bamlanivimab-and-etesevimab_en.pdf	**No orphan drugs**	11/2/21
Zynyz,	Retifanlimab	Retifanlimab	Incyte Biosciences Distribution B.V.	Incyte Biosciences Distribution B.V.	**Monoclonal antibodies**	**L01FX06**	**02 Neoplasms**	ICD-11 Chapter 02	neoplasms of the anus; squamous cell carcinoma	**other proteins (Antagonism/Binding) (N5.A)**	**/N5.A //**	other proteins (Immune checkpoint proteins/CD-molecules, Antagonism/Binding) (N5.4.A)	**/N5.4.A //**	**Initial authorisation**		10/14/21	• open questions from the EMA (a17)<br>• concerns regarding the manufacturer's responses/information (a1)<br>• doubts about efficacy<br>reason: lack of data to adequately demonstrate efficacy (a27.3)<br>• uncertainties regarding the requested indication (a19)<br>• criteria for conditional marketing authorisation not met (a11)	**/A17<br>/A1<br>/A27.3<br>/A19<br>/A11<br>//**	15/10/2021	**No authorisation, because of no positive benefit-risk ratio**	**EMA evaluation**	**Number of clinical studies 01**	**Participants 0075-0099**	**No placebo control**		**No comparison with approved drugs**	**No special drug approval procedure**	**No consequences for patients in ongoing clinical trials or compassionate use programmes**	Ongoing clinical trials (at the time the application was withdrawn)	Zynyz; INN-retifanlimab (europa.eu)	https://www.ema.europa.eu/en/documents/withdrawal-letter/withdrawal-letter-zynyz_en.pdf	**Orphan drugs**	11/29/21
Flynpovi,	Eflornithin, Sulindac	Eflornithine Hydrochloride, Sulindac	Cancer Prevention Pharma (Ireland) Limited	Cancer Prevention Pharma (Ireland) Limited	**Other medicines**	**L01**	**02 Neoplasms**	ICD-11 Chapter 02	familial adenomatous polyposis	**Enzyme (Antagonism/Agonism)(N1.C)**	**/N1.C //**	Enzyme (Lyases, Carboxylases and Decarboxylases, Antagonism) (N1.4.A)<br><br>Enzyme (Transferases, Activation)(N1.2.B)	**/N1.4.A<br>/N1.2.B<br>//**	**Initial authorisation**		10/12/21	• concerns regarding the manufacturer's responses/information (a1)<br>• doubts about efficacy<br>reason: lack of data to adequately demonstrate efficacy(a27.3)<br>• doubts about efficacy<br>reason: poor study quality or inadequate study design (missing comparison with placebo treatment and/or reference medicinal products) (a27.1.4)<br>• doubts about assurance of patient safety<br>reason: lack of data and/or inadequate long-term follow-up (a21.3)<br>• doubts about assurance of patient safety reason: no evidence against gonadotoxicity (a21)	**/A1<br>/A21.<br>/A27.3<br>/A27.1.4<br>/A21.3<br>//**	12/11/2021	**No authorisation, because of no positive benefit-risk ratio**	**EMA evaluation**	**Number of clinical studies 01**	**Participants 0150-0199**	**No placebo control**		**No comparison with approved drugs**	**No special drug approval procedure**	**No consequences for patients in ongoing clinical trials or compassionate use programmes**	No ongoing clinical trials (at the time the application was withdrawn)	Flynpovi - 11-2021 - withdrawal Q&A (europa.eu)	https://www.ema.europa.eu/en/documents/withdrawal-letter/withdrawal-letter-flynpovi_en.pdf	**Orphan drugs**	12/7/21
CureVac's COVID-19 vaccine (CVnCoV),	COVID-19 mRNA vaccine	Single-stranded, 5'-Capped messenger RNA (mRNA), produced with cell-free in vitro transcription from the corresponding DNA templates that encode the viral spike protein (S) of SARS-CoV-2	CureVac AG	CureVac AG	**Other medicines**	**J07BX03**	**X Extension codes (Viral vaccines)**	ICD-11 Chapter X	COVID-19 (SARS-CoV-2 virus)	**other (N6)**	**/N6.U //**	other (N6)	**/N6.U //**	**Initial authorisation**		10/11/21	not available (documents had not yet been evaluated or recommendations not yet published)(a14.1)<br><br>note:<br>questions on drug quality and efficacy were still open	**/A14.1<br>//**	21/10/2021	**None (documents have not yet been evaluated)**	**Marketing strategy**	**Number of clinical studies unknown**	**Participants unknown**	**placebo control unknown**	**SD**	**Comparison with approved drugs unknown**	**No special drug approval procedure**	**No consequences for patients in ongoing clinical trials or compassionate use programmes**	Ongoing clinical trials (at the time the application was withdrawn)	Questions and answers on end of rolling review for CureVac's COVID-19 vaccine (CVnCoV) (europa.eu)	nicht vorhanden	**No orphan drugs**	10/21/21
Teriparatide Cinnagen,	Teriparatid	Teriparatide	CinnaGen Co, Unipessoal LDA	CinnaGen Co, Unipessoal LDA	**Other medicines**	**H05AA02**	**15 Diseases of the musculoskeletal system or connective tissue**	ICD-11 Chapter 15	osteoporosis; postmenopausal osteoporosis;<br>prevention after long-term treatment with glucocorticoids	**other (N6)**<br><br>**Receptors (Agonism) (N4.B)**	**/N4.B<br>/N6.U<br>//**	other (N6)<br><br>Receptors (GPCR, Agonism) (N4.1.B)	**/N6.U<br>/N4.1.B<br>//**	**Initial authorisation**		9/9/21	• open questions from the EMA (a17)<br>• concerns regarding the manufacturer's responses/information (a1)<br>• concerns regarding drug quality (manufacturing process)(a3.1)<br>• concerns regarding recommended dose (pen with too high a dose)(a6)<br>• doubts about equivalent/improved efficacy compared with eu reference medicinal product (a25)<br>• doubts about efficacy<br>reason: poor study quality (data analysis)(a27.1.3)<br>• open questions about bioavailability, distribution and metabolism (one or more topics)(a29)	**/A17<br>/A1<br>/A25<br>/A3.1<br>/A6<br>/A27.1.3<br>/A29<br>//**	17/09/2021	**No authorisation, because of no positive benefit-risk ratio**	**EMA evaluation**	**Number of clinical studies 0**	**Participants 0**	**No placebo control**	**SDB**	**Comparison with approved drugs**	**Drug application for biosimilars**	**No consequences for patients in ongoing clinical trials or compassionate use programmes**	Ongoing clinical trials (at the time the application was withdrawn)	Teriparatide Cinnagen, INN-teriparatide (europa.eu)	https://www.ema.europa.eu/en/documents/withdrawal-letter/withdrawal-letter-teriparatide-cinnagen_en.pdf	**No orphan drugs**	9/17/21
Livmarli,	Maralixibat	Maralixibat Chloride	FGK Representative Service GmbH	FGK Representative Service GmbH	**Other medicines**	**A05AX**	**05 Endocrine diseases, nutritional or metabolic diseases**	ICD-11 Chapter 05	progressive familial intrahepatic cholestasis type 2 (PFIC2)	**Transporter (Antagonism)(N3.A)**	**/N3.A //**	Transporter (SLC-Transporters, Antagonism)(N3.1.A)	**/N3.1.A //**	**Initial authorisation**		8/23/21	• open questions from the EMA (a17)<br>• concerns regarding the manufacturer's responses/information (a1)<br>• doubts about efficacy<br>reason: lack of data to adequately demonstrate efficacy (a27.3)<br>• doubts about efficacy<br>reason: poor study quality (conduct)(a27.1.6)<br>• doubts about efficacy<br>reason: inadequate study design (a27.2)<br>• doubts about eu gmp (good manufacturing practice)(a20)	**/A17<br>/A1<br>/A27.3<br>/A20<br>/A27.1.6<br>/A27.2<br>//**	17/09/2021	**No authorisation, because of no positive benefit-risk ratio**	**Marketing strategy**	**Number of clinical studies 01**	**Participants 0025-0049**	**No placebo control**		**No comparison with approved drugs**	**No special drug approval procedure**	**No consequences for patients in ongoing clinical trials or compassionate use programmes**	Ongoing clinical trials (at the time the application was withdrawn)	"Livmarli, INN-maralixibat" (europa.eu)	https://www.ema.europa.eu/en/documents/withdrawal-letter/withdrawal-letter-livmarli_en.pdf	**Orphan drugs**	12/17/21
Oportuzumab monatox DLRC Pharma Services,	Oportuzumab monatox	Oportuzumab monatox	DLRC Pharma Services Ltd	DLRC Pharma Services Ltd	**Monoclonal antibodies**	**L01FX06**	**02 Neoplasms**	ICD-11 Chapter 02	urological neoplasms; bladder neoplasms (papillary tumors, carcinoma in situ)<br><br>Note:<br>Use after unsuccessful BCG immunotherapy or surgery	**Ligands (N9.U)**	**/N9.U //**	Ligands (N9)	**/N9.U //**	**Initial authorisation**		8/20/21	• open questions from the EMA (a17)<br>• concerns regarding the manufacturer's responses/information (a1)<br>• concerns regarding drug quality (different manufacturing processes)(a3.1)<br>• doubts about eu gmp (good manufacturing practice)(a20)<br>• doubts about efficacy<br>reason: patients in the study were not compatible with the target indication (a27.4)<br>• doubts about efficacy<br>reason: poor study quality (change of study design during study, poor efficacy indicators)(a27.1.1)<br>• doubts about assurance of patient safety<br>reason: conflicting data regarding drug safety (a21)	**/A17<br>/A1<br>/A21.<br>/A3.1<br>/A20<br>/A27.1.1<br>/A27.4<br>//**	17/09/2021	**No authorisation, because of no positive benefit-risk ratio**	**Marketing strategy**	**Number of clinical studies 01**	**Participants 0100-0149**	**No placebo control**		**No comparison with approved drugs**	**No special drug approval procedure**	**No consequences for patients in ongoing clinical trials or compassionate use programmes**	Ongoing clinical trials (at the time the application was withdrawn)	Oportuzumab monatox DLRC Pharma Services; INN-oportuzumab monatox (europa.eu)	https://www.ema.europa.eu/en/documents/withdrawal-letter/withdrawal-letter-oportuzumab-monatox-dlrc-pharma-services_en.pdf	**No orphan drugs**	10/20/21
Sildenafil FGK,	Sildenafil	Sildenafil citrate	FGK Representative Service GmbH	FGK Representative Service GmbH	**PDE-5 inhibitors**	**G04BE03**	**17 Diseases related to sexual health**	ICD-11 Chapter 17	erectile dysfunction	**Enzyme (Antagonism)(N1.A)**	**/N1.A //**	Enzyme (Hydrolases, Antagonism)(N1.3.A)	**/N1.3.A //**	**Initial authorisation**		7/19/21	• open questions from the EMA (a17)<br>• concerns regarding the manufacturer's responses/information (a1)<br>• concerns regarding the form of application application (sublingual) leads to poor absorption compared with the reference medicinal product viagra (a2)	**/A17<br>/A1<br>/A2<br>//**	17/09/2021	**No authorisation, because of no positive benefit-risk ratio**	**EMA evaluation**	**Number of clinical studies 01**	**Participants 0025-0049**	**No placebo control**	**SDH**	**No comparison with approved drugs**	**Drug application for hybrid medicines**	**No consequences for patients in ongoing clinical trials or compassionate use programmes**	No ongoing clinical trials (at the time the application was withdrawn)	Sildenafil citrate FGK, INN-sildenafil (europa.eu)	https://www.ema.europa.eu/en/documents/withdrawal-letter/withdrawal-letter-sildenafil-fgk_en.pdf	**No orphan drugs**	10/5/21

Tecentriq,	Atezolizumab	Atezolizumab	Roche Registration GmbH	Roche Registration GmbH	**Monoclonal antibodies**	**L01XC32**	**02 Neoplasms**	ICD-11 Chapter 02	Expansion of use for the treatment of triple-negative breast cancer. Use should also be preoperatively in combination with chemotherapy based on anthracycline and nab-paclitaxel (previously only if tumor resection was not possible).<br><br>already approved indications:<br>urothelial carcinoma,<br>lung carcinoma (small cell, non-small cell);<br>hepatocellular carcinoma; breast cancer (triple-negative)	**Ligands (antagonism)(N9.A)**	**/N9.A<br>//**	Ligands<br>(Antagonism/Binding)<br>(N9.A)	**/N9.A<br>//**	**Post-authorisation**	9/20/17	6/23/21	• open questions from the EMA (a17)<br>• concerns regarding the manufacturer's responses/information (a1)<br>• doubts about efficacy<br>reason: lack of data to sufficiently demonstrate efficacy (a27.3)	**/A17<br>/A1<br>/A27.3<br>//**	23/07/2021	**No extension of authorisation, because of no positive benefit-risk ratio**	**EMA evaluation**	**Number of clinical studies 01**	**Participants 0300-0499**	**Placebo controlled clinical studies**		**Comparison with approved drugs**	**No special drug approval procedure**	**No consequences for patients in ongoing clinical trials or compassionate use programmes**	Ongoing clinical trials (at the time the application was withdrawn)	Tecentriq, INN-atezolizumab (europa.eu)	https://www.ema.europa.eu/en/documents/withdrawal-letter/withdrawal-letter-tecentriq-ii-52_en.pdf	**No orphan drugs**	7/23/21
Esbriet,	Pirfenidon	Pirfenidone	Roche Registration GmbH	Roche Registration GmbH	**Antifibrotics**	**L04AX05**	**12 Respiratory disea**	**ICD-11 Chapter 12**	expansion of use for the treatment of unclassifiable interstitial lung disease (ILD (UILD))<br><br>already approved indications:<br>idiopathic pulmonary fibrosis (IPF)	**Ligands (antagonism)(N9.A)**	**/N9.A<br>//**	Ligands<br>(TGFβ, PDGF, Antagonism)(N9.A)	**/N9.A<br>//**	**Post-authorisation**	2/27/11	5/19/21	• open questions from the EMA (a17)<br>• concerns regarding the manufacturer's responses/information (a1)<br>• doubts about efficacy<br>reason: poor study quality (regarding the indication in the extension application)(a27.1.2)<br>• uncertainties regarding the requested indication (a19)	**/A17<br>/A1<br>/A19<br>/A27.1.2<br>//**	25/06/2021	**No authorisation, because of no positive benefit-risk ratio**	**EMA evaluation**	**Number of clinical studies 01**	**Participants 0200-0299**	**Placebo controlled clinical studies**		**No comparison with approved drugs**	**No special drug approval procedure**	**No consequences for patients in ongoing clinical trials or compassionate use programmes**	Ongoing clinical trials (at the time the application was withdrawn)	Esbriet II-69_2021-06_Q-and-A on withdrawal of application_DE (europa.eu)	https://www.ema.europa.eu/en/documents/withdrawal-letter/withdrawal-letter-esbriet-ii-69_en.pdf	**Orphan drugs**	6/25/21
Brilique,	Ticagrelor	Ticagrelor	AstraZeneca AB	AstraZeneca	**Platelet aggregation inhibitors**	**B01AC24**	**11 Diseases of the circulatory system**	ICD-11 Chapter 11	expansion of use to prevent atherothrombotic events in adults with type 2 diabetes and coronary heart disease (no prior myocardial infarction but with percutaneous coronary intervention (PCI)) in combination with ASS.<br><br>already approved indications:<br>peripheral artery disease; acute coronary syndrome; myocardial infarction (at least one year after event)<br>Use in combination with ASS	**Receptors (Antagonism)(N4.A)**	**/N4.A<br>//**	Receptors<br>(GPCR, Antagonism) (N4.1.A)	**/N4.1.A<br>//**	**Post-authorisation**	12/3/10	3/5/21	• open questions from the EMA (a17)<br>• concerns regarding the manufacturer's responses/information (a1)<br>• doubts about assurance of patient safety<br>reason: adverse reactions (bleeding)(a21.1.1)<br>• doubts about efficacy<br>reason: poor study quality (regarding the indication in the extension application) because patients with pci were not included in the study (a27.1.2)	**/A17<br>/A1<br>/A21.1.1<br>/A27.1.2<br>//**	26/03/2021	**No authorisation, because of no positive benefit-risk ratio**	**EMA evaluation**	**Number of clinical studies 01**	**Participants over 2000**	**Placebo controlled clinical studies**		**Comparison with approved drugs**	**No special drug approval procedure**	**No consequences for patients in ongoing clinical trials or compassionate use programmes**	Ongoing clinical trials (at the time the application was withdrawn)	Brilique Q-and-A on withdrawal of application (extension of indication) (europa.eu)	https://www.ema.europa.eu/en/documents/withdrawal-letter/withdrawal-letter-brilique_en.pdf	**No orphan drugs**	5/25/21
Dexamethasone Taw,	Dexamethasonphos phat	Dexamethasone (Sodium Phosphate)	Taw Pharma (Ireland) Ltd	Taw Pharma (Ireland) Ltd	**Immunosuppressan ts (no monoclonal antibodies)**	**H02AB02**	**25.1 Codes for special purposes (COVID-19)/**<br><br>**12 Respiratory diseases /**<br><br>**14 Skin diseases /**<br><br>**09 Diseases of the visual system /**<br><br>**04 Diseases of the immune system /**<br><br>**21 Symptoms, signs or clinical findings not elsewhere classified/**<br><br>**08 Diseases of the nervous system**	ICD-11 Chapter 25.1<br><br>ICD-11 Chapter 12<br><br>ICD-11 Chapter 14<br><br>ICD-11 Chapter 09<br><br>ICD-11 Chapter 04<br><br>ICD-11 Chapter 21<br><br>ICD-11 Chapter 08	infection with the COVID-19 virus; brain edema; respiratory distress syndrome in adults; asthma; skin diseases; autoimmune diseases; arthritis; vomiting; inflammation; eye diseases; postoperative nausea and vomiting	**Enzyme (Antagonism)(N1.A)**<br><br>**Receptors (Agonism)(N4.B)**	**/N1.A<br>/N4.B<br>//**	Receptors<br>(nuclear receptors, Agonism)<br>(N4.4.B)<br><br>Enzyme<br>(Hydrolases,<br>Antagonism)(N1.3.A)	**/N4.4.B<br>/N1.3.A<br>//**	**Initial authorisation**		1/20/21	• open questions from the EMA (a17)<br>• concerns regarding responses/information from the manufacturer (a1)<br>• doubts about assurance of patient safety<br>reason: side effects (allergic reactions due to parabens)(a21.1)<br>• concerns regarding medicinal product quality (preservatives)(a3.2)<br>• eu gmp (good manufacturing practice) certificate not available (a8)	**/A17<br>/A1<br>/A21.1<br>/A3.2<br>/A8<br>//**	29/01/2021	**No authorisation, because of no positive benefit-risk ratio**	**EMA evaluation**	**Number of clinical studies 0**	**Participants 0**	**No placebo control**	**SDH**	**No comparison with approved drugs**	**Drug application for hybrid medicines**	**No consequences for patients in ongoing clinical trials or compassionate use programmes**	Ongoing clinical trials unknown (at the time the application was withdrawn)	Dexamethasone Taw (DEXAMETHASONE PHOSPHATE) (europa.eu)	https://www.ema.europa.eu/en/documents/withdrawal-letter/withdrawal-letter-dexamethasone-taw_en.pdf	**No orphan drugs**	3/19/21
Artobend,	autologe humane Chondrozyten in vitro expandiert	Autologous human chondrocytes (expanded in vitro)	TETEC Tissue Engineering Technologies AG	TETEC Tissue Engineering Technologies AG	**Other medicines**	**M09AX02**	**15 Diseases of the musculoskeletal system or connective tissue**	ICD-11 Chapter 15	cartilage defects in the knee	**other (N6)**	**/N6.U<br>//**	other (N6)	**/N6.U<br>//**	**Initial authorisation**		11/18/20	not available (documents have not yet been evaluated or recommendations not yet published) (a14.1)<br><br>note:<br>data have not yet been fully evaluated by the EMA	**/A14.1<br>//**	11/12/2020	**None (documents have not yet been evaluated)**	**Suspected EMA evaluation**	**Number of clinical studies 01**	**Participants 0200-0299**	**No placebo control**		**Comparison with approved drugs**	**No special drug approval procedure**	**No consequences for patients in ongoing clinical trials or compassionate use programmes**	Ongoing clinical trials (at the time the application was withdrawn)	Artobend (autologous human chondrocytes in vitro expanded) (europa.eu)	https://www.ema.europa.eu/en/documents/withdrawal-letter/withdrawal-letter-artobend_en.pdf	**No orphan drugs**	12/11/20
Tecentriq,	Atezolizumab	Atezolizumab	Roche Registration GmbH	Roche Registration GmbH	**Monoclonal antibodies**	**L01XC32**	**02 Neoplasms**	ICD-11 Chapter 02	expansion of use for first-line treatment of advanced or metastatic urothelial carcinoma in combination with a platinum-based chemotherapy<br><br>already approved indications:<br>urothelial carcinoma,<br>lung carcinoma (small cell, non-small cell);<br>hepatocellular carcinoma; breast cancer (triple-negative)	**Ligands (antagonism)(N9.A)**	**/N9.A<br>//**	Ligands<br>(PD-L1, Antagonism/Binding)<br>(N9.A)	**/N9.A<br>//**	**Post-authorisation**	9/20/17	1/8/21	• open questions from the EMA (a17)<br>• concerns regarding responses/information from the manufacturer (a1)<br>• doubts about efficacy<br>reason: lack of data to sufficiently demonstrate efficacy (a27.3)<br>• doubts about efficacy<br>reason: inadequate study design (a27.2)	**/A17<br>/A1<br>/A27.2<br>/A27.3<br>//**	1/02/2021	**No extension of authorisation, because of no positive benefit-risk ratio**	**EMA evaluation**	**Number of clinical studies 01**	**Participants 1000-1999**	**Placebo controlled clinical studies**		**Comparison with approved drugs**	**No special drug approval procedure**	**No consequences for patients in ongoing clinical trials or compassionate use programmes**	Ongoing clinical trials (at the time the application was withdrawn)	Tecentriq: Antrag zurückgezogen | Europäische Arzneimittel-Agentur (europa.eu)<br><br>https://www.ema.europa.eu/en/documents/medicine-qa/questions-and-answers-withdrawal-application-change-marketing-authorisation-tecentriq-atezolizumab_en.pdf-0	https://www.ema.europa.eu/en/documents/withdrawal-letter/withdrawal-letter-tecentriq-ii-42_en.pdf	**No orphan drugs**	2/1/21
Roctavian,	Valoctocogen Roxaparvovec	Valoctocogene Roxaparvovec	BioMarin International Limited	BioMarin International Limited	**Other medicines**	**B02BD15**	**03 Diseases of the blood or blood-forming organs**	ICD-11 Chapter 03	hemophilia A	**other (N6)**	**/N6.U<br>//**	other (N6)	**/N6.U<br>//**	**Initial authorisation**		11/4/20	• open questions from the EMA (a17)<br>• concerns regarding responses/information from the manufacturer (a1)<br>• doubts about efficacy<br>reason: lack of data to sufficiently demonstrate efficacy (a27.3)<br>• doubts about assurance of patient safety<br>reason: lack of data and/or inadequate long-term observation (a21.3)	**/A17<br>/A1<br>/A21.3<br>/A27.3<br>//**	13/11/2020	**No authorisation, because of no positive benefit-risk ratio**	**EMA evaluation**	**Number of clinical studies 01**	**Participants 0025-0049**	**placebo control unknown**		**Comparison with approved drugs unknown**	**No special drug approval procedure**	**No consequences for patients in ongoing clinical trials or compassionate use programmes**	Ongoing clinical trials (at the time the application was withdrawn)	Roctavian - 11-2020 - withdrawal Q&A (europa.eu)	https://www.ema.europa.eu/en/documents/withdrawal-letter/withdrawal-letter-roctavian_en.pdf	**Orphan drugs**	11/13/20
Puldysa,	Idebenon	Idebenone, Titanium dioxide	Santhera Pharmaceuticals (Deutschland) GmbH	Santhera Pharmaceuticals (Deutschland) GmbH	**Other medicines**	**N06BX13**	**08 Diseases of the nervous system**	ICD-11 Chapter 08	muscular dystrophy Duchenne<br><br>Note:<br>Respiratory difficulties in patients with Duchenne muscular dystrophy	**other (N6)**	**/N6.U<br>//**	other (N6)	**/N6.U<br>//**	**Initial authorisation**		10/28/20	• open questions from the EMA (a17)<br>• concerns regarding responses/information from the manufacturer (a1)<br>• doubts about efficacy<br>reason: lack of data to sufficiently demonstrate efficacy (a27.3)	**/A17<br>/A1<br>/A27.3<br>//**	13/11/2020	**No authorisation, because of no positive benefit-risk ratio**	**Other reasons**	**Number of clinical studies 03**	**Participants 0100-0149**	**Placebo controlled clinical studies**	**SDH**	**No comparison with approved drugs**	**Drug application for hybrid medicines**	**Consequences for patients in ongoing clinical trials or compassionate use programmes**	No ongoing clinical trials (at the time the application was withdrawn)	Puldysa, INN-idebenone (europa.eu)	https://www.ema.europa.eu/en/documents/withdrawal-letter/withdrawal-letter-puldysa_en.pdf	**Orphan drugs**	1/18/21
Tibsovo,	Ivosidenib	Ivosidenib	Les Laboratoires Servier	Les Laboratoires Servier	**Kinase inhibitors**	**L01XX62**	**X Extension codes (Leukemias) /**<br><br>**02 Neoplasms**	ICD-11 Chapter 02<br><br>ICD-11 Chapter X	acute myeloid leukemia<br><br>Note:<br>Application for use in patients with relapsed (treatment received) or therapy-resistant AML	**Enzyme (Antagonism)(N1.A)**	**/N1.A<br>//**	Enzyme<br>(Oxidoreductases,<br>Antagonism)(N1.1.A)	**/N1.1.A<br>//**	**Initial authorisation**		10/13/20	• open questions from the EMA (a17)<br>• concerns regarding responses/information from the manufacturer (a1)<br>• doubts about efficacy<br>reason: lack of data to sufficiently demonstrate efficacy (a27.3)	**/A17<br>/A1<br>/A27.3<br>//**	13/11/2020	**No authorisation, because of no positive benefit-risk ratio**	**EMA evaluation**	**Number of clinical studies 01**	**Participants 0150-0199**	**No placebo control**		**No comparison with approved drugs**	**No special drug approval procedure**	**No consequences for patients in ongoing clinical trials or compassionate use programmes**	Ongoing clinical trials (at the time the application was withdrawn)	Tibsovo, INN-ivosidenib (europa.eu)	https://www.ema.europa.eu/en/documents/withdrawal-letter/withdrawal-letter-tibsovo_en.pdf	**Orphan drugs**	4/16/21
Upkanz,	Deferipron	Deferiprone	Apotex B.V.	Apotex B.V.	**Other medicines**	**V03AC02**	**05 Endocrine diseases, nutritional or metabolic diseases**	ICD-11 Chapter 05	pantothenate kinase-associated neurodegeneration (PKAN)	**other (N6)**	**/N6.U<br>//**	other (N6)	**/N6.U<br>//**	**Initial authorisation**		8/10/20	• open questions from the EMA (a17)<br>• concerns regarding responses/information from the manufacturer (a1)<br>• doubts about efficacy<br>reason: lack of data to sufficiently demonstrate efficacy (a27.3)	**/A17<br>/A1<br>/A27.3<br>//**	18/09/2020	**No authorisation, because of no positive benefit-risk ratio**	**EMA evaluation**	**Number of clinical studies 01**	**Participants 0075-0099**	**Placebo controlled clinical studies**		**No comparison with approved drugs**	**No special drug approval procedure**	**No consequences for patients in ongoing clinical trials or compassionate use programmes**	Ongoing clinical trials (at the time the application was withdrawn)	"UPKANZ, INN-deferiprone" (europa.eu)	https://www.ema.europa.eu/en/documents/withdrawal-letter/withdrawal-letter-upkanz_en.pdf	**Orphan drugs**	10/28/20
Rayoqta,	Abiciparpegol	Abicipar pegol	Allergan Pharmaceuticals International Limited	Allergan Pharmaceuticals International Limited	**Kinase inhibitors**	**S01LA**	**09 Diseases of the visual system**	**ICD-11 Chapter 09**	wet age-related macular degeneration (AMD)	**Ligands (antagonism)(N9.A)**	**/N9.A<br>//**	Ligands<br>(Vascular endothelial growth factor (VEGF), Antagonism)<br>(N9.1.A)	**/N9.1.A<br>//**	**Initial authorisation**		7/17/20	• open questions from the EMA (a17)<br>• concerns regarding responses/information from the manufacturer (a1)<br>• doubts about assurance of patient safety<br>reason: side effects such as ocular inflammations (a25)<br>• doubts about equivalent/improved efficacy compared to the eu reference medicinal product<br>(no meaningful study data)(a25)	**/A17<br>/A1<br>/A25<br>/A21.1<br>//**	24/07/2020	**No authorisation, because of no positive benefit-risk ratio**	**EMA evaluation**	**Number of clinical studies 02**	**Participants 1000-1999**	**No placebo control**		**Comparison with approved drugs**	**No special drug approval procedure**	**No consequences for patients in ongoing clinical trials or compassionate use programmes**	No ongoing clinical trials (at the time the application was withdrawn)	Abicipar Pegol Allergan, INN-abicipar pegol (europa.eu)	https://www.ema.europa.eu/en/documents/withdrawal-letter/withdrawal-letter-rayoqta_en.pdf	**No orphan drugs**	10/21/20
Abilify MyCite,	aripiprazole	Aripiprazole	Otsuka Pharmaceutical Netherlands B.V.	Otsuka Pharmaceutical Netherlands B.V.	**Antipsychotics**	**N05AX12**	**06 Mental, behavioral or neurological developmental disorders**	ICD-11 Chapter 06	schizophrenia, bipolar I disorder, prevention of manic episodes	**Receptors (Agonism/Antagonism) (N4.C)**	**/N4.C<br>//**	Receptors<br>(GPCR, Agonism/Antagonism)<br>(N4.1.C)	**/N4.1.C<br>//**	**Initial authorisation**		7/17/20	• open questions from the EMA (a17)<br>• concerns regarding responses/information from the manufacturer (a1)<br>• doubts about assurance of patient safety<br>reason: lack of data on correct detection of doses taken of the medicinal product; patch causes skin reactions (a21)	**/A17<br>/A1<br>/A21.<br>//**	24/07/2020	**No authorisation, because of no positive benefit-risk ratio**	**EMA evaluation**	**Number of clinical studies 02**	**Participants 0100-0149**	**No placebo control**	**SDH**	**No comparison with approved drugs**	**Drug application for hybrid medicines**	**No consequences for patients in ongoing clinical trials or compassionate use programmes**	Ongoing clinical trials (at the time the application was withdrawn)	Abilify MyCite, INN-aripiprazole (europa.eu)	https://www.ema.europa.eu/en/documents/withdrawal-letter/withdrawal-letter-abilify-mycite_en.pdf	**No orphan drugs**	10/22/20

Sondelbay,	Teriparatid	Teriparatide	Accord Healthcare S.L.U.	Accord Healthcare	**Other medicines**	**H05AA02**	**15 Diseases of the musculoskeletal system or connective tissue**	ICD-11 Chapter 15	osteoporosis; postmenopausal osteoporosis;<br>prevention after long-term treatment with glucocorticoids	**other (N6)**<br><br>**Receptors (Agonism) (N4.B)**	**/N4.B<br>/N6.U<br>//**	other (N6)<br><br>Receptors (GPCR, Agonism) (N4.1.B)	**/N6.U<br>/N4.1.B<br>//**	**Initial authorisation**		6/19/20	• open questions from the EMA (a17)<br>• concerns regarding responses/information from the manufacturer (a1)<br>• concerns regarding medicinal product quality (manufacturing process of the finished pen)(a3.1)<br>• doubts about equivalent/improved efficacy compared to the eu reference medicinal product (poor study quality in the distribution study)(a25)<br>• doubts about efficacy<br>reason: poor study quality (conduct)(a27.1.6)<br>• outstanding questions on bioavailability, distribution and metabolism (one or more of these topics)(a29)	**/A17<br>/A1<br>/A25<br>/A3.1<br>/A27.1.6<br>/A29<br>//**	26/06/2020	**No authorisation, because of no positive benefit-risk ratio**	**Other reasons**	**Number of clinical studies 0**	**Participants 0**	**No placebo control**	**SDB**	**Comparison with approved drugs**	**Drug application for biosimilars**	**No consequences for patients in ongoing clinical trials or compassionate use programmes**	Ongoing clinical trials (at the time the application was withdrawn)	Sondelbay-INN-teriparatide - withdrawal Q&A (europa.eu)	https://www.ema.europa.eu/en/documents/withdrawal-letter/withdrawal-letter-sondelbay_en.pdf	**No orphan drugs**	9/28/20
Xiidra,	Lifitegrast	Lifitegrast	Novartis Europharm Limited	Novartis	**Immunomodulators**	**S01XA25**	**09 Diseases of the visual system**	ICD-11 Chapter 09	dry eye syndrome<br><br>(when tear substitutes are insufficient)	**Receptors (Antagonism) (N4.A)**	**/N4.A<br>//**	Receptors (catalytic receptors, Antagonism) (N4.3.A)	**/N4.3.A<br>//**	**Initial authorisation**		6/18/20	• open questions from the EMA (a17)<br>• concerns regarding responses/information from the manufacturer (a1)<br>• doubts about assurance of patient safety<br>reason: lack of data and/or inadequate long-term observation (a21.3)<br>• doubts about equivalent/improved efficacy compared to the eu reference medicinal product (missing comparison with reference medicinal product) (A25)<br>• doubts about efficacy<br>reason: lack of data to sufficiently demonstrate efficacy (a27.3)	**/A17<br>/A1<br>/A25<br>/A21.3<br>/A27.3<br>//**	26/06/2020	**No authorisation, because of no positive benefit-risk ratio**	**EMA evaluation**	**Number of clinical studies 02**	**Participants 1000-1999**	**Placebo controlled clinical studies**		**No comparison with approved drugs**	**No special drug approval procedure**	**No consequences for patients in ongoing clinical trials or compassionate use programmes**	No ongoing clinical trials (at the time the application was withdrawn)	Xiidra, INN-lifitegrast (europa.eu)	https://www.ema.europa.eu/en/documents/withdrawal-letter/withdrawal-letter-xiidra_en.pdf	**No orphan drugs**	8/19/20
Zemdri,	Plazomicin	Plazomicin (Sulfate)	Cipla Europe NV	Cipla Europe NV	**Antibacterial drugs**	**J01GB14**	**16 Diseases of the urogenital system**	**ICD-11 Chapter 16**	complicated urinary tract infections, pyelonephritis<br><br>(use when standard therapy is not possible)	**other proteins (Antagonism/Binding) (N5.A)**	**/N5.A<br>//**	other proteins (bacterial proteins / bacterial protein synthesis, Antagonism)(N5.9.A)	**/N5.9.A<br>//**	**Initial authorisation**		6/16/20	• open questions from the EMA (a17)<br>• concerns regarding responses/information from the manufacturer (a1)<br>• concerns regarding medicinal product quality (manufacturing process, method of sterilisation of the medicinal product) (a3.1)	**/A17<br>/A1<br>/A3.1<br>//**	26/06/2020	**No authorisation, because of no positive benefit-risk ratio**	**Marketing strategy**	**Number of clinical studies 01**	**Participants 0999**	**No placebo control**	**SD**	**Comparison with approved drugs**	**No special drug approval procedure**	**No consequences for patients in ongoing clinical trials or compassionate use programmes**	Ongoing clinical trials (at the time the application was withdrawn)	Zemdri Q-and-A on withdrawal of application (europa.eu)	https://www.ema.europa.eu/en/documents/withdrawal-letter/withdrawal-letter-zemdri_en.pdf	**No orphan drugs**	6/26/20
Erlotinib Accord,	Erlotinib	Erlotinib	Accord Healthcare S.L.U.	Accord Healthcare	**Kinase inhibitors**	**L01XE03**	**02 Neoplasms**	ICD-11 Chapter 02	non-small cell lung carcinoma (NSCLC) (advanced or metastatic), advanced metastatic pancreatic carcinoma (in combination with gemcitabine)	**Receptors (Antagonism) (N4.A)**	**/N4.A<br>//**	Receptors (catalytic receptors, Antagonism) (N4.3.A)	**/N4.3.A<br>//**	**Initial authorisation**		5/28/20	• open questions from the EMA (a17)<br>• concerns regarding responses/information from the manufacturer (a1)<br>• doubts about efficacy<br>reason: poor study quality (data analysis) (a27.1.3)<br>• doubts about efficacy<br>reason: inadequate study design (a27.2)<br>• doubts about efficacy<br>reason: poor study quality (conduct of bioequivalence study) (a27.1.6)<br>• doubts about compliance with good clinical practice (gcp) (a30)<br>• outstanding questions on bioavailability, bioequivalence, distribution and metabolism (one or more topics) (a29)<br>• doubts about equivalent/improved efficacy compared to the eu reference medicinal product (a25)	**/A17<br>/A1<br>/A25<br>/A29<br>/A27.1.6<br>/A27.1.3<br>/A30<br>/A27.2<br>//**	29/05/2020	**No authorisation, because of no positive benefit-risk ratio**	**EMA evaluation**	**Number of clinical studies 0**	**Participants unknown**	**No placebo control**	**SDG**	**Comparison with approved drugs**	**Drug application for generic medicines**	**No consequences for patients in ongoing clinical trials or compassionate use programmes**	Ongoing clinical trials (at the time the application was withdrawn)	Erlotinib Accord - INN-erlotinib (europa.eu)	https://www.ema.europa.eu/en/documents/withdrawal-letter/withdrawal-letter-erlotinib-accord_en.pdf	**No orphan drugs**	5/29/20
Fingolimod Mylan,	Fingolimod	Fingolimod Hydrochloride	Mylan Ireland Limited	Mylan	**Immunosuppressants (no monoclonal antibodies)**	**L04AA27**	**08 Diseases of the nervous system**	ICD-11 Chapter 08	multiple sclerosis, relapsing-remitting	**Receptors (Agonism) (N4.B)**	**/N4.B<br>//**	Receptors (GPCR, Agonism) (N4.1.B)	**/N4.1.B<br>//**	**Initial authorisation**		5/8/20	• open questions from the EMA (a17)<br>• concerns regarding responses/information from the manufacturer (a1)<br>• doubts about equivalent/improved efficacy compared to the eu reference medicinal product (a25)<br>• concerns regarding medicinal product quality (manufacturing process, nitrosamines) (a3.1)<br>• doubts about efficacy<br>reason: poor study quality (conduct of bioequivalence study) (a27.1.6)<br>• outstanding questions on bioavailability, bioequivalence, distribution and metabolism (one or more topics) (a29)	**/A17<br>/A1<br>/A3.1<br>/A25<br>/A29<br>/A27.1.6<br>//**	29/05/2020	**No authorisation, because of no positive benefit-risk ratio**	**EMA evaluation**	**Number of clinical studies 0**	**Participants 0**	**No placebo control**	**SDG**	**Comparison with approved drugs**	**Drug application for generic medicines**	**No consequences for patients in ongoing clinical trials or compassionate use programmes**	Ongoing clinical trials (at the time the application was withdrawn)	Fingolimod Mylan, INN-fingolimod (europa.eu)	https://www.ema.europa.eu/en/documents/withdrawal-letter/withdrawal-letter-fingolimod-mylan_en.pdf-0	**No orphan drugs**	6/17/20
Rituximab Mabion,	Rituximab	Rituximab	Mabion Spolka Akcyjna	Mabion Spolka Akcyjna	**Monoclonal antibodies**	**L01XC02**	**02 Neoplasms/**<br><br>**X Extension codes (Leukemias) /**<br><br>**X Extension codes (Hodgkin and Non-Hodgkin lymphomas)**<br><br>**15 Diseases of the musculoskeletal system or connective tissue/**<br><br>**04 Diseases of the immune system**	ICD-11 Chapter 02<br><br>ICD-11 Chapter 15<br><br>ICD-11 Chapter 04<br><br>ICD-11 Chapter X	non-Hodgkin lymphomas, chronic lymphocytic leukaemias (CLL) (B-cells), rheumatoid arthritis, microscopic polyangiitis	**other proteins (N5.U)**	**/N5.U<br>//**	other proteins (CD molecules) (N5.1)	**/N5.1.U<br>//**	**Initial authorisation**		3/16/20	• open questions from the EMA (a17)<br>• concerns regarding responses/information from the manufacturer (a1)<br>• concerns regarding medicinal product quality (manufacturing process) (a3.1)<br>• doubts about equivalent/improved efficacy compared to the eu reference medicinal product (a25)	**/A17<br>/A1<br>/A3.1<br>/A25<br>//**	30/03/2020	**No authorisation, because of no positive benefit-risk ratio**	**EMA evaluation**	**Number of clinical studies 01**	**Participants 0500-0999**	**No placebo control**	**SDB**	**Comparison with approved drugs**	**Drug application for biosimilars**	**No consequences for patients in ongoing clinical trials or compassionate use programmes**	Ongoing clinical trials (at the time the application was withdrawn)	Rituximab Mabion; INN-rituximab (europa.eu)	https://www.ema.europa.eu/en/documents/withdrawal-letter/rituximab-mabion-withdrawal-letter_en.pdf	**No orphan drugs**	6/24/20
Doxorubicin Hydrochloride Tillomed,	Doxorubicin	Doxorubicin hydrochloride	Laboratorios Tillomed Spain S.L.U	Laboratorios Tillomed Spain S.L.U	**Cytostatics**	**L01DB01**	**02 Neoplasms**	ICD-11 Chapter 02	neoplasms of the breast, neoplasms of the ovaries, multiple myeloma, Kaposi's sarcoma (AIDS-associated)	**Enzyme (Antagonism)(N1.A)**	**/N1.A<br>//**	Enzyme (Isomerases, Antagonism) (N1.5.A)	**/N1.5.A<br>//**	**Initial authorisation**		3/2/20	• open questions from the EMA (a17)<br>• concerns regarding responses/information from the manufacturer (a1)<br>• doubts about efficacy<br>reason: poor study quality (data analysis)(a27.1.3)<br>• deficiencies in compliance with good clinical practice (gcp)(a30)	**/A17<br>/A1<br>/A27.1.3<br>/A30<br>//**	27/03/2020	**No authorisation, because of no positive benefit-risk ratio**	**EMA evaluation**	**Number of clinical studies 0**	**Participants 0**	**No placebo control**	**SDGH**	**Comparison with approved drugs**	**Drug application for hybrid medicines and generic medicines**	**No consequences for patients in ongoing clinical trials or compassionate use programmes**	Ongoing clinical trials (at the time the application was withdrawn)	Doxorubicin Tillomed, INN-doxorubicin hydrochloride pegylated liposomal concentrate for dispersion for infusion, 2 mg/ml (europa.eu)	https://www.ema.europa.eu/en/documents/withdrawal-letter/withdrawal-letter-doxorubicin-hydrochloride-tillomed_en.pdf	**No orphan drugs**	7/27/20
Idhifa,	enasidenib	Enasidenib	Celgene Europe BV	Celgene	**Kinase inhibitors**	**L01XX**	**X Extension codes (Leukemias) /**<br><br>**02 Neoplasms**	ICD-11 Chapter 02<br><br>ICD-11 Chapter X	refractory or relapsed acute myeloid leukemia (AML) when no intensive cancer treatment is possible	**Enzyme (Antagonism)(N1.A)**	**/N1.A<br>//**	Enzyme (Oxidoreductases, Antagonism)(N1.1.A)	**/N1.1.A<br>//**	**Initial authorisation**		12/6/19	• open questions from the EMA (a17)<br>• concerns regarding responses/information from the manufacturer (a1)<br>• doubts about efficacy<br>reason: lack of data to sufficiently demonstrate efficacy (a27.3)	**/A17<br>/A1<br>/A27.3<br>//**	31/01/2020	**No authorisation, because of no positive benefit-risk ratio**	**EMA evaluation**	**Number of clinical studies 01**	**Participants 0200-0299**	**No placebo control**		**No comparison with approved drugs**	**No special drug approval procedure**	**No consequences for patients in ongoing clinical trials or compassionate use programmes**	Ongoing clinical trials (at the time the application was withdrawn)	Idhifa, INN-enasidenib (europa.eu)	https://www.ema.europa.eu/en/documents/withdrawal-letter/withdrawal-letter-idhifa_en.pdf	**Orphan drugs**	2/7/20
Yervoy,	ipilimumab	Ipilimumab	Bristol-Myers Squibb Pharma EEIG	Bristol-Myers Squibb Pharma EEIG	**Monoclonal antibodies**	**L01FX04**	**02 Neoplasms**	ICD-11 Chapter 02	untreated metastatic non-small cell lung carcinoma (NSCLC) (combination Opdivo and Yervoy in patients without ALK and EGFR mutation)<br><br>Already approved indications: melanoma, renal cell carcinoma, non-small cell lung carcinoma, malignant pleural mesothelioma, colorectal carcinoma, squamous cell carcinoma of the esophagus	**other proteins (Antagonism/Binding) (N5.A)**	**/N5.A<br>//**	other proteins (Immune checkpoint proteins/CD-molecules, Antagonism/Binding) (N5.4.A)	**/N5.4.A<br>//**	**Post-authorisation**	7/13/11	1/30/20	• open questions from the EMA (a17)<br>• concerns regarding responses/information from the manufacturer (a1)<br>• doubts about efficacy<br>reason: lack of data to sufficiently demonstrate efficacy (a27.3)<br>• doubts about efficacy<br>reason: poor study quality (change of study design during the study, inadequate indicators of efficacy) (a27.1.1)<br>• doubts about efficacy<br>reason: poor study quality (data analysis) (a27.1.3)	**/A17<br>/A1<br>/A27.3<br>/A27.1.1<br>/A27.1.3<br>//**	28/02/2020	**No extension of authorisation, because of no positive benefit-risk ratio**	**EMA evaluation**	**Number of clinical studies 01**	**Participants 1000-1999**	**No placebo control**		**Comparison with approved drugs**	**No special drug approval procedure**	**No consequences for patients in ongoing clinical trials or compassionate use programmes**	Ongoing clinical trials (at the time the application was withdrawn)	Opdivo; INN-nivolumab / Yervoy; INN-ipiliumumab (europa.eu)	https://www.ema.europa.eu/en/documents/withdrawal-letter/withdrawal-letter-opdivo-and-yervoy-ws1372_en.pdf	**No orphan drugs**	4/23/20
Opdivo,	nivolumab	Nivolumab	Bristol-Myers Squibb Pharma EEIG	Bristol-Myers Squibb Pharma EEIG	**Monoclonal antibodies**	**L01FF01**	**02 Neoplasms**	ICD-11 Chapter 02	untreated metastatic non-small cell lung carcinoma (NSCLC) (combination Opdivo and Yervoy in patients without ALK and EGFR mutation)<br><br>Already approved indications: melanoma, Hodgkin lymphoma, renal cell carcinoma, non-small cell lung carcinoma, squamous cell carcinoma of the esophagus, gastroesophageal junction cancer, squamous cell carcinoma of the head and neck (SCCHN), urothelial carcinoma, malignant pleural mesothelioma, colorectal carcinoma, gastric carcinoma	**other proteins (Antagonism/Binding) (N5.A)**	**/N5.A<br>//**	other proteins (Immune checkpoint proteins/CD-molecules, Antagonism/Binding) (N5.4.A)	**/N5.4.A<br>//**	**Post-authorisation**	6/19/15	1/30/20	• open questions from the EMA (a17)<br>• concerns regarding responses/information from the manufacturer (a1)<br>• doubts about efficacy<br>reason: lack of data to sufficiently demonstrate efficacy (a27.3)<br>• doubts about efficacy<br>reason: poor study quality (change of study design during the study, inadequate indicators of efficacy) (a27.1.1)<br>• doubts about efficacy<br>reason: poor study quality (data analysis) (a27.1.3)	**/A17<br>/A1<br>/A27.3<br>/A27.1.1<br>/A27.1.3<br>//**	28/02/2020	**No extension of authorisation, because of no positive benefit-risk ratio**	**EMA evaluation**	**Number of clinical studies 01**	**Participants 1000-1999**	**No placebo control**		**Comparison with approved drugs**	**No special drug approval procedure**	**No consequences for patients in ongoing clinical trials or compassionate use programmes**	Ongoing clinical trials (at the time the application was withdrawn)	Opdivo; INN-nivolumab / Yervoy; INN-ipiliumumab (europa.eu)	https://www.ema.europa.eu/en/documents/withdrawal-letter/withdrawal-letter-opdivo-and-yervoy-ws1372_en.pdf	**No orphan drugs**	4/23/20

Keytruda,	pembrolizumab	Pembrolizumab	Merck Sharp & Dohme B.V.	Merck Sharp & Dohme	**Monoclonal antibodies**	**L01FF02**	**02 Neoplasms**	ICD-11 Chapter 02	repeatedly occurring, locally advanced or metastatic esophageal cancer after prior treatment with other cancer drugs<br><br>Already approved indications: melanoma, non-small cell lung carcinoma, classical Hodgkin lymphoma, urothelial carcinoma, head and neck squamous cell carcinoma, renal cell carcinoma, specific cancers when microsatellite instability is high (MSI-H) or mismatch repair deficiency (colon cancer, endometrial cancer, gastric cancer, small bowel cancer, bile duct cancer) triple-negative breast cancer, gastric carcinoma, esophageal carcinoma, endometrial carcinoma, cervical carcinoma	**other proteins (Antagonism/Binding) (N5.A)**	**/N5.A //**	other proteins (immune checkpoint proteins/CD-molecules, Antagonism/Binding) (N5.4.A)	**/N5.4.A //**	**Post-authorisation**	7/17/15	12/10/19	• open questions from the EMA (a17)<br>• concerns regarding responses/information from the manufacturer (a1)<br>• doubts about efficacy<br>reason: lack of data to sufficiently demonstrate efficacy (a27.3)	**/A17 /A1 /A27.3 //**	31/01/2020	**No extension of authorisation, because of no positive benefit-risk ratio**	**EMA evaluation**	**Number of clinical studies 01**	**Participants 0500-0999**	**No placebo control**		**Comparison with approved drugs**	**No special drug approval procedure**	**No consequences for patients in ongoing clinical trials or compassionate use programmes**	Ongoing clinical trials (at the time the application was withdrawn)	Keytruda II-72 - 2020-01 - withdrawal Q&A (extension of indication) (europa.eu)	https://www.ema.europa.eu/en/documents/withdrawal-letter/withdrawal-letter-keytruda-ii-72_en.pdf	**No orphan drugs**	3/19/20
Luxceptar,	(luxceptar)	particularly treated T cells of the transplant donor<br><br>Note: Medicinal products for novel therapies	Kiadis Pharma Netherlands B.V.	Kiadis Pharma Netherlands B.V.	**Other medicines**	**Unknown**	**04 Diseases of the immune system/**<br><br>**24 Factors influencing health status or contact with health services**	ICD-11 Chapter 04<br><br>ICD-11 Chapter 24	graft vs host disease (GVHD), hematopoietic stem cell transplantation (T-cell-depleted blood stem cell transplantation)	**other (N6)**	**/N6.U //**	other (N6)	**/N6.U //**	**Initial authorisation**		11/6/19	• open questions from the EMA (a17)<br>• concerns regarding responses/information from the manufacturer (a1)<br>• doubts about efficacy<br>reason: poor study quality (small participant number) (a27.1.5)<br>• doubts about efficacy<br>reason: inadequate study design (a27.2)<br>• doubts about efficacy<br>reason: lack of data to sufficiently demonstrate efficacy (a27.3)	**/A17 /A1 /A27.1.5 /A27.2 /A27.3 //**	15/11/2019	**No authorisation, because of no positive benefit-risk ratio**	**EMA evaluation**	**Number of clinical studies 01**	**Participants 0001-0024**	**No placebo control**		**No comparison with approved drugs**	**No special drug approval procedure**	**No consequences for patients in ongoing clinical trials or compassionate use programmes**	Ongoing clinical trials (at the time the application was withdrawn)	Luxceptar - INN; VIABLE T-CELLS (europa.eu)	https://www.ema.europa.eu/en/documents/withdrawal-letter/withdrawal-letter-luxceptar_en.pdf	**Orphan drugs**	2/17/20
Axumin,	fluciclovine (18F)	Fluciclovine (18F)	Blue Earth Diagnostics Ireland Ltd	Blue Earth Diagnostics Ireland Ltd	**Other medicines**	**V09IX12**	**02 Neoplasms**<br><br>**X Extension codes (Medicines used in diagnostic tests, not elsewhere classified)**	ICD-11 Chapter X<br><br>ICD-11 Chapter 02	diagnostics and monitoring of gliomas in adults (in PET scan (positron emission tomography))<br><br>already approved indications: diagnostic drugs for prostate carcinoma (especially in PET scan)	**Transporter (N3.U)**	**/N3.U //**	Transporter (SLC-Transporter)(N3.1)	**/N3.1.U //**	**Post-authorisation**	5/21/17	2/11/20	• open questions from the EMA (a17)<br>• concerns regarding responses/information from the manufacturer (a1)<br>• doubts about efficacy<br>reason: lack of data to sufficiently demonstrate efficacy (a27.3)<br>• missing justification of the dosing regimen (use so far only in asia) (a9)<br>• doubts about efficacy<br>reason: other reasons (questionable differentiation between non-cancerous tumors, cancerous tumors and inflammatory diseases) (a27)	**/A17 /A1 /A27.3 /A27. /A9 //**	28/02/2020	**No extension of authorisation, because of no positive benefit-risk ratio**	**EMA evaluation**	**Number of clinical studies 04**	**Participants 0075-0099**	**No placebo control**	**SD**	**No comparison with approved drugs**	**No special drug approval procedure**	**No consequences for patients in ongoing clinical trials or compassionate use programmes**	Ongoing clinical trials (at the time the application was withdrawn)	Akynzeo; INN-netupitant/palonosetron or fosnetupitant/palonosetron (europa.eu)	https://www.ema.europa.eu/en/documents/withdrawal-letter/withdrawal-letter-axumin-ii-11_en.pdf	**Orphan drugs**	2/28/20
Linhaliq,	ciprofloxacine	Ciprofloxacin	Aradigm Pharmaceuticals Limited	Aradigm Pharmaceuticals Limited	**Antibacterial drugs**	**J01MA02**	**12 Respiratory diseases**	ICD-11 Chapter 12	bronchiectasis (in lung infections with Pseudomonas aeruginosa bacteria, not due to cystic fibrosis related bronchiectasis)	**other proteins (Antagonism/Binding) (N5.A)**	**/N5.A //**	other proteins (bacterial proteins / bacterial protein synthesis, Antagonism)(N5.9.A)	**/N5.9.A //**	**Initial authorisation**		10/29/19	• open questions from the EMA (a17)<br>• concerns regarding responses/information from the manufacturer (a1)<br>• concerns regarding medicinal product quality (lack of data on medicinal product quality) (a3)<br>• doubts about efficacy<br>reason: lack of data to sufficiently demonstrate efficacy (a27.3)	**/A17 /A1 /A27.3 /A3. //**	15/11/2019	**No authorisation, because of no positive benefit-risk ratio**	**EMA evaluation**	**Number of clinical studies 02**	**Participants 0500-0999**	**Placebo controlled clinical studies**		**No comparison with approved drugs**	**No special drug approval procedure**	**No consequences for patients in ongoing clinical trials or compassionate use programmes**	No ongoing clinical trials (at the time the application was withdrawn)	Linhaliq; INN-ciprofloxacin hydrochloride (europa.eu)	https://www.ema.europa.eu/en/documents/withdrawal-letter/withdrawal-letter-linhaliq_en.pdf	**No orphan drugs**	1/24/20
Opsumit,	macitentan	Macitentan	Janssen-Cilag International NV	Janssen Pharmaceutical Companies of Johnson & Johnson	**Endothelin receptor antagonist**	**C02KX04**	**11 Diseases of the circulatory system**	**ICD-11 Chapter 11**	chronic thromboembolic pulmonary hypertension (CTEPH) (use in severe symptomatic cases and where CTEPH is not operable)<br><br>already approved indications: pulmonary hypertension	**Receptors (Antagonism) (N4.A)**	**/N4.A //**	Receptors (GPCR, Antagonism) (N4.1.A)	**/N4.1.A //**	**Post-authorisation**	12/20/13	11/8/19	• open questions from the EMA (a17)<br>• concerns regarding responses/information from the manufacturer (a1)<br>• doubts about efficacy<br>reason: poor study quality (conduct) (a27.1.6)<br>• doubts about efficacy<br>reason: lack of data to sufficiently demonstrate efficacy (a27.3)<br>• lack of data to sufficiently demonstrate efficacy (a12)	**/A17 /A1 /A27.1.6 /A27.3 /A12 //**	15/11/2019	**No extension of authorisation, because of no positive benefit-risk ratio**	**EMA evaluation**	**Number of clinical studies 01**	**Participants 0075-0099**	**Placebo controlled clinical studies**		**No comparison with approved drugs**	**No special drug approval procedure**	**No consequences for patients in ongoing clinical trials or compassionate use programmes**	Ongoing clinical trials (at the time the application was withdrawn)	Opsumit_19-11_QandA on withdrawal of ext indication (europa.eu)	https://www.ema.europa.eu/en/documents/withdrawal-letter/withdrawal-letter-opsumit-ii-0029_en.pdf	**Orphan drugs**	2/17/20
Nuzyra,	omadacycline tosylate	Omadacycline	Paratek Ireland Limited	Paratek Ireland Limited	**Tetracycline**	**J01AA**	**12 Respiratory diseases/**<br><br>**14 Skin diseases**	ICD-11 Chapter 12<br><br>ICD-11 Chapter 14	community-acquired bacterial pneumonias, bacterial infections of the skin and soft tissues	**other (N6)**	**/N6.U //**	other (N6)	**/N6.U //**	**Initial authorisation**		10/17/19	withdrawal reasons only for the indication of community-acquired pneumonia:<br><br>• concerns regarding responses/information from the manufacturer (a1)<br>• doubts about efficacy<br>reason: lack of data to sufficiently demonstrate efficacy (a27.3)<br>• lack of data to sufficiently demonstrate efficacy (further studies needed) (a12)	**/A27.3 /A12 /A1 //**	18/10/2019	**No authorisation, because of no positive benefit-risk ratio**	**Marketing strategy**	**Number of clinical studies 03**	**Participants over 2000**	**No placebo control**		**Comparison with approved drugs**	**No special drug approval procedure**	**No consequences for patients in ongoing clinical trials or compassionate use programmes**	Ongoing clinical trials (at the time the application was withdrawn)	Nuzyra,INN-omadacycline tosylate (europa.eu)	https://www.ema.europa.eu/en/documents/withdrawal-letter/withdrawal-letter-nuzyra_en.pdf	**No orphan drugs**	2/5/20
Ekesivy,	diclofenamide	Diclofenamide	Sun Pharmaceutical Industries Europe B.V.	Sun Pharmaceutical Industries	**Other medicines**	**S01EC02**	**08 Diseases of the nervous system**	**ICD-11 Chapter 08**	familial (hereditary) periodic paralysis	**Enzyme (Antagonism)(N1.A)**	**/N1.A //**	Enzyme (Lyases, Antagonism)(N1.4.A)	**/N1.4.A //**	**Initial authorisation**		10/2/19	• open questions from the EMA (a17)<br>• concerns regarding responses/information from the manufacturer (a1)<br>• concerns regarding medicinal product quality (a3)<br>• doubts about assurance of patient safety (a21)<br>• outstanding questions on bioavailability, bioequivalence, distribution and metabolism (one or more topics) (a29)<br>• doubts about efficacy<br>reason: other reasons (no laboratory studies for comparison with reference medicinal product fenamide) (a27)<br>• doubts about efficacy<br>reason: poor study quality (conduct) (a27.1.6)	**/A17 /A1 /A27.1.3 /A3. /A21. /A29 /A27. /A27.1.6 //**	18/10/2019	**No authorisation, because of no positive benefit-risk ratio**	**EMA evaluation**	**Number of clinical studies 01**	**Participants 0050-0074**	**Placebo controlled clinical studies**	**SDH**	**No comparison with approved drugs**	**Drug application for hybrid medicines**	**No consequences for patients in ongoing clinical trials or compassionate use programmes**	No ongoing clinical trials (at the time the application was withdrawn)	Ekesivy - withdrawal Q&A (initial) (europa.eu)	https://www.ema.europa.eu/en/documents/withdrawal-letter/withdrawal-letter-ekesivy_en.pdf	**Orphan drugs**	3/6/20
Xyndari,	(glutamine)	Glutamine	Emmaus Medical Europe Ltd	Emmaus Medical Europe Ltd	**Other medicines**	**A16AA03**	**03 Diseases of the blood or blood-forming organs**	ICD-11 Chapter 03	sickle cell anemia	**other (N6)**	**/N6.U //**	other (N6)	**/N6.U //**	**Initial authorisation**		9/18/19	• concerns regarding responses/information from the manufacturer (a1)<br>• doubts about efficacy<br>reason: lack of data to sufficiently demonstrate efficacy (a27.3)<br>• doubts about efficacy<br>reason: other reasons (study discontinuation of many patients (especially with xyndari treatment), therefore missing information) (a27)<br>• doubts about efficacy<br>reason: poor study quality (data analysis) (a27.1.3)<br>• doubts about efficacy<br>reason: poor study quality (small participant number in supportive study) (a27.1.5)<br>• doubts about efficacy<br>reason: poor study quality (conduct of supportive study, as patients partly received hydroxyurea as medication for sickle cell anaemia) (a27.1.6)	**/A1 /A27.3 /A27. /A27.1.3 /A27.1.5 /A27.1.6 //**	18/10/2019	**No authorisation, because of no positive benefit-risk ratio**	**Marketing strategy**	**Number of clinical studies 02**	**Participants 0300-0499**	**Placebo controlled clinical studies**		**No comparison with approved drugs**	**No special drug approval procedure**	**No consequences for patients in ongoing clinical trials or compassionate use programmes**	No ongoing clinical trials (at the time the application was withdrawn)	Xyndari; INN-glutamine (europa.eu)	https://www.ema.europa.eu/en/documents/withdrawal-letter/withdrawal-letter-xyndari_en.pdf	**Orphan drugs**	2/11/20
ABP 710,	infliximab	Infliximab	Amgen Europe B.V.	Amgen Europe B.V.	**Monoclonal antibodies**	**L04AB02**	**15 Diseases of the musculoskeletal system or connective tissue/**<br><br>**13 Diseases of the digestive system /**<br><br>**14 Skin diseases**	ICD-11 Chapter 15<br><br>ICD-11 Chapter 13<br><br>ICD-11 Chapter 14	rheumatoid arthritis, Crohn's disease, ulcerative colitis, ankylosing spondylitis, psoriatic arthritis, psoriasis	**Ligands (antagonism)(N9.A)**	**/N9.A //**	Ligands (Cytokines, Antagonism)(N9.2.A)	**/N9.2.A //**	**Initial authorisation**		5/27/19	not available (documents have not yet been evaluated or recommendations not yet published) (a14.1)<br><br>note:<br>documents had not been evaluated at the time of withdrawal of the application.	**/A14.1 //**	28/06/2019	**None (documents have not yet been evaluated)**	**Marketing strategy**	**Number of clinical studies 01**	**Participants 0500-0999**	**No placebo control**	**SDB**	**Comparison with approved drugs**	**Drug application for biosimilars**	**No consequences for patients in ongoing clinical trials or compassionate use programmes**	No ongoing clinical trials (at the time the application was withdrawn)	ABP 710_19-06_withdrawal Q&A (europa.eu)	https://www.ema.europa.eu/en/documents/withdrawal-letter/withdrawal-letter-abp-710_en.pdf	**No orphan drugs**	6/28/19

Radicava,	edaravone	Edaravone	Mitsubishi Tanabe Pharma GmbH	Mitsubishi Tanabe Pharma GmbH	**Other medicines**	**N07**	**08 Diseases of the nervous system**	ICD-11 Chapter 08	amyotrophic lateral sclerosis (ALS)<br><br>(use to inhibit the progression of symptoms in patients who can perform normal daily activities)	**other (N6)**	**/N6.U<br>//**	other (N6)	**/N6.U<br>//**	**Initial authorisation**		5/24/19	• open questions from the EMA (a17)<br>• concerns regarding responses/information from the manufacturer (a1)<br>• doubts about assurance of patient safety<br>reason: lack of data and/or inadequate long-term observation (a21.3)<br>• doubts about efficacy<br>reason: poor study quality (selection of participants) (a27.1.7)<br>• doubts about efficacy<br>reason: poor study quality (small participant number) (a27.1.5)<br>• lack of data to sufficiently demonstrate efficacy (proposed registry study considered unsuitable by EMA) (a12)<br>• doubts about efficacy<br>reason: lack of data to sufficiently demonstrate efficacy (few patients, hardly any evidence for other important indicators of efficacy) (a27.3)	**/A17<br>/A1<br>/A27.3<br>/A21.3<br>/A27.1.7<br>/A27.1.5<br>/A12<br>//**	29/05/2019	**No authorisation, because of no positive benefit-risk ratio**	**EMA evaluation**	**Number of clinical studies 01**	**Participants 0100-0149**	**Placebo controlled clinical studies**		**No comparison with approved drugs**	**No special drug approval procedure**	**No consequences for patients in ongoing clinical trials or compassionate use programmes**	Ongoing clinical trials (at the time the application was withdrawn)	Radicava, INN-edaravone (europa.eu)	https://www.ema.europa.eu/en/documents/withdrawal-letter/withdrawal-letter-radicava_en.pdf	**Orphan drugs**	8/14/19
Ambrisentan Zentiva,	ambrisentan	Ambrisentan	Zentiva, k.s.	Zentiva, k.s.	**Endothelin receptor antagonist**	**C02KX02**	**11 Diseases of the circulatory system**	ICD-11 Chapter 11	pulmonary arterial hypertension (PAH)<br><br>(use alone or in combination in severity classes II-III)	**Receptors (Antagonism) (N4.A)**	**/N4.A<br>//**	Receptors<br>(GPCR, Antagonism) (N4.1.A)	**/N4.1.A<br>//**	**Initial authorisation**		4/29/19	• open questions from the EMA (a17)<br>• concerns regarding responses/information from the manufacturer (a1)<br>• concerns regarding medicinal product quality (manufacturing process) (a3.1)<br>• outstanding questions on bioavailability, bioequivalence, distribution and metabolism (one or more topics) related to lower dose (5 mg) (a29)	**/A17<br>/A1<br>/A3.1<br>/A29<br>//**	29/05/2019	**No authorisation, because of no positive benefit-risk ratio**	**EMA evaluation**	**Number of clinical studies 0**	**Participants 0**	**No placebo control**	**SDG**	**Comparison with approved drugs**	**Drug application for generic medicines**	**No consequences for patients in ongoing clinical trials or compassionate use programmes**	No ongoing clinical trials (at the time the application was withdrawn)	Ambrisentan Zentiva, INN-ambrisentan (europa.eu)		**No orphan drugs**	5/29/19
Epjevy,	pacritinib citrate	Pacritinib Citrate	CTI Life Sciences Limited	CTI Life Sciences Limited	**Kinase inhibitors**	**L01XE**	**02 Neoplasms /**<br><br>**X Extension codes (Chronic myeloproliferative diseases, malignant)**	ICD-11 Chapter 02<br><br>ICD-11 Chapter X	myelofibrosis<br>(in patients with severe thrombocytopenia)	**Enzyme (Antagonism)(N1.A)**<br><br>**Receptors (Antagonism)(N4.A)**	**/N1.A<br>/N4.A<br>//**	Enzyme<br>(Transferases, Antagonism)(N1.2.A)<br><br>Receptors<br>(catalytic receptors, Antagonism)(N4.3.A)	**/N1.2.A<br>/N4.3.A<br>//**	**Initial authorisation**		2/7/19	• open questions from the EMA (a17)<br>• concerns regarding responses/information from the manufacturer (a1)<br>• concerns regarding recommended dose (a6)<br>• doubts about efficacy<br>reason: lack of data to sufficiently demonstrate efficacy (with respect to symptom relief) (a27.3)	**/A17<br>/A1<br>/A27.3<br>/A6<br>//**	1/03/2019	**No authorisation, because of no positive benefit-risk ratio**	**EMA evaluation**	**Number of clinical studies 02**	**Participants 0500-0999**	**No placebo control**		**Comparison with approved drugs**	**No special drug approval procedure**	**No consequences for patients in ongoing clinical trials or compassionate use programmes**	Ongoing clinical trials (at the time the application was withdrawn)	Epjevy; INN-pacritinib citrate (europa.eu)	https://www.ema.europa.eu/en/documents/withdrawal-letter/withdrawal-letter-epjevy_en.pdf	**Orphan drugs**	3/1/19
Vynpenta (previously AvacopanChemoCentryx),	Avacopan<br><br>INN Schreibweise vorher korrigieren!!	Avacopan	ChemoCentryx Ltd	ChemoCentryx Ltd	**Immunomodulators**	**L04**	**04 Diseases of the immune system**	ICD-11 Chapter 04	granulomatosis with polyangiitis (GPA), microscopic polyangiitis (MPA)<br><br>Note:<br>Use in combination with cyclophosphamide or rituximab	**Receptors (Antagonism) (N4.A)**	**/N4.A<br>//**	Receptors<br>(GPCR, Antagonism) (N4.1.A)	**/N4.1.A<br>//**	**Initial authorisation**		1/23/19	• open questions from the EMA (a17)<br>• concerns regarding responses/information from the manufacturer (a1)<br>• doubts about efficacy<br>reason: inadequate study design (a27.2)<br>• doubts about assurance of patient safety<br>reason: side effects (a21.1)<br>• concerns regarding medicinal product quality (a3)<br>• doubts about efficacy<br>reason: lack of data to sufficiently demonstrate efficacy (a27.3)	**/A17<br>/A1<br>/A27.2<br>/A27.3<br>/A21.1<br>/A3.<br>//**	1/02/2019	**No authorisation, because of no positive benefit-risk ratio**	**EMA evaluation**	**Number of clinical studies 02**	**Participants 0100-0149**	**No placebo control**		**Comparison with approved drugs**	**No special drug approval procedure**	**No consequences for patients in ongoing clinical trials or compassionate use programmes**	Ongoing clinical trials (at the time the application was withdrawn)	"Vynpenta, INN-Avacopan" (europa.eu)	https://www.ema.europa.eu/en/documents/withdrawal-letter/withdrawal-letter-vynpenta_en.pdf	**Orphan drugs**	5/15/20
Efgratin,	pegfilgrastim	Pegfilgrastim	Gedeon Richter Plc.	Gedeon Richter Plc.	**Immunomodulators**	**L03AA13**	**04 Diseases of the immune system**	ICD-11 Chapter 04	neutropenia,<br>reduction in the occurrence of febrile neutropenia (in connection with cancer therapies)	**Receptors (Agonism) (N4.B)**	**/N4.B<br>//**	Receptors<br>(catalytic receptors, Agonism) (N4.3.B)	**/N4.3.B<br>//**	**Initial authorisation**		12/20/18	• open questions from the EMA (a17)<br>• concerns regarding responses/information from the manufacturer (a1)<br>• doubts about equivalent/improved efficacy (bioequivalence) compared to reference medicinal product (neulasta) (a25)<br>• doubts about assurance of patient safety<br>reason: side effects (a21.1)<br>• doubts about efficacy<br>reason: poor study quality (a27.1)	**/A17<br>/A1<br>/A25<br>/A21.1<br>/A27.1<br>//**	1/02/2019	**No authorisation, because of no positive benefit-risk ratio**	**EMA evaluation**	**Number of clinical studies 01**	**Participants 0200-0299**	**No placebo control**	**SDB**	**Comparison with approved drugs**	**Drug application for biosimilars**	**No consequences for patients in ongoing clinical trials or compassionate use programmes**	No ongoing clinical trials (at the time the application was withdrawn)	Efgratin; INN-pegfilgrastim (europa.eu)	https://www.ema.europa.eu/en/documents/withdrawal-letter/withdrawal-letter-efgratin_en.pdf-0	**No orphan drugs**	4/5/19
Cavoley,	pegfilgrastim	Pegfilgrastim	Stada Arzneimittel AG	Stada Arzneimittel AG	**Immunomodulators**	**L03AA13**	**04 Diseases of the immune system**	ICD-11 Chapter 04	neutropenia,<br>reduction in the occurrence of febrile neutropenia (in connection with cancer therapies)	**Receptors (Agonism) (N4.B)**	**/N4.B<br>//**	Receptors<br>(catalytic receptors, Agonism) (N4.3.B)	**/N4.3.B<br>//**	**Initial authorisation**		12/20/18	• open questions from the EMA (a17)<br>• concerns regarding responses/information from the manufacturer (a1)<br>• doubts about equivalent/improved efficacy (bioequivalence) compared to reference medicinal product (neulasta) (a25)<br>• doubts about assurance of patient safety<br>reason: side effects (a21.1)<br>• doubts about efficacy<br>reason: poor study quality (a27.1)	**/A17<br>/A1<br>/A25<br>/A21.1<br>/A27.1<br>//**	1/02/2019	**No authorisation, because of no positive benefit-risk ratio**	**EMA evaluation**	**Number of clinical studies 01**	**Participants 0200-0299**	**No placebo control**	**SDB**	**Comparison with approved drugs**	**Drug application for biosimilars**	**No consequences for patients in ongoing clinical trials or compassionate use programmes**	No ongoing clinical trials (at the time the application was withdrawn)	Cavoley; INN-pegfilgrastim (europa.eu)	https://www.ema.europa.eu/en/documents/withdrawal-letter/withdrawal-letter-cavoley_en.pdf-0	**No orphan drugs**	4/5/19
Fyzoclad,	adalimumab	Adalimumab	Pfizer Europe MA EEIG	Pfizer	**Monoclonal antibodies**	**L04AB04**	**15 Diseases of the musculoskeletal system or connective tissue/**<br><br>**14 Skin diseases/**<br><br>**09 Diseases of the visual system**	ICD-11 Chapter 15<br><br>ICD-11 Chapter 14<br><br>ICD-11 Chapter 09	rheumatoid arthritis, juvenile idiopathic arthritis,<br>axial spondyloarthritis,<br>psoriatic arthritis,<br>psoriasis and pediatric plaque psoriasis, uveitis and pediatric uveitis	**Ligands (antagonism)(N9.A)**	**/N9.A<br>//**	Ligands<br>(Cytokines, Antagonism)(N9.2.A)	**/N9.2.A<br>//**	**Initial authorisation**		12/5/18	• not available (documents have not yet been evaluated or recommendations not yet published) (a14.1)<br><br>note:<br>documents had not been fully evaluated at the time of withdrawal of the application.	**/A14.1<br>//**	14/12/2018	**None (documents have not yet been evaluated)**	**Marketing strategy**	**Number of clinical studies 01**	**Participants unknown**	**No placebo control**	**SDB**	**Comparison with approved drugs**	**Drug application for biosimilars**	**No consequences for patients in ongoing clinical trials or compassionate use programmes**	No ongoing clinical trials (at the time the application was withdrawn)	Fyzoclad, INN-adalimumab (europa.eu)	https://www.ema.europa.eu/en/documents/withdrawal-letter/withdrawal-letter-fyzoclad_en.pdf	**No orphan drugs**	12/14/18
Canakinumab Novartis,	canakinumab	Canakinumab	Novartis Europharm Limited	Novartis	**Monoclonal antibodies**	**L04AC08**	**11 Diseases of the circulatory system/**<br><br>**08 Diseases of the nervous system**	ICD-11 Chapter 11<br><br>ICD-11 Chapter 08	acute coronary syndrome, myocardial infarction, stroke<br><br>(use after having had a myocardial infarction to prevent serious events such as strokes, myocardial infarctions, and death)	**Ligands (antagonism)(N9.A)**	**/N9.A<br>//**	Ligands<br>(Cytokines, Antagonism)(N9.2.A)	**/N9.2.A<br>//**	**Initial authorisation**		12/4/18	• open questions from the EMA (a17)<br>• concerns regarding responses/information from the manufacturer (a1)<br>• doubts about efficacy<br>reason: lack of data to sufficiently demonstrate efficacy (a27.3)<br>• doubts about efficacy<br>reason: poor study quality (selection of participants) (a27.1.7)<br>• doubts about benefit (a18)<br>• doubts about assurance of patient safety<br>reason: side effects (a21.1)<br>• doubts about efficacy<br>reason: poor study quality (conduct) (a27.1.6)	**/A17<br>/A1<br>/A27.3<br>/A27.1.7<br>/A18<br>/A21.1<br>/A27.1.6<br>//**	14/12/2018	**No authorisation, because of no positive benefit-risk ratio**	**EMA evaluation**	**Number of clinical studies 01**	**Participants 2000**	**Placebo controlled clinical studies**		**No comparison with approved drugs**	**No special drug approval procedure**	**Consequences for patients in ongoing clinical trials or compassionate use programmes**	Ongoing clinical trials (at the time the application was withdrawn)	Canakinumab Novartis | Europäische Arzneimittel-Agentur (europa.eu)	https://www.ema.europa.eu/en/documents/withdrawal-letter/withdrawal-letter-canakinumab-novartis_en.pdf	**No orphan drugs**	5/7/19
Treprostinil SciPharm Sàrl	treprostinil	Treprostinil	SciPharm Sàrl	SciPharm Sàrl	**Prostacyclin analogs**	**B01AC21**	**11 Diseases of the circulatory system**	**ICD-11 Chapter 11**	chronic thromboembolic pulmonary hypertension (CTEPH)<br><br>Note:<br>Use when surgery is not possible or in recurrent CTEPH	**Receptors (Agonism) (N4.B)**	**/N4.B<br>//**	Receptors<br>(GPCR, Agonism) (N4.1.B)	**/N4.1.B<br>//**	**Initial authorisation**		9/5/18	• not available (documents have not yet been evaluated or recommendations not yet published) (a14.1)<br><br>note:<br>documents had not been fully evaluated at the time of withdrawal of the application.	**/A14.1<br>//**	21/09/2018	**None (documents have not yet been evaluated)**	**EMA evaluation**	**Number of clinical studies 01**	**Participants 0100-0149**	**No placebo control**		**No comparison with approved drugs**	**No special drug approval procedure**	**No consequences for patients in ongoing clinical trials or compassionate use programmes**	Ongoing clinical trials (at the time the application was withdrawn)	Treprostinil, INN-treprostinil (europa.eu)	https://www.ema.europa.eu/en/documents/withdrawal-letter/withdrawal-letter-treprostinil-scipharm-sarl_en.pdf	**Orphan drugs**	9/21/18
Entolimod TMC,	entolimod	Entolimod	TMC Pharma Services Ltd	TMC Pharma Services Ltd	**Immunomodulators**	**L03A**	**X Extension codes (Dimensions of external causes)**	ICD-11 Chapter X	acute radiation syndrome<br>(in nuclear accidents)	**Receptors (Agonism) (N4.B)**	**/N4.B<br>//**	Receptors<br>(catalytic receptors, Agonism) (N4.3.B)	**/N4.3.B<br>//**	**Initial authorisation**		7/31/18	• open questions from the EMA (a17)<br>• concerns regarding responses/information from the manufacturer (a1)<br>• concerns regarding medicinal product quality (a3)<br>• doubts about assurance of patient safety<br>reason: lack of data and/or inadequate long-term observation (study conducted in monkeys) (a21.3)	**/A17<br>/A1<br>/A3.<br>/A21.3<br>//**	26/09/2018	**No authorisation, because of no positive benefit-risk ratio**	**EMA evaluation**	**Number of clinical studies 02**	**Participants 0150-0199**	**No placebo control**		**No comparison with approved drugs**	**No special drug approval procedure**	**No consequences for patients in ongoing clinical trials or compassionate use programmes**	No ongoing clinical trials (at the time the application was withdrawn)	Entolimod TMC; INN-entolimod (europa.eu)	https://www.ema.europa.eu/en/documents/withdrawal-letter/withdrawal-letter-entolimod-tmc_en.pdf	**Orphan drugs**	12/12/18
Opdivo,	nivolumab	Nivolumab	Bristol-Myers Squibb Pharma EEIG	Bristol-Myers Squibb Pharma EEIG	**Monoclonal antibodies**	**L01FF01**	**02 Neoplasms**	ICD-11 Chapter 02	gastric cancer, gastroesophageal junction cancer<br><br>already approved indications:<br>melanoma, Hodgkin lymphoma, renal cell carcinoma, non-small cell lung carcinoma, squamous cell carcinoma of the esophagus, gastroesophageal junction cancer, squamous cell carcinoma of the head and neck (SCCHN), urothelial carcinoma, malignant pleural mesothelioma, colorectal carcinoma, gastric carcinoma	**other proteins (Antagonism/Binding) (N5.A)**	**/N5.A<br>//**	other proteins<br>(Immune checkpoint proteins/CD-molecules, Antagonism/Binding) (N5.4.A)	**/N5.4.A<br>//**	**Post-authorisation**	6/19/15	6/27/18	• open questions from the EMA (a17)<br>• concerns regarding responses/information from the manufacturer (a1)<br>• lack of data to sufficiently demonstrate efficacy (a12)<br>• doubts about benefit (improvement in survival time approx. 1 month) (a18)<br>• doubts about efficacy<br>reason: poor study quality (selection of participants, because no european patients) (a27.1.7)	**/A17<br>/A1<br>/A18<br>/A27.1.7<br>/A12<br>//**	27/07/2018	**No extension of authorisation, because of no positive benefit-risk ratio**	**EMA evaluation**	**Number of clinical studies 01**	**Participants 0300-0499**	**Placebo controlled clinical studies**		**No comparison with approved drugs**	**No special drug approval procedure**	**No consequences for patients in ongoing clinical trials or compassionate use programmes**	Ongoing clinical trials (at the time the application was withdrawn)	Withdrawal of application for a change to the marketing authorisation for Opdivo (nivolumab) (europa.eu)	https://www.ema.europa.eu/en/documents/withdrawal-letter/withdrawal-letter-opdivo-ii0039_en.pdf	**No orphan drugs**	12/11/18
Tecentriq,	atezolizumab	Atezolizumab	Roche Registration GmbH	Roche Registration GmbH	**Monoclonal antibodies**	**L01XC32**	**02 Neoplasms/**<br><br>**X Extension codes (Adenomas and adenocarcinomas)**	ICD-11 Chapter 02<br><br>ICD-11 Chapter X	advanced or metastatic renal cell carcinoma (in combination with bevacizumab)<br><br>already approved indications:<br>urological carcinoma, non-small cell lung cancer (NSCLC) (early stage or metastatic, small cell lung cancer (SCLC), triple-negative breast carcinoma, hepatocellular carcinoma	**Ligands (antagonism)(N9.A)**	**/N9.A<br>//**	Ligands<br>(PD-L1, Antagonism/Binding)<br>(N9.A)	**/N9.A<br>//**	**Post-authorisation**	9/20/17	10/22/18	• not available (documents have not yet been evaluated or recommendations not yet published) (a14.1)<br><br>note:<br>documents had not been evaluated at the time of withdrawal of the application.	**/A14.1<br>//**	16/11/2018	**None (documents have not yet been evaluated)**	**Other reasons**	**Number of clinical studies 01**	**Participants 0500-0999**	**No placebo control**		**Comparison with approved drugs**	**No special drug approval procedure**	**No consequences for patients in ongoing clinical trials or compassionate use programmes**	Ongoing clinical trials (at the time the application was withdrawn)	Tecentriq_18-11_withdrawal Q&A (europa.eu)	https://www.ema.europa.eu/en/documents/withdrawal-letter/withdrawal-letter-tecentriq_en.pdf	**No orphan drugs**	11/16/18

Raligize,	axalimogen filolisbac	Axalimogene Filolisbac	FGK Representative Service GmbH	FGK Representative Service GmbH	**Other medicines**	**L01X**	**02 Neoplasms**	ICD-11 Chapter 02	cervical cancer (use in cases of unsuccessful first treatment or recurrences)	**other (N6)**	**/N6.U<br>//**	other (N6)	**/N6.U<br>//**	**Initial authorisation**		7/10/18	• not available (documents have not yet been evaluated or recommendations not yet published) (a14.1)<br><br>remark:<br>documents had not been fully evaluated at the time of withdrawal of the application.	**/A14.1<br>//**	27/07/2018	**None (documents have not yet been evaluated)**	**Suspected EMA evaluation**	**Number of clinical studies 01**	**Participants 0050-0074**	**No placebo control**		**Comparison with approved drugs**	**No special drug approval procedure**	**No consequences for patients in ongoing clinical trials or compassionate use programmes**	Ongoing clinical trials (at the time the application was withdrawn)	Raligize, INN-axalimogene filolisbac (europa.eu)	https://www.ema.europa.eu/en/documents/withdrawal-letter/withdrawal-letter-raligize_en.pdf	**No orphan drugs**	7/27/18
Sutent,	sunitinib	Sunitinib	Pfizer Limited	Pfizer	**Kinase inhibitors**	**L01EX01**	**02 Neoplasms/<br><br>X Extension codes (Adenomas and adenocarcinomas)**	ICD-11 Chapter 02<br><br>ICD-11 Chapter X	delay or prevention of recurrences in patients with a high risk of recurrence after surgical procedures with renal cell carcinomas<br><br>already approved indications:<br>gastrointestinal stromal tumors, pancreatic neuroendocrine tumors, metastatic renal cell carcinomas	**Receptors (Antagonism) (N4.A)**	**/N4.A<br>//**	Receptors (catalytic receptors, Antagonism) (N4.3.A)	**/N4.3.A<br>//**	**Post-authorisation**	19.07.2006<br><br>(conditional Marketing Authorisation 27.04.2006)	6/26/18	• concerns regarding responses/information from the manufacturer (a1)<br>• doubts about assurance of patient safety<br>reason: side effects (a21.1)<br>• doubts about efficacy<br>reason: lack of data to sufficiently demonstrate efficacy (a27.3)<br>• doubts about benefit (a18)	**/A1<br>/A21.1<br>/A27.3<br>/A18<br>//**	27/07/2018	**No extension of authorisation, because of no positive benefit-risk ratio**	**EMA evaluation**	**Number of clinical studies 01**	**Participants 0500-0999**	**Placebo controlled clinical studies**		**No comparison with approved drugs**	**No special drug approval procedure**	**No consequences for patients in ongoing clinical trials or compassionate use programmes**	Ongoing clinical trials (at the time the application was withdrawn)	Sutent; INN-sunitinib (europa.eu)	https://www.ema.europa.eu/en/documents/withdrawal-letter/withdrawal-letter-sutent_en.pdf	**Orphan drugs**	8/17/18
Graspa,	L-asparaginase	L-Asparaginase	ERYTECH Pharma S.A.	ERYTECH Pharma S.A.	**Cytostatics**	**LX1XX02**	**02 Neoplasms/<br><br>X Extension codes (Leukemias)**	ICD-11 Chapter 02<br><br>ICD-11 Chapter X	acute lymphoblastic leukemia (ALL) ("Philadelphia chromosome negative")<br><br>(use in combination with other cancer drugs in adults and children)	**Ligands (antagonism)(N9.A)**	**/N9.A<br>//**	Ligands (Asparagine, Antagonism (degradation)) (N9.A)	**/N9.A<br>//**	**Initial authorisation**		6/22/18	• open questions from the EMA (a17)<br>• concerns regarding responses/information from the manufacturer (a1)<br>• doubts about same/improved efficacy compared with the eu reference medicinal product (a25)<br>• open questions on bioavailability, bioequivalence, distribution and metabolism (for one or more topics) (a29)<br>• doubts about efficacy<br>reason: other reasons (asparaginase in studies may not be the same as the asparaginase in the medicinal product) (a27)<br>• doubts about assurance of patient safety<br>reason: lack of data and/or inadequate long-term follow-up (a21.3)<br>• doubts about efficacy<br>reason: poor study quality (a27.1)	**/A17<br>/A1<br>/A25<br>/A27.<br>/A29<br>/A21.3<br>/A27.1<br>//**	29/06/2018	**No authorisation, because of no positive benefit-risk ratio**	**EMA evaluation**	**Number of clinical studies 01**	**Participants 0075-0099**	**No placebo control**		**Comparison with approved drugs**	**No special drug approval procedure**	**No consequences for patients in ongoing clinical trials or compassionate use programmes**	Ongoing clinical trials (at the time the application was withdrawn)	Graspa; INN-L-asparaginase (europa.eu)	https://www.ema.europa.eu/en/documents/withdrawal-letter/withdrawal-letter-graspa_en.pdf	**Orphan drugs**	1/23/19
Restaysis,	ciclosporin	Cyclosporine	Allergan Pharmaceuticals International Limited	Allergan Pharmaceuticals International Limited	**Immunosuppressants (no monoclonal antibodies)**	**S01XA18**	**09 Diseases of the visual system**	ICD-11 Chapter 09	dry eye syndrome (Sicca syndrome), moderate<br><br>Note:<br>Use in insufficient efficacy of tear substitutes or ocular lubricants	**Enzyme (Antagonism)(N1.A)**	**/N1.A<br>//**	Enzyme (Hydrolases, Antagonism)(N1.3.A)	**/N1.3.A<br>//**	**Initial authorisation**		4/25/18	• open questions from the EMA (a17)<br>• concerns regarding responses/information from the manufacturer (a1)<br>• doubts about efficacy<br>reason: poor study quality (data analysis) (a27.1.3)<br>• doubts about efficacy<br>reason: lack of data to sufficiently demonstrate efficacy (a27.3)	**/A17<br>/A1<br>/A27.1.3<br>/A27.3<br>//**	1/06/2018	**No authorisation, because of no positive benefit-risk ratio**	**EMA evaluation**	**Number of clinical studies 0**	**Participants unknown**	**placebo control unknown**	**SD**	**Comparison with approved drugs unknown**	**No special drug approval procedure**	**No consequences for patients in ongoing clinical trials or compassionate use programmes**	Ongoing clinical trials (at the time the application was withdrawn)	Restaysis, INN-ciclosporin (europa.eu)	https://www.ema.europa.eu/en/documents/withdrawal-letter/withdrawal-letter-restaysis_en.pdf	**No orphan drugs**	8/6/18
Prohippur,	Sodium benzoate	Sodium benzoate	Lucane Pharma	Lucane Pharma	**Other medicines**	**A16AX11**	**05 Endocrine diseases, nutritional or metabolic diseases**	ICD-11 Chapter 05	non-ketotic hyperglycinemia (hereditary disease), urea cycle disorders (hereditary disease)	**other (N6)**	**/N6.U<br>//**	other (N6)	**/N6.U<br>//**	**Initial authorisation**		4/3/18	• open questions from the EMA (a17)<br>• concerns regarding responses/information from the manufacturer (a1)<br>• doubts about assurance of patient safety (a21)<br>• doubts about efficacy<br>reason: lack of data to sufficiently demonstrate efficacy (a27.3)<br>• concerns about medicinal product quality (high concentration of butylated methacrylate copolymer, concerns about quality control) (a3)<br>• concerns regarding the form of application (a2)<br>• doubts about efficacy<br>reason: other reasons (lack of studies on comparable mode of action of prohippur granules with sodium benzoate) (a27)	**/A17<br>/A1<br>/A21.<br>/A27.3<br>/A3.<br>/A2<br>/A27.<br>//**	27/04/2018	**No authorisation, because of no positive benefit-risk ratio**	**EMA evaluation**	**Number of clinical studies 0**	**Participants 0**	**No placebo control**	**SD**	**No comparison with approved drugs**	**No special drug approval procedure**	**No consequences for patients in ongoing clinical trials or compassionate use programmes**	No ongoing clinical trials (at the time the application was withdrawn)	Prohippur, INN-sodium benzoate (europa.eu)	https://www.ema.europa.eu/en/documents/withdrawal-letter/withdrawal-letter-prohippur_en.pdf	**Orphan drugs**	7/5/18
Balimek,	binimetinib	Binimetinib	Pierre Fabre Médicament	Pierre Fabre Médicament	**Kinase inhibitors**	**L01XE**	**02 Neoplasms**	ICD-11 Chapter 02	melanoma (unresectable or highly advanced with NRAS Q61 mutation)	**Enzyme (Antagonism)(N1.A)**	**/N1.A<br>//**	Enzyme (Transferases, Antagonism)(N1.2.A)	**/N1.2.A<br>//**	**Initial authorisation**		1/4/18	• open questions from the EMA (a17)<br>• concerns regarding responses/information from the manufacturer (a1)<br>• doubts about assurance of patient safety<br>reason: side effects (stronger than with reference medicinal product dacarbazine) (a21.1)<br>• doubts about same/improved efficacy compared with the eu reference medicinal product (a25)<br>• doubts about efficacy<br>reason: lack of data to sufficiently demonstrate efficacy (a27.3)	**/A17<br>/A1<br>/A21.1<br>/A25<br>/A27.3<br>//**	26/01/2018	**No authorisation, because of no positive benefit-risk ratio**	**EMA evaluation**	**Number of clinical studies 01**	**Participants 0300-0499**	**No placebo control**		**Comparison with approved drugs**	**No special drug approval procedure**	**No consequences for patients in ongoing clinical trials or compassionate use programmes**	Ongoing clinical trials (at the time the application was withdrawn)	Balimek, INN-binimetinib (europa.eu)	https://www.ema.europa.eu/en/documents/withdrawal-letter/withdrawal-letter-balimek_en.pdf	**No orphan drugs**	3/27/18
Rotigotine Mylan,	rotigotine	Rotigotine	Mylan S.A.S	Mylan	**Parkinson's therapeutics**	**N04BC09**	**08 Diseases of the nervous system<br><br>07 Sleep-wake disorders**	ICD-11 Chapter 08<br><br>ICD-11 Chapter 07	restless legs syndrome, Parkinson's disease	**Receptors (Agonism) (N4.B)**	**/N4.B<br>//**	Receptors (GPCR, Agonism) (N4.1.B)	**/N4.1.B<br>//**	**Initial authorisation**		12/22/17	• open questions from the EMA (a17)<br>• concerns regarding responses/information from the manufacturer (a1)<br>• concerns regarding medicinal product quality (manufacturing process) (a3.1)<br>• concerns regarding medicinal product quality (a3)<br>• doubts about efficacy<br>reason: poor study quality (selection of participants, as patients in the patch study were young adults while the indication mostly applies to older people) (a27.1.7)	**/A17<br>/A1<br>/A3.1<br>/A3.<br>/A27.1.7<br>//**	26/01/2018	**No authorisation, because of no positive benefit-risk ratio**	**EMA evaluation**	**Number of clinical studies 01**	**Participants 0200-0299**	**No placebo control**	**SDG**	**Comparison with approved drugs**	**Drug application for generic medicines**	**No consequences for patients in ongoing clinical trials or compassionate use programmes**	No ongoing clinical trials (at the time the application was withdrawn)	Rotigotine Mylan, INN-rotigotine (europa.eu)	https://www.ema.europa.eu/en/documents/withdrawal-letter/withdrawal-letter-rotigotine-mylan_en.pdf	**No orphan drugs**	7/11/18
Qtern,	saxagliptin, dapagliflozin	Saxagliptin, Dapagliflozin Propenediol Monohydrate	AstraZeneca AB	AstraZeneca	**Other antidiabetics**	**A10BD21**	**05 Endocrine diseases, nutritional or metabolic diseases**	ICD-11 Chapter 05	previous application when combination with other diabetes medications for type 2 diabetes management is not sufficiently effective (e.g. metformin and/or sulfonylurea plus saxagliptin or dapagliflozin)<br><br>already approved indications:<br>type 2 diabetes<br>(use when combination with other diabetes drugs is not adequately effective (e.g. metformin and/or sulfonylurea plus saxagliptin or dapagliflozin) or as a replacement for saxagliptin and dapagliflozin when taken separately)<br>nutritional and metabolic disorders, glucose metabolism disorders	**Enzyme (Antagonism)(N1.A)<br><br>Transporter (Antagonism)(N3.A)**	**/N1.A<br>/N3.A<br>//**	Transporter (SLC-Transporters, Antagonism)(N3.1.A)<br><br>Enzyme (Hydrolases, Antagonism)(N1.3.A)	**/N3.1.A<br>/N1.3.A<br>//**	**Post-authorisation**	7/15/16	4/3/18	• not available (documents have not yet been evaluated or recommendations not yet published) (a14.1)<br><br>remark:<br>documents had not been fully evaluated at the time of withdrawal of the application.	**/A14.1<br>//**	27/04/2018	**None (documents have not yet been evaluated)**	**Suspected EMA evaluation**	**Number of clinical studies 02**	**Participants over 2000**	**No placebo control**		**Comparison with approved drugs**	**No special drug approval procedure**	**No consequences for patients in ongoing clinical trials or compassionate use programmes**	Ongoing clinical trials (at the time the application was withdrawn)	Qtern withdrawal QandA (europa.eu)	https://www.ema.europa.eu/en/documents/withdrawal-letter/withdrawal-letter-qtern_en.pdf	**No orphan drugs**	4/27/18
Aranesp,	darbepoetin alfa	Darbepoetin alfa	Amgen Europe B.V.	Amgen Europe B.V.	**Other medicines**	**B03XA02**	**02 Neoplasms**	ICD-11 Chapter 02	anemia in patients with myelodysplastic syndromes (when frequent blood transfusions are not needed and the risk of acute myeloid leukemia (AML) is low to medium)<br><br>already approved indications:<br>chronic kidney insufficiency (adults and children), adults receiving chemotherapy for "non-myeloid cancer"	**Receptors (Agonism) (N4.B)**	**/N4.B<br>//**	Receptors (catalytic receptors, Agonism) (N4.3.B)	**/N4.3.B<br>//**	**Post-authorisation**	6/8/01	2/21/18	• open questions from the EMA (a17)<br>• concerns regarding responses/information from the manufacturer (a1)<br>• doubts about efficacy<br>reason: poor study quality (change of study design during the study, inadequate indicators of efficacy) (a27.1.1)<br>• doubts about efficacy<br>reason: poor study quality (data analysis, exclusion of a large number of patients from the evaluation) (a27.1.3)<br>• uncertainties regarding the requested indication (a19)	**/A17<br>/A1<br>/A27.1.1<br>/A27.1.3<br>/A19<br>//**	23/03/2018	**No extension of authorisation, because of no positive benefit-risk ratio**	**EMA evaluation**	**Number of clinical studies 02**	**Participants 0300-0499**	**Placebo controlled clinical studies**		**No comparison with approved drugs**	**No special drug approval procedure**	**No consequences for patients in ongoing clinical trials or compassionate use programmes**	Ongoing clinical trials unknown (at the time the application was withdrawn)	Aranesp, INN-darbepoetin alfa (europa.eu)	https://www.ema.europa.eu/en/documents/withdrawal-letter/withdrawal-letter-aranesp_en.pdf	**No orphan drugs**	24.04.2018
Qizenday,	unknown	Biotin	Medday Pharmaceuticals	Medday Pharmaceuticals	**Other medicines**	**A11HA05**	**08 Diseases of the nervous system**	ICD-11 Chapter 08	progressive multiple sclerosis	**Enzyme (Antagonism)(N1.A)**	**/N1.A<br>//**	Enzyme (Ligases, Activation) (N1.6.B)	**/N1.6.B<br>//**	**Initial authorisation**		11/13/17	• open questions from the EMA (a17)<br>• concerns regarding responses/information from the manufacturer (a1)<br>• doubts about efficacy<br>reason: lack of data to sufficiently demonstrate efficacy (a27.3)<br>• doubts about efficacy<br>reason: poor study quality (small sample size) (a27.1.5)<br>• doubts about assurance of patient safety (a21)<br>• open questions on bioavailability, bioequivalence, distribution and metabolism (for one or more topics) (a29)	**/A17<br>/A1<br>/A27.3<br>/A27.1.5<br>/A21.<br>/A29<br>//**	15/12/2017	**No authorisation, because of no positive benefit-risk ratio**	**EMA evaluation**	**Number of clinical studies 02**	**Participants 0200-0299**	**Placebo controlled clinical studies**		**No comparison with approved drugs**	**No special drug approval procedure**	**No consequences for patients in ongoing clinical trials or compassionate use programmes**	Ongoing clinical trials (at the time the application was withdrawn)	Qizenday_17-11_ withdrawal Q&A (europa.eu)	https://www.ema.europa.eu/en/documents/withdrawal-letter/withdrawal-letter-qizenday_en.pdf	**No orphan drugs**	2/6/18

Zydelig,	idelalisib	Idelalisib	Gilead Sciences Ireland UC	Gilead Sciences	**Kinase inhibitors**	**L01XX47**	**02 Neoplasms/**<br><br>**X Extension codes (Leukemias)**	ICD-11 Chapter 02<br><br>ICD-11 Chapter X	application in combination with rituximab and bendamustine in chronic lymphocytic leukemia (CLL) after at least one prior treatment<br><br>already approved indications:<br>• chronic lymphocytic leukemia (CLL) (in combination with rituximab or ofatumumab in patients who have received at least one previous treatment and in patients with genetic mutations such as 17p-deletion or TP53-mutation which cannot be treated with another therapy)<br>• follicular lymphoma (non-Hodgkin) (monotherapy if there is no response to two prior treatments)	**Enzyme (Antagonism)(N1.A)**	**/N1.A //**	Enzyme (Transferases, Antagonism)(N1.2.A)	**/N1.2.A //**	**Post-authorisation**	9/18/14	1/30/18	• open questions from the EMA (a17)<br>• concerns regarding responses/information from the manufacturer (a1)<br>• doubts about assurance of patient safety<br>reason: side effects (a21.1)<br>• doubts about assurance of patient safety<br>reason: lack of data and/or inadequate long-term follow-up (a21.3)<br>• doubts about efficacy<br>reason: flawed study design (study design not sufficient to conclusively evaluate data) (a27.2)	**/A17<br>/A1<br>/A21.1<br>/A21.3<br>/A27.2<br>//**	23/02/2018	**No authorisation, because of no positive benefit-risk ratio**	**EMA evaluation**	**Number of clinical studies 01**	**Participants 0300-0499**	**Placebo controlled clinical studies**		**Comparison with approved drugs**	**No special drug approval procedure**	**No consequences for patients in ongoing clinical trials or compassionate use programmes**	Ongoing clinical trials (at the time the application was withdrawn)	Zydelig, INN-idelalisib (europa.eu)	https://www.ema.europa.eu/en/documents/withdrawal-letter/withdrawal-letter-zydelig_en.pdf	**No orphan drugs**	2/23/18
Plivensia,	sirukumab	Sirukumab	Janssen-Cilag International NV	Janssen Pharmaceutical Companies of Johnson & Johnson	**Monoclonal antibodies**	**L04AC**	**15 Diseases of the musculoskeletal system or connective tissue**	ICD-11 Chapter 15	rheumatoid arthritis (moderate to severe)<br><br>Note:<br>Use if treatment with one or more disease-modifying antirheumatic drugs (DMARD) was not sufficiently effective or caused unpleasant side effects, use together with methotrexate or alone if methotrexate cannot be taken	**Ligands (antagonism)(N9.A)**	**/N9.A //**	Ligands (Cytokines, Antagonism)(N9.2.A)	**/N9.2.A //**	**Initial authorisation**		10/26/17	• open questions from the EMA (a17)<br>• concerns regarding responses/information from the manufacturer (a1)<br>• doubts about assurance of patient safety<br>reason: lack of data and/or inadequate long-term follow-up (a21.3)<br>• doubts about efficacy<br>reason: flawed study design (a27.2)	**/A17<br>/A1<br>/A21.3<br>/A27.2<br>//**	10/11/2017	**No authorisation, because of no positive benefit-risk ratio**	**Marketing strategy EMA evaluation**	**Number of clinical studies 03**	**Participants over 2000**	**Placebo controlled clinical studies**		**Comparison with approved drugs**	**No special drug approval procedure**	**Consequences for patients in ongoing clinical trials or compassionate use programmes**	Ongoing clinical trials (at the time the application was withdrawn)	Plivensia, INN-sirukumab (europa.eu)	https://www.ema.europa.eu/en/documents/withdrawal-letter/withdrawal-letter-plivensia_en.pdf	**No orphan drugs**	11/10/17
Kyomarc,	bevacizumab	Bevacizumab	Amgen Europe B.V.	Amgen Europe B.V.	**Monoclonal antibodies**	**L01XC07**	**02 Neoplasms**	ICD-11 Chapter 02	colon cancer, rectal cancer, breast cancer, non-small cell lung carcinoma (NSCLC), renal cell carcinoma, cervical cancer, ovarian cancer, tubal cancer, peritoneal carcinosis	**Ligands (antagonism)(N9.A)**	**/N9.A //**	Ligands (Vascular endothelial growth factor (VEGF), Antagonism) (N9.1.A)	**/N9.1.A //**	**Initial authorisation**		10/17/17	• not available (marketing authorisation was recommended) (a14.2)	**/A14.2<br>//**	10/11/2017	**Authorisation recommended**	**Marketing strategy**	**Number of clinical studies 01**	**Participants 0500-0999**	**No placebo control**	**SDB**	**Comparison with approved drugs**	**Drug application for biosimilars**	**No consequences for patients in ongoing clinical trials or compassionate use programmes**	No ongoing clinical trials (at the time the application was withdrawn)	Kyomarc_17-11_withdrawal Q-and-A (europa.eu)	https://www.ema.europa.eu/en/documents/withdrawal-letter/withdrawal-letter-kyomarc_en.pdf	**No orphan drugs**	11/10/17
Tigecycline Accord,	tigecycline	Tigecycline	Accord Healthcare Ltd	Accord Healthcare	**Antibacterial drugs**	**J01AA12**	**14 Skin diseases**	ICD-11 Chapter 14	infections of the skin and soft tissue (adults and children over eight years old, except foot infections in people with diabetes), complicated abdominal infections (belly)<br><br>Note:<br>Use only when other antibiotics are unsuitable	**other proteins (Antagonism/Binding) (N5.A)**	**/N5.A //**	other proteins (bacterial proteins / bacterial protein synthesis, Antagonism)(N5.9.A)	**/N5.9.A //**	**Initial authorisation**		9/12/17	• open questions from the EMA (a17)<br>• concerns regarding responses/information from the manufacturer (a1)<br>• concerns regarding medicinal product quality (manufacturing process, lack of compliance with gmp) (a3.1)	**/A17<br>/A1<br>/A3.1<br>//**	15/09/2017	**No authorisation, because of no positive benefit-risk ratio**	**Marketing strategy**	**Number of clinical studies 0**	**Participants 0**	**No placebo control**	**SDG**	**Comparison with approved drugs**	**Drug application for generic medicines**	**No consequences for patients in ongoing clinical trials or compassionate use programmes**	Ongoing clinical trials (at the time the application was withdrawn)	Tigecycline Accord, INN-tigecycline (europa.eu)	https://www.ema.europa.eu/en/documents/withdrawal-letter/withdrawal-letter-tigecycline-accord_en.pdf	**No orphan drugs**	10/10/17
Opdivo,	nivolumab	Nivolumab	Bristol-Myers Squibb Pharma EEIG	Bristol-Myers Squibb Pharma EEIG	**Monoclonal antibodies**	**L01FF01**	**02 Neoplasms**	ICD-11 Chapter 02	metastatic colorectal cancer (with "mismatch repair deficiency" or high "microsatellite instability", use in adults previously treated with fluoropyrimidines in combination with other cancer drugs)<br><br>already approved indications:<br>melanoma, Hodgkin lymphoma, renal cell carcinoma, non-small cell lung carcinoma, squamous cell carcinoma of the esophagus, squamous cell carcinoma of the head and neck (SCCHN), urothelial carcinoma, malignant pleural mesothelioma, colorectal carcinoma,	**other proteins (Antagonism/Binding) (N5.A)**	**/N5.A //**	other proteins (Immune checkpoint proteins/CD-molecules, Antagonism/Binding) (N5.4.A)	**/N5.4.A //**	**Post-authorisation**	6/19/15	12/13/17	• open questions from the EMA (a17)<br>• concerns regarding responses/information from the manufacturer (a1)<br>• doubts about efficacy<br>reason: lack of data to sufficiently demonstrate efficacy (a27.3)<br>• doubts about efficacy<br>reason: poor study quality (selection of participants) (a27.1.7)<br>• doubts about efficacy<br>reason: flawed study design (a27.2)<br>• doubts about efficacy<br>reason: poor study quality and/or flawed study design (lack of comparison with placebo treatment and/or reference medicinal products) (a27.1.4)	**/A17<br>/A1<br>/A27.3<br>/A27.1.4<br>/A27.2<br>/A27.1.7<br>//**	26/01/2018	**No extension of authorisation, because of no positive benefit-risk ratio**	**EMA evaluation**	**Number of clinical studies 01**	**Participants 0050-0074**	**No placebo control**		**No comparison with approved drugs**	**No special drug approval procedure**	**No consequences for patients in ongoing clinical trials or compassionate use programmes**	Ongoing clinical trials (at the time the application was withdrawn)	Withdrawal of the application for a change to the marketing authorisation for Opdivo (nivolumab) (europa.eu)	https://www.ema.europa.eu/en/documents/withdrawal-letter/withdrawal-letter-opdivo-ii-30_en.pdf	**No orphan drugs**	1/26/18
Opdivo,	nivolumab	Nivolumab	Bristol-Myers Squibb Pharma EEIG	Bristol-Myers Squibb Pharma EEIG	**Monoclonal antibodies**	**L01FF01**	**02 Neoplasms**	ICD-11 Chapter 02	hepatocellular carcinoma (use in adults previously treated with sorafenib)<br><br>already approved indications:<br>melanoma, Hodgkin lymphoma, renal cell carcinoma, non-small cell lung carcinoma, squamous cell carcinoma of the esophagus, squamous cell carcinoma of the head and neck (SCCHN), urothelial carcinoma, malignant pleural mesothelioma, colorectal carcinoma	**other proteins (Antagonism/Binding) (N5.A)**	**/N5.A //**	other proteins (Immune checkpoint proteins/CD-molecules, Antagonism/Binding) (N5.4.A)	**/N5.4.A //**	**Post-authorisation**	6/19/15	7/20/17	• open questions from the EMA (a17)<br>• concerns regarding responses/information from the manufacturer (a1)<br>• doubts about efficacy<br>reason: other reasons (very little information about patients in the study available) (a27)<br>• doubts about efficacy<br>reason: poor study quality and/or flawed study design (lack of comparison with placebo treatment and/or reference medicinal products) (a27.1.4)<br>• doubts about efficacy<br>reason: lack of data to sufficiently demonstrate efficacy (a27.3)	**/A17<br>/A1<br>/A27.1.4<br>/A27.3<br>/A27.<br>//**	15/09/2017	**No extension of authorisation, because of no positive benefit-risk ratio**	**EMA evaluation**	**Number of clinical studies 01**	**Participants 0100-0149**	**No placebo control**		**No comparison with approved drugs**	**No special drug approval procedure**	**No consequences for patients in ongoing clinical trials or compassionate use programmes**	Ongoing clinical trials (at the time the application was withdrawn)	Opdivo "INN nivoluman" (europa.eu)	https://www.ema.europa.eu/en/documents/withdrawal-letter/withdrawal-letter-opdivo_en.pdf-0	**No orphan drugs**	12/6/17
Ogivri,	trastuzumab	Trastuzumab	Mylan S.A.S	Mylan	**Monoclonal antibodies**	**L01FD01**	**02 Neoplasms**	ICD-11 Chapter 02	early and metastatic breast cancer, metastatic<br>gastric cancer (use only if cancer overexpresses HER2)	**Receptors (Antagonism) (N4.A)**	**/N4.A //**	Receptors (catalytic receptors, Antagonism) (N4.3.A)	**/N4.3.A //**	**Initial authorisation**		8/3/17	• open questions from the EMA (a17)<br>• concerns regarding responses/information from the manufacturer (a1)<br>• eu gmp (good manufacturing practice) certificate not available (a8)	**/A17<br>/A1<br>/A8<br>//**	15/09/2017	**No authorisation, because of no positive benefit-risk ratio**	**EMA evaluation**	**Number of clinical studies 01**	**Participants 0500-0999**	**No placebo control**	**SDB**	**Comparison with approved drugs**	**Drug application for biosimilars**	**No consequences for patients in ongoing clinical trials or compassionate use programmes**	Ongoing clinical trials (at the time the application was withdrawn)	Ogivri Withdrawal Q&A (europa.eu)	https://www.ema.europa.eu/en/documents/withdrawal-letter/withdrawal-letter-ogivri_en.pdf	**No orphan drugs**	11/28/17
Keytruda,	pembrolizumab	Pembrolizumab	Merck Sharp & Dohme B.V.	Merck Sharp & Dohme	**Monoclonal antibodies**	**L01FF02**	**02 Neoplasms**	ICD-11 Chapter 02	non-small cell lung carcinoma (NSCLC)<br><br>(use if NSCLC is metastatic, without squamous cell histology, in combination with pemetrexed and carboplatin, regardless of whether the tumor produces the PD-L1 protein)<br><br>already approved indications:<br>melanoma, non-small cell lung carcinoma, classical Hodgkin lymphoma, urothelial carcinoma, head and neck squamous cell carcinoma, renal cell carcinoma,<br>specific cancers when microsatellite instability is high (MSI-H) or mismatch repair deficiency (colon cancer, endometrial cancer, gastric cancer, small bowel cancer, bile duct cancer)<br>triple-negative breast cancer,<br>gastric cancer,<br>esophageal cancer, endometrial cancer,<br>cervical cancer	**other proteins (Antagonism/Binding) (N5.A)**	**/N5.A //**	other proteins (Immune checkpoint proteins/CD-molecules, Antagonism/Binding) (N5.4.A)	**/N5.4.A //**	**Post-authorisation**	7/17/15	10/11/17	• open questions from the EMA (a17)<br>• concerns regarding responses/information from the manufacturer (a1)<br>• doubts about efficacy<br>reason: lack of data to sufficiently demonstrate efficacy (a27.3)<br>• doubts about assurance of patient safety (a21)<br>• lack of data to sufficiently demonstrate efficacy (data from not-yet-completed studies necessary to assess benefit and risk) (a12)	**/A17<br>/A1<br>/A27.3<br>/A21.<br>/A12<br>//**	10/11/2017	**No extension of authorisation, because of no positive benefit-risk ratio**	**EMA evaluation**	**Number of clinical studies 01**	**Participants 0100-0149**	**No placebo control**		**Comparison with approved drugs**	**No special drug approval procedure**	**No consequences for patients in ongoing clinical trials or compassionate use programmes**	Ongoing clinical trials (at the time the application was withdrawn)	Keytruda, INN - pembrolizumab (europa.eu)	https://www.ema.europa.eu/en/documents/withdrawal-letter/withdrawal-letter-keytruda_en.pdf	**No orphan drugs**	11/28/17
Fulphila,	pegfilgrastim	Pegfilgrastim	Mylan S.A.S	Mylan	**Immunomodulators**	**L03AA13**	**04 Diseases of the immune system**	ICD-11 Chapter 04	neutropenia,<br>reduction in the occurrence of febrile neutropenia (in connection with cancer therapies)	**Receptors (Agonism) (N4.B)**	**/N4.B //**	Receptors (catalytic receptors, Agonism) (N4.3.B)	**/N4.3.B //**	**Initial authorisation**		8/3/17	• open questions from the EMA (a17)<br>• concerns regarding responses/information from the manufacturer (a1)<br>• concerns regarding medicinal product quality (manufacturing process) (a3.1)<br>• concerns regarding medicinal product quality (contaminants and/or concerns regarding sterilisation) (a3.3)<br>• eu gmp (good manufacturing practice) certificate not available (a8)	**/A17<br>/A1<br>/A3.1<br>/A3.3<br>/A8<br>//**	15/09/2017	**No authorisation, because of no positive benefit-risk ratio**	**EMA evaluation**	**Number of clinical studies 01**	**Participants 0150-0199**	**No placebo control**	**SDB**	**Comparison with approved drugs**	**Drug application for biosimilars**	**No consequences for patients in ongoing clinical trials or compassionate use programmes**	Ongoing clinical trials (at the time the application was withdrawn)	Fulphila, INN - pegfilgrastim (europa.eu)	https://www.ema.europa.eu/en/documents/withdrawal-letter/withdrawal-letter-fulphila_en.pdf	**No orphan drugs**	5/29/18
Infinia,	alpha-1-antitrypsin	Alpha-1-Antitrypsin	Kamada BioPharma Limited at Fieldfisher LLP	Kamada BioPharma Limited at Fieldfisher LLP	**Other medicines**	**B02AB02**	**05 Endocrine diseases, nutritional or metabolic diseases**<br><br>**12 Respiratory diseases**	ICD-11 Chapter 05<br><br>ICD-11 Chapter 12	lung damage due to inherited (hereditary)<br>alpha-1-antitrypsin deficiency (e.g., emphysema)	**Enzyme (Antagonism)(N1.A)**	**/N1.A //**	Enzyme (Hydrolases, Antagonism)(N1.3.A)	**/N1.3.A //**	**Initial authorisation**		6/21/17	• open questions from the EMA (a17)<br>• concerns regarding responses/information from the manufacturer (a1)<br>• doubts about efficacy<br>reason: lack of data to sufficiently demonstrate efficacy (a27.3)<br>• doubts about assurance of patient safety<br>reason: side effects (a21.1)	**/A17<br>/A1<br>/A21.1<br>/A27.3<br>//**	21/07/2017	**No authorisation, because of no positive benefit-risk ratio**	**EMA evaluation**	**Number of clinical studies 01**	**Participants 0150-0199**	**Placebo controlled clinical studies**		**No comparison with approved drugs**	**No special drug approval procedure**	**No consequences for patients in ongoing clinical trials or compassionate use programmes**	Ongoing clinical trials (at the time the application was withdrawn)	Withdrawal for Infinia-Q&A (europa.eu)	https://www.ema.europa.eu/en/documents/withdrawal-letter/withdrawal-letter-infinia_en.pdf	**Orphan drugs**	2/16/18

Zafiride,	unknown	NGR-human tumor necrosis factor alpha	MolMed SpA	MolMed SpA	Other medicines	L01X	02 Neoplasms	ICD-11 Chapter 02	advanced malignant pleural mesothelioma (use in adults with cancer that worsens within 6 months after treatment with cancer drugs (including pemetrexed).	Enzyme (N1.U)<br><br>Receptors (Agonism) (N4.B)	/N1.U<br>/N4.B<br>//	Enzyme (Hydrolases)(N1.3)<br><br>Receptors (catalytic receptors, Agonism) (N4.3.B)	/N1.3.U<br>/N4.3.B<br>//	Initial authorisation		6/1/17	• open questions from the EMA (a17)<br>• concerns regarding responses/information from the manufacturer (a1)<br>• concerns regarding medicinal product quality (manufacturing process) (a3.1)<br>• doubts about assurance of patient safety<br>reason: lack of data and/or inadequate long-term follow-up (a21.3)<br>• doubts about efficacy<br>reason: lack of data to sufficiently demonstrate efficacy (a27.3)	/A17<br>/A1<br>/A3.1<br>/A21.3<br>/A27.3<br>//	23/06/2017	No authorisation, because of no positive benefit-risk ratio	EMA evaluation	Number of clinical studies 01	Participants 0300-0499	Placebo controlled clinical studies		Comparison with approved drugs	No special drug approval procedure	No consequences for patients in ongoing clinical trials or compassionate use programmes	Ongoing clinical trials (at the time the application was withdrawn)	Zafiride_Withdrawal Q&A (europa.eu)	https://www.ema.europa.eu/en/documents/withdrawal-letter/withdrawal-letter-zafiride_en.pdf	Orphan drugs	2/8/18
Elmisol,	levamisole	Levamisole Hydrochloride	ACE Pharmaceuticals BV	ACE Pharmaceuticals BV	Other medicines	P02CE01	16 Diseases of the urogenital system	ICD-11 Chapter 16	nephrotic syndrome<br><br>Note:<br>Use in recurring symptoms after initial treatment with corticosteroids (steroid-sensitive nephrotic syndrome)	other (N6)<br><br>Receptors (Agonism) (N4.B)	/N4.B<br>/N6.U<br>//	other (N6)<br><br><br>Receptors (Ligand-gated ion channels, Agonism) (N4.2.B)	/N6.U<br>/N4.2.B<br>//	Initial authorisation		5/29/17	• open questions from the EMA (a17)<br>• concerns regarding responses/information from the manufacturer (a1)<br>• doubts about compliance with good clinical practice (gcp) (a30)<br>• open questions on bioavailability, bioequivalence, distribution and metabolism (for one or more topics) (a29)<br>• doubts about efficacy<br>reason: poor study quality (conduct) (a27.1.6)<br>• concerns regarding medicinal product quality (stability of the active substance in tablets) (a3)<br>• lack of data to sufficiently demonstrate efficacy (a12)<br>• doubts about assurance of patient safety (dosing errors due to risk of confusion of tablets) (a21)<br>• doubts about assurance of patient safety<br>reason: interactions with other medicinal products (a21.2)	/A17<br>/A1<br>/A30<br>/A29<br>/A27.1.6<br>/A3.<br>/A12<br>/A21.<br>/A21.2<br>//	23/06/2017	No authorisation, because of no positive benefit-risk ratio	EMA evaluation	Number of clinical studies 01	Participants 0100-0149	Placebo controlled clinical studies	SD	No comparison with approved drugs	No special drug approval procedure	No consequences for patients in ongoing clinical trials or compassionate use programmes	Ongoing clinical trials (at the time the application was withdrawn)	Elmisol, INN-levamisole (europa.eu)	https://www.ema.europa.eu/en/documents/withdrawal-letter/withdrawal-letter-elmisol_en.pdf	Orphan drugs	7/18/17
Qinprezo,	unknown	Vosaroxin	Sunesis Europe Ltd	Sunesis Europe Ltd	Cytostatics	L01	X Extension codes (Leukemias) /<br><br>02 Neoplasms	ICD-11 Chapter 02<br><br>ICD-11 Chapter X	acute myeloid leukemia (AML)<br><br>Note:<br>Use in combination with cytarabine in refractory or recurrent AML from age 60	Enzyme (Antagonism)(N1.A)	/N1.A<br>//	Enzyme (Isomerases, Antagonism) (N1.5.A)	/N1.5.A<br>//	Initial authorisation		5/10/17	• open questions from the EMA (a17)<br>• concerns regarding responses/information from the manufacturer (a1)<br>• doubts about assurance of patient safety<br>reason: side effects (infections) (a21.1)<br>• doubts about efficacy<br>reason: lack of data to sufficiently demonstrate efficacy (especially in patients over 60 years no benefit regarding overall survival) (a27.3)	/A17<br>/A1<br>/A21.1<br>/A27.3<br>//	19/05/2017	No authorisation, because of no positive benefit-risk ratio	Suspected EMA evaluation Marketing strategy	Number of clinical studies 01	Participants 0500-0999	No placebo control		Comparison with approved drugs	No special drug approval procedure	No consequences for patients in ongoing clinical trials or compassionate use programmes	Ongoing clinical trials (at the time the application was withdrawn)	Qinprezo_withdrawal Q&A (europa.eu)	https://www.ema.europa.eu/en/documents/withdrawal-letter/withdrawal-letter-qinprezo_en.pdf	No orphan drugs	8/15/17
Solithromycin Triskel EU Services,	solithromycin	Solithromycin	Triskel EU Services Ltd	Triskel EU Services Ltd	Antibacterial drugs	J01FA	12 Respiratory diseases/<br><br>01 Certain infectious or parasitic diseases	ICD-11 Chapter 12<br><br>ICD-11 Chapter 01	community-acquired pneumonia, inhalational anthrax, inhaled tularemia	other proteins (Antagonism/Binding) (N5.A)	/N5.A<br>//	other proteins (bacterial proteins / bacterial protein synthesis, Antagonism)(N5.9.A)	/N5.9.A<br>//	Initial authorisation		3/27/17	• open questions from the EMA (a17)<br>• concerns regarding responses/information from the manufacturer (a1)<br>• doubts about assurance of patient safety<br>reason: lack of data and/or inadequate long-term follow-up (related to inhaled anthrax and inhaled tularemia) (a21.3)<br>• doubts about assurance of patient safety<br>reason: side effects (liver damage) (a21.1)<br>• concerns regarding medicinal product quality (contaminants and/or concerns regarding sterilisation) (a3.3)<br>• concerns regarding medicinal product quality (manufacturing process) (a3.1)	/A17<br>/A1<br>/A21.3<br>/A3.3<br>/A21.1<br>/A3.1<br>//	21/04/2017	No authorisation, because of no positive benefit-risk ratio	Marketing strategy	Number of clinical studies 03	Participants 1000-1999	No placebo control	SD	Comparison with approved drugs	No special drug approval procedure	No consequences for patients in ongoing clinical trials or compassionate use programmes	Ongoing clinical trials (at the time the application was withdrawn)	Solithromycin Triskel EU Services, INN-solithromycin (europa.eu)	https://www.ema.europa.eu/en/documents/withdrawal-letter/withdrawal-letter-solithromycin-triskel-eu-services_en.pdf	No orphan drugs	6/2/17
Blectifor,	caffeine citrate	Caffeine Citrate	Viridian Pharma Ltd	Viridian Pharma Ltd	Other medicines	R07AB	19 Certain diseases originating in the perinatal period	ICD-11 Chapter 19	bronchopulmonary dysplasia (prevention of bronchopulmonary dysplasia in prolonged mechanical ventilation of premature infants)	Receptors (Antagonism) (N4.A)	/N4.A<br>//	Receptors (GPCR, Antagonism) (N4.1.A)	/N4.1.A<br>//	Initial authorisation		3/9/17	• open questions from the EMA (a17)<br>• concerns regarding responses/information from the manufacturer (a1)<br>• concerns regarding the requested indication (a28) (target population not sufficiently described)<br>• general medical use (systEMAtic and documented use in the eu for at least 10 years) for the requested indication not demonstrated (a32)<br>• eu gmp (good manufacturing practice) certificate not available (a8)<br>• lack of data to sufficiently demonstrate efficacy (a12)	/A17<br>/A1<br>/A28<br>/A32<br>/A8<br>/A12<br>//	24/03/2017	No authorisation, because of no positive benefit-risk ratio	Marketing strategy	Number of clinical studies 0	Participants 0	No placebo control	SD	No comparison with approved drugs	No special drug approval procedure	No consequences for patients in ongoing clinical trials or compassionate use programmes	No ongoing clinical trials (at the time the application was withdrawn)	Blectifor, INN-caffeine citrate (europa.eu)	https://www.ema.europa.eu/en/documents/withdrawal-letter/withdrawal-letter-blectifor_en.pdf	Orphan drugs	5/16/17
Enpaxiq,	pacritinib	Pacritinib	CTI Life Sciences Limited	CTI Life Sciences Limited	Kinase inhibitors	L01XE	02 Neoplasms /<br><br>X Extension codes (Chronic myeloproliferative diseases, malignant)	ICD-11 Chapter 02<br><br>ICD-11 Chapter X	myelofibrosis (enlarged spleen or other symptoms of myelofibrosis)	Enzyme (Antagonism)(N1.A)<br><br>Receptors (Antagonism)(N4.A)	/N1.A<br>/N4.A<br>//	Enzyme (Transferases, Antagonism)(N1.2.A)<br><br><br>Receptors (catalytic receptors, Antagonism)(N4.3.A)	/N1.2.A<br>/N4.3.A<br>//	Initial authorisation		2/20/17	• open questions from the EMA (a17)<br>• concerns regarding responses/information from the manufacturer (a1)<br>• doubts about efficacy<br>reason: lack of data to sufficiently demonstrate efficacy (a27.3)<br>• doubts about assurance of patient safety<br>reason: side effects (incidence of low platelets higher -> increased bleeding risk) (a21.1)<br>• concerns regarding medicinal product quality (manufacturing process) (a3.1)<br>• increased number of deaths compared with the best available therapy (a31)<br>• doubts about assurance of patient safety (open questions on effect on certain target proteins in the body) (a21)	/A17<br>/A1<br>/A27.3<br>/A21.1<br>/A3.1<br>/A31<br>/A21.<br>//	24/03/2017	No authorisation, because of no positive benefit-risk ratio	EMA evaluation	Number of clinical studies 01	Participants 0300-0499	No placebo control		Comparison with approved drugs	No special drug approval procedure	No consequences for patients in ongoing clinical trials or compassionate use programmes	Ongoing clinical trials (at the time the application was withdrawn)	Enpaxiq Withdrawal Q&A (europa.eu)	https://www.ema.europa.eu/en/documents/withdrawal-letter/withdrawal-letter-enpaxiq_en.pdf	Orphan drugs	6/9/17
Zioxtenzo,	pegfilgrastim	Pegfilgrastim	Sandoz GmbH	Sandoz GmbH	Immunomodulator s	L03AA13	04 Diseases of the immune system	ICD-11 Chapter 04	neutropenia,<br>reduction in the occurrence of febrile neutropenia (in connection with cancer therapies)	Receptors (Agonism) (N4.B)	/N4.B<br>//	Receptors (catalytic receptors, Agonism) (N4.3.B)	/N4.3.B<br>//	Initial authorisation		1/18/17	• open questions from the EMA (a17)<br>• concerns regarding responses/information from the manufacturer (a1)<br>• eu gmp (good manufacturing practice) certificate not available (a8)<br>• open questions on bioavailability, bioequivalence, distribution and metabolism (for one or more topics) (a29)<br>• doubts about same/improved efficacy compared with the eu reference medicinal product (a25)	/A17<br>/A1<br>/A8<br>/A29<br>/A25<br>//	27/01/2017	No authorisation, because of no positive benefit-risk ratio	EMA evaluation	Number of clinical studies 02	Participants 0500-0999	No placebo control	SDB	Comparison with approved drugs	Drug application for biosimilars	No consequences for patients in ongoing clinical trials or compassionate use programmes	Ongoing clinical trials (at the time the application was withdrawn)	Zioxtenzo, INN-pegfilgrastim (europa.eu)	https://www.ema.europa.eu/en/documents/withdrawal-letter/withdrawal-letter-zioxtenzo_en.pdf	No orphan drugs	4/25/17
Cavoley,	pegfilgrastim	Pegfilgrastim	STADA Arzneimittel AG	STADA Arzneimittel AG	Immunomodulator s	L03AA13	04 Diseases of the immune system	ICD-11 Chapter 04	neutropenia,<br>reduction in the occurrence of febrile neutropenia (in connection with cancer therapies)	Receptors (Agonism) (N4.B)	/N4.B<br>//	Receptors (catalytic receptors, Agonism) (N4.3.B)	/N4.3.B<br>//	Initial authorisation		11/16/16	• open questions from the EMA (a17)<br>• concerns regarding responses/information from the manufacturer (a1)<br>• doubts about same/improved efficacy compared with the eu reference medicinal product (very similar metabolism compared with the reference medicinal product could not be demonstrated) (a25)	/A17<br>/A1<br>/A25<br>//	16/12/2016	No authorisation, because of no positive benefit-risk ratio	EMA evaluation	Number of clinical studies 01	Participants 0200-0299	No placebo control	SDB	Comparison with approved drugs	Drug application for biosimilars	No consequences for patients in ongoing clinical trials or compassionate use programmes	No ongoing clinical trials (at the time the application was withdrawn)	Cavoley, INN-pegfilgrastim (europa.eu)	https://www.ema.europa.eu/en/documents/withdrawal-letter/withdrawal-letter-cavoley_en.pdf	No orphan drugs	6/27/17
Xgeva,	denosumab	Denosumab	Amgen Europe B.V.	Amgen Europe B.V.	Monoclonal antibodies	M05BX04	05 Endocrine diseases, nutritional or metabolic diseases/<br><br>02 Neoplasms	ICD-11 Chapter 05<br><br>ICD-11 Chapter 02	malignant hypercalcemia (as a result of cancer)<br><br>Note:<br>Use when there is insufficient response to treatment with bisphosphonates<br><br>already approved indications:<br>prevention of bone complications in adults with advanced cancer (metastatic to bone), treatment of giant cell tumors in mature bone (adults and adolescents) when surgery is not possible or too risky	Ligands (antagonism)(N9.A)	/N9.A<br>//	Ligands (RANK-Ligand, Antagonism)(N9.A)	/N9.A<br>//	Post-authorisation	7/13/11	1/13/17	• open questions from the EMA (a17)<br>• concerns regarding responses/information from the manufacturer (a1)<br>• doubts about efficacy<br>reason: poor study quality and/or flawed study design (lack of comparison with placebo treatment and/or comparator products/reference medicinal products) (a27.1.4)<br>• doubts about efficacy<br>reason: poor study quality (small sample size) (a27.1.5)<br>• doubts about efficacy<br>reason: poor study quality (conduct) (a27.1.6)<br>• doubts about efficacy<br>reason: lack of data to sufficiently demonstrate efficacy (a27.3)<br>• doubts about efficacy<br>reason: flawed study design (a27.2)	/A17<br>/A1<br>/A27.1.4<br>/A27.1.5<br>/A27.3<br>/A27.1.6<br>/A27.2<br>//	27/01/2017	No extension of authorisation, because of no positive benefit-risk ratio	EMA evaluation	Number of clinical studies 01	Participants 0025-0049	No placebo control		No comparison with approved drugs	No special drug approval procedure	No consequences for patients in ongoing clinical trials or compassionate use programmes	No ongoing clinical trials (at the time the application was withdrawn)	Xgeva, INN-denosumab (europa.eu)	https://www.ema.europa.eu/en/documents/withdrawal-letter/withdrawal-letter-xgeva_en.pdf	No orphan drugs	5/5/17
Efgratin,	pegfilgrastim	Pegfilgrastim	Gedeon Richter Plc.	Gedeon Richter Plc.	Immunomodulator s	L03AA13	04 Diseases of the immune system	ICD-11 Chapter 04	neutropenia,<br>reduction in the occurrence of febrile neutropenia (in connection with cancer therapies)	Receptors (Agonism) (N4.B)	/N4.B<br>//	Receptors (catalytic receptors, Agonism) (N4.3.B)	/N4.3.B<br>//	Initial authorisation		11/16/16	• open questions from the EMA (a17)<br>• concerns regarding responses/information from the manufacturer (a1)<br>• doubts about same/improved efficacy compared with the eu reference medicinal product (very similar metabolism compared with the reference medicinal product could not be demonstrated) (a25)	/A17<br>/A1<br>/A25<br>//	16/12/2016	No authorisation, because of no positive benefit-risk ratio	EMA evaluation	Number of clinical studies 01	Participants 0200-0299	No placebo control	SDB	Comparison with approved drugs	Drug application for biosimilars	No consequences for patients in ongoing clinical trials or compassionate use programmes	No ongoing clinical trials (at the time the application was withdrawn)	Efgratin, INN-pegfilgrastim (europa.eu)	https://www.ema.europa.eu/en/documents/withdrawal-letter/withdrawal-letter-efgratin_en.pdf	No orphan drugs	6/27/17

Graspa,	eryaspase	Eryaspase<br><br>(Version of the enzyme Asparaginase)	ERYTECH Pharma S.A.	ERYTECH Pharma S.A.	**Cytostatics**	**L01**	**X Extension codes (Leukemias) /**<br><br>**02 Neoplasms**	ICD-11 Chapter 02<br><br>ICD-11 Chapter X	acute lymphoblastic leukemia (ALL)<br><br>Note:<br>Use in cases of insufficient efficacy of first-line treatment or relapsed ALL (in "Philadelphia chromosome-negative" adults and children over one year) in combination with other cancer drugs.	**Ligands (antagonism)(N9.A)**	**/N9.A<br>//**	Ligands (Asparagine, Antagonism (degradation)) (N9.A)	**/N9.A<br>//**	**Initial authorisation**		11/14/16	• open questions from the EMA (a17)<br>• concerns regarding the applicant's answers/information (a1)<br>• doubts about efficacy<br>reason: poor study quality (conduct) (a27.1.6)<br>• doubts about efficacy<br>reason: poor study quality (selection of participants, concerns about extrapolating study results to other drug combinations in all therapy) (a27.1.7)<br>• concerns regarding medicinal product quality (manufacturing process, change in manufacturing) (a3.1)	**/A17<br>/A1<br>/A27.1.6<br>/A27.1.7<br>/A3.1<br>//**	16/12/2016	**No authorisation, because of no positive benefit-risk ratio**	**EMA evaluation**	**Number of clinical studies 01**	**Participants 0075-0099**	**No placebo control**	**SD**	**Comparison with approved drugs**	**No special drug approval procedure**	**No consequences for patients in ongoing clinical trials or compassionate use programmes**	Ongoing clinical trials (at the time the application was withdrawn)	Graspa, INN-eryaspase (europa.eu)	https://www.ema.europa.eu/en/documents/withdrawal-letter/withdrawal-letter-graspa_en.pdf-0	**Orphan drugs**	4/25/17
Translarna,	ataluren	Ataluren	PTC Therapeutics International Limited	PTC Therapeutics International Limited	**Other medicines**	**M09AX03**	**12 Respiratory diseases**	ICD-11 Chapter 12	cystic fibrosis (use in patients with nonsense mutation in CFTR gene)<br><br>already approved indications: duchenne muscular dystrophy<br><br>Note: use in ambulatory patients with "nonsense mutation" in dystrophin gene, from age 5.	**other (N6)**	**/N6.U<br>//**	other (N6)	**/N6.U<br>//**	**Post-authorisation**	7/31/14	3/6/17	• open questions from the EMA (a17)<br>• concerns regarding the applicant's answers/information (a1)<br>• doubts about efficacy<br>reason: lack of data to sufficiently demonstrate efficacy (a27.3)<br>• lack of data to sufficiently demonstrate efficacy (a12)<br>• doubts about assurance of patient safety<br>reason: adverse effects (interaction with aminoglycosides and vancomycin) (a21.1)	**/A17<br>/A1<br>/A27.3<br>/A12<br>/A21.1<br>//**	24/03/2017	**No extension of authorisation, because of no positive benefit-risk ratio**	**EMA evaluation**	**Number of clinical studies 01**	**Participants 0200-0299**	**Placebo controlled clinical studies**		**No comparison with approved drugs**	**No special drug approval procedure**	**Consequences for patients in ongoing clinical trials or compassionate use programmes**	Ongoing clinical trials (at the time the application was withdrawn)	Translarna_17-03_ext of ind withdrawal Q&A (europa.eu)	https://www.ema.europa.eu/en/documents/withdrawal-letter/withdrawal-letter-translarna_en.pdf	**Orphan drugs**	3/24/17
Kepnetic,	unknown	N-Acetylneuraminic acid	Ultragenyx UK Limited	Ultragenyx UK Limited	**Other medicines**	**M09AX**	**04 Diseases of the immune system**	ICD-11 Chapter 04	GNE myopathy (previously known as hereditary inclusion body myopathy)	**other (N6)**	**/N6.U<br>//**	other (N6)	**/N6.U<br>//**	**Initial authorisation**		11/10/16	• open questions from the EMA (a17)<br>• concerns regarding the applicant's answers/information (a1)<br>• doubts about efficacy<br>reason: inadequate study design (a27.2)<br>• doubts about efficacy<br>reason: lack of data to sufficiently demonstrate efficacy (a27.3)	**/A17<br>/A1<br>/A27.2<br>/A27.3<br>//**	16/12/2016	**No authorisation, because of no positive benefit-risk ratio**	**EMA evaluation**	**Number of clinical studies 01**	**Participants 0025-0049**	**Placebo controlled clinical studies**		**No comparison with approved drugs**	**No special drug approval procedure**	**No consequences for patients in ongoing clinical trials or compassionate use programmes**	Ongoing clinical trials (at the time the application was withdrawn)	Withdrawal of Kepnetic, aceneuramic acid, 500mg, prolonged release tablet (europa.eu)	https://www.ema.europa.eu/en/documents/withdrawal-letter/withdrawal-letter-kepnetic_en.pdf	**Orphan drugs**	12/16/16
Pemetrexed ditromethamine Hospira,	pemetrexed	Pemetrexed Ditromethamine	Hospira UK Limited	Hospira UK Limited	**Cytostatics**	**L01BA04**	**02 Neoplasms**	ICD-11 Chapter 02	malignant pleural mesothelioma, advanced non-small cell lung carcinoma (non-squamous epithelial)	**Enzyme (Antagonism)(N1.A)**	**/N1.A<br>//**	Enzyme (Transferases, Antagonism)(N1.2.A)<br><br>Enzyme (Oxidoreductases, Antagonism)(N1.1.A)	**/N1.2.A<br>/N1.1.A<br>//**	**Initial authorisation**		9/22/16	• open questions from the EMA (questions on manufacturing, storage, safety and efficacy, which according to the EMA should be clarified after approval) (a17)	**/A17<br>//**	14/10/2016	**Authorisation recommended**	**Marketing strategy**	**Number of clinical studies 0**	**Participants 0**	**No placebo control**	**SDG**	**Comparison with approved drugs**	**Drug application for generic medicines**	**No consequences for patients in ongoing clinical trials or compassionate use programmes**	No ongoing clinical trials (at the time the application was withdrawn)	Pemetrexed ditromethamine Hospira_16-10_withdrawal Q&A (europa.eu)	https://www.ema.europa.eu/en/documents/withdrawal-letter/withdrawal-letter-pemetrexed-ditromethamine-hospira_en.pdf	**No orphan drugs**	12/21/16
Zemfirza,	cediranib	Cediranib Maleate	AstraZeneca AB	AstraZeneca	**Kinase inhibitors**	**L01XE32**	**02 Neoplasms**	ICD-11 Chapter 02	ovarian cancer, tubal cancer, peritoneal carcinosis<br><br>Note:<br>Use in each case for relapses	**Receptors (Antagonism) (N4.A)**	**/N4.A<br>//**	Receptors (catalytic receptors, Antagonism) (N4.3.A)	**/N4.3.A<br>//**	**Initial authorisation**		9/19/16	• open questions from the EMA (a17)<br>• concerns regarding the applicant's answers/information (a1)<br>• doubts about compliance with good clinical practice (gcp) (a30)<br>• doubts about benefit (moderate improvement in progression-free survival) (a18)<br>• doubts about assurance of patient safety<br>reason: adverse effects (diarrhea, fatigue) (a21.1)	**/A17<br>/A1<br>/A30<br>/A18<br>/A21.1<br>//**	14/10/2016	**No authorisation, because of no positive benefit-risk ratio**	**EMA evaluation**	**Number of clinical studies 01**	**Participants 0300-0499**	**Placebo controlled clinical studies**		**Comparison with approved drugs**	**No special drug approval procedure**	**No consequences for patients in ongoing clinical trials or compassionate use programmes**	Ongoing clinical trials (at the time the application was withdrawn)	Zemfirza, INN-cediranib (europa.eu)	https://www.ema.europa.eu/en/documents/withdrawal-letter/withdrawal-letter-zemfirza_en.pdf	**Orphan drugs**	12/21/16
Arzerra,	ofatumumab	Ofatumumab	Novartis Europharm Limited	Novartis	**Monoclonal antibodies**	**L01XC10**	**02 Neoplasms/**<br><br>**X Extension codes (Leukemias)**	ICD-11 Chapter 02<br><br>ICD-11 Chapter X	recurrent chronic lymphocytic leukemia (CLL) (use in combination with bendamustine)<br><br>already approved indications (since 28.02.2019 no longer approved):<br>- chronic lymphocytic leukemia (CLL) in untreated patients who cannot be treated with fludarabine (then treatment together with chlorambucil or bendamustine)<br>- chronic lymphocytic leukemia (CLL), if patients do not respond to treatment with fludarabine and alemtuzumab (refractory)<br>- in relapsed chronic lymphocytic leukemia (CLL), treatment together with fludarabine and cyclophosphamide	**other proteins (N5.U)**	**/N5.U<br>//**	other proteins (CD molecules) (N5.1)	**/N5.1.U<br>//**	**Post-authorisation**	19.04.2010<br><br>**Bemerkung:**<br>Arzneimittel auf Wunsch des Herstellers seit dem 28.02.2019 nicht mehr in der EU zugelassen.	11/8/16	• open questions from the EMA (a17)<br>• concerns regarding the applicant's answers/information (a1)<br>• doubts about efficacy<br>reason: poor study quality or inadequate study design (no comparison with placebo and/or comparator/reference medicinal products) (a27.1.4)<br>• doubts about efficacy<br>reason: poor study quality (small number of participants) (a27.1.5)<br>• doubts about efficacy<br>reason: other reasons (study results were considered unreliable) (a27)	**/A17<br>/A1<br>/A27.1.4<br>/A27.1.5<br>/A27.<br>//**	16/12/2016	**No extension of authorisation, because of no positive benefit-risk ratio**	**EMA evaluation**	**Number of clinical studies 01**	**Participants 0050-0074**	**No placebo control**		**No comparison with approved drugs**	**No special drug approval procedure**	**No consequences for patients in ongoing clinical trials or compassionate use programmes**	Ongoing clinical trials (at the time the application was withdrawn)	Arzerra, INN-ofatumumab (europa.eu)	https://www.ema.europa.eu/en/documents/withdrawal-letter/withdrawal-letter-arzerra_en.pdf	**Orphan drugs**	12/16/16
Ertapenem Hospira,	ertapenem	Ertapenem Sodium	Hospira UK Limited	Hospira UK Limited	**Antibacterial drugs**	**J01DH03**	**12 Respiratory diseases/**<br><br>**14 Skin diseases/**<br><br>**13 Diseases of the digestive system /**<br><br>**01 Certain infectious or parasitic diseases/**<br><br>**16 Diseases of the urogenital system**	ICD-11 Chapter 12<br><br>ICD-11 Chapter 14<br><br>ICD-11 Chapter 13<br><br>ICD-11 Chapter 01<br><br>ICD-11 Chapter 16	bacterial infections (abdominal infections, community-acquired pneumonia, gynecological infections, foot infections in diabetes patients, prevention of infections at surgical sites during colorectal procedures)	**other proteins (Antagonism/Binding) (N5.A)**	**/N5.A<br>//**	other proteins (bacterial proteins / bacterial protein synthesis, Antagonism)(N5.9.A)	**/N5.9.A<br>//**	**Initial authorisation**		9/13/16	• open questions from the EMA (a17)<br>• concerns regarding the applicant's answers/information (a1)<br>• concerns regarding medicinal product quality (manufacturing process) (a3.1)<br>• concerns regarding medicinal product quality (shelf-life, starting materials) (a3)<br>• concerns regarding medicinal product quality (contaminants and/or concerns about sterilization) (a3.3)	**/A17<br>/A1<br>/A3.1<br>/A3.3<br>/A3.<br>//**	14/10/2016	**No authorisation, because of no positive benefit-risk ratio**	**Marketing strategy**	**Number of clinical studies 0**	**Participants 0**	**No placebo control**	**SDG**	**Comparison with approved drugs**	**Drug application for generic medicines**	**Consequences for patients in ongoing clinical trials or compassionate use programmes unknown**	Ongoing clinical trials unknown (at the time the application was withdrawn)	Ertapenem Hospira, INN-ertapenem (europa.eu)	https://www.ema.europa.eu/en/documents/withdrawal-letter/withdrawal-letter-ertapenem-hospira_en.pdf	**No orphan drugs**	12/6/16
Cokiera,	dasabuvir /ombitasvir/paritaprevir/ritonavir	Dasabuvir, Ombitasvir, Paritaprevir, Ritonavir	AbbVie Ltd.	AbbVie Ltd.	**Antivirals**	**J05AX66**	**01 Certain infectious or parasitic diseases**	ICD-11 Chapter 01	chronic hepatitis C	**Enzyme (Antagonism)(N1.A)**<br><br>**other proteins (Antagonism)(N5.A)**	**/N1.A<br>/N5.A<br>//**	other proteins (N5.A)<br><br>Enzyme (Oxidoreductases, Antagonism)(N1.1.A)<br><br>Enzyme (Hydrolases, Antagonism)(N1.3.A)<br><br>Enzyme (Transferases, Antagonism)(N1.2.A)	**/N5.A<br>/N1.1.A<br>/N1.3.A<br>/N1.2.A<br>//**	**Initial authorisation**		8/3/16	• open questions from the EMA (a17)<br>• concerns regarding the applicant's answers/information (a1)<br>• open questions on bioavailability, bioequivalence, distribution and metabolism (for one or more topics) — concerns particularly regarding absorption (a29)<br>• doubts about efficacy<br>reason: other reasons (concerns about efficacy particularly due to concerns about absorption) (a27)	**/A17<br>/A1<br>/A29<br>/A27.<br>//**	16/09/2016	**No authorisation, because of no positive benefit-risk ratio**	**Marketing strategy**	**Number of clinical studies 0**	**Participants 0**	**No placebo control**	**SD**	**Comparison with approved drugs**	**No special drug approval procedure**	**No consequences for patients in ongoing clinical trials or compassionate use programmes**	No ongoing clinical trials (at the time the application was withdrawn)	Cokiera, INN-dasabuvir / ombitasvir / paritaprevir / ritonavir (europa.eu)	https://www.ema.europa.eu/en/documents/withdrawal-letter/withdrawal-letter-cokiera_en.pdf	**No orphan drugs**	10/24/16
Begedina,	begelomab	Begelomab	Adienne S.r.l. S.U.	Adienne S.r.l. S.U.	**Monoclonal antibodies**	**L04AA35**	**04 Diseases of the immune system**	ICD-11 Chapter 04	graft vs host disease (GVHD) (use after hematopoietic stem cell transplantation, when steroids are not sufficiently effective)	**Enzyme (Antagonism)(N1.A)**	**/N1.A<br>//**	Enzyme (Hydrolases, Antagonism)(N1.3.A)	**/N1.3.A<br>//**	**Initial authorisation**		7/4/16	• open questions from the EMA (a17)<br>• concerns regarding the applicant's answers/information (a1)<br>• doubts about assurance of patient safety<br>reason: lack of data and/or inadequate long-term follow-up (a21.3)<br>• concerns regarding medicinal product quality (manufacturing process) (a3.1)<br>• doubts about efficacy<br>reason: lack of data to sufficiently demonstrate efficacy (a27.3)<br>• doubts about assurance of patient safety (open questions on mode of action in the body) (a21)	**/A17<br>/A1<br>/A21.3<br>/A3.1<br>/A27.3<br>/A21.<br>//**	22/07/2016	**No authorisation, because of no positive benefit-risk ratio**	**EMA evaluation**	**Number of clinical studies 02**	**Participants 0025-0049**	**No placebo control**		**No comparison with approved drugs**	**No special drug approval procedure**	**No consequences for patients in ongoing clinical trials or compassionate use programmes**	Ongoing clinical trials (at the time the application was withdrawn)	Begedina, INN - begelomab (europa.eu)	https://www.ema.europa.eu/en/documents/withdrawal-letter/withdrawal-letter-begedina_en.pdf	**Orphan drugs**	7/22/16
Arikayce,	amikacin	Amikacin Sulfate	Insmed Limited	Insmed	**antibacterial drugs**	**J01GB06**	**01 Certain infectious or parasitic diseases**	ICD-11 Chapter 01	Mycobacterium avium complex (MAC) lung disease<br><br>Note:<br>Use in persistent infection despite prior treatment	**other proteins (Antagonism/Binding) (N5.A)**	**/N5.A<br>//**	other proteins (bacterial proteins / bacterial protein synthesis, Antagonism)(N5.9.A)	**/N5.9.A<br>//**	**Initial authorisation**		6/8/16	• open questions from the EMA (a17)<br>• concerns regarding the applicant's answers/information (a1)<br>• doubts about efficacy<br>reason: lack of data to sufficiently demonstrate efficacy (a27.3)	**/A17<br>/A1<br>/A27.3<br>//**	24/06/2016	**No authorisation, because of no positive benefit-risk ratio**	**EMA evaluation**	**Number of clinical studies 01**	**Participants 0075-0099**	**Placebo controlled clinical studies**		**No comparison with approved drugs**	**No special drug approval procedure**	**No consequences for patients in ongoing clinical trials or compassionate use programmes**	Ongoing clinical trials (at the time the application was withdrawn)	Arikayce, INN-amikacin (europa.eu)	https://www.ema.europa.eu/en/documents/withdrawal-letter/withdrawal-letter-arikayce_en.pdf	**Orphan drugs**	10/14/16
Docetaxel SUN,	docetaxel	Docetaxel	Sun Pharmaceutical Industries Europe B.V.	Sun Pharmaceutical Industries	**Cytostatics**	**L01CD02**	**02 Neoplasms**	ICD-11 Chapter 02	breast cancer, non-small cell lung carcinoma (NSCLC), prostate carcinoma, adenocarcinoma of the stomach, head and neck carcinoma<br><br>Note:<br>Use alone or in combination with other tumor therapies, depending on prior treatment	**other proteins (Antagonism/Binding) (N5.A)**	**/N5.A<br>//**	other proteins (Microtubules, Antagonism) (N5.8.A)	**/N5.8.A<br>//**	**Initial authorisation**		6/6/16	• open questions from the EMA (a17)<br>• concerns regarding the applicant's answers/information (a1)<br>• concerns regarding medicinal product quality (manufacturing process, medicinal product contained in micelles, therefore bioequivalence studies to the reference product are needed or data demonstrating similarity of the composition of the product solutions) (a3.1)	**/A17<br>/A1<br>/A3.1<br>//**	24/06/2016	**No authorisation, because of no positive benefit-risk ratio**	**Marketing strategy**	**Number of clinical studies 0**	**Participants 0**	**No placebo control**	**SDG**	**Comparison with approved drugs**	**Drug application for generic medicines**	**Consequences for patients in ongoing clinical trials or compassionate use programmes unknown**	Ongoing clinical trials unknown (at the time the application was withdrawn)	Docetaxel-SUN, INN-Docetaxel Anhydrous (europa.eu)	https://www.ema.europa.eu/en/documents/withdrawal-letter/withdrawal-letter-docetaxel-sun_en.pdf	**No orphan drugs**	9/29/16

Kyndrisa,	drisapersen	Drisapersen	BioMarin International Limited	BioMarin International Limited	**Other medicines**	**M09AX04**	**08 Diseases of the nervous system**	ICD-11 Chapter 08	muscular dystrophy Duchenne<br><br>Note:<br>Use from the age of 5 when walking ability is retained and certain genetic defects are present ("Exon-51-skipping technology")	**other (N6)**	**/N6.U**<br>**//**	other (N6)	**/N6.U**<br>**//**	**Initial authorisation**		5/31/16	• open questions from the EMA (a17)<br>• concerns regarding the applicant's answers/information (a1)<br>• doubts about efficacy<br>reason: lack of data to sufficiently demonstrate efficacy (positive effect in clinical studies not sufficiently demonstrated) (a27.3)<br>• concerns regarding medicinal product quality (contaminants and/or concerns about sterilization) (a3.3)<br>• doubts about assurance of patient safety<br>reason: adverse effects (bleeding, injection site reactions) (a21.1)	**/A17**<br>**/A1**<br>**/A27.3**<br>**/A3.3**<br>**/A21.1**<br>**//**	24/06/2016	**No authorisation, because of no positive benefit-risk ratio**	**EMA evaluation**	**Number of clinical studies 03**	**Participants 0200-0299**	**Placebo controlled clinical studies**		**No comparison with approved drugs**	**No special drug approval procedure**	**Consequences for patients in ongoing clinical trials or compassionate use programmes**	Ongoing clinical trials (at the time the application was withdrawn)	Kyndrisa withdrawal Q-and-A (europa.eu)	https://www.ema.europa.eu/en/documents/withdrawal-letter/withdrawal-letter-kyndrisa_en.pdf	**Orphan drugs**	9/6/16
Adempas,	riociguat	Riociguat	Bayer AG	Bayer AG	**Stimulators of soluble guanylate cyclase (sGC)**	**C02KX05**	**11 Diseases of the circulatory system**	ICD-11 Chapter 11	pulmonary arterial hypertension PAH (in connection with congenital heart defects)<br><br>already approved indications:<br>• chronic thromboembolic pulmonary hypertension (CTEPH), use when surgery is not possible or when surgery was not sufficiently successful or PAH returns<br><br>• pulmonary arterial hypertension (PAH), use alone or in combination with endothelin receptor antagonists.<br><br>Note:<br>treatment of PAH and CTEPH of Class II-III	**Receptors (Agonism) (N4.B)**	**/N4.B**<br>**//**	Receptors (catalytic receptors, Agonism) (N4.3.B)	**/N4.3.B**<br>**//**	**Post-authorisation**	3/27/14	8/11/16	• open questions from the EMA (a17)<br>• concerns regarding the applicant's answers/information (a1)<br>• doubts about efficacy<br>reason: other reasons (no new indication-adapted study, only analysis of existing study data) (a27)<br>• doubts about efficacy<br>reason: poor study quality (small number of participants (patients fitting the indication) in analysis of existing study data) (a27.1.5)<br>• doubts about assurance of patient safety (analysis of adverse effects not sufficient) (a21)	**/A17**<br>**/A1**<br>**/A27.**<br>**/A27.1.5**<br>**/A21.**<br>**//**	16/09/2016	**No extension of authorisation, because of no positive benefit-risk ratio**	**Development strategy**	**Number of clinical studies 0**	**Participants 0**	**No placebo control**	**SD**	**No comparison with approved drugs**	**No special drug approval procedure**	**No consequences for patients in ongoing clinical trials or compassionate use programmes**	Ongoing clinical trials (at the time the application was withdrawn)	Adempas, INN - riociguat (europa.eu)	https://www.ema.europa.eu/en/documents/withdrawal-letter/withdrawal-letter-adempas_en.pdf	**Orphan drugs**	9/16/16
Alendronic Acid / Colecalciferol Mylan,	alendronic acid and colecalciferol	Alendronic acid, Colecalciferol	Mylan S.A.S	Mylan	**Bisphosphonates**<br><br>**Vitamins**	**M05BB03**	**15 Diseases of the musculoskeletal system or connective tissue**	ICD-11 Chapter 15	postmenopausal osteoporosis (with risk of vitamin D deficiency)	**Enzyme (Antagonism)(N1.A)**<br><br>**other (N6)**	**/N1.A**<br>**/N6.U**<br>**//**	Enzyme (Transferases, Antagonism) (N1.2.A)<br><br>other (N6)	**/N1.2.A**<br>**/N6.U**<br>**//**	**Initial authorisation**		5/27/16	• open questions from the EMA (a17)<br>• concerns regarding the applicant's answers/information (a1)<br>• doubts about efficacy<br>reason: poor study quality (conduct of bioequivalence study) (a27.1.6)<br>• doubts about same/improved efficacy compared with the eu reference medicinal product (similarity not sufficiently demonstrated) (a25)	**/A17**<br>**/A1**<br>**/A27.1.6**<br>**/A25**<br>**//**	24/06/2016	**No authorisation, because of no positive benefit-risk ratio**	**EMA evaluation and marketing strategy**	**Number of clinical studies 0**	**Participants 0**	**No placebo control**	**SDG**	**Comparison with approved drugs**	**Drug application for generic medicines**	**No consequences for patients in ongoing clinical trials or compassionate use programmes**	No ongoing clinical trials (at the time the application was withdrawn)	Alendronic Acid Colecalciferol Mylan, INN-alendronic acid and colecalciferol (europa.eu)	https://www.ema.europa.eu/en/documents/withdrawal-letter/withdrawal-letter-alendronic-acidcolecalciferol-mylan_en.pdf	**No orphan drugs**	2/2/17
Opsiria,	sirolimus	Sirolimus	Santen Oy	Santen Oy	**Immunosuppressants (no monoclonal antibodies)**	**S01XA23**	**09 Diseases of the visual system**	ICD-11 Chapter 09	non-infectious uveitis	**Enzyme (Antagonism)(N1.A)**	**/N1.A**<br>**//**	Enzyme (Transferases, Antagonism)(N1.2.A)	**/N1.2.A**<br>**//**	**Initial authorisation**		5/20/16	• open questions from the EMA (a17)<br>• concerns regarding the applicant's answers/information (a1)<br>• concerns regarding medicinal product quality (contaminants and/or concerns about sterilization) (a3.3)<br>• doubts about benefit (particularly in european patients) (a18)	**/A17**<br>**/A1**<br>**/A3.3**<br>**/A18**<br>**//**	27/05/2016	**No authorisation, because of no positive benefit-risk ratio**	**EMA evaluation**	**Number of clinical studies 01**	**Participants 0300-0499**	**No placebo control**		**No comparison with approved drugs**	**No special drug approval procedure**	**No consequences for patients in ongoing clinical trials or compassionate use programmes**	Ongoing clinical trials (at the time the application was withdrawn)	Opsiria, INN-sirolimus (europa.eu)	https://www.ema.europa.eu/en/documents/withdrawal-letter/withdrawal-letter-opsiria_en.pdf	**Orphan drugs**	9/30/16
Xegafri,	rociletinib	Rociletinib	Clovis Oncology UK Ltd	Covis	**Kinase inhibitors**	**L01XE**	**02 Neoplasms**	ICD-11 Chapte	non-small cell lung carcinoma (NSCLC) (use in T790M mutation after treatment with EGFR inhibitors)	**Receptors (Antagonism) (N4.A)**	**/N4.A**<br>**//**	Receptors (catalytic receptors, Antagonism) (N4.3.A)	**/N4.3.A**<br>**//**	**Initial authorisation**		5/5/16	• open questions from the EMA (a17)<br>• concerns regarding the applicant's answers/information (a1)<br>• doubts about assurance of patient safety<br>reason: adverse effects (qt prolongation, heart problems) (a21.1)<br>• doubts about efficacy<br>reason: lack of data to sufficiently demonstrate efficacy (a27.3)	**/A17**<br>**/A1**<br>**/A27.3**<br>**/A21.1**<br>**//**	27/05/2016	**No authorisation, because of no positive benefit-risk ratio**	**Marketing strategy**	**Number of clinical studies 02**	**Participants 0300-0499**	**No placebo control**		**No comparison with approved drugs**	**No special drug approval procedure**	**No consequences for patients in ongoing clinical trials or compassionate use programmes**	Ongoing clinical trials (at the time the application was withdrawn)	Xegafri, INN-rociletinib (europa.eu)	https://www.ema.europa.eu/en/documents/withdrawal-letter/withdrawal-letter-xegafri_en.pdf	**Unknown**	6/22/16
Aripiprazole Mylan,	aripiprazole	Aripiprazole	Mylan S.A.S	Mylan	**Antipsychotics**	**N05AX12**	**06 Mental, behavioral or neurological developmental disorders**	ICD-11 Chapter 06	schizophrenia (from 15 years), bipolar I disorder, prevention of manic episodes<br><br>Note:<br>Use up to 12 weeks in patients from 13 years in moderate to severe manic episodes in bipolar disorder type I, also use for the prevention of manic episodes in bipolar disorder type I	**Receptors (Agonism/Antagonism) (N4.C)**	**/N4.C**<br>**//**	Receptors (GPCR, Agonism/Antagonism) (N4.1.C)	**/N4.1.C**<br>**//**	**Initial authorisation**		1/8/16	• open questions from the EMA (a17)<br>• concerns regarding the applicant's answers/information (a1)<br>• studies supporting a biowaiver were inadequately conducted (bioequivalence not demonstrated) (a33)	**/A17**<br>**/A1**<br>**/A33**<br>**//**	29/01/2016	**No authorisation, because of no positive benefit-risk ratio**	**Manufacturing issues**	**Number of clinical studies 0**	**Participants 0**	**No placebo control**	**SDG**	**Comparison with approved drugs**	**Drug application for generic medicines**	**No consequences for patients in ongoing clinical trials or compassionate use programmes**	Ongoing clinical trials (at the time the application was withdrawn)	Aripiprazole Mylan, INN-aripiprazole (europa.eu)	https://www.ema.europa.eu/en/documents/withdrawal-letter/withdrawal-letter-aripiprazole-mylan_en.pdf-1	**No orphan drugs**	3/29/16
Veraseal,	human fibrinogen/ human thrombin	human fibrinogen/human thrombin	Instituto Grifols, S.A.	Instituto Grifols, S.A.	**Other medicines**	**B02BC**	**23 External causes of morbidity or mortality**	ICD-11 Chapter 23	bleeding during vascular surgeries (when standard operative techniques are not possible)	**other (N6)**<br><br>**Receptors (N4.U)**	**/N4.U**<br>**/N6.U**<br>**//**	other (N6)<br><br>Receptors (GPCR, Agonism) (N4.1.B)<br><br>Receptors (catalytic receptors) (N4.3)	**/N6.U**<br>**/N4.1.B**<br>**/N4.3.U**<br>**//**	**Initial authorisation**		9/29/15	• open questions from the EMA (a17)<br>• concerns regarding the applicant's answers/information (a1)<br>• doubts about compliance with good clinical practice (gcp) (a30)<br>• doubts about efficacy<br>reason: poor study quality (gcp concerns in the pivotal study) (a27.1)	**/A17**<br>**/A1**<br>**/A27.1**<br>**/A30**<br>**//**	23/10/2015	**No authorisation, because of no positive benefit-risk ratio**	**EMA evaluation**	**Number of clinical studies 01**	**Participants 0150-0199**	**No placebo control**		**Comparison with approved drugs**	**No special drug approval procedure**	**No consequences for patients in ongoing clinical trials or compassionate use programmes**	Ongoing clinical trials (at the time the application was withdrawn)	Veraseal, INN-human fibrinogen/human thrombin (europa.eu)	https://www.ema.europa.eu/en/documents/withdrawal-letter/withdrawal-letter-veraseal_en.pdf	**No orphan drugs**	10/23/15
Aripiprazole Mylan,	aripiprazole	Aripiprazole	Generics (UK) Limited	Generics (UK) Limited	**Antipsychotics**	**N05AX12**	**06 Mental, behavioral or neurological developmental disorders**	ICD-11 Chapter 06	schizophrenia (from 15 years), bipolar I disorder, prevention of manic episodes<br><br>Note:<br>Use up to 12 weeks in patients from 13 years in moderate to severe manic episodes in bipolar disorder type I, also use for the prevention of manic episodes in bipolar disorder type I	**Receptors (Agonism/Antagonism) (N4.C)**	**/N4.C**<br>**//**	Receptors (GPCR, Agonism/Antagonism) (N4.1.C)	**/N4.1.C**<br>**//**	**Initial authorisation**		5/7/15	• open questions from the EMA (a17)<br>• concerns regarding the applicant's answers/information (a1)<br>• doubts about compliance with good clinical practice (gcp) (regarding bioequivalence study) (a30)<br>• concerns regarding medicinal product quality (starting materials) (a3)	**/A17**<br>**/A1**<br>**/A3.**<br>**/A30**<br>**//**	22/05/2015	**No authorisation, because of no positive benefit-risk ratio**	**EMA evaluation**	**Number of clinical studies 0**	**Participants 0**	**No placebo control**	**SDG**	**Comparison with approved drugs**	**Drug application for generic medicines**	**No consequences for patients in ongoing clinical trials or compassionate use programmes**	No ongoing clinical trials (at the time the application was withdrawn)	Aripiprazole Mylan, INN-aripiprazole (europa.eu)	https://www.ema.europa.eu/en/documents/withdrawal-letter/withdrawal-letter-aripiprazole-mylan_en.pdf-0	**No orphan drugs**	7/20/15
Duloxetine Sandoz,	duloxetine	Duloxetine	Sandoz GmbH	Sandoz GmbH	**Serotonin-norepinephrine reuptake inhibitors (SNRI)**	**N06AX21**	**06 Mental, behavioral or neurological developmental disorders/**<br><br>**08 Diseases of the nervous system**	ICD-11 Chapter 06<br><br>ICD-11 Chapter 08	major depression, pain in diabetic peripheral<br>ne neuropathy, generalized anxiety disorder	**Transporter (Antagonism)(N3.A)**	**/N3.A**<br>**//**	Transporter (SLC-Transporters, Antagonism)(N3.1.A)	**/N3.1.A**<br>**//**	**Initial authorisation**		4/8/15	• open questions from the EMA (a17)<br>• concerns regarding the applicant's answers/information (a1)<br>• doubts about compliance with good clinical practice (gcp) (in relation to bioequivalence study) (a30)	**/A17**<br>**/A1**<br>**/A30**<br>**//**	24/04/2015	**No authorisation, because of no positive benefit-risk ratio**	**EMA evaluation**	**Number of clinical studies 0**	**Participants 0**	**No placebo control**	**SDG**	**Comparison with approved drugs**	**Drug application for generic medicines**	**No consequences for patients in ongoing clinical trials or compassionate use programmes**	Ongoing clinical trials (at the time the application was withdrawn)	Duloxetine Sandoz, INN-duloxetine (europa.eu)	https://www.ema.europa.eu/en/documents/withdrawal-letter/withdrawal-letter-duloxetine-sandoz_en.pdf	**No orphan drugs**	4/24/15
Corluxin,	mifepristone	Mifepristone	FGK Representative Service GmbH	FGK Representative Service GmbH	**selective progesterone receptor modulators (SPRM)**	**G03XB01**	**05 Endocrine diseases, nutritional or metabolic diseases**	ICD-11 Chapter 05	Cushing syndrome<br><br>Note:<br>Use when surgery is not possible or not successful	**Receptors (Agonism/Antagonism) (N4.C)**	**/N4.C**<br>**//**	Receptors (nuclear receptors, Antagonism, Agonism) (N4.4.C)	**/N4.4.C**<br>**//**	**Initial authorisation**		3/23/15	• open questions from the EMA (a17)<br>• concerns regarding the applicant's answers/information (a1)<br>• concerns regarding medicinal product quality (manufacturing process) (a3.1)<br>• concerns regarding medicinal product quality (contaminants and/or concerns about sterilization) (a3.3)<br>• doubts about efficacy<br>reason: poor study quality or inadequate study design (no comparison with placebo and/or comparator/reference medicinal products) (a27.1.4)<br>• concerns regarding the requested indication (a28)<br>• doubts about assurance of patient safety<br>reason: adverse effects (fluid and electrolyte balance) (a21.1)<br>• doubts about efficacy<br>reason: lack of data to sufficiently demonstrate efficacy (a27.3)	**/A17**<br>**/A1**<br>**/A3.1**<br>**/A3.3**<br>**/A27.1.4**<br>**/A28**<br>**/A21.1**<br>**/A27.3**<br>**//**	22/05/2015	**No authorisation, because of no positive benefit-risk ratio**	**Marketing strategy**	**Number of clinical studies 01**	**Participants 0050-0074**	**No placebo control**		**No comparison with approved drugs**	**No special drug approval procedure**	**No consequences for patients in ongoing clinical trials or compassionate use programmes**	No ongoing clinical trials (at the time the application was withdrawn)	Corluxin, INN-mifepristone (europa.eu)	https://www.ema.europa.eu/en/documents/withdrawal-letter/withdrawal-letter-corluxin_en.pdf	**Orphan drugs**	6/12/15
Ketoconazole AID-SCFM,	ketoconazole	Ketoconazole	Agenzia Industrie Difesa-Stabilimento Chimico Farmaceutico Militare	Agenzia Industrie Difesa-Stabilimento Chimico Farmaceutico Militare	**Antimycotics**	**V03**	**05 Endocrine diseases, nutritional or metabolic diseases**	ICD-11 Chapter 05	Cushing syndrome<br><br>Note:<br>Use when surgery is not possible or not successful	**Enzyme (Antagonism)(N1.A)**<br><br>**other (N6)**	**/N1.A**<br>**/N6.U**<br>**//**	Enzyme (Oxidoreductases, Antagonism)(N1.1.A)<br><br>other (N6)	**/N1.1.A**<br>**/N6.U**<br>**//**	**Initial authorisation**		2/23/15	• open questions from the EMA (a17)<br>• concerns regarding the applicant's answers/information (a1)<br>• concerns regarding medicinal product quality (starting materials) (a3)<br>• concerns regarding medicinal product quality (contaminants and/or concerns about sterilization) (a3.3)<br>• doubts about assurance of patient safety<br>reason: lack of data and/or inadequate long-term follow-up (a21.3)<br>• doubts about efficacy<br>reason: other reasons (a27)	**/A17**<br>**/A1**<br>**/A3.**<br>**/A3.3**<br>**/A21.3**<br>**/A27.**<br>**//**	27/03/2015	**No authorisation, because of no positive benefit-risk ratio**	**EMA evaluation and marketing strategy**	**Number of clinical studies 0**	**Participants 0**	**No placebo control**	**SD**	**No comparison with approved drugs**	**No special drug approval procedure**	**No consequences for patients in ongoing clinical trials or compassionate use programmes**	No ongoing clinical trials (at the time the application was withdrawn)	Ketoconazole AID-SCFM, INN-ketoconazole (europa.eu)	https://www.ema.europa.eu/en/documents/withdrawal-letter/withdrawal-letter-ketoconazole-aid-scfm_en.pdf	**Orphan drugs**	5/20/15

Rienso,	ferumoxytol	Ferumoxytol	Takeda Pharma A/S	Takeda	**Other medicines**	**B03**	**03 Diseases of the blood or blood-forming organs**	ICD-11 Chapter 03	iron deficiency anemia<br><br>Note:<br>Use when oral supplementation of iron is insufficient/inappropriate or rapid increase in iron levels is needed<br><br>already approved indications:<br>iron deficiency anemia in chronic kidney disease	**other (N6)**	**/N6.U<br>//**	other (N6)	**/N6.U<br>//**	**Post-authorisation**	6/15/12	1/19/15	• open questions from the EMA (a17)<br>• concerns regarding the applicant's answers/information (a1)<br>• doubts about assurance of patient safety<br>reason: adverse effects (allergic reactions with expanded indication) (a21.1)	**/A17<br>/A1<br>/A21.1<br>//**	27/02/2015	**No extension of authorisation, because of no positive benefit-risk ratio**	**EMA evaluation**	**Number of clinical studies 02**	**Participants 1000-1999**	**Placebo controlled clinical studies**		**Comparison with approved drugs**	**No special drug approval procedure**	**No consequences for patients in ongoing clinical trials or compassionate use programmes**	Ongoing clinical trials (at the time the application was withdrawn)	Rienso, INN: FERUMOXYTOL (europa.eu)	https://www.ema.europa.eu/en/documents/withdrawal-letter/withdrawal-letter-rienso_en.pdf	**No orphan drugs**	2/27/15
Egranli,	balugrastim	Balugrastim	Teva Pharma B.V	Teva Pharma B.V	**Immunomodulators**	**L03AA**	**04 Diseases of the immune system**	ICD-11 Chapter 04	neutropenia,<br>reduction in the occurrence of febrile neutropenia (in connection with cancer therapies)	**Receptors (Agonism) (N4.B)**	**/N4.B<br>//**	Receptors<br>(catalytic receptors, Agonism)<br>(N4.3.B)	**/N4.3.B<br>//**	**Initial authorisation**		11/4/14	• not applicable (marketing authorisation was recommended) (a14.2)	**/A14.2<br>//**	21/11/2014	**Authorisation recommended**	**Marketing strategy**	**Number of clinical studies 01**	**Participants 0300-0499**	**No placebo control**		**Comparison with approved drugs**	**No special drug approval procedure**	**No consequences for patients in ongoing clinical trials or compassionate use programmes**	No ongoing clinical trials (at the time the application was withdrawn)	Egranli, INN - balugrastim (europa.eu)	https://www.ema.europa.eu/en/documents/withdrawal-letter/withdrawal-letter-egranli_en.pdf	**No orphan drugs**	2/16/15
Imagify,	perflubutane	Perfluorobutane	Acusphere Ltd	Acusphere Ltd	**Other medicines**	**V08D**	**X Extension codes (Medicines used in diagnostic tests, not elsewhere classified)**<br><br>**11 Diseases of the circulatory system**	ICD-11 Chapter X<br><br>ICD-11 Chapter 11	imaging agent (echocardiography) for the diagnosis of coronary heart disease<br><br>Note:<br>Use in patients with stable chest pain	**other (N6)**	**/N6.U<br>//**	other (N6)	**/N6.U<br>//**	**Initial authorisation**		7/24/14	• open questions from the EMA (a17)<br>• concerns regarding the applicant's answers/information (a1)<br>• doubts about same/improved efficacy compared with the eu reference medicinal product (here diagnostic reference spect) (a25)	**/A17<br>/A1<br>/A25<br>//**	28/08/2014	**No authorisation, because of no positive benefit-risk ratio**	**Other reasons**	**Number of clinical studies unknown**	**Participants 0500-0999**	**No placebo control**		**Comparison with approved drugs**	**No special drug approval procedure**	**No consequences for patients in ongoing clinical trials or compassionate use programmes**	No ongoing clinical trials (at the time the application was withdrawn)	Withdrawal of the marketing authorisation application for Imagify (perflubutane) (europa.eu)	https://www.ema.europa.eu/en/documents/withdrawal-letter/withdrawal-letter-imagify_en.pdf	**No orphan drugs**	2/4/15
Teysuno,	tegafur, gimeracil, oteracil	Tegafur, Gimeracil, Oteracil	Nordic Group B.V.	Nordic Group B.V.	**Cytostatics**	**L01BC53**	**02 Neoplasms**	ICD-11 Chapte	expansion of use for the treatment of advanced gastric cancers with other platinum-based drugs than cisplatin<br><br>already approved indications:<br>treatment of advanced stomach cancer (in combination with cisplatin),<br>metastatic colorectal cancer when due to hand-foot syndrome or cardiovascular toxicity treatment with another fluoropyrimidine is not possible (as monotherapy or in combination with oxaliplatin or irinotecan, with or without bevacizumab)	**Enzyme (Antagonism)(N1.A)**<br><br>**other (N6)**	**/N1.A<br>/N6.U<br>//**	other (N6)<br><br>Enzyme<br>(Oxidoreductases, Antagonism)(N1.1.A)<br><br>Enzyme<br>(Transferases, Antagonism)(N1.2.A)	**/N6.U<br>/N1.1.A<br>/N1.2.A<br>//**	**Post-authorisation**	3/14/11	1/6/15	• open questions from the EMA (a17)<br>• concerns regarding the applicant's answers/information (a1)<br>• doubts about efficacy<br>reason: other reasons (lack of data) (a27)<br>• lack of data to sufficiently demonstrate efficacy (a12)	**/A17<br>/A1<br>/A27.<br>/A12<br>//**	23/01/2015	**No extension of authorisation, because of no positive benefit-risk ratio**	**EMA evaluation**	**Number of clinical studies 02**	**Participants 0025-0049**	**No placebo control**	**SD**	**Comparison with approved drugs unknown**	**No special drug approval procedure**	**No consequences for patients in ongoing clinical trials or compassionate use programmes**	Ongoing clinical trials (at the time the application was withdrawn)	Teysuno, INN-tegafur/gimeracil/oteracil (europa.eu)	https://www.ema.europa.eu/en/documents/withdrawal-letter/withdrawal-letter-teysuno_en.pdf	**Orphan drugs**	1/23/15
Neofordex,	dexamethasone	Dexamethasone	Laboratoires CTRS - Boulogne Billancourt	Laboratoires CTRS - Boulogne Billancourt	**Immunosuppressants (no monoclonal antibodies)**	**H02AB02**	**02 Neoplasms/**<br><br>**X Extension codes (Plasma cell tumors)**	ICD-11 Chapter 02<br><br>ICD-11 Chapter X	multiple myeloma	**Enzyme (Antagonism)(N1.A)**<br><br>**Receptors (Agonism)(N4.B)**	**/N1.A<br>/N4.B<br>//**	Receptors<br>(nuclear receptors, Agonism)<br>(N4.4.B)<br><br>Enzyme<br>(Hydrolases, Antagonism)(N1.3.A)	**/N4.4.B<br>/N1.3.A<br>//**	**Initial authorisation**		7/17/14	• open questions from the EMA (a17)<br>• concerns regarding the applicant's answers/information (a1)<br>• concerns regarding medicinal product quality (a3)	**/A17<br>/A1<br>/A3.<br>//**	25/07/2014	**No authorisation, because of no positive benefit-risk ratio**	**EMA evaluation**	**Number of clinical studies 0**	**Participants 0**	**No placebo control**	**SDH**	**Comparison with approved drugs**	**Drug application for hybrid medicines**	**No consequences for patients in ongoing clinical trials or compassionate use programmes**	Ongoing clinical trials (at the time the application was withdrawn)	Neofordex, inn: DEXAMETHASONE (europa.eu)	https://www.ema.europa.eu/en/documents/withdrawal-letter/withdrawal-letter-neofordex_en.pdf	**Orphan drugs**	11/20/14
Somavert,	pegvisomant	Pegvisomant	Pfizer Europe MA EEIG	Pfizer	**Other medicines**	**H01AX01**	**05 Endocrine diseases, nutritional or metabolic diseases**	ICD-11 Chapter 05	acromegaly when surgery and/or radiation therapy and/or treatment with somatostatin analogs are not sufficiently effective (25 mg and 30 mg powder or solvent)<br><br>already approved indications:<br>acromegaly when surgery and/or radiation therapy and/or treatment with somatostatin analogs are not sufficiently effective (10,15 and 20 mg powder or solvent)	**Receptors (Antagonism) (N4.A)**	**/N4.A<br>//**	Receptors<br>(catalytic receptors, Antagonism) (N4.3.A)	**/N4.3.A<br>//**	**Post-authorisation**	11/12/02	2/16/12	• open questions from the EMA (a17)<br>• concerns regarding the applicant's answers/information (a1)<br>• concerns regarding medicinal product quality (a3)<br>• doubts about assurance of patient safety (a21	**/A17<br>/A1<br>/A3.<br>/A21.<br>//**	19/09/2014	**No extension of authorisation, because of no positive benefit-risk ratio**	**EMA evaluation**	**Number of clinical studies 01**	**Participants 0001-0024**	**No placebo control**		**Comparison with approved drugs**	**No special drug approval procedure**	**Consequences for patients in ongoing clinical trials or compassionate use programmes unknown**	Ongoing clinical trials unknown (at the time the application was withdrawn)	Final withdrawal AR Somavert X-52 (europa.eu)	https://www.ema.europa.eu/en/documents/withdrawal-letter/withdrawal-letter-somavert_en.pdf	**Orphan drugs**	9/19/14
Faldaprevir Boehringer Ingelheim,	faldaprevir	Faldaprevir	Boehringer Ingelheim International GmbH	Boehringer Ingelheim International GmbH	**Antivirals**	**J05AE**	**01 Certain infectious or parasitic diseases**	ICD-11 Chapter 01	chronic hepatitis C	**Enzyme (Antagonism)(N1.A)**	**/N1.A<br>//**	Enzyme<br>(Hydrolases, Antagonism)(N1.3.A)	**/N1.3.A<br>//**	**Initial authorisation**		6/10/14	• open questions from the EMA (a17)<br>• concerns regarding the applicant's answers/information (a1)<br>• concerns regarding medicinal product quality (starting materials, release of the active ingredient from capsules) (a3)	**/A17<br>/A1<br>/A3.<br>//**	27/06/2014	**No authorisation, because of no positive benefit-risk ratio**	**Marketing strategy**	**Number of clinical studies 04**	**Participants over 2000**	**Placebo controlled clinical studies**		**Comparison with approved drugs**	**No special drug approval procedure**	**No consequences for patients in ongoing clinical trials or compassionate use programmes**	Ongoing clinical trials (at the time the application was withdrawn)	EMA-2014-0273-00-00-ENDE (europa.eu)	https://www.ema.europa.eu/en/documents/withdrawal-letter/withdrawal-letter-faldaprevir-boehringer-ingelheim_en.pdf	**No orphan drugs**	9/16/14
Neocepri,	folic acid	Folic acid	Endocyte Europe, B.V.	Endocyte Europe, B.V.	**Other medicines**	**V04**	**X Extension codes (Medicines used in diagnostic tests, not elsewhere classified)**<br><br>**02 Neoplasms**	ICD-11 Chapter 02<br><br>ICD-11 Chapter X	radionuclide imaging (SPECT scan) in ovarian cancer	**other proteins (N5.U)**	**/N5.U<br>//**	other proteins (N5)	**/N5.U<br>//**	**Initial authorisation**		5/16/14	• doubts about efficacy<br>reason: lack of data to sufficiently demonstrate efficacy (requested study data did not confirm the efficacy of vynfinit in ovarian cancer) (a27.3)	**/A27.3<br>//**	17/06/2014	**No authorisation, because of no positive benefit-risk ratio**	**EMA evaluation**	**Number of clinical studies 02**	**Participants 0025-0049**	**No placebo control**		**No comparison with approved drugs**	**No special drug approval procedure**	**Consequences for patients in ongoing clinical trials or compassionate use programmes unknown**	Ongoing clinical trials unknown (at the time the application was withdrawn)	Withdrawal of the marketing authorisation application for Neocepri (folic acid) (europa.eu)	https://www.ema.europa.eu/en/documents/withdrawal-letter/withdrawal-letter-neocepri_en.pdf	**Orphan drugs**	8/14/14
Folcepri,	etarfolatide	Etarfolatide	Endocyte Europe, B.V.	Endocyte Europe, B.V.	**Other medicines**	**V09**	**X Extension codes (Medicines used in diagnostic tests, not elsewhere classified)**<br><br>**02 Neoplasms**	ICD-11 Chapter 02<br><br>ICD-11 Chapter X	radionuclide imaging (SPECT scan) in ovarian cancer	**other proteins (N5.U)**	**/N5.U<br>//**	other proteins (N5)	**/N5.U<br>//**	**Initial authorisation**		5/16/14	• doubts about efficacy<br>reason: lack of data to sufficiently demonstrate efficacy (requested study data did not confirm the efficacy of vynfinit in ovarian cancer) (a27.3)	**/A27.3<br>//**	13/06/2014	**No authorisation, because of no positive benefit-risk ratio**	**EMA evaluation**	**Number of clinical studies 01**	**Participants 0100-0149**	**No placebo control**	**SD**	**No comparison with approved drugs**	**No special drug approval procedure**	**Consequences for patients in ongoing clinical trials or compassionate use programmes unknown**	Ongoing clinical trials unknown (at the time the application was withdrawn)	Withdrawal of the marketing authorisation application for Folcepri (etarfolatide) (europa.eu)	https://www.ema.europa.eu/en/documents/withdrawal-letter/withdrawal-letter-folcepri_en.pdf	**Orphan drugs**	8/14/14
Vynfinit,	vintafolide	Vintafolid	Endocyte Europe, B.V.	Endocyte Europe, B.V.	**Cytostatics**	**L01**	**02 Neoplasms**	ICD-11 Chapter 02	ovarian cancer with high folate receptor density (use in combination with pegylated liposomal doxorubicin when resistant to platinum-based treatment)	**other proteins (N5.U)**	**/N5.U<br>//**	other proteins (Tumor-associated antigens, Folate receptors) (N5)<br><br>other proteins (Microtubules, Antagonism) (N5.8.A)	**/N5.U<br>/N5.8.A<br>//**	**Initial authorisation**		5/16/14	• doubts about efficacy<br>reason: lack of data to sufficiently demonstrate efficacy (requested study data did not confirm the efficacy of vynfinit in ovarian cancer) (a27.3)	**/A27.3<br>//**	16/06/2014	**No authorisation, because of no positive benefit-risk ratio**	**EMA evaluation**	**Number of clinical studies 01**	**Participants 0150-0199**	**No placebo control**		**Comparison with approved drugs**	**No special drug approval procedure**	**No consequences for patients in ongoing clinical trials or compassionate use programmes**	No ongoing clinical trials (at the time the application was withdrawn)	Withdrawal of the marketing authorisation application for Vynfinit (vintafolide) (europa.eu)	https://www.ema.europa.eu/en/documents/withdrawal-letter/withdrawal-letter-vynfinit_en.pdf	**Orphan drugs**	8/14/14
Simponi,	golimumab	Golimumab	Janssen Biologics B.V.	Janssen Pharmaceutical Companies of Johnson & Johnson	**Monoclonal antibodies**	**L04AB06**	**15 Diseases of the musculoskeletal system or connective tissue/**<br><br>**13 Diseases of the digestive system**	ICD-11 Chapter 15<br><br>ICD-11 Chapter 13	Application for the approval of a concentrate (12.5 mg/ml) for intravenous infusion<br><br>Note:<br>Injection solution available in 50 and 100 mg in ready pens and syringes (s.c.)<br><br>Already approved indications:<br>active rheumatoid arthritis,<br>active and progressive psoriatic arthritis,<br>axial spondyloarthritis (including adults with severe active ankylosing spondylitis or severe non-radiographic axial spondyloarthritis),<br>moderate to severe active ulcerative colitis,<br>multilocular juvenile idiopathic arthritis	**Ligands (antagonism)(N9.A)**	**/N9.A<br>//**	Ligands<br>(Cytokines, Antagonism)(N9.2.A)	**/N9.2.A<br>//**	**Post-authorisation**	10/1/09	5/12/14	• doubts about efficacy<br>reason: other reasons (doubts about efficacy compared with s.c. injection) (a27)<br>• doubts about assurance of patient safety (a21)	**/A27.<br>/A21.<br>//**	15/07/2014	**No extension of authorisation, because of no positive benefit-risk ratio**	**EMA evaluation**	**Number of clinical studies unknown**	**Participants unknown**	**placebo control unknown**		**Comparison with approved drugs unknown**	**No special drug approval procedure**	**No consequences for patients in ongoing clinical trials or compassionate use programmes**	Ongoing clinical trials unknown (at the time the application was withdrawn)	Simponi - Rücknahme des Antrags auf Änderung der Genehmigung für das Inverkehrbringen | Europäische Arzneimittel-Agentur (europa.eu)	https://www.ema.europa.eu/en/documents/other/withdrawal-letter-simponi_en.pdf	**No orphan drugs**	7/15/14
Issarlos,	strontium ranelate /colecalciferol	Strontium ranelate/Colecalciferol	Les Laboratoires Servier	Les Laboratoires Servier	**Other medicines**	**M05BX**	**15 Diseases of the musculoskeletal system or connective tissue**	ICD-11 Chapter 15	severe osteoporosis in postmenopausal women and men (with risk of vitamin D deficiency and increased fracture risk)	**other (N6)**	**/N6.U<br>//**	other (N6)	**/N6.U<br>//**	**Initial authorisation**		3/17/14	• open questions from the EMA (a17)<br>• concerns regarding the applicant's answers/information (a1)<br>• concerns regarding medicinal product quality (lack of data on product manufacture) (a3)	**/A17<br>/A1<br>/A3.<br>//**	25/04/2014	**No authorisation, because of no positive benefit-risk ratio**	**EMA evaluation**	**Number of clinical studies 01**	**Participants 0500-0999**	**No placebo control**		**Comparison with approved drugs**	**No special drug approval procedure**	**No consequences for patients in ongoing clinical trials or compassionate use programmes**	No ongoing clinical trials (at the time the application was withdrawn)	Ditelos Issarlos_14-04_Withdrawal Q&A (europa.eu)	https://www.ema.europa.eu/en/documents/withdrawal-letter/withdrawal-letter-issarlos_en.pdf	**No orphan drugs**	7/10/14
Ditelos,	strontium ranelate /colecalciferol	Strontium ranelate/Colecalciferol	Les Laboratoires Servier	Les Laboratoires Servier	**Other medicines**	**M05BX**	**15 Diseases of the musculoskeletal system or connective tissue**	ICD-11 Chapter 15	severe osteoporosis in postmenopausal women and men (with risk of vitamin D deficiency and increased fracture risk)	**other (N6)**	**/N6.U<br>//**	other (N6)	**/N6.U<br>//**	**Initial authorisation**		3/17/14	• open questions from the EMA (a17)<br>• concerns regarding the applicant's answers/information (a1)<br>• concerns regarding medicinal product quality (lack of data on product manufacture) (a3)	**/A17<br>/A1<br>/A3.<br>//**	25/04/2014	**No authorisation, because of no positive benefit-risk ratio**	**EMA evaluation**	**Number of clinical studies 01**	**Participants 0500-0999**	**No placebo control**		**Comparison with approved drugs**	**No special drug approval procedure**	**No consequences for patients in ongoing clinical trials or compassionate use programmes**	No ongoing clinical trials (at the time the application was withdrawn)	Ditelos Issarlos_14-04_Withdrawal Q&A (europa.eu)	https://www.ema.europa.eu/en/documents/withdrawal-letter/withdrawal-letter-ditelos_en.pdf	**No orphan drugs**	7/10/14

Protelos,	strontium ranelate	Strontium ranelate	Les Laboratoires Servier	Les Laboratoires Servier	**Other medicines**	**M05BX03**	**15 Diseases of the musculoskeletal system or connective tissue**	ICD-11 Chapter 15	osteoarthritis of the knee and hip<br><br>already approved indications:<br>severe osteoporosis in postmenopausal women and men (with increased fracture risk)	**other (N6)**	**/N6.U<br>//**	other (N6)	**/N6.U<br>//**	**Post-authorisation**	9/20/04	3/21/14	• open questions from the EMA (a17)<br>• concerns regarding the applicant's answers/information (a1)<br>• doubts about efficacy<br>reason: inadequate study design (concerns about the procedure for controlling cartilage loss) (a27.2)<br>• doubts about assurance of patient safety<br>reason: adverse effects (demonstrated risks of serious adverse reactions) (a21.1)<br>• doubts about benefit (compared with risks) (a18)	**/A17<br>/A1<br>/A27.2<br>/A21.1<br>/A18<br>//**	23/05/2014	**No extension of authorisation, because of no positive benefit-risk ratio**	**EMA evaluation**	**Number of clinical studies 01**	**Participants 1000-1999**	**Placebo controlled clinical studies**		**No comparison with approved drugs**	**No special drug approval procedure**	**No consequences for patients in ongoing clinical trials or compassionate use programmes**	Ongoing clinical trials (at the time the application was withdrawn)	Protelos, INN-strontium ranelate (europa.eu)	https://www.ema.europa.eu/en/documents/other/withdrawal-letter-protelos_en.pdf	**No orphan drugs**	7/1/14
Osseor,	strontium ranelate	Strontium ranelate	Les Laboratoires Servier	Les Laboratoires Servier	**Other medicines**	**M05BX03**	**15 Diseases of the musculoskeletal system or connective tissue**	ICD-11 Chapter 15	osteoarthritis of the knee and hip<br><br>already approved indications:<br>severe osteoporosis in postmenopausal women and men (with increased fracture risk)	**other (N6)**	**/N6.U<br>//**	other (N6)	**/N6.U<br>//**	**Post-authorisation**	9/20/04	3/21/14	• open questions from the EMA (a17)<br>• concerns regarding the applicant's answers/information (a1)<br>• doubts about efficacy<br>reason: inadequate study design (concerns about the procedure for controlling cartilage loss) (a27.2)<br>• doubts about assurance of patient safety<br>reason: adverse effects (demonstrated risks of serious adverse reactions) (a21.1)<br>• doubts about benefit (compared with risks) (a18)	**/A17<br>/A1<br>/A27.2<br>/A21.1<br>/A18<br>//**	23/05/2014	**No extension of authorisation, because of no positive benefit-risk ratio**	**EMA evaluation**	**Number of clinical studies 01**	**Participants 1000-1999**	**Placebo controlled clinical studies**		**No comparison with approved drugs**	**No special drug approval procedure**	**No consequences for patients in ongoing clinical trials or compassionate use programmes**	No ongoing clinical trials (at the time the application was withdrawn)	Osseor, INN-strontium ranelate (europa.eu)	https://www.ema.europa.eu/en/documents/other/withdrawal-letter-osseor_en.pdf	**No orphan drugs**	7/1/14
Tasigna,	nilotinib	Nilotinib	Novartis Europharm Limited	Novartis	**Kinase inhibitors**	**L01EA03**	**X Extension codes (Leukemias) /<br><br>02 Neoplasms**	IICD-11 Chapter 02<br><br>ICD-11 Chapter X	chronic myeloid leukemia (CML) (Philadelphia chromosome-positive)<br>Use when there is no complete molecular response to therapy with imatinib<br><br>already approved indications:<br>chronic myeloid leukemia (CML) (Philadelphia chromosome-positive)<br><br>Note:<br>Use under special conditions	**Enzyme (Antagonism)(N1.A)**	**/N1.A<br>//**	Enzyme (Transferases, Antagonism)(N1.2.A)	**/N1.2.A<br>//**	**Post-authorisation**	11/19/07	5/21/14	• concerns regarding the applicant's answers/information (a1)<br>• doubts about efficacy<br>reason: poor study quality (dose of comparator imatinib possibly too low) (a27.1)<br>• doubts about assurance of patient safety<br>reason: adverse effects (a21.1)<br>• doubts about efficacy<br>reason: other reasons (improvement in treatment outcomes through complete molecular response questionable) (a27)<br>• doubts about same/improved efficacy compared with the eu reference medicinal product (a25)<br>• doubts about efficacy<br>reason: lack of data to sufficiently demonstrate efficacy (a27.3)	**/A1<br>/A27.1<br>/A21.1<br>/A27.<br>/A25<br>/A27.3<br>//**	27/06/2014	**No extension of authorisation, because of no positive benefit-risk ratio**	**EMA evaluation**	**Number of clinical studies 01**	**Participants 0200-0299**	**No placebo control**		**Comparison with approved drugs**	**No special drug approval procedure**	**No consequences for patients in ongoing clinical trials or compassionate use programmes**	Ongoing clinical trials (at the time the application was withdrawn)	Tasigna II-61_14_06_withdrawal Q&A (europa.eu)	https://www.ema.europa.eu/en/documents/other/withdrawal-letter-tasigna_en.pdf	**Orphan drugs**	6/27/14
Heplisav,	common name: Hepatitis B vaccine (rDNA, adjuvanted)	(Hepatitis-B-(rDNA)-vaccine, adjuvanted)	Dynavax International B.V.	Dynavax International B.V.	**Other medicines**	**J07BC01**	**X Extension codes (viral vaccines)**	ICD-11 Chapter X	vaccine against hepatitis B	**other (N6)**	**/N6.U<br>//**	other (N6)	**/N6.U<br>//**	**Initial authorisation**		2/10/14	• open questions from the EMA (a17)<br>• concerns regarding the applicant's answers/information (a1)<br>• doubts about efficacy<br>reason: poor study quality (study conduct in patients with chronic kidney disease) (a27.1.6)<br>• doubts about efficacy<br>reason: poor study quality (poor documentation in patients with chronic kidney disease) (a27.1)<br>• doubts about efficacy<br>reason: poor study quality (small number of participants in safety studies) (a27.1.5)<br>• doubts about compliance with good clinical practice (gcp) (a30)	**/A17<br>/A1<br>/A27.1.6<br>/A27.1<br>/A27.1.5<br>/A30<br>//**	21/02/2014	**No authorisation, because of no positive benefit-risk ratio**	**EMA evaluation**	**Number of clinical studies 03**	**Participants over 2000**	**No placebo control**		**Comparison with approved drugs**	**No special drug approval procedure**	**No consequences for patients in ongoing clinical trials or compassionate use programmes**	No ongoing clinical trials (at the time the application was withdrawn)	Heplisav, hepatitis B (rDNA) vaccine, adjuvanted (europa.eu)	https://www.ema.europa.eu/en/documents/withdrawal-letter/heplisav-withdrawal-letter_en.pdf	**No orphan drugs**	5/26/14
Votrient,	pazopanib	pazopanib	Novartis Europharm Limited	Novartis	**Kinase inhibitors**	**L01XE11**	**02 Neoplasms**	ICD-11 Chapte	tubal cancer, ovarian cancer, peritoneal carcinosis (each maintenance therapies)<br><br>already approved indications: renal cell carcinoma	**Receptors (Antagonism) (N4.A)**	**/N4.A<br>//**	Receptors (catalytic receptors, Antagonism) (N4.3.A)	**/N4.3.A<br>//**	**Post-authorisation**	6/14/10	3/27/14	• open questions from the EMA (a17)<br>• concerns regarding the applicant's answers/information (a1)<br>• doubts about efficacy<br>reason: patients in study not compatible with target indication (a27.4)<br>• doubts about efficacy<br>reason: lack of data to sufficiently demonstrate efficacy (with respect to older fEMAle patients) (a27.3)<br>• doubts about assurance of patient safety<br>reason: adverse effects (a21.1)	**/A17<br>/A1<br>/A27.4<br>/A27.3<br>/A21.1<br>//**	25/04/2014	**No extension of authorisation, because of no positive benefit-risk ratio**	**EMA evaluation**	**Number of clinical studies 01**	**Participants 0500-0999**	**Placebo controlled clinical studies**		**No comparison with approved drugs**	**No special drug approval procedure**	**No consequences for patients in ongoing clinical trials or compassionate use programmes**	Ongoing clinical trials (at the time the application was withdrawn)	https://www.ema.europa.eu/en/documents/medicine-qa/questions-and-answers-withdrawal-application-change-marketing-authorisation-votrient-pazopanib_en.pdf	https://www.ema.europa.eu/en/documents/other/withdrawal-letter-votrient_en.pdf	**No orphan drugs**	4/25/14
Winfuran,	nalfurafin	nalfurafine	Toray International U.K. Limited	Toray International U.K. Limited	**Opioids**	**V03AX02**	**14 Skin diseases**	ICD-11 Chapter 14	uremic pruritus in kidney insufficiency	**Receptors (Agonism/Antagonism) (N4.C)**	**/N4.C<br>//**	Receptors (GPCR, Agonism/Antagonism) (N4.1.C)	**/N4.1.C<br>//**	**Initial authorisation**		1/17/14	• concerns regarding the applicant's answers/information (a1)<br>• doubts about efficacy<br>reason: lack of data to sufficiently demonstrate efficacy (a27.3)<br>• doubts about benefit (a18)	**/A1<br>/A18<br>/A27.3<br>//**	24/01/2014	**No authorisation, because of no positive benefit-risk ratio**	**EMA evaluation**	**Number of clinical studies 01**	**Participants 0300-0499**	**Placebo controlled clinical studies**		**No comparison with approved drugs**	**No special drug approval procedure**	**No consequences for patients in ongoing clinical trials or compassionate use programmes**	No ongoing clinical trials (at the time the application was withdrawn)	https://www.ema.europa.eu/de/documents/medicine-qa/withdrawal-marketing-authorisation-application-winfuran-nalfurafine_de.pdf	https://www.ema.europa.eu/en/documents/withdrawal-letter/withdrawal-letter-winfuran_en.pdf	**Orphan drugs**	3/11/14
Firazyr,	icatibant	icatibant	Takeda Pharmaceuticals International AG	Takeda	**Other medicines**	**B06AC02**	**14 Skin diseases**	ICD-11 Chapter 14	angioedema (caused by angiotensin-converting enzyme inhibitors)<br><br>already approved indications:<br>hereditary angioedema	**Receptors (Antagonism) (N4.A)**	**/N4.A<br>//**	Receptors (GPCR, Antagonism) (N4.1.A)	**/N4.1.A<br>//**	**Post-authorisation**	7/11/08	2/14/14	• concerns regarding the applicant's answers/information (a1)<br>• doubts about efficacy<br>reason: poor study quality (conduct) (a27.1.6)<br>• doubts about efficacy<br>reason: poor study quality (documentation) (a27.1)<br>• concerns regarding inconsistent information in the application and documentation (a7)<br>• doubts about efficacy<br>reason: lack of data to sufficiently demonstrate efficacy (a27.3)	**/A1<br>/A7<br>/A27.1.6<br>/A27.1<br>/A27.3<br>//**	21/02/2014	**No extension of authorisation, because of no positive benefit-risk ratio**	**EMA evaluation**	**Number of clinical studies 01**	**Participants 0025-0049**	**No placebo control**		**Comparison with approved drugs**	**No special drug approval procedure**	**No consequences for patients in ongoing clinical trials or compassionate use programmes**	Ongoing clinical trials (at the time the application was withdrawn)	https://www.ema.europa.eu/en/documents/medicine-qa/questions-and-answers-withdrawal-application-change-marketing-authorisation-firazyr-icatibant_en.pdf	https://www.ema.europa.eu/en/documents/other/withdrawal-letter-firazyr-icatibant_en.pdf	**Orphan drugs**	2/21/14
Prometax,	rivastigmine	Rivastigmine	Novartis Europharm Limited	Novartis	**Other medicines**	**N06DA03**	**06 Mental, behavioral or neurological developmental disorders**	ICD-11 Chapter 06	Alzheimer's disease, severe (transdermal patch)<br><br>Already approved indications:<br>Alzheimer's disease (mild to moderate); dementia in Parkinson's disease (mild to moderate)	**Enzyme (Antagonism)(N1.A)**	**/N1.A<br>//**	Enzyme (Hydrolases, Antagonism)(N1.3.A)	**/N1.3.A<br>//**	**Post-authorisation**	12/4/98	11/21/13	• open questions from the EMA (a17)<br>• concerns regarding the applicant's answers/information (a1)<br>• doubts about benefit (a18)<br>• doubts about assurance of patient safety<br>reason: adverse effects (a21.1)	**/A17<br>/A1<br>/A18<br>/A21.1<br>//**	20/12/2013	**No extension of authorisation, because of no positive benefit-risk ratio**	**EMA evaluation**	**Number of clinical studies 01**	**Participants 0500-0999**	**No placebo control**		**No comparison with approved drugs**	**No special drug approval procedure**	**No consequences for patients in ongoing clinical trials or compassionate use programmes**	Ongoing clinical trials (at the time the application was withdrawn)	https://www.ema.europa.eu/en/documents/medicine-qa/questions-and-answers-withdrawal-application-change-marketing-authorisation-exelon-and-prometax-rivastigmine_en.pdf-0	https://www.ema.europa.eu/en/documents/other/withdrawal-letter-exelon-and-prometax-rivastigmine_en.pdf	**No orphan drugs**	2/18/14
Exelon,	rivastigmine	Rivastigmine	Novartis Europharm Limited	Novartis	**Other medicines**	**N06DA03**	**06 Mental, behavioral or neurological developmental disorders**	ICD-11 Chapter 06	Alzheimer's disease, severe (transdermal patch)<br><br>Already approved indications:<br>Alzheimer's disease (mild to moderate); dementia in Parkinson's disease (mild to moderate)	**Enzyme (Antagonism)(N1.A)**	**/N1.A<br>//**	Enzyme (Hydrolases, Antagonism)(N1.3.A)	**/N1.3.A<br>//**	**Post-authorisation**	12/4/98	11/21/13	• open questions from the EMA (a17)<br>• concerns regarding the applicant's answers/information (a1)<br>• doubts about benefit (a18)<br>• doubts about assurance of patient safety<br>reason: adverse effects (a21.1)	**/A17<br>/A1<br>/A18<br>/A21.1<br>//**	20/12/2013	**No extension of authorisation, because of no positive benefit-risk ratio**	**EMA evaluation**	**Number of clinical studies 01**	**Participants 0500-0999**	**No placebo control**		**No comparison with approved drugs**	**No special drug approval procedure**	**No consequences for patients in ongoing clinical trials or compassionate use programmes**	Ongoing clinical trials (at the time the application was withdrawn)	https://www.ema.europa.eu/en/documents/medicine-qa/questions-and-answers-withdrawal-application-change-marketing-authorisation-exelon-and-prometax-rivastigmine_en.pdf-0	https://www.ema.europa.eu/en/documents/other/withdrawal-letter-exelon-and-prometax-rivastigmine_en.pdf	**No orphan drugs**	2/18/14
Ceprotin,	human protein C	human protein C	Takeda Manufacturing Austria AG	Takeda	**Anticoagulants**	**B01AD12**	**03 Diseases of the blood or blood-forming organs**	ICD-11 Chapter 03	purpura fulminans;<br>protein C deficiency (acquired)<br><br>already approved indications:<br>severe congenital protein C deficiency, short-term prophylaxis of coagulation in patients with severe congenital protein C deficiency	**Enzyme (Antagonism)(N1.A)<br><br>other (N6)**	**/N1.A<br>/N6.U<br>//**	other (N6)<br><br>Enzyme (Hydrolases, Antagonism)(N1.3.A)	**/N6.U<br>/N1.3.A<br>//**	**Post-authorisation**	7/16/01	10/22/14	• not available (documents have not yet been reviewed or recommendations not yet published) (a14.1)	**/A14.1<br>//**	21/11/2013	**None (documents have not yet been evaluated)**	**EMA evaluation**	**Number of clinical studies 02**	**Participants 0050-0074**	**Placebo controlled clinical studies**	**SD**	**No comparison with approved drugs**	**No special drug approval procedure**	**No consequences for patients in ongoing clinical trials or compassionate use programmes**	Ongoing clinical trials (at the time the application was withdrawn)	https://www.ema.europa.eu/en/documents/medicine-qa/questions-and-answers-withdrawal-application-change-marketing-authorisation-ceprotin-human-protein-c_en.pdf	https://www.ema.europa.eu/en/documents/withdrawal-letter/withdrawal-letter-ceprotin_en.pdf	**No orphan drugs**	11/21/13
Omontys,	peginesatid	peginesatide	Takeda Global Research and Development Centre (Europe)	Takeda	**Other medicines**	**B03XA**	**03 Diseases of the blood or blood-forming organs**	ICD-11 Chapter 03	anemia (symptomatic, in connection with chronic kidney disease with dialysis)	**Receptors (Agonism) (N4.B)**	**/N4.B<br>//**	Receptors (catalytic receptors, Agonism) (N4.3.B)	**/N4.3.B<br>//**	**Initial authorisation**		6/28/13	• open questions from the EMA (a17)<br>• concerns regarding the applicant's answers/information (a1)<br>• doubts about assurance of patient safety<br>reason: adverse effects (hypersensitivity reactions, death or cardiovascular problems in patients treated for the first time compared with reference products) (a21.1)<br>• doubts about efficacy<br>reason: poor study quality (a27.1)	**/A17<br>/A1<br>/A21.1<br>/A27.1<br>//**	28/06/2013	**No authorisation, because of no positive benefit-risk ratio**	**EMA evaluation**	**Number of clinical studies 02**	**Participants 1000-1999**	**No placebo control**		**Comparison with approved drugs**	**No special drug approval procedure**	**No consequences for patients in ongoing clinical trials or compassionate use programmes**	No ongoing clinical trials (at the time the application was withdrawn)	https://www.ema.europa.eu/en/documents/medicine-qa/questions-and-answers-withdrawal-marketing-authorisation-application-omontys_en.pdf	https://www.ema.europa.eu/en/documents/withdrawal-letter/withdrawal-letter-omontys_en.pdf	**No orphan drugs**	6/28/13
Effentora,	fentanyl	Fentanyl	Teva Pharma B.V	Teva Pharma B.V	**Opioids**	**N02AB03**	**21 Symptoms, signs or clinical findings not elsewhere classified (chronic pain, unspecified)**	ICD-11 Chapter 21	chronic pain without cancer disease<br><br>already approved indication:<br>chronic pain in cancer diseases	**Receptors (Agonism) (N4.B)**	**/N4.B<br>//**	Receptors (GPCR, Agonism) (N4.1.B)	**/N4.1.B<br>//**	**Post-authorisation**	4/4/08	7/11/13	• open questions from the EMA (a17)<br>• concerns regarding the applicant's answers/information (a1)<br>• doubts about assurance of patient safety<br>reason: lack of data and/or inadequate long-term follow-up (a21.3)<br>• doubts about efficacy<br>reason: patients in study not compatible with target indication (a27.4)<br>• doubts about assurance of patient safety<br>reason: adverse effects (addiction) (a21.1)	**/A17<br>/A1<br>/A21.3<br>/A27.4<br>/A21.1<br>//**	26/07/2013	**No extension of authorisation, because of no positive benefit-risk ratio**	**EMA evaluation**	**Number of clinical studies 03**	**Participants 0300-0499**	**Placebo controlled clinical studies**		**No comparison with approved drugs**	**No special drug approval procedure**	**No consequences for patients in ongoing clinical trials or compassionate use programmes**	Ongoing clinical trials (at the time the application was withdrawn)	https://www.ema.europa.eu/en/documents/medicine-qa/questions-and-answers-withdrawal-application-change-marketing-authorisation-effentora-fentanyl_en.pdf	https://www.ema.europa.eu/en/documents/other/withdrawal-letter-effentora_en.pdf	**No orphan drugs**	8/29/13

IXinity,	trenonacog alfa?	Trenonacog alfa	Cangene Europe Limited	Cangene Europe Limited	**Other medicines**	**B02BD12**	**03 Diseases of the blood or blood-forming organs**	ICD-11 Chapter 03	hemophilia B	**Enzyme (Agonism)(N1.B)**<br><br>**other (N6)**	**/N1.B<br>/N6.U<br>//**	other (N6)<br><br>Enzyme (Hydrolases, Activation)(N1.3.B)	**/N6.U<br>/N1.3.B<br>//**	**Initial authorisation**		6/13/13	• open questions from the EMA (a17)<br>• concerns regarding the applicant's answers/information (a1)<br>• doubts about assurance of patient safety<br>reason: (patients partly developed antibodies against proteins from the cells used for manufacture) (a21)<br>• doubts about assurance of patient safety<br>reason: lack of data and/or inadequate long-term follow-up (lack of data on mode of action of the product after refinement of the manufacturing process due to antibody formation) (a21.3)	**/A17<br>/A1<br>/A21.<br>/A21.3<br>//**	28/06/2013	**No authorisation, because of no positive benefit-risk ratio**	**EMA evaluation**	**Number of clinical studies 03**	**Participants 0025-0049**	**No placebo control**		**Comparison with approved drugs unknown**	**No special drug approval procedure**	**No consequences for patients in ongoing clinical trials or compassionate use programmes**	Ongoing clinical trials (at the time the application was withdrawn)	https://www.ema.europa.eu/en/documents/medicine-qa/questions-and-answers-withdrawal-marketing-authorisation-application-ixinity_en.pdf	https://www.ema.europa.eu/en/documents/withdrawal-letter/withdrawal-letter-ixinity_en.pdf	**No orphan drugs**	9/18/13
Belviq,	lorcaserin	lorcaserin	Arena Pharmaceutical Enterprises Limited	Arena Pharmaceutical Enterprises Limited	**Other medicines**	**A08AA**	**05 Endocrine diseases, nutritional or metabolic diseases**	ICD-11 Chapter 05	obesity (BMI of at least 30 kg/m2)	**Receptors (Agonism) (N4.B)**	**/N4.B<br>//**	Receptors (GPCR, Agonism) (N4.1.B)	**/N4.1.B<br>//**	**Initial authorisation**		5/3/13	• open questions from the EMA (a17)<br>• concerns regarding the applicant's answers/information (a1)<br>• doubts about assurance of patient safety<br>reason: adverse effects (tumour risk, psychiatric disorders and valvulopathy) (a21.1)	**/A17<br>/A1<br>/A21.1<br>//**	31/05/2013	**No authorisation, because of no positive benefit-risk ratio**	**EMA evaluation**	**Number of clinical studies 03**	**Participants over 2000**	**Placebo controlled clinical studies**		**No comparison with approved drugs**	**No special drug approval procedure**	**No consequences for patients in ongoing clinical trials or compassionate use programmes**	Ongoing clinical trials (at the time the application was withdrawn)	https://www.ema.europa.eu/en/documents/medicine-qa/questions-and-answers-withdrawal-marketing-authorisation-application-belviq_en.pdf	https://www.ema.europa.eu/en/documents/withdrawal-letter/withdrawal-letter-belviq_en.pdf	**No orphan drugs**	8/20/13
Raxone,	idebenone	Idebenone	Santhera Pharmaceuticals (Deutschland) GmbH	Santhera Pharmaceuticals (Deutschland) GmbH	**Other medicines**	**N06BX13**	**08 Diseases of the nervous system**	ICD-11 Chapter 08	Leber's hereditary optic neuropathy (LHON)	**other (N6)**	**/N6.U<br>//**	other (N6)	**/N6.U<br>//**	**Initial authorisation**		3/21/13	• concerns regarding the applicant's answers/information (a1)<br>• doubts about efficacy<br>reason: patients in study not compatible with target indication (with respect to change of the requested indication) (a27.4)<br>• doubts about efficacy<br>reason: lack of data to sufficiently demonstrate efficacy (a27.3)	**/A1<br>/A27.4<br>/A27.3<br>//**	26/04/2013	**No authorisation, because of no positive benefit-risk ratio**	**Strategic reasons**	**Number of clinical studies 01**	**Participants 0075-0099**	**Placebo controlled clinical studies**		**No comparison with approved drugs**	**No special drug approval procedure**	**No consequences for patients in ongoing clinical trials or compassionate use programmes**	No ongoing clinical trials (at the time the application was withdrawn)	https://www.ema.europa.eu/en/documents/medicine-qa/questions-and-answers-withdrawal-marketing-authorisation-application-raxone_en.pdf	https://www.ema.europa.eu/en/documents/withdrawal-letter/withdrawal-letter-raxone_en.pdf	**Orphan drugs**	8/6/13
Eviplera,	emtricitabine, rilpivirine, tenofovir disoproxil	Emtricitabine, Rilpivirine Hydrochloride, Tenofovir Disoproxil fumarate	Gilead Sciences International Ltd	Gilead Sciences	**Antivirals**	**J05AR08**	**01 Certain infectious or parasitic diseases**	ICD-11 Chapter 01	HIV infections (treatment of HIV-1 in previously untreated adult patients (viral load between 100,000 and 500,000 copies/ml)<br><br>already approved indications: treatment of HIV-1 in previously untreated adult patients (viral load of 100,000 copies/ml or less)	**Enzyme (Antagonism)(N1.A)**	**/N1.A<br>//**	Enzyme (Transferases, Antagonism)(N1.2.A)	**/N1.2.A<br>//**	**Post-authorisation**	11/27/11	7/16/13	• open questions from the EMA (a17)<br>• concerns regarding the manufacturer's responses/information (a1)<br>• doubts about efficacy<br>reason: other reasons (with viral loads over 100,000 copies/ml increased risk of treatment failure and resistance) (a27)	**/A17<br>/A1<br>/A27.<br>//**	26/07/2013	**No extension of authorisation, because of no positive benefit-risk ratio**	**EMA evaluation**	**Number of clinical studies 01**	**Participants 0500-0999**	**No placebo control**		**Comparison with approved drugs**	**No special drug approval procedure**	**No consequences for patients in ongoing clinical trials or compassionate use programmes**	Ongoing clinical trials (at the time the application was withdrawn)	https://www.ema.europa.eu/en/documents/medicine-qa/questions-and-answers-withdrawal-application-change-marketing-authorisation-eviplera-emtricitabine-rilpivirine-tenofovir-disoproxil_en.pdf	https://www.ema.europa.eu/en/documents/other/withdrawal-letter-eviplera_en.pdf	**No orphan drugs**	7/26/13
OraNera,	autologe orale Schleimhautepithelzellen?	autologous oral mucosal epithelial cells	CellSeed Europe Ltd	CellSeed Europe Ltd	**Other medicines**	**Unknown**	**09 Diseases of the visual system**	ICD-11 Chapter 09	limbal stem cell deficiency (LSCD) (corneal disease)	**other (N6)**	**/N6.U<br>//**	other (N6)	**/N6.U<br>//**	**Initial authorisation**		3/14/13	• open questions from the EMA (a17)<br>• concerns regarding the manufacturer's responses/information (a1)<br>• doubts about efficacy<br>reason: lack of data to adequately demonstrate efficacy (a27.3)<br>• concerns regarding medicinal product quality (a3)<br>• doubts about efficacy<br>reason: poor study quality (conduct) (a27.1.6)	**/A17<br>/A1<br>/A27.3<br>/A3.<br>/A27.1.6<br>//**	22/03/2013	**No authorisation, because of no positive benefit-risk ratio**	**EMA evaluation**	**Number of clinical studies 01**	**Participants 0025-0049**	**No placebo control**		**No comparison with approved drugs**	**No special drug approval procedure**	**No consequences for patients in ongoing clinical trials or compassionate use programmes**	No ongoing clinical trials (at the time the application was withdrawn)	https://www.ema.europa.eu/en/documents/medicine-qa/questions-and-answers-withdrawal-marketing-authorisation-application-oranera_en.pdf	https://www.ema.europa.eu/en/documents/withdrawal-letter/withdrawal-letter-oranera_en.pdf	**No orphan drugs**	7/30/13
Tysabri,	natalizumab	Natalizumab	Biogen Netherlands B.V.	Biogen Netherlands B.V.	**Monoclonal antibodies**	**L04AG03**	**08 Diseases of the nervous system**	ICD-11 Chapter 08	multiple sclerosis (not highly active, no antibodies against JC virus)<br><br>already approved indications: multiple sclerosis (highly active, relapsing, remitting, when treatment with beta-interferon is not sufficient)	**Receptors (Antagonism) (N4.A)**	**/N4.A<br>//**	Receptors (catalytic receptors, Antagonism) (N4.3.A)	**/N4.3.A<br>//**	**Post-authorisation**	6/27/06	5/14/13	• concerns regarding the manufacturer's responses/information (a1)<br>• doubts about ensuring patient safety<br>reason: possible overtreatment (risks) in patients with not highly active multiple sclerosis (a21)<br>• doubts about ensuring patient safety<br>reason: jc virus antibody status can change or be false negative, relevant with regard to risk for pml (progressive multifocal leukoencephalopathy) (a21)	**/A1<br>/A21.<br>//**	31/05/2013	**No extension of authorisation, because of no positive benefit-risk ratio**	**EMA evaluation**	**Number of clinical studies 0**	**Participants 0**	**No placebo control**	**SD**	**No comparison with approved drugs**	**No special drug approval procedure**	**No consequences for patients in ongoing clinical trials or compassionate use programmes**	Ongoing clinical trials (at the time the application was withdrawn)	https://www.ema.europa.eu/en/documents/medicine-qa/questions-and-answers-withdrawal-application-change-marketing-authorisation-tysabri-natalizumab_en.pdf	https://www.ema.europa.eu/en/documents/other/withdrawal-letter-tysabri_en.pdf	**No orphan drugs**	5/31/13
Fanaptum,	iloperidone	Iloperidone	Vanda Pharmaceuticals Ltd	Vanda Pharmaceuticals Ltd	**Antipsychotics**	**N05AX14**	**06 Mental, behavioral or neurological developmental disorders**	ICD-11 Chapter 06	schizophrenia	**Receptors (Antagonism) (N4.A)**	**/N4.A<br>//**	Receptors (GPCR, Antagonism) (N4.1.A)	**/N4.1.A<br>//**	**Initial authorisation**		3/13/13	• concerns regarding the manufacturer's responses/information (a1)<br>• doubts about efficacy<br>reason: lack of data to adequately demonstrate efficacy (especially long-term efficacy) (a27.3)<br>• doubts about ensuring patient safety<br>reason: adverse reactions (arrhythmias) (a21.1)	**/A1<br>/A27.3<br>/A21.1<br>//**	22/03/2013	**No authorisation, because of no positive benefit-risk ratio**	**EMA evaluation**	**Number of clinical studies 04**	**Participants over 2000**	**Placebo controlled clinical studies**		**No comparison with approved drugs**	**No special drug approval procedure**	**No consequences for patients in ongoing clinical trials or compassionate use programmes**	Ongoing clinical trials (at the time the application was withdrawn)	https://www.ema.europa.eu/en/documents/medicine-qa/questions-and-answers-withdrawal-marketing-authorisation-application-fanaptum_en.pdf	https://www.ema.europa.eu/en/documents/withdrawal-letter/withdrawal-letter-fanaptum_en.pdf	**No orphan drugs**	5/17/13
Ruvise,	imatinib	Imatinib	Novartis Europharm Limited	Novartis	**Kinase inhibitors**	**L01EA01**	**11 Diseases of the circulatory system**	ICD-11 Chapter 11	pulmonary arterial hypertension (PAH, adjunct therapy after insufficient treatment by at least two specific drugs)	**Enzyme (Antagonism)(N1.A)**	**/N1.A<br>//**	Enzyme (Transferases, Antagonism)(N1.2.A)	**/N1.2.A<br>//**	**Initial authorisation**		2/21/13	• open questions from the EMA (a17)<br>• concerns regarding the manufacturer's responses/information (a1)<br>• doubts about efficacy<br>reason: lack of data to adequately demonstrate efficacy (a27.3)<br>• doubts about ensuring patient safety<br>reason: adverse reactions (a21.1)<br>• doubts about ensuring patient safety<br>reason: adverse reactions (bleeding) (a21.1.1)	**/A17<br>/A1<br>/A27.3<br>/A21.1<br>/A21.1.1<br>//**	22/02/2013	**No authorisation, because of no positive benefit-risk ratio**	**EMA evaluation**	**Number of clinical studies 01**	**Participants 0200-0299**	**Placebo controlled clinical studies**		**Comparison with approved drugs**	**No special drug approval procedure**	**No consequences for patients in ongoing clinical trials or compassionate use programmes**	Ongoing clinical trials (at the time the application was withdrawn)	https://www.ema.europa.eu/en/documents/medicine-qa/questions-and-answers-withdrawal-marketing-authorisation-application-ruvise_en.pdf	https://www.ema.europa.eu/en/documents/withdrawal-letter/withdrawal-letter-ruvise_en.pdf	**No orphan drugs**	4/10/13
Hyalograft C autograft,	autologe humane Chondrozyten in vitro expandiert	Autologous human chondrocytes (expanded in vitro)	Anika Therapeutics S.r.l.	Anika Therapeutics S.r.l.	**Other medicines**	**M09AX02**	**22 Injuries, poisonings or certain other consequences of external causes**	ICD-11 Chapter 22	cartilage defects in the knee	**other (N6)**	**/N6.U<br>//**	other (N6)	**/N6.U<br>//**	**Initial authorisation**		1/14/13	• open questions from the EMA (a17)<br>• concerns regarding the manufacturer's responses/information (a1)<br>• concerns regarding medicinal product quality (manufacturing process) (a3.1)<br>• doubts about efficacy<br>reason: poor study quality (conduct) (a27.1.6)	**/A17<br>/A1<br>/A3.1<br>/A27.1.6<br>//**	27/02/2013	**No authorisation, because of no positive benefit-risk ratio**	**EMA evaluation**	**Number of clinical studies 0**	**Participants 0**	**No placebo control**	**SD**	**No comparison with approved drugs**	**No special drug approval procedure**	**Consequences for patients in ongoing clinical trials or compassionate use programmes**	No ongoing clinical trials (at the time the application was withdrawn)	https://www.ema.europa.eu/en/documents/medicine-qa/questions-and-answers-withdrawal-marketing-authorisation-application-hyalograft-c-autograft-characterised-viable-autologous-chondrocytes-expanded-vitro-seeded-and-cultured-hyaluronan-based-scaffol_en.pdf	https://www.ema.europa.eu/en/documents/withdrawal-letter/withdrawal-letter-hyalograft-c-autograft_en.pdf	**No orphan drugs**	5/14/13
Loulla,	mercaptopurine	Mercaptopurine	Only For Children Pharmaceuticals	Only For Children Pharmaceuticals	**Cytostatics**	**L01BB02**	**02 Neoplasms/**<br><br>**X Extension codes (Leukemias)**	ICD-11 Chapter 02<br><br>ICD-11 Chapter X	acute lymphoblastic leukemia (ALL) (maintenance therapy)	**Enzyme (Antagonism)(N1.A)**	**/N1.A<br>//**	Enzyme (Antagonism) (N1.A)	**/N1.A<br>//**	**Initial authorisation**		12/19/12	• open questions from the EMA (a17)<br>• concerns regarding the manufacturer's responses/information (a1)<br>• lack of evidence of superior efficacy compared with the authorised medicinal product protected by market exclusivity (a34)<br>• doubts about compliance with good clinical practice (gcp) (a30)	**/A17<br>/A1<br>/A34<br>/A30<br>//**	18/01/2013	**No authorisation, because of no positive benefit-risk ratio**	**EMA evaluation**	**Number of clinical studies 01**	**Participants unknown**	**placebo control unknown**		**Comparison with approved drugs unknown**	**No special drug approval procedure**	**No consequences for patients in ongoing clinical trials or compassionate use programmes**	Ongoing clinical trials (at the time the application was withdrawn)	https://www.ema.europa.eu/de/documents/medicine-qa/questions-and-answers-withdrawal-marketing-authorisation-application-loulla_de.pdf	https://www.ema.europa.eu/en/documents/withdrawal-letter/withdrawal-letter-loulla_en.pdf	**Orphan drugs**	1/18/13
Memantine FGK,	memantine	memantine	FGK Representative Service GmbH	FGK Representative Service GmbH	**Other medicines**	**N06DX01**	**06 Mental, behavioral or neurological developmental disorders**	ICD-11 Chapter 06	Alzheimer's disease (moderate to severe)	**Receptors (Antagonism) (N4.A)**	**/N4.A<br>//**	(Transferases, Antagonism)(N1.2.A)	**/N4.2.A<br>//**	**Initial authorisation**		12/18/12	• open questions from the EMA (a17)<br>• concerns regarding the manufacturer's responses/information (a1)<br>• doubts about efficacy<br>reason: poor study quality or flawed study design (lack of comparison with placebo treatment and/or comparator/reference medicinal products) (a27.1.4)<br>• missing justification of the dosing regimen (a9)	**/A17<br>/A1<br>/A27.1.4<br>/A9<br>//**	18/01/2013	**No authorisation, because of no positive benefit-risk ratio**	**Strategic reasons**	**Number of clinical studies 01**	**Participants 0500-0999**	**Placebo controlled clinical studies**	**SDH**	**Comparison with approved drugs unknown**	**Drug application for hybrid medicines**	**No consequences for patients in ongoing clinical trials or compassionate use programmes**	No ongoing clinical trials (at the time the application was withdrawn)	https://www.ema.europa.eu/en/documents/medicine-qa/questions-and-answers-withdrawal-marketing-authorisation-application-memantine-fgk_en.pdf	https://www.ema.europa.eu/en/documents/withdrawal-letter/withdrawal-letter-memantine-fgk_en.pdf	**No orphan drugs**	4/10/13
Jenzyl,	Ridaforolimus	Ridaforolimus	Merck Sharp and Dohme Ltd.	Merck Sharp & Dohme	**Immunosuppressants (no monoclonal antibodies)**	**L01EG03**	**02 Neoplasms**	ICD-11 Chapte	soft tissue or bone sarcoma (metastatic and already treated with chemotherapy)	**Enzyme (Antagonism)(N1.A)**	**/N1.A<br>//**	Enzyme (Transferases, Antagonism)(N1.2.A)	**/N1.2.A<br>//**	**Initial authorisation**		11/27/12	• open questions from the EMA (a17)<br>• concerns regarding the manufacturer's responses/information (a1)<br>• doubts about efficacy<br>reason: lack of data to adequately demonstrate efficacy (a27.3)<br>• doubts about ensuring patient safety<br>reason: adverse reactions (a21.1)	**/A17<br>/A1<br>/A27.3<br>/A21.1<br>//**	14/12/2012	**No authorisation, because of no positive benefit-risk ratio**	**EMA evaluation**	**Number of clinical studies 01**	**Participants 0500-0999**	**Placebo controlled clinical studies**		**No comparison with approved drugs**	**No special drug approval procedure**	**No consequences for patients in ongoing clinical trials or compassionate use programmes**	Ongoing clinical trials (at the time the application was withdrawn)	https://www.ema.europa.eu/en/documents/medicine-qa/questions-and-answers-withdrawal-marketing-authorisation-application-jenzyl-ridaforolimus_en.pdf	https://www.ema.europa.eu/en/documents/withdrawal-letter/withdrawal-letter-jenzyl-ridaforolimus_en.pdf	**No orphan drugs**	2/18/13
Solumarv,	insulin human	Insulin human	Marvel Life Sciences Ltd.	Marvel Life Sciences Ltd.	**Human insulin**	**A10AB01**	**05 Endocrine diseas**	ICD-11 Chapter 05	diabetes mellitus	**other (N6)**<br><br>**Receptors (Agonism) (N4.B)**	**/N4.B<br>/N6.U<br>//**	other (N6)<br><br>Receptors (catalytic receptors, Activation)(N4.3.B)	**/N6.U<br>/N4.3.B<br>//**	**Initial authorisation**		11/15/12	• open questions from the EMA (a17)<br>• concerns regarding the manufacturer's responses/information (a1)<br>• concerns regarding medicinal product quality (manufacturing process) (a3.1)<br>• doubts about same/improved efficacy compared with the eu reference medicinal product (a25)<br>• doubts about compliance with good clinical practice (gcp) (a30)<br>• doubts about efficacy<br>reason: poor study quality (data analysis) (a27.1.3)	**/A17<br>/A1<br>/A3.1<br>/A25<br>/A30<br>/A27.1.3<br>//**	14/12/2012	**No authorisation, because of no positive benefit-risk ratio**	**Other reasons**	**Number of clinical studies 01**	**Participants 0300-0499**	**No placebo control**	**SDB**	**Comparison with approved drugs**	**Drug application for biosimilars**	**No consequences for patients in ongoing clinical trials or compassionate use programmes**	No ongoing clinical trials (at the time the application was withdrawn)	https://www.ema.europa.eu/de/documents/medicine-qa/questions-and-answers-withdrawal-marketing-authorisation-application-solumarv-human-insulin_de.pdf	https://www.ema.europa.eu/en/documents/withdrawal-letter/withdrawal-letter-combimarv-isomarv-solumarv_en.pdf	**No orphan drugs**	12/14/12
Isomarv medium,	insulin human	Insulin human	Marvel Life Sciences Ltd.	Marvel Life Sciences Ltd.	**Human insulin**	**A10AB01**	**05 Endocrine diseas**	ICD-11 Chapter 05	diabetes mellitus	**other (N6)**<br><br>**Receptors (Agonism) (N4.B)**	**/N4.B<br>/N6.U<br>//**	other (N6)<br><br>Receptors (catalytic receptors, Activation)(N4.3.B)	**/N6.U<br>/N4.3.B<br>//**	**Initial authorisation**		11/15/12	• open questions from the EMA (a17)<br>• concerns regarding the manufacturer's responses/information (a1)<br>• concerns regarding medicinal product quality (manufacturing process) (a3.1)<br>• doubts about same/improved efficacy compared with the eu reference medicinal product (a25)<br>• doubts about compliance with good clinical practice (gcp) (a30)<br>• doubts about efficacy<br>reason: poor study quality (data analysis) (a27.1.3)	**/A17<br>/A1<br>/A3.1<br>/A25<br>/A30<br>/A27.1.3<br>//**	14/12/2012	**No authorisation, because of no positive benefit-risk ratio**	**Other reasons**	**Number of clinical studies 01**	**Participants 0300-0499**	**No placebo control**	**SDB**	**Comparison with approved drugs**	**Drug application for biosimilars**	**No consequences for patients in ongoing clinical trials or compassionate use programmes**	No ongoing clinical trials (at the time the application was withdrawn)	https://www.ema.europa.eu/en/documents/medicine-qa/questions-and-answers-withdrawal-marketing-authorisation-application-isomarv-human-insulin_en.pdf	https://www.ema.europa.eu/en/documents/withdrawal-letter/withdrawal-letter-combimarv-isomarv-solumarv_en.pdf	**No orphan drugs**	12/14/12

Combimarv,	insulin human	Insulin human	Marvel Life Sciences Ltd.	Marvel Life Sciences Ltd.	**Human insulin**	**A10AB01**	**05 Endocrine diseas**	ICD-11 Chapter 05	diabetes mellitus	**other (N6)**<br><br>**Receptors (Agonism) (N4.B)**	**/N4.B<br>/N6.U<br>//**	other (N6)<br><br>Receptors (catalytic receptors, Activation)(N4.3.B)	**/N6.U<br>/N4.3.B<br>//**	**Initial authorisation**		11/15/12	• open questions from the EMA (a17)<br>• concerns regarding the manufacturer's responses/information (a1)<br>• concerns regarding medicinal product quality (manufacturing process) (a3.1)<br>• doubts about same/improved efficacy compared with the eu reference medicinal product (a25)<br>• doubts about compliance with good clinical practice (gcp) (a30)<br>• doubts about efficacy<br>reason: poor study quality (data analysis) (a27.1.3)	**/A17<br>/A1<br>/A3.1<br>/A25<br>/A30<br>/A27.1.3<br>//**	14/12/2012	**No authorisation, because of no positive benefit-risk ratio**	**Other reasons**	**Number of clinical studies 01**	**Participants 0300-0499**	**No placebo control**	**SDB**	**Comparison with approved drugs**	**Drug application for biosimilars**	**No consequences for patients in ongoing clinical trials or compassionate use programmes**	No ongoing clinical trials (at the time the application was withdrawn)	https://www.ema.europa.eu/en/documents/medicine-qa/questions-and-answers-withdrawal-marketing-authorisation-application-combimarv-human-insulin_en.pdf	https://www.ema.europa.eu/en/documents/withdrawal-letter/withdrawal-letter-combimarv-isomarv-solumarv_en.pdf	**No orphan drugs**	12/14/12
Erbitux,	cetuximab	Cetuximab	Merck Europe B.V.	Merck Europe B.V.	**Monoclonal antibodies**	**L01FE01**	**02 Neoplasms**	ICD-11 Chapte	non-small cell lung carcinoma (NSCLC)<br><br>Note:<br>Use in previously untreated patients in combination with platinum-based chemotherapy<br><br>already approved indications:<br>squamous cell carcinomas in the head and neck area, metastatic colon or rectal carcinomas	**Receptors (Antagonism) (N4.A)**	**/N4.A<br>//**	Receptors (catalytic receptors, Antagonism) (N4.3.A)	**/N4.3.A<br>//**	**Post-authorisation**	6/29/04	9/17/12	• open questions from the EMA (a17)<br>• concerns regarding the manufacturer's responses/information (a1)<br>• doubts about efficacy<br>reason: flawed study design (retrospective analysis) (a27.2)<br>• doubts about benefit (clinical relevance of study results unclear, as other studies could not confirm positive effects) (a18)	**/A17<br>/A1<br>/A27.2<br>/A18<br>//**	12/10/2012	**No extension of authorisation, because of no positive benefit-risk ratio**	**EMA evaluation**	**Number of clinical studies 0**	**Participants 0**	**No placebo control**	**SD**	**No comparison with approved drugs**	**No special drug approval procedure**	**No consequences for patients in ongoing clinical trials or compassionate use programmes**	No ongoing clinical trials (at the time the application was withdrawn)	https://www.ema.europa.eu/en/documents/medicine-qa/questions-and-answers-withdrawal-application-change-marketing-authorisation-erbitux-cetuximab_en.pdf	https://www.ema.europa.eu/en/documents/other/withdrawal-letter-erbitux-cetuximab_en.pdf	**No orphan drugs**	11/22/12
SecreFlo,	secretin human	Secretin human	Repligen Europe Limited	Repligen Europe Limited	**Other medicines**	**V04CK01**	**X Extension codes (Medicines used in diagnostic tests, not elsewhere classified)**<br><br>**13 Diseases of the digestive system**	ICD-11 Chapter X<br><br>ICD-11 Chapter 13	magnetic resonance imaging in suspicion of pancreatitis (to improve image quality).<br><br>Anomalies of the pancreatic ducts should be more easily recognized this way.	**Receptors (Agonism) (N4.B)**	**/N4.B<br>//**	Receptors (GPCR, Agonism) (N4.1.B)	**/N4.1.B<br>//**	**Initial authorisation**		8/15/12	• open questions from the EMA (a17)<br>• concerns regarding the manufacturer's responses/information (a1)<br>• doubts about efficacy<br>reason: poor study quality (conduct) (a27.1.6)<br>• doubts about efficacy<br>reason: patients in the study not compatible with the target indication (a27.4)<br>• doubts about benefit (clinical relevance of the study results unclear) (a18)	**/A17<br>/A1<br>/A27.1.6<br>/A27.4<br>/A18<br>//**	24/09/2012	**No authorisation, because of no positive benefit-risk ratio**	**EMA evaluation**	**Number of clinical studies 01**	**Participants 0200-0299**	**No placebo control**		**Comparison with approved drugs**	**No special drug approval procedure**	**No consequences for patients in ongoing clinical trials or compassionate use programmes**	No ongoing clinical trials (at the time the application was withdrawn)	https://www.ema.europa.eu/en/documents/medicine-qa/questions-and-answers-withdrawal-marketing-authorisation-application-secreflo-secretin-human_en.pdf	https://www.ema.europa.eu/en/documents/withdrawal-letter/withdrawal-letter-secreflo-secretin-human_en.pdf	**No orphan drugs**	9/24/12
Velcade,	bortezomib	Bortezomib	Janssen-Cilag International NV	Janssen Pharmaceutical Companies of Johnson & Johnson	**Proteasome inhibitors**	**L01XG01**	**02 Neoplasms/**<br><br>**X Extension codes (malignant lymphomas, not otherwise classified)**	ICD-11 Chapter 02<br><br>ICD-11 Chapter X	follicular non-Hodgkin lymphoma (recurrences)<br><br>(treatment in combination with rituximab)<br><br>already approved indications:<br>multiple myeloma, mantle cell lymphoma<br><br>(treatment under certain conditions, partially in combination with other drugs for tumor therapy)	**Enzyme (Antagonism)(N1.A)**	**/N1.A<br>//**	Enzyme (Hydrolases, Antagonism)(N1.3.A)	**/N1.3.A<br>//**	**Post-authorisation**	4/26/04	7/2/12	• open questions from the EMA (a17)<br>• concerns regarding the manufacturer's responses/information (a1)<br>• doubts about benefit (clinical relevance of the study results) (a18)<br>• doubts about efficacy<br>reason: poor study quality (data analysis) (a27.1.3)<br>• doubts about ensuring patient safety<br>reason: adverse reactions (a21.1)	**/A17<br>/A1<br>/A18<br>/A27.1.3<br>/A21.1<br>//**	31/07/2012	**No extension of authorisation, because of no positive benefit-risk ratio**	**EMA evaluation**	**Number of clinical studies 01**	**Participants 0500-0999**	**No placebo control**		**Comparison with approved drugs**	**No special drug approval procedure**	**No consequences for patients in ongoing clinical trials or compassionate use programmes**	Ongoing clinical trials (at the time the application was withdrawn)	https://www.ema.europa.eu/en/documents/medicine-qa/questions-and-answers-withdrawal-application-change-marketing-authorisation-velcade-bortezomib_en.pdf	https://www.ema.europa.eu/en/documents/other/withdrawal-letter-velcade-bortezomib_en.pdf	**No orphan drugs**	8/30/12
Mulsevo,	semuloparin sodium	Semuloparin Sodium	Sanofi-Aventis	Sanofi	**Anticoagulants**	**Unknown**	**11 Diseases of the circulatory system**	ICD-11 Chapter 11	prevention of venous thromboembolic events<br><br>Note:<br>Use in cancer patients	**Ligands (agonism)(N9.B)**	**/N9.B<br>//**	Ligands (Agonism)(N9.B)	**/N9.B<br>//**	**Initial authorisation**		7/5/12	• open questions from the EMA (a17)<br>• concerns regarding the manufacturer's responses/information (a1)<br>• doubts about benefit (clinical relevance of the study results, as the risk of thrombotic events was only slightly reduced) (a18)<br>• doubts about efficacy<br>reason: poor study quality (selection of participants; study participants were not representative for european hiv patients) (a27.1.7)<br>• doubts about ensuring patient safety<br>reason: lack of data and/or inadequate long-term follow-up (a21.3)<br>• doubts about ensuring patient safety<br>reason: adverse reactions (tumor progression) (a21.1)	**/A17<br>/A1<br>/A27.1.7<br>/A18<br>/A21.3<br>/A21.1<br>//**	20/07/2012	**No authorisation, because of no positive benefit-risk ratio**	**EMA evaluation**	**Number of clinical studies 01**	**Participants over 2000**	**Placebo controlled clinical studies**		**No comparison with approved drugs**	**No special drug approval procedure**	**No consequences for patients in ongoing clinical trials or compassionate use programmes**	No ongoing clinical trials (at the time the application was withdrawn)	https://www.ema.europa.eu/en/documents/medicine-qa/questions-and-answers-withdrawal-marketing-authorisation-application-mulsevo-semuloparin-sodium_en.pdf	https://www.ema.europa.eu/en/documents/withdrawal-letter/withdrawal-letter-mulsevo-semuloparin-sodium_en.pdf	**No orphan drugs**	7/20/12
Egrifta,	tesamorelin	Tesamorelin	Ferrer Internacional S.A.	Ferrer Internacional S.A.	**Other medicines**	**H01AC06**	**14 Skin diseases**	ICD-11 Chapter 14	excess abdominal fat in HIV-infected patients with lipodystrophy	**Receptors (Agonism) (N4.B)**	**/N4.B<br>//**	Receptors (GPCR, Agonism) (N4.1.B)	**/N4.1.B<br>//**	**Initial authorisation**		6/21/12	• open questions from the EMA (a17)<br>• concerns regarding the manufacturer's responses/information (a1)<br>• doubts about efficacy<br>reason: poor study quality (selection of participants; study participants were not representative for european hiv patients) (a27.1.7)<br>• concerns regarding the requested indication (distinguishing between obesity and excess abdominal fat due to lipodystrophy in clinical practice difficult) (a28)<br>• doubts about benefit (doubt about the clinical relevance of the health benefit of treatment with egrifta) (a18)<br>• doubts about ensuring patient safety<br>reason: adverse reactions (igf-1 increase) (a21.1)<br>• doubts about ensuring patient safety<br>reason: lack of data and/or inadequate long-term follow-up (a21.3)	**/A17<br>/A1<br>/A27.1.7<br>/A28<br>/A18<br>/A21.1<br>/A21.3<br>//**	18/07/2012	**No authorisation, because of no positive benefit-risk ratio**	**EMA evaluation**	**Number of clinical studies 02**	**Participants 0500-0999**	**Placebo controlled clinical studies**		**No comparison with approved drugs**	**No special drug approval procedure**	**No consequences for patients in ongoing clinical trials or compassionate use programmes**	Ongoing clinical trials unknown (at the time the application was withdrawn)	https://www.ema.europa.eu/en/documents/medicine-qa/questions-and-answers-withdrawal-marketing-authorisation-application-egrifta-tesamorelin_en.pdf	https://www.ema.europa.eu/en/documents/withdrawal-letter/withdrawal-letter-egrifta-tesamorelin_en.pdf	**No orphan drugs**	7/18/12
Revlimid,	lenalidomide	Lenalidomide	Bristol-Myers Squibb Pharma EEIG	Bristol-Myers Squibb Pharma EEIG	**Immunomodulators**	**L04AX04**	**02 Neoplasms/**<br><br>**X Extension codes (Tumor of the plasma cells)**	ICD-11 Chapter 02<br><br>ICD-11 Chapter X	multiple myeloma (maintenance therapy)<br><br>already approved indications:<br>multiple myeloma (second-line therapy), myelodysplastic syndromes, mantle cell lymphoma and follicular lymphoma	**Enzyme (Regulation)(N1.A)**<br><br>**other proteins (Regulation)(N5.U)**<br><br>**Ligands (Regulation) (N9.U)**	**/N1.U<br>/N9.U<br>/N5.U<br>//**	Enzyme (Regulation)(N1)<br><br>Ligands (Regulation) (N9)<br><br>other proteins (Regulation)(N5)	**/N1.U<br>/N9.U<br>/N5.U<br>//**	**Post-authorisation**	6/14/07	6/20/12	• open questions from the EMA (a17)<br>• concerns regarding the manufacturer's responses/information (a1)<br>• doubts about ensuring patient safety<br>reason: adverse reactions (risks for second malignancies) (a21.1)<br>• doubts about efficacy<br>reason: prolonged overall benefit and symptom relief questionable (a27)<br>• doubts about ensuring patient safety<br>reason: lack of data and/or inadequate long-term follow-up (a21.3)	**/A17<br>/A1<br>/A21.1<br>/A27.<br>/A21.3<br>//**	6/07/2012	**No extension of authorisation, because of no positive benefit-risk ratio**	**EMA evaluation**	**Number of clinical studies 02**	**Participants 1000-1999**	**Placebo controlled clinical studies**		**Comparison with approved drugs**	**No special drug approval procedure**	**No consequences for patients in ongoing clinical trials or compassionate use programmes**	Ongoing clinical trials (at the time the application was withdrawn)	https://www.ema.europa.eu/en/documents/medicine-qa/questions-and-answers-withdrawal-application-change-marketing-authorisation-revlimid-lenalidomide_en.pdf		**Orphan drugs**	7/6/12
Megestrol Alkermes,	megestrol	Megestrol	Alkermes Pharma Ireland Ltd.	Alkermes Pharma Ireland Ltd.	**Progestogens**	**L02AB01**	**21 Symptoms, signs or clinical findings not elsewhere classified (unintentional weight loss)**	ICD-11 Chapter 21	weight and appetite loss in patients with cancer<br>or AIDS	**Receptors (Agonism) (N4.B)**	**/N4.B<br>//**	Receptors (nuclear receptors, Agonism) (N4.4.B)	**/N4.4.B<br>//**	**Initial authorisation**		3/6/12	• open questions from the EMA (a17)<br>• concerns regarding the manufacturer's responses/information (a1)<br>• doubts about same/improved efficacy (bioequivalence) compared with the eu reference medicinal product (a25)	**/A17<br>/A1<br>/A25<br>//**	20/03/2012	**No authorisation, because of no positive benefit-risk ratio**	**Marketing strategy**	**Number of clinical studies 01**	**Participants unknown**	**placebo control unknown**	**SDGH**	**Comparison with approved drugs**	**Drug application for hybrid medicines and generic medicines**	**No consequences for patients in ongoing clinical trials or compassionate use programmes**	No ongoing clinical trials (at the time the application was withdrawn)	Q-and-A on the withdrawal of the marketing authorisation application for Megestrol.Acetate.125mg. ml.Oral.Suspension (europa.eu)	https://www.ema.europa.eu/en/documents/withdrawal-letter/withdrawal-letter-megestrol-alkermes-megestrol_en.pdf	**No orphan drugs**	3/20/12
Fluad Paediatric,	unknown	Influenza vaccine, surface antigen, inactivated, adjuvanted with MF59C.1	Novartis Vaccines and Diagnostics S.r.l.	Novartis	**Other medicines**	**J07BB02**	**X Extension codes (viral vaccines)**	ICD-11 Chapter X	influenza<br><br>Note:<br>vaccine against influenza for infants and children aged 6 months to nine years	**other (N6)**	**/N6.U<br>//**	other (N6)	**/N6.U<br>//**	**Initial authorisation**		2/10/12	• open questions from the EMA (a17)<br>• concerns regarding the manufacturer's responses/information (a1)<br>• doubts about efficacy<br>reason: lack of data to adequately demonstrate efficacy (a27.3)<br>• doubts about efficacy<br>reason: poor study quality (inadequate laboratory tests) (a27.1)<br>• doubts about compliance with good clinical practice (gcp) (a30)	**/A17<br>/A1<br>/A27.3<br>/A27.1<br>/A30<br>//**	21/02/2012	**No authorisation, because of no positive benefit-risk ratio**	**EMA evaluation**	**Number of clinical studies 01**	**Participants over 2000**	**No placebo control**	**SD**	**Comparison with approved drugs**	**No special drug approval procedure**	**No consequences for patients in ongoing clinical trials or compassionate use programmes**	Ongoing clinical trials (at the time the application was withdrawn)	https://www.ema.europa.eu/en/documents/medicine-qa/questions-and-answers-withdrawal-marketing-authorisation-application-fluad-paediatric-influenza-vaccine-surface-antigen-inactivated-adjuvanted_en.pdf	https://www.ema.europa.eu/en/documents/withdrawal-letter/withdrawal-letter-fluad-paediatric_en.pdf	**No orphan drugs**	4/24/12
Pioglitazone ratiopharm GmbH,	pioglitazone	Pioglitazone	ratiopharm GmbH	ratiopharm GmbH	**Other antidiabetics**	**A10BG03**	**05 Endocrine diseases, nutritional or metabolic diseases**	ICD-11 Chapter 05	diabetes mellitus, type 2<br><br>Note:<br>Use particularly in patients with type 2 diabetes and overweight	**Receptors (Agonism) (N4.B)**	**/N4.B<br>//**	Receptors (nuclear receptors, Agonism) (N4.4.B)	**/N4.4.B<br>//**	**Initial authorisation**		2/3/12	• not applicable (marketing authorisation was recommended) (a14.2)	**/A14.2<br>//**	30/04/2012	**Authorisation recommended**	**Unknown**	**Number of clinical studies 0**	**Participants 0**	**No placebo control**	**SDG**	**Comparison with approved drugs**	**Drug application for generic medicines**	**No consequences for patients in ongoing clinical trials or compassionate use programmes**	No ongoing clinical trials (at the time the application was withdrawn)	https://www.ema.europa.eu/en/documents/medicine-qa/questions-and-answers-withdrawal-marketing-authorisation-application-pioglitazone-ratiopharm-gmbh_en.pdf	https://www.ema.europa.eu/en/documents/other/withdrawal-letter-pioglitazone-ratiopharm_en.pdf	**No orphan drugs**	4/30/12
Pioglitazone ratiopharm,	pioglitazone	Pioglitazone	ratiopharm GmbH	ratiopharm GmbH	**Other antidiabetics**	**A10BG03**	**05 Endocrine diseases, nutritional or metabolic diseases**	ICD-11 Chapter 05	diabetes mellitus, type 2	**Receptors (Agonism) (N4.B)**	**/N4.B<br>//**	Receptors (nuclear receptors, Agonism) (N4.4.B)	**/N4.4.B<br>//**	**Initial authorisation**		2/3/12	• not applicable (marketing authorisation was recommended) (a14.2)	**/A14.2<br>//**	30/04/2012	**Authorisation recommended**	**Unknown**	**Number of clinical studies 0**	**Participants 0**	**No placebo control**	**SDG**	**Comparison with approved drugs**	**Drug application for generic medicines**	**No consequences for patients in ongoing clinical trials or compassionate use programmes**	No ongoing clinical trials (at the time the application was withdrawn)	https://www.ema.europa.eu/en/documents/medicine-qa/questions-and-answers-withdrawal-marketing-authorisation-application-pioglitazone-ratiopharm_en.pdf	https://www.ema.europa.eu/en/documents/other/withdrawal-letter-pioglitazone-ratiopharm_en.pdf	**No orphan drugs**	4/30/12
Pioglitazone ratio,	pioglitazone	Pioglitazone	ratiopharm GmbH	ratiopharm GmbH	**Other antidiabetics**	**A10BG03**	**05 Endocrine diseases, nutritional or metabolic diseases**	ICD-11 Chapter 05	diabetes mellitus, type 2<br><br>Note:<br>Use particularly in patients with type 2 diabetes and overweight	**Receptors (Agonism) (N4.B)**	**/N4.B<br>//**	Receptors (nuclear receptors, Agonism) (N4.4.B)	**/N4.4.B<br>//**	**Initial authorisation**		2/3/12	• not applicable (marketing authorisation was recommended) (a14.2)	**/A14.2<br>//**	30/04/2012	**Authorisation recommended**	**Unknown**	**Number of clinical studies 0**	**Participants 0**	**No placebo control**	**SDG**	**Comparison with approved drugs**	**Drug application for generic medicines**	**No consequences for patients in ongoing clinical trials or compassionate use programmes**	No ongoing clinical trials (at the time the application was withdrawn)	https://www.ema.europa.eu/en/documents/medicine-qa/questions-and-answers-withdrawal-marketing-authorisation-application-pioglitazone-ratio_en.pdf	https://www.ema.europa.eu/en/documents/other/withdrawal-letter-pioglitazone-ratiopharm_en.pdf	**No orphan drugs**	4/30/12

Prometax,	rivastigmine	Rivastigmine	Novartis Europharm Limited	Novartis	**Other medicines**	**N06DA03**	**06 Mental, behavioral or neurological developmental disorders**	ICD-11 Chapter 06	Parkinson's disease with mild to moderate dementia (use as a transdermal patch)<br><br>already approved indications: Alzheimer's dementia (mild to moderate), dementia in Parkinson's disease (mild to moderate)	**Enzyme (Antagonism)(N1.A)**	**/N1.A //**	**Enzyme (Hydrolases, Antagonism)(N1.3.A)**	**/N1.3.A //**	**Post-authorisation**	12/4/98	3/14/12	• open questions from the EMA (a17)<br>• concerns regarding the manufacturer's responses/information (a1)<br>• doubts about efficacy<br>reason: lack of data to adequately demonstrate efficacy (a27.3)<br>• doubts about efficacy<br>reason: flawed study design (a27.2)	**/A17 /A1 /A27.3 /A27.2 //**	19/04/2012	**No extension of authorisation, because of no positive benefit-risk ratio**	**EMA evaluation**	**Number of clinical studies 01**	**Participants 0500-0999**	**No placebo control**	**SD**	**Comparison with approved drugs**	**No special drug approval procedure**	**No consequences for patients in ongoing clinical trials or compassionate use programmes**	Ongoing clinical trials (at the time the application was withdrawn)	https://www.ema.europa.eu/de/documents/medicine-qa/questions-and-answers-withdrawal-application-change-marketing-authorisation-exelon-and-prometax-rivastigmine_de.pdf	https://www.ema.europa.eu/en/documents/other/withdrawal-letter-exelon-and-prometax_en.pdf	**No orphan drugs**	4/19/12
Exelon,	rivastigmine	Rivastigmine	Novartis Europharm Limited	Novartis	**Other medicines**	**N06DA03**	**06 Mental, behavioral or neurological developmental disorders**	ICD-11 Chapter 06	Parkinson's disease with mild to moderate dementia (use as a transdermal patch)<br><br>already approved indications: Alzheimer's dementia (mild to moderate), dementia in Parkinson's disease (mild to moderate)	**Enzyme (Antagonism)(N1.A)**	**/N1.A //**	**Enzyme (Hydrolases, Antagonism)(N1.3.A)**	**/N1.3.A //**	**Post-authorisation**	12/4/98	3/14/12	• open questions from the EMA (a17)<br>• concerns regarding the manufacturer's responses/information (a1)<br>• doubts about efficacy<br>reason: lack of data to adequately demonstrate efficacy (a27.3)<br>• doubts about efficacy<br>reason: flawed study design (a27.2)	**/A17 /A1 /A27.3 /A27.2 //**	19/04/2012	**No extension of authorisation, because of no positive benefit-risk ratio**	**EMA evaluation**	**Number of clinical studies 01**	**Participants 0500-0999**	**No placebo control**	**SD**	**Comparison with approved drugs**	**No special drug approval procedure**	**No consequences for patients in ongoing clinical trials or compassionate use programmes**	Ongoing clinical trials (at the time the application was withdrawn)	https://www.ema.europa.eu/en/documents/medicine-qa/questions-and-answers-withdrawal-application-change-marketing-authorisation-exelon-and-prometax-rivastigmine_en.pdf	https://www.ema.europa.eu/en/documents/other/withdrawal-letter-exelon-and-prometax_en.pdf	**No orphan drugs**	4/19/12
Qutenza,	capsaicin	Capsaicin	Grunenthal GmbH	Grunenthal GmbH	**Other medicines**	**N01BX04**	**08 Diseases of the nervous system**	ICD-11 Chapter 08	peripheral neuropathic pain in adults with diabetes (when neuropathic pain is not caused by diabetes)<br><br>already approved indications: peripheral neuropathic pain in adults (without diabetes)	**Ion channels (Activation) (N2.B)**	**/N2.B //**	Ion channels (Activation) (N2.B)	**/N2.B //**	**Post-authorisation**	5/15/09	3/14/12	• open questions from the EMA (a17)<br>• concerns regarding the manufacturer's responses/information (a1)<br>• concerns regarding the requested indication (distinguishing between non-diabetic neuropathic pain and diabetic neuropathic pain in clinical practice difficult) (a28)<br>• doubts about ensuring patient safety<br>reason: adverse reactions (risks for ulcers and complications) (a21.1)	**/A17 /A1 /A28 /A21.1 //**	16/04/2012	**No extension of authorisation, because of no positive benefit-risk ratio**	**EMA evaluation**	**Number of clinical studies 0**	**Participants 0**	**No placebo control**	**SD**	**No comparison with approved drugs**	**No special drug approval procedure**	**No consequences for patients in ongoing clinical trials or compassionate use programmes**	Ongoing clinical trials (at the time the application was withdrawn)	https://www.ema.europa.eu/en/documents/medicine-qa/questions-and-answers-withdrawal-application-change-marketing-authorisation-qutenza-capsaicin_en.pdf	https://www.ema.europa.eu/en/documents/other/withdrawal-letter-qutenza_en.pdf	**No orphan drugs**	4/16/12
Tyverb,	lapatinib	Lapatinib	Novartis Europharm Limited	Novartis	**Kinase inhibitors**	**L01EH01**	**02 Neoplasms**	ICD-11 Chapte	use also in combination with paclitaxel in metastatic breast cancer (with HER2 overexpression)<br><br>already approved indications: treatment of breast cancers (advanced or metastatic with HER2 overexpression) in combination with capecitabine or an aromatase inhibitor	**Receptors (Antagonism) (N4.A)**	**/N4.A //**	Receptors (catalytic receptors, Antagonism) (N4.3.A)	**/N4.3.A //**	**Post-authorisation**	6/10/08	2/15/12	• open questions from the EMA (a17)<br>• concerns regarding the manufacturer's responses/information (a1)<br>• doubts about efficacy<br>reason: poor study quality or flawed study design (lack of comparison with placebo treatment and/or comparator/reference medicinal products) (a27.1.4)	**/A17 /A1 /A27.1.4 //**	16/03/2012	**No extension of authorisation, because of no positive benefit-risk ratio**	**EMA evaluation**	**Number of clinical studies 01**	**Participants 0300-0499**	**Placebo controlled clinical studies**		**Comparison with approved drugs**	**No special drug approval procedure**	**No consequences for patients in ongoing clinical trials or compassionate use programmes**	Ongoing clinical trials (at the time the application was withdrawn)	https://www.ema.europa.eu/en/documents/medicine-qa/questions-and-answers-withdrawal-application-change-marketing-authorisation-tyverb-lapatinib_en.pdf	https://www.ema.europa.eu/en/documents/other/tyverb-withdrawal-letter_en.pdf	**No orphan drugs**	3/16/12
Pioglitazone Teva Generics,	pioglitazone	Pioglitazone Hydrochloride	Teva Pharma B.V	Teva Pharma B.V	**Other antidiabetics**	**A10BG03**	**05 Endocrine diseases, nutritional or metabolic diseases**	ICD-11 Chapter 05	diabetes mellitus, type 2<br><br>Note:<br>Use particularly in patients with type 2 diabetes and overweight	**Receptors (Agonism) (N4.B)**	**/N4.B //**	Receptors (nuclear receptors, Agonism) (N4.4.B)	**/N4.4.B //**	**Initial authorisation**		1/26/12	• not applicable (marketing authorisation was recommended) (a14.2)	**/A14.2 //**	30/04/2012	**Authorisation recommended**	**Unknown**	**Number of clinical studies 0**	**Participants 0**	**No placebo control**	**SDG**	**Comparison with approved drugs**	**Drug application for generic medicines**	**No consequences for patients in ongoing clinical trials or compassionate use programmes**	No ongoing clinical trials (at the time the application was withdrawn)	https://www.ema.europa.eu/en/documents/medicine-qa/questions-and-answers-withdrawal-marketing-authorisation-application-pioglitazone-teva-generics_en.pdf		**No orphan drugs**	7/3/12
Kalbitor,	ecallantide	Ecallantide	Dyax s.a.	Dyax s.a.	**Other medicines**	**B06AC03**	**04 Diseases of the immune system**	ICD-11 Chapter 04	hereditary angioedemas (acute attacks)	**Enzyme (Antagonism)(N1.A)**	**/N1.A //**	Enzyme (Hydrolases, Antagonism)(N1.3.A)	**/N1.3.A //**	**Initial authorisation**		11/11/11	• open questions from the EMA (a17)<br>• concerns regarding the manufacturer's responses/information (a1)<br>• doubts about ensuring patient safety<br>reason: adverse reactions (hypersensitivity reaction) (a21.1)<br>• concerns regarding recommended dose (relating to efficacy of the recommended dose in severe seizures) (a6)	**/A17 /A1 /A21.1 /A6 //**	23/11/2011	**No authorisation, because of no positive benefit-risk ratio**	**EMA evaluation**	**Number of clinical studies 02**	**Participants 0150-0199**	**Placebo controlled clinical studies**		**No comparison with approved drugs**	**No special drug approval procedure**	**No consequences for patients in ongoing clinical trials or compassionate use programmes**	Ongoing clinical trials (at the time the application was withdrawn)	https://www.ema.europa.eu/en/documents/medicine-qa/questions-and-answers-withdrawal-marketing-authorisation-application-kalbitor_en.pdf	https://www.ema.europa.eu/en/documents/withdrawal-letter/withdrawal-letter-kalbitor_en.pdf	**Orphan drugs**	11/23/11
Janacti,	sitagliptin/ pioglitazone	Sitagliptin/ Pioglitazone	Merck Sharp and Dohme Ltd.	Merck Sharp & Dohme	**Other antidiabetics**	**A10BD12**	**05 Endocrine diseases, nutritional or metabolic diseases**	ICD-11 Chapter 05	diabetes mellitus, type 2	**Enzyme (Antagonism)(N1.A)**<br><br>**Receptors (Agonism)(N4.B)**	**/N1.A /N4.B //**	Enzyme (Hydrolases, Antagonism)(N1.3.A)<br><br>Receptors (nuclear receptors, Agonism) (N4.4.B)	**/N1.3.A /N4.4.B //**	**Initial authorisation**		11/7/11	• not applicable (documents have not yet been assessed or recommendations not yet published) (a14.1)<br><br>note:<br>documents had not been fully assessed at the time of withdrawal of the application.	**/A14.1 //**	22/11/2011	**None (documents have not yet been evaluated)**	**Marketing strategy**	**Number of clinical studies unknown**	**Participants unknown**	**placebo control unknown**	**SD**	**Comparison with approved drugs unknown**	**No special drug approval procedure**	**No consequences for patients in ongoing clinical trials or compassionate use programmes**	No ongoing clinical trials (at the time the application was withdrawn)	https://www.ema.europa.eu/en/documents/medicine-qa/questions-and-answers-withdrawal-marketing-authorisation-application-janacti-and-related-names_en.pdf	https://www.ema.europa.eu/en/documents/withdrawal-letter/withdrawal-letter-janacti_en.pdf	**No orphan drugs**	11/22/11
Desloratadine Krka,	desloratadine	Desloratadine	Krka, d.d., Novo mesto	Krka, d.d., Novo mesto	**Antihistamines**	**R06AX27**	**12 Respiratory diseases/**<br><br>**14 Skin diseases**	ICD-11 Chapter 12<br><br>ICD-11 Chapter 14	allergic rhinitis, urticaria	**Receptors (Antagonism) (N4.A)**	**/N4.A //**	Receptors (GPCR, Antagonism) (N4.1.A)	**/N4.1.A //**	**Initial authorisation**		10/14/11	• not applicable (marketing authorisation was recommended) (a14.2)	**/A14.2 //**	17/11/2011	**Authorisation recommended**	**Marketing strategy**	**Number of clinical studies 0**	**Participants 0**	**No placebo control**	**SDG**	**Comparison with approved drugs**	**Drug application for generic medicines**	**No consequences for patients in ongoing clinical trials or compassionate use programmes**	No ongoing clinical trials (at the time the application was withdrawn)	https://www.ema.europa.eu/en/documents/medicine-qa/questions-and-answers-withdrawal-marketing-authorisation-application-desloratadine-krka_en.pdf	https://www.ema.europa.eu/en/documents/withdrawal-letter/withdrawal-letter-desloratadine-krka_en.pdf	**No orphan drugs**	11/17/11
Luveniq	Voclosporin	Voclosporin	Lux Biosciences GmbH	Lux Biosciences GmbH	**Immunosuppressants (no monoclonal antibodies)**	**L04AD03**	**09 Diseases of the visual system**	ICD-11 Chapter 09	uveitis (chronic, active, non-infectious)	**Enzyme (Antagonism)(N1.A)**	**/N1.A //**	Enzyme (Isomerases, Antagonism)(N1.5.A)	**/N1.5.A //**	**Initial authorisation**		10/13/11	• concerns regarding the manufacturer's responses/information (a1)<br>• doubts about ensuring patient safety<br>reason: adverse reactions (a21.1)<br>• doubts about efficacy<br>reason: lack of data to adequately demonstrate efficacy (no difference compared with placebo) (a27.3)	**/A1 /A21.1 /A27.3 //**	24/10/2011	**No authorisation, because of no positive benefit-risk ratio**	**EMA evaluation**	**Number of clinical studies 01**	**Participants 0200-0299**	**Placebo controlled clinical studies**		**No comparison with approved drugs**	**No special drug approval procedure**	**No consequences for patients in ongoing clinical trials or compassionate use programmes**	Ongoing clinical trials (at the time the application was withdrawn)	https://www.ema.europa.eu/en/documents/medicine-qa/questions-and-answers-withdrawal-marketing-authorisation-application-luveniq_en.pdf	https://www.ema.europa.eu/en/documents/withdrawal-letter/withdrawal-letter-luveniq_en.pdf	**Orphan drugs**	10/24/11
Ibandronic Acid Hexal,	ibandronic acid	Ibandronic acid	Hexal AG	Hexal AG	**Bisphosphonates**	**M05BA06**	**15 Diseases of the musculoskeletal system or connective tissue**	ICD-11 Chapter 15	prevention of skeletal events (fractures, bone complications) in patients with breast cancer and bone metastases	**Enzyme (Antagonism)(N1.A)**	**/N1.A //**	Enzyme (Transferases, Antagonism)(N1.2.A)	**/N1.2.A //**	**Initial authorisation**		7/21/11	• not applicable (marketing authorisation was recommended) (a14.2)	**/A14.2 //**	22/08/2011	**Authorisation recommended**	**Marketing strategy**	**Number of clinical studies 0**	**Participants 0**	**No placebo control**	**SDG**	**Comparison with approved drugs**	**Drug application for generic medicines**	**No consequences for patients in ongoing clinical trials or compassionate use programmes**	No ongoing clinical trials (at the time the application was withdrawn)	https://www.ema.europa.eu/en/documents/medicine-qa/questions-and-answers-withdrawal-marketing-authorisation-application-ibandronic-acid-hexal_en.pdf	https://www.ema.europa.eu/en/documents/withdrawal-letter/withdrawal-letter-ibandronic-acid-hexal_en.pdf	**No orphan drugs**	8/22/11
Doxorubicin SUN,	doxorubicin	Doxorubicin hydrochloride	Sun Pharmaceutical Industries Europe B.V.	Sun Pharmaceutical Industries	**Cytostatics**	**L01DB01**	**02 Neoplasms**	ICD-11 Chapter 02	metastatic breast cancer (in patients with cardiac risks), advanced ovarian cancer (under certain conditions), multiple myeloma (under certain conditions)	**Enzyme (Antagonism)(N1.A)**	**/N1.A //**	Enzyme (Isomerases, Antagonism) (N1.5.A)	**/N1.5.A //**	**Initial authorisation**		7/20/11	• open questions from the EMA (a17)<br>• concerns regarding the manufacturer's responses/information (a1)<br>• doubts about same/improved efficacy compared with the eu reference medicinal product (similarity not sufficiently demonstrated) (a25)	**/A17 /A1 /A25 //**	13/09/2011	**No authorisation, because of no positive benefit-risk ratio**	**EMA evaluation**	**Number of clinical studies 0**	**Participants 0**	**No placebo control**	**SDGH**	**Comparison with approved drugs**	**Drug application for hybrid medicines and generic medicines**	**No consequences for patients in ongoing clinical trials or compassionate use programmes**	No ongoing clinical trials (at the time the application was withdrawn)	https://www.ema.europa.eu/en/documents/medicine-qa/withdrawal-marketing-authorisation-application-doxorubicin-sun_en.pdf	https://www.ema.europa.eu/en/documents/withdrawal-letter/withdrawal-letter-doxorubicin-sun_en.pdf	**No orphan drugs**	9/26/11
Macugen,	pegaptanib	Pegaptanib	PharmaSwiss Ceska Republika s.r.o	PharmaSwiss Ceska Republika s.r.o	**Kinase inhibitors**	**S01LA03**	**09 Diseases of the visual system**	**ICD-11 Chapter 09**	visual disturbances in patients with diabetic macular edema (DME)<br><br>already approved indications: w et age-related macular degeneration (AMD)	**Ligands (antagonism)(N9.A)**	**/N9.A //**	Ligands (Vascular endothelial growth factor (VEGF), Antagonism) (N9.1.A)	**/N9.1.A //**	**Post-authorisation**	1/31/06	7/15/11	• concerns regarding the manufacturer's responses/information (a1)<br>• doubts about efficacy<br>reason: lack of data to adequately demonstrate efficacy (in particular efficacy in outcomes after two years of treatment compared with placebo treatment questionable) (a27.3)	**/A1 /A27.3 //**	27/07/2011	**No extension of authorisation, because of no positive benefit-risk ratio**	**EMA evaluation**	**Number of clinical studies 01**	**Participants 0300-0499**	**Placebo controlled clinical studies**	**SD**	**No comparison with approved drugs**	**No special drug approval procedure**	**No consequences for patients in ongoing clinical trials or compassionate use programmes**	Ongoing clinical trials (at the time the application was withdrawn)	https://www.ema.europa.eu/en/documents/medicine-qa/questions-and-answers-withdrawal-application-change-marketing-authorisation-macugen-pegaptanib_en.pdf	https://www.ema.europa.eu/en/documents/other/macugen-withdrawal-letter_en.pdf	**No orphan drugs**	7/27/11
Beprana,	naproxcinod	Naproxcinod	NicOx S.A.	NicOx S.A.	**Non-steroidal anti-inflammatory drugs (NSAIDs)**	**M01AE18**	**15 Diseases of the musculoskeletal system or connective tissue**	ICD-11 Chapter 15	osteoarthritis (osteoarthritis) of the knee and hip in adults (use to relieve signs and symptoms of osteoarthritis)	**Enzyme (Antagonism)(N1.A)**	**/N1.A //**	Enzyme (Oxidoreductases, Antagonism)(N1.1.A)	**/N1.1.A //**	**Initial authorisation**		4/19/11	• open questions from the EMA (a17)<br>• concerns regarding the manufacturer's responses/information (a1)<br>• doubts about ensuring patient safety<br>reason: adverse reactions (risk of hepatotoxicity, effect on blood pressure) (a21.1)	**/A17 /A1 /A21.1 //**	27/05/2011	**No authorisation, because of no positive benefit-risk ratio**	**EMA evaluation**	**Number of clinical studies 03**	**Participants over 2000**	**Placebo controlled clinical studies**		**Comparison with approved drugs**	**No special drug approval procedure**	**No consequences for patients in ongoing clinical trials or compassionate use programmes**	Ongoing clinical trials (at the time the application was withdrawn)	https://www.ema.europa.eu/en/documents/medicine-qa/questions-and-answers-withdrawal-marketing-authorisation-application-beprana-naproxcinod_en.pdf	https://www.ema.europa.eu/en/documents/withdrawal-letter/withdrawal-letter-beprana_en.pdf	**No orphan drugs**	5/27/11
Joicela,	lumiracoxib	Lumiracoxib	Novartis	Novartis	**Non-steroidal anti-inflammatory drugs (NSAIDs)**	**M01AH06**	**15 Diseases of the musculoskeletal system or connective tissue**	ICD-11 Chapter 15	osteoarthritis (relief of symptoms of osteoarthritis, such as swelling and pain in the joints of the knee and hip, in adults who do not carry the gene variant DQA1*0102)<br><br>Note:<br>DQA1*0102 presumably increases the risk of liver toxicity in patients taking Joicela.	**Enzyme (Antagonism)(N1.A)**	**/N1.A //**	Enzyme (Oxidoreductases, Antagonism)(N1.1.A)	**/N1.1.A //**	**Initial authorisation**		4/15/11	• open questions from the EMA (a17)<br>• concerns regarding the manufacturer's responses/information (a1)<br>• doubts about ensuring patient safety<br>reason: adverse reactions (risk of hepatotoxicity) (a21.1)	**/A17 /A1 /A21.1 //**	27/05/2011	**No authorisation, because of no positive benefit-risk ratio**	**EMA evaluation**	**Number of clinical studies 03**	**Participants over 2000**	**Placebo controlled clinical studies**		**Comparison with approved drugs**	**No special drug approval procedure**	**No consequences for patients in ongoing clinical trials or compassionate use programmes**	No ongoing clinical trials (at the time the application was withdrawn)	https://www.ema.europa.eu/en/documents/medicine-qa/questions-and-answers-withdrawal-marketing-authorisation-application-joicela-lumiracoxib_en.pdf	https://www.ema.europa.eu/en/documents/withdrawal-letter/withdrawal-letter-joicela_en.pdf	**No orphan drugs**	5/27/11
Epostim,	epoetin alfa	Epoetin Alpha	Reliance GeneMedix Plc	Reliance GeneMedix Plc	**Other medicines**	**B03XA01**	**03 Diseases of the blood or blood-forming organs**<br><br>**23 External causes of morbidity or mortality**	ICD-11 Chapter 03<br><br>ICD-11 Chapter 23	anemia (in chronic kidney insufficiency, in treatment with chemotherapy, to increase blood volume (autologous blood donation) and reduce the need for blood transfusions before a major orthopedic surgery)	**Receptors (Agonism) (N4.B)**	**/N4.B //**	Receptors (catalytic receptors, Agonism) (N4.3.B)	**/N4.3.B //**	**Initial authorisation**		3/15/11	• not applicable (documents have not yet been assessed or recommendations not yet published) (a14.1)<br><br>note:<br>documents had not been fully assessed at the time of withdrawal of the application.	**/A14.1 //**	19/04/2011	**None (documents have not yet been evaluated)**	**EMA evaluation**	**Number of clinical studies 02**	**Participants 0200-0299**	**No placebo control**	**SDB**	**Comparison with approved drugs**	**Drug application for biosimilars**	**No consequences for patients in ongoing clinical trials or compassionate use programmes**	Ongoing clinical trials (at the time the application was withdrawn)	https://www.ema.europa.eu/en/documents/medicine-qa/questions-and-answers-withdrawal-marketing-authorisation-application-epostim-epoetin-alfa_en.pdf	https://www.ema.europa.eu/en/documents/withdrawal-letter/withdrawal-letter-epostim_en.pdf	**No orphan drugs**	4/19/11
Movectro,	cladribine	Cladribine	Merck Serono Europe Limited	Merck Europe B.V.	**Cytostatics**	**L01BB04**	**08 Diseases of the nervous system**	ICD-11 Chapter 08	multiple sclerosis, relapsing-remitting<br><br>Note:<br>Use under certain conditions	**Enzyme (Antagonism)(N1.A)**	**/N1.A //**	Enzyme (Antagonism) (N1.A)	**/N1.A //**	**Initial authorisation**		2/8/11	• concerns regarding the manufacturer's responses/information (a1)<br>• doubts about ensuring patient safety (a21)	**/A1 /A21. //**	23/02/2011	**No authorisation, because of no positive benefit-risk ratio**	**EMA evaluation**	**Number of clinical studies 01**	**Participants 1000-1999**	**Placebo controlled clinical studies**		**No comparison with approved drugs**	**No special drug approval procedure**	**No consequences for patients in ongoing clinical trials or compassionate use programmes**	Ongoing clinical trials (at the time the application was withdrawn)	https://www.ema.europa.eu/en/documents/smop-initial/questions-and-answers-recommendation-refusal-marketing-authorisation-application-movectro-outcome-re-examination_en.pdf	https://www.ema.europa.eu/en/documents/withdrawal-letter/movectro-withdrawal-letter_en.pdf	**No orphan drugs**	2/23/11

Ozespa ,	unknown	Briakinumab	Abbott Laboratories Limited	Abbott Laboratories Limited	**Monoclonal antibodies**	**L04AC09**	**14 Skin diseases**	ICD-11 Chapter 14	plaque psoriasis (moderate to severe, in adults who have not responded to or cannot tolerate other systemic treatments for psoriasis, including ciclosporin, methotrexate, and PUVA)	**Ligands (antagonism)(N9.A)**	**/N9.A<br>//**	Ligands (Cytokines, Antagonism)(N9.2.A)	**/N9.2.A<br>//**	**Initial authorisation**		1/14/11	• not applicable (documents have not yet been assessed or recommendations not yet published) (a14.1)<br><br>note:<br>documents had not been fully assessed at the time of withdrawal of the application.	**/A14.1<br>//**	31/01/2011	**None (documents have not yet been evaluated)**	**Suspected EMA evaluation**	**Number of clinical studies 04**	**Participants over 2000**	**Placebo controlled clinical studies**		**Comparison with approved drugs**	**No special drug approval procedure**	**No consequences for patients in ongoing clinical trials or compassionate use programmes**	Ongoing clinical trials (at the time the application was withdrawn)	https://www.ema.europa.eu/en/documents/medicine-qa/questions-and-answers-withdrawal-marketing-authorisation-application-ozespa_en.pdf	https://www.ema.europa.eu/en/documents/withdrawal-letter/ozespa-withdrawal-letter_en.pdf	**No orphan drugs**	1/31/11
Tekinex,	unknown	Omacetaxin mepesuccinate	ChemGenex Europe SAS	ChemGenex Europe SAS	**Other medicines**	**L01XX40**	**02 Neoplasms**	ICD-11 Chapte	chronic myeloid leukemia (CML), Philadelphia chromosome-positive (Ph+) (use when a previous treatment with imatinib has failed, e.g. with T315I mutation in the Bcr-Abl kinase gene)	**other proteins (N5.U)**	**/N5.U<br>//**	other proteins (N5)	**/N5.U<br>//**	**Initial authorisation**		1/11/11	• open questions from the EMA (a17)<br>• concerns regarding the manufacturer's responses/information (a1)<br>• doubts about ensuring patient safety<br>reason: lack of data and/or inadequate long-term follow-up (a21.3)<br>• doubts about benefit (a18)<br>• doubts about efficacy<br>reason: poor study quality (conduct) (a27.1.6)	**/A17<br>/A1<br>/A21.3<br>/A18<br>/A27.1.6<br>//**	31/01/2011	**No authorisation, because of no positive benefit-risk ratio**	**EMA evaluation**	**Number of clinical studies 01**	**Participants 0050-0074**	**No placebo control**		**No comparison with approved drugs**	**No special drug approval procedure**	**No consequences for patients in ongoing clinical trials or compassionate use programmes**	Ongoing clinical trials (at the time the application was withdrawn)	https://www.ema.europa.eu/en/documents/medicine-qa/questions-and-answers-withdrawal-marketing-authorisation-application-tekinex_en.pdf	https://www.ema.europa.eu/en/documents/withdrawal-letter/tekinex-withdrawal-letter_en.pdf	**Orphan drugs**	1/31/11
Topotecan SUN,	topotecan	Topotecan	Sun Pharmaceutical Industries Europe B.V.	Sun Pharmaceutical Industries	**Cytostatics**	**L01CE01**	**02 Neoplasms**	ICD-11 Chapte	ovarian cancer (metastatic with failed primary therapy), small cell lung cancer (recurrence), use also in combination with cisplatin (when tumor disease has recurred after radiotherapy or is very advanced (stage IVb))	**Enzyme (Antagonism)(N1.A)**	**/N1.A<br>//**	Enzyme (Isomerases, Antagonism) (N1.5.A)	**/N1.5.A<br>//**	**Initial authorisation**		1/3/11	• not applicable (documents have not yet been assessed or recommendations not yet published) (a14.1)<br><br>note:<br>documents had not been fully assessed at the time of withdrawal of the application.	**/A14.1<br>//**	23/02/2011	**None (documents have not yet been evaluated)**	**Marketing strategy**	**Number of clinical studies 0**	**Participants 0**	**No placebo control**	**SDG**	**Comparison with approved drugs**	**Drug application for generic medicines**	**Consequences for patients in ongoing clinical trials or compassionate use programmes unknown**	Ongoing clinical trials unknown (at the time the application was withdrawn)	https://www.ema.europa.eu/en/documents/medicine-qa/questions-and-answers-withdrawal-marketing-authorisation-application-topotecan-sun_en.pdf	https://www.ema.europa.eu/en/documents/withdrawal-letter/topotecan-sun-withdrawal-letter_en.pdf	**No orphan drugs**	2/23/11
Emerflu,	unknown	pandemic influenza vaccine (H5N1) (split virus, inactivated, adjuvanted)	Sanofi Pasteur S.A.	Sanofi	**Other medicines**	**J07BB**	**X Extension codes (viral vaccines)**	ICD-11 Chapter X	influenza<br><br>Note:<br>Use in adults for protection against "pandemic" influenza (use only if influenza pandemic has been officially declared)	**other (N6)**	**/N6.U<br>//**	other (N6)	**/N6.U<br>//**	**Initial authorisation**		12/1/10	• concerns regarding the manufacturer's responses/information (a1)<br>• doubts about efficacy<br>reason: other reasons (concerns about adequate production of antibodies) (a27)<br>• doubts about efficacy<br>reason: other reasons (immunogenicity not sufficient) (a27)	**/A1<br>/A27.<br>//**	20/12/2010	**No authorisation, because of no positive benefit-risk ratio**	**EMA evaluation**	**Number of clinical studies 02**	**Participants 0500-0999**	**No placebo control**		**No comparison with approved drugs**	**No special drug approval procedure**	**No consequences for patients in ongoing clinical trials or compassionate use programmes**	No ongoing clinical trials (at the time the application was withdrawn)	https://www.ema.europa.eu/en/documents/medicine-qa/questions-and-answers-withdrawal-marketing-authorisation-application-emerflu-pandemic-influenza-vaccine-h5n1-split-virion-inactivated-adjuvanted_en.pdf	https://www.ema.europa.eu/en/documents/withdrawal-letter/withdrawal-letter-emerflu_en.pdf	**No orphan drugs**	12/20/10
Zenhale,	unknown	Mometasone furoate/Formoterol fumarate	Schering-Plough Europe	Schering-Plough Europe	**Glucocorticoids (Corticosteroids)**<br><br>**Beta-2 sympathomimetics**	**R03AK07**	**12 Respiratory diseases**	ICD-11 Chapter 12	asthma (maintenance treatment, reduction of exacerbations, use under certain conditions)	**Receptors (Agonism) (N4.B)**	**/N4.B<br>//**	Receptors (GPCR, Agonism) (N4.1.B)	**/N4.1.B<br>//**	**Initial authorisation**		11/5/10	• open questions from the EMA (a17)<br>• concerns regarding the manufacturer's responses/information (a1)<br>• doubts about efficacy<br>reason: poor study quality (conduct) (a27.1.6)<br>• doubts about efficacy<br>reason: other reasons (concerns regarding the use and formulation of the comparator product mometasone furoate) (a27)	**/A17<br>/A1<br>/A27.1.6<br>/A27.<br>//**	25/11/2010	**No authorisation, because of no positive benefit-risk ratio**	**EMA evaluation**	**Number of clinical studies 04**	**Participants over 2000**	**Placebo controlled clinical studies**		**Comparison with approved drugs**	**No special drug approval procedure**	**No consequences for patients in ongoing clinical trials or compassionate use programmes**	Ongoing clinical trials (at the time the application was withdrawn)	Zenhale Withdrawal Q&A (europa.eu)	https://www.ema.europa.eu/en/documents/withdrawal-letter/withdrawal-letter-zenhale_en.pdf	**No orphan drugs**	1/20/11
Zometa,	zoledronic acid	Zoledronic acid	Phoenix Labs Unlimited Company (Erweiterung der Zulassung durch Novartis Europharm Ltd. beantragt)	Phoenix Labs Unlimited Company (Erweiterung der Zulassung durch Novartis Europharm Ltd. beantragt)	**Bisphosphonates**	**M05BA08**	**02 Neoplasms**	ICD-11 Chapte	use as adjuvant therapy in hormone receptor-positive early-stage breast cancer, only in combination with hormone therapy<br><br>already approved indications:<br>prevention of fractures in cancer patients, treatment of tumor-induced hypercalcemia	**Enzyme (Antagonism)(N1.A)**	**/N1.A<br>//**	Enzyme (Transferases, Antagonism)(N1.2.A)	**/N1.2.A<br>//**	**Post-authorisation**	3/20/01	12/14/10	• concerns regarding the manufacturer's responses/information (a1)<br>• doubts about efficacy<br>reason: lack of data to adequately demonstrate efficacy (a27.3)<br>• doubts about efficacy<br>reason: poor study quality (conduct) (a27.1.6)<br>• doubts about efficacy<br>reason: other reasons (treatments in the study were partly not according to eu standard of care, concerns about reliability of study data) (a27)	**/A1<br>/A27.3<br>/A27.1.6<br>/A27.<br>//**	25/01/2011	**No extension of authorisation, because of no positive benefit-risk ratio**	**EMA evaluation**	**Number of clinical studies 01**	**Participants 1000-1999**	**No placebo control**		**Comparison with approved drugs**	**No special drug approval procedure**	**No consequences for patients in ongoing clinical trials or compassionate use programmes**	Ongoing clinical trials (at the time the application was withdrawn)	https://www.ema.europa.eu/en/documents/medicine-qa/questions-and-answers-withdrawal-application-change-marketing-authorisation-zometa-zoledronic-acid_en.pdf	https://www.ema.europa.eu/en/documents/other/zometa-withdrawal-letter_en.pdf	**No orphan drugs**	1/25/11
Rasival,	unknown	Aliskiren/Valsartan	Novartis Europharm Limited	Novartis	**RAS inhibitors**	**C09DX02**	**11 Diseases of the circulatory system**	ICD-11 Chapter 11	essential hypertension (use in adults when blood pressure is already appropriately controlled by combination treatment with separately used aliskiren and valsartan)	**Enzyme (Antagonism)(N1.A)**<br><br>**Receptors (Antagonism)(N4.A)**	**/N1.A<br>/N4.A<br>//**	Enzyme (Hydrolases, Antagonism)(N1.3.A)<br><br>Receptors (GPCR, Antagonism) (N4.1.A)	**/N1.3.A<br>/N4.1.A<br>//**	**Initial authorisation**		9/15/10	• open questions from the EMA (a17)<br>• concerns regarding the manufacturer's responses/information (a1)<br>• open questions on bioavailability, bioequivalence, distribution and metabolism (on one or more topics) (particularly in relation to food intake) (a29)<br>• doubts about efficacy<br>reason: other reasons (lack of evidence for frequent use of the combination of active substances in clinical practice) (a27)	**/A17<br>/A1<br>/A29<br>/A27.<br>//**	17/09/2010	**No authorisation, because of no positive benefit-risk ratio**	**EMA evaluation**	**Number of clinical studies 01**	**Participants 1000-1999**	**No placebo control**	**SD**	**Comparison with approved drugs**	**No special drug approval procedure**	**No consequences for patients in ongoing clinical trials or compassionate use programmes**	No ongoing clinical trials (at the time the application was withdrawn)	https://www.ema.europa.eu/en/documents/medicine-qa/questions-and-answers-withdrawal-marketing-authorisation-application-rasival-aliskiren-valsartan_en.pdf	https://www.ema.europa.eu/en/documents/withdrawal-letter/withdrawal-letter-rasival_en.pdf	**No orphan drugs**	11/22/10
Brilence,	bazedoxifene	Bazedoxifene	Wyeth Europa Ltd	Wyeth	**Selective estrogen receptor modulators (SERM)**	**G03XC02**	**15 Diseases of the musculoskeletal system or connective tissue**	ICD-11 Chapter 15	postmenopausal osteoporosis	**Receptors (Antagonism) (N4.A)**	**/N4.A<br>//**	Receptors (nuclear receptors, Antagonism) (N4.4.A)	**/N4.4.A<br>//**	**Initial authorisation**		5/21/10	• not applicable (marketing authorisation was recommended) (a14.2)	**/A14.2<br>//**	28/05/2010	**Authorisation recommended**	**Marketing strategy**	**Number of clinical studies unknown**	**Participants unknown**	**placebo control unknown**	**SD**	**Comparison with approved drugs unknown**	**No special drug approval procedure**	**No consequences for patients in ongoing clinical trials or compassionate use programmes**	No ongoing clinical trials (at the time the application was withdrawn)	https://www.ema.europa.eu/en/documents/medicine-qa/questions-and-answers-withdrawal-marketing-authorisation-application-brilence_en.pdf	https://www.ema.europa.eu/en/documents/withdrawal-letter/withdrawal-letter-brilence_en.pdf	**No orphan drugs**	6/29/10
Intrinsa,	testosterone	Testosterone	Warner Chilcott UK Ltd.	Warner Chilcott UK Ltd.	**Androgens**	**G03BA03**	**17 Diseases related to sexual health**	ICD-11 Chapter 17	hypoactive sexual desire disorder (HSDD) in postmenopausal women with or without hormone therapy (regardless of surgical removal of uterus and ovaries)<br><br>already approved indications:<br>women with sexual appetite disorder (hypoactive sexual desire disorder, HSDD, use when uterus and ovaries were surgically removed and in combination with estrogens)	**other (N6)**<br><br>**Receptors (Agonism) (N4.B)**	**/N4.B<br>/N6.U<br>//**	other (N6)<br><br>Receptors (nuclear receptors, Agonism) (N4.4.B)	**/N6.U<br>/N4.4.B<br>//**	**Post-authorisation**	7/28/06	9/29/10	• open questions from the EMA (a17)<br>• concerns regarding the manufacturer's responses/information (a1)<br>• doubts about ensuring patient safety<br>reason: lack of data and/or inadequate long-term follow-up (a21.3)	**/A17<br>/A1<br>/A21.3<br>//**	29/09/2010	**No extension of authorisation, because of no positive benefit-risk ratio**	**Marketing strategy**	**Number of clinical studies 04**	**Participants over 2000**	**Placebo controlled clinical studies**		**No comparison with approved drugs**	**No special drug approval procedure**	**No consequences for patients in ongoing clinical trials or compassionate use programmes**	Ongoing clinical trials (at the time the application was withdrawn)	https://www.ema.europa.eu/en/documents/medicine-qa/questions-and-answers-withdrawal-application-change-marketing-authorisation-intrinsa-testosterone_en.pdf	https://www.ema.europa.eu/en/documents/other/withdrawal-letter-intrinsa_en.pdf	**No orphan drugs**	9/29/10
Joulferon,	unknown	Albinterferon alfa-2b	Novartis Europharm Limited	Novartis	**Interferons**	**L03AB12**	**01 Certain infectious or parasitic diseases**	ICD-11 Chapter 01	chronic hepatitis C	**Receptors (Agonism) (N4.B)**	**/N4.B<br>//**	Receptors (catalytic receptors, Agonism) (N4.3.B)	**/N4.3.B<br>//**	**Initial authorisation**		4/16/10	• not applicable (documents have not yet been assessed or recommendations not yet published) (a14.1)<br><br>note:<br>documents had not been fully assessed at the time of withdrawal of the application.	**/A14.1<br>//**	29/04/2010	**None (documents have not yet been evaluated)**	**Suspected EMA evaluation**	**Number of clinical studies 02**	**Participants over 2000**	**No placebo control**		**Comparison with approved drugs**	**No special drug approval procedure**	**No consequences for patients in ongoing clinical trials or compassionate use programmes**	Ongoing clinical trials (at the time the application was withdrawn)	https://www.ema.europa.eu/en/documents/medicine-qa/questions-and-answers-withdrawal-marketing-authorisation-application-joulferon_en.pdf	https://www.ema.europa.eu/en/documents/withdrawal-letter/withdrawal-letter-joulferon_en.pdf	**No orphan drugs**	4/29/10
Repaglinide Sun,	repaglinide	Repaglinide	Sun Pharmaceutical Industries B.V.	Sun Pharmaceutical Industries	**Other antidiabetics**	**A10BX02**	**05 Endocrine diseases, nutritional or metabolic diseases**	ICD-11 Chapter 05	diabetes mellitus, type 2<br><br>Note:<br>Use under certain conditions	**Transporter (Antagonism)(N3.A)**	**/N3.A<br>//**	Transporter (Antagonism) (N3.A)	**/N3.A<br>//**	**Initial authorisation**		3/25/10	• open questions from the EMA (a17)<br>• concerns regarding the manufacturer's responses/information (a1)<br>• concerns regarding medicinal product quality (contaminants and/or concerns regarding sterilisation) (a3.3)<br>• doubts about efficacy<br>reason: poor study quality (data analysis) (a27.1.3)	**/A17<br>/A1<br>/A3.3<br>/A27.1.3<br>//**	25/03/2010	**No authorisation, because of no positive benefit-risk ratio**	**Marketing strategy**	**Number of clinical studies 0**	**Participants 0**	**No placebo control**	**SDG**	**Comparison with approved drugs**	**Drug application for generic medicines**	**No consequences for patients in ongoing clinical trials or compassionate use programmes**	No ongoing clinical trials (at the time the application was withdrawn)	https://www.ema.europa.eu/en/documents/medicine-qa/questions-and-answers-withdrawal-marketing-authorisation-application-repaglinide-sun-repaglinide_en.pdf	https://www.ema.europa.eu/en/documents/withdrawal-letter/withdrawal-letter-repaglinide-sun_en.pdf	**No orphan drugs**	5/15/10
Cerepro,	sitimagene ceradenovec	Sitimagen ceradenovec	Ark Therapeutics	Ark Therapeutics	**Other medicines**	**L01XL01**	**02 Neoplasms**	ICD-11 Chapte	high-grade gliomas (use when surgery is possible and in combination with ganciclovir sodium)	**other (N6)**	**/N6.U<br>//**	other (N6)	**/N6.U<br>//**	**Initial authorisation**		3/8/10	• concerns regarding the manufacturer's responses/information (a1)<br>• doubts about assurance of patient safety<br>reason: side effects (a21.1)<br>• doubts about efficacy<br>reason: lack of data to sufficiently demonstrate efficacy (a27.3)	**/A1<br>/A21.1<br>/A27.3<br>//**	23/03/2010	**No authorisation, because of no positive benefit-risk ratio**	**EMA evaluation**	**Number of clinical studies 01**	**Participants 0200-0299**	**No placebo control**		**Comparison with approved drugs**	**No special drug approval procedure**	**No consequences for patients in ongoing clinical trials or compassionate use programmes**	No ongoing clinical trials (at the time the application was withdrawn)	https://www.ema.europa.eu/en/documents/medicine-qa/questions-and-answers-withdrawal-marketing-authorisation-application-cerepro_en.pdf	https://www.ema.europa.eu/en/documents/withdrawal-letter/withdrawal-letter-cerepro_en.pdf-0	**Orphan drugs**	2/1/11
Docetaxel Mylan,	docetaxel	Docetaxel	Mylan S.A.S	Mylan	**Cytostatics**	**L01CD02**	**02 Neoplasms**	ICD-11 Chapte	breast cancer, non-small cell lung carcinoma (NSCLC),<br>prostate carcinoma, adenocarcinoma of the stomach, head and neck carcinoma	**other proteins (Antagonism/Binding) (N5.A)**	**/N5.A<br>//**	other proteins (Microtubules, Antagonism) (N5.8.A)	**/N5.8.A<br>//**	**Initial authorisation**		3/8/10	• open questions from the EMA (a17)<br>• concerns regarding the manufacturer's responses/information (a1)<br>• open questions on bioavailability, bioequivalence, distribution and metabolism (for one or more topics) (a29)<br>• doubts about assurance of patient safety<br>reason: lack of data and/or inadequate long-term follow-up (a21.3)	**/A17<br>/A1<br>/A29<br>/A21.3<br>//**	26/03/2010	**No authorisation, because of no positive benefit-risk ratio**	**EMA evaluation**	**Number of clinical studies 0**	**Participants 0**	**No placebo control**	**SDG**	**Comparison with approved drugs**	**Drug application for generic medicines**	**No consequences for patients in ongoing clinical trials or compassionate use programmes**	No ongoing clinical trials (at the time the application was withdrawn)	https://www.ema.europa.eu/en/documents/medicine-qa/questions-and-answers-withdrawal-marketing-authorisation-application-docetaxel-mylan-docetaxel_en.pdf	https://www.ema.europa.eu/en/documents/withdrawal-letter/withdrawal-letter-docetaxel-mylan_en.pdf	**No orphan drugs**	3/26/10
Ratioepo,	epoetin theta	Epoetin Theta	Ratiopharm GmbH	Ratiopharm GmbH	**Other medicines**	**B03XA01**	**03 Diseases of the blood or blood-forming organs**<br><br>**23 External causes of morbidity or mortality**	ICD-11 Chapter 03<br><br>ICD-11 Chapter 23	anemia in<br>chronic kidney failure (adults) or<br>non-myeloid tumor in adults receiving chemotherapy	**Receptors (Agonism) (N4.B)**	**/N4.B<br>//**	Receptors (catalytic receptors, Agonism) (N4.3.B)	**/N4.3.B<br>//**	**Initial authorisation**		2/25/10	• not applicable (marketing authorisation was recommended) (a14.2)	**/A14.2<br>//**	22/03/2010	**Authorisation recommended**	**Marketing strategy**	**Number of clinical studies 07**	**Participants 1000-1999**	**Placebo controlled clinical studies**		**Comparison with approved drugs**	**No special drug approval procedure**	**No consequences for patients in ongoing clinical trials or compassionate use programmes**	No ongoing clinical trials (at the time the application was withdrawn)	https://www.ema.europa.eu/en/documents/medicine-qa/questions-and-answers-withdrawal-marketing-authorisation-application-ratioepo-epoetin-theta_en.pdf	https://www.ema.europa.eu/en/documents/withdrawal-letter/withdrawal-letter-ratioepo_en.pdf	**No orphan drugs**	3/22/10

Tyvaso,	unknown	Treprostinil sodium	United Therapeutics Europe Ltd	United Therapeutics Europe Ltd	**Prostacyclin analogs**	**B01AC21**	**11 Diseases of the circulatory system**	**ICD-11 Chapter 11**	pulmonary arterial hypertension (PAH)<br><br>Note:<br>Use under certain conditions (severity etc.)	**Receptors (Agonism) (N4.B)**	**/N4.B<br>//**	Receptors<br>(GPCR, Agonism) (N4.1.B)	**/N4.1.B<br>//**	**Initial authorisation**		2/17/10	• open questions from the EMA (a17)<br>• concerns regarding the manufacturer's responses/information (a1)<br>• doubts about compliance with good clinical practice (gcp) (a30)	**/A17<br>/A1<br>/A30<br>//**	23/03/2010	**No authorisation, because of no positive benefit-risk ratio**	**EMA evaluation**	**Number of clinical studies 01**	**Participants 0200-0299**	**Placebo controlled clinical studies**	**SD**	**Comparison with approved drugs**	**No special drug approval procedure**	**No consequences for patients in ongoing clinical trials or compassionate use programmes**	Ongoing clinical trials (at the time the application was withdrawn)	https://www.ema.europa.eu/en/documents/medicine-qa/questions-and-answers-withdrawal-marketing-authorisation-application-tyvaso_en.pdf	https://www.ema.europa.eu/en/documents/withdrawal-letter/withdrawal-letter-tyvaso_en.pdf	**Orphan drugs**	5/17/10
Comfyde,	carisbamate	Carisbamate	Janssen-Cilag International NV	Janssen Pharmaceutical Companies of Johnson & Johnson	**Antiepileptics**	**N03AX19**	**08 Diseases of the nervous system**	ICD-11 Chapter 08	epilepsy (partial seizures with or without secondary generalization)	**Ion channels (Antagonism) (N2.A)**	**/N2.A<br>//**	Ion channels (Antagonism) (N2.A)	**/N2.A<br>//**	**Initial authorisation**		1/15/10	• not applicable (documents have not yet been assessed or recommendations not yet published) (a14.1)<br><br>comment:<br>documents were not fully assessed at the time the application was withdrawn.	**/A14.1<br>//**	20/01/2010	**None (documents have not yet been evaluated)**	**Suspected EMA evaluation**	**Number of clinical studies 03**	**Participants 1000-1999**	**Placebo controlled clinical studies**		**Comparison with approved drugs**	**No special drug approval procedure**	**Consequences for patients in ongoing clinical trials or compassionate use programmes**	Ongoing clinical trials (at the time the application was withdrawn)	https://www.ema.europa.eu/en/documents/medicine-qa/questions-and-answers-withdrawal-marketing-authorisation-application-comfyde_en.pdf	https://www.ema.europa.eu/en/documents/withdrawal-letter/withdrawal-letter-comfyde_en.pdf	**No orphan drugs**	1/20/10
Ibuprofen/Diphenhydramine Hydrochloride Wyeth,	unknown	Ibuprofen/ Diphenhydramine Hydrochloride	Wyeth Consumer Healthcare	Wyeth	**Non-steroidal anti-inflammatory drugs (NSAIDs)<br><br>Antihistamines**	**M01AE51**	**21 Symptoms, signs or clinical findings not elsewhere classified (pain, unspecified)**	ICD-11 Chapter 21	pain<br>(mild to moderate<br>pain in adults with pain-related sleep disturbances, short-term treatment)	**Enzyme (Antagonism)(N1.A)**	**/N1.A<br>//**	Enzyme<br>(Oxidoreductases, Antagonism)(N1.1.A)	**/N1.1.A<br>//**	**Initial authorisation**		1/7/10	• open questions from the EMA (a17)<br>• concerns regarding the manufacturer's responses/information (a1)<br>• doubts about efficacy<br>reason: poor study quality (selection of participants) (a27.1.7)<br>• doubts about benefit (doubt about advantage over treatment with ibuprofen alone) (a18)<br>• doubts about assurance of patient safety (insufficient information regarding drug safety and interactions with other medicinal products) (a21)	**/A17<br>/A1<br>/A27.1.7<br>/A18<br>/A21.<br>//**	15/01/2010	**No authorisation, because of no positive benefit-risk ratio**	**EMA evaluation**	**Number of clinical studies 03**	**Participants 0500-0999**	**Placebo controlled clinical studies**		**Comparison with approved drugs**	**No special drug approval procedure**	**No consequences for patients in ongoing clinical trials or compassionate use programmes**	No ongoing clinical trials (at the time the application was withdrawn)	https://www.ema.europa.eu/en/documents/medicine-qa/questions-and-answers-withdrawal-marketing-authorisation-ibuprofendiphenhydramine-hydrochloride-wyeth_en.pdf	https://www.ema.europa.eu/en/documents/withdrawal-letter/withdrawal-letter-ibuprofen-diphenhydramine-hydrochloride-wyeth_en.pdf	**No orphan drugs**	4/20/10
Sliwens,	unknown	Eplivanserin	Sanofi Aventis	Sanofi	**Other medicines**	**Unknown**	**07 Sleep-wake disorders**	ICD-11 Chapter 07	long-term insomnia (sleep difficulties), problems with staying asleep	**Receptors (Antagonism) (N4.A)**	**/N4.A<br>//**	Receptors<br>(GPCR, Antagonism) (N4.1.A)	**/N4.1.A<br>//**	**Initial authorisation**		12/18/09	• open questions from the EMA (a17)<br>• concerns regarding the manufacturer's responses/information (a1)<br>• doubts about assurance of patient safety<br>reason: side effects (diverticulitis) (a21.1)<br>• doubts about assurance of patient safety<br>reason: lack of data and/or inadequate long-term follow-up (a21.3)<br>• doubts about efficacy<br>reason: other reasons (effect on sleep considered too small by chmp) (a27)	**/A17<br>/A1<br>/A21.1<br>/A21.3<br>/A27.<br>//**	21/12/2009	**No authorisation, because of no positive benefit-risk ratio**	**EMA evaluation**	**Number of clinical studies 05**	**Participants over 2000**	**Placebo controlled clinical studies**		**Comparison with approved drugs**	**No special drug approval procedure**	**No consequences for patients in ongoing clinical trials or compassionate use programmes**	No ongoing clinical trials (at the time the application was withdrawn)	https://www.ema.europa.eu/en/documents/medicine-qa/questions-and-answers-withdrawal-marketing-authorisation-application-sliwens-eplivanserin_en.pdf	https://www.ema.europa.eu/en/documents/withdrawal-letter/withdrawal-letter-sliwens_en.pdf	**No orphan drugs**	6/1/10
Recothrom,	thrombin alfa	Thrombin alfa	Bayer Schering Pharma AG	Bayer AG	**Other medicines**	**B02BD30**	**23 External causes of morbidity or mortality**	ICD-11 Chapter 23	hemostasis (in surgeries when surgical standard procedures are insufficient)	**other (N6)<br><br><br>Ligands (Agonism)(N9.B)**	**/N6.U<br>/N9.B<br>//**	other (N6)<br><br><br>Ligands (Agonism)(N9.B)	**/N6.U<br>/N9.B<br>//**	**Initial authorisation**		12/11/09	• open questions from the EMA (a17)<br>• concerns regarding the manufacturer's responses/information (a1)<br>• doubts about efficacy<br>reason: patients in the study not compatible with the target indication (a27.4)<br>• doubts about efficacy<br>reason: lack of data to sufficiently demonstrate efficacy (a27.3)<br>• doubts about efficacy<br>reason: flawed study design (sponge and spray were not investigated separately, concerns about the chosen comparator) (a27.2)	**/A17<br>/A1<br>/A27.4<br>/A27.3<br>/A27.2<br>//**	14/12/2009	**No authorisation, because of no positive benefit-risk ratio**	**EMA evaluation**	**Number of clinical studies 01**	**Participants 0300-0499**	**No placebo control**		**Comparison with approved drugs**	**No special drug approval procedure**	**No consequences for patients in ongoing clinical trials or compassionate use programmes**	No ongoing clinical trials (at the time the application was withdrawn)	https://www.ema.europa.eu/en/documents/medicine-qa/questions-and-answers-withdrawal-marketing-authorisation-application-recothrom-thrombin-alfa_en.pdf	https://www.ema.europa.eu/en/documents/withdrawal-letter/withdrawal-letter-recothrom_en.pdf	**No orphan drugs**	12/14/09
Ethyl Eicosapent soft gelatin capsules,	unknown	Ethyl Eicosapentaenoate	Amarin Neuroscience Ltd	Amarin Neuroscience Ltd	**Other medicines**	**Unknown**	**08 Diseases of the nervous system**	ICD-11 Chapter 08	Huntington's chorea	**other (N6)**	**/N6.U<br>//**	other (N6)	**/N6.U<br>//**	**Initial authorisation**		12/1/09	• open questions from the EMA (a17)<br>• concerns regarding the manufacturer's responses/information (a1)<br>• doubts about efficacy<br>reason: lack of data to sufficiently demonstrate efficacy (a27.3)<br>• open questions on bioavailability, bioequivalence, distribution and metabolism (for one or more topics) (a29)	**/A17<br>/A1<br>/A27.3<br>/A29<br>//**	8/12/2009	**No authorisation, because of no positive benefit-risk ratio**	**EMA evaluation**	**Number of clinical studies 03**	**Participants 0500-0999**	**Placebo controlled clinical studies**	**SD**	**No comparison with approved drugs**	**No special drug approval procedure**	**No consequences for patients in ongoing clinical trials or compassionate use programmes**	No ongoing clinical trials (at the time the application was withdrawn)	https://www.ema.europa.eu/en/documents/medicine-qa/questions-and-answers-withdrawal-marketing-authorisation-application-ethyl-eicosapent-soft-gelatin-capsules-ethyl-eicosapent_en.pdf	https://www.ema.europa.eu/en/documents/withdrawal-letter/withdrawal-letter-ethyl-eicosapent_en.pdf	**Orphan drugs**	12/8/09
Nenad,	Lisuride	Lisurid	Axxonis Pharma AG	Axxonis Pharma AG	**Other medicines**	**G02CB02**	**07 Sleep-wake disorders**	ICD-11 Chapter 07	restless legs syndrome (idiopathic, moderate to severe)	**Receptors (Agonism) (N4.B)**	**/N4.B<br>//**	Receptors<br>(GPCR, Agonism) (N4.1.B)	**/N4.1.B<br>//**	**Initial authorisation**		11/30/09	• concerns regarding the manufacturer's responses/information (a1)<br>• doubts about assurance of patient safety<br>reason: lack of data and/or inadequate long-term follow-up (a21.3)<br>• doubts about assurance of patient safety<br>reason: side effects (a21.1)<br>• concerns regarding the form of application (patch adheres poorly to the skin) (a2)	**/A1<br>/A21.3<br>/A21.1<br>/A2<br>//**	3/12/2009	**No authorisation, because of no positive benefit-risk ratio**	**EMA evaluation**	**Number of clinical studies 03**	**Participants 0500-0999**	**Placebo controlled clinical studies**	**SD**	**Comparison with approved drugs**	**No special drug approval procedure**	**No consequences for patients in ongoing clinical trials or compassionate use programmes**	Ongoing clinical trials (at the time the application was withdrawn)	https://www.ema.europa.eu/en/documents/medicine-qa/questions-and-answers-withdrawal-marketing-authorisation-application-nenad-lisuride_en.pdf	https://www.ema.europa.eu/en/documents/withdrawal-letter/withdrawal-letter-nenad_en.pdf	**No orphan drugs**	12/3/09
Oncophage,	unknown	Vitespen	Antigenics Therapeutics Limited	Antigenics Therapeutics Limited	**Other medicines**	**Unknown**	**02 Neoplasms**	ICD-11 Chapte	renal cell carcinoma (non-metastatic, with high risk of recurrence)	**other (N6)**	**/N6.U<br>//**	other (N6)	**/N6.U<br>//**	**Initial authorisation**		11/23/09	• concerns regarding the manufacturer's responses/information (a1)<br>• concerns regarding medicinal product quality (manufacturing process) (a3.1)<br>• concerns regarding medicinal product quality (insufficient information on ingredients) (a3)<br>• concerns regarding recommended dosage (a6)<br>• doubts about efficacy<br>reason: lack of data to sufficiently demonstrate efficacy (a27.3)	**/A1<br>/A3.1<br>/A3.<br>/A6<br>/A27.3<br>//**	21/12/2009	**No authorisation, because of no positive benefit-risk ratio**	**EMA evaluation**	**Number of clinical studies 01**	**Participants 0500-0999**	**No placebo control**		**No comparison with approved drugs**	**No special drug approval procedure**	**No consequences for patients in ongoing clinical trials or compassionate use programmes**	No ongoing clinical trials (at the time the application was withdrawn)	https://www.ema.europa.eu/en/documents/medicine-qa/questions-and-answers-withdrawal-marketing-authorisation-application-oncophage-vitespen_en.pdf	https://www.ema.europa.eu/en/documents/withdrawal-letter/withdrawal-letter-oncophage_en.pdf	**Orphan drugs**	12/21/09
Zactima,	vandetanib	Vandetanib	AstraZeneca	AstraZeneca	**Kinase inhibitors**	**L01EX04**	**02 Neoplasms**	ICD-11 Chapte	non-small cell lung carcinoma (NSCLC) (locally advanced or metastatic, use in combination with chemotherapy)	**Receptors (Antagonism) (N4.A)**	**/N4.A<br>//**	Receptors<br>(catalytic receptors, Antagonism) (N4.3.A)	**/N4.3.A<br>//**	**Initial authorisation**		10/27/09	• not applicable (documents have not yet been assessed or recommendations not yet published) (a14.1)<br><br>comment:<br>documents were not fully assessed at the time the application was withdrawn.	**/A14.1<br>//**	30/10/2009	**None (documents have not yet been evaluated)**	**Suspected EMA evaluation**	**Number of clinical studies 02**	**Participants 1000-1999**	**Placebo controlled clinical studies**		**Comparison with approved drugs**	**No special drug approval procedure**	**No consequences for patients in ongoing clinical trials or compassionate use programmes**	Ongoing clinical trials (at the time the application was withdrawn)	https://www.ema.europa.eu/en/documents/medicine-qa/questions-and-answers-withdrawal-marketing-authorisation-application-zactima-vandetanib_en.pdf	https://www.ema.europa.eu/en/documents/withdrawal-letter/withdrawal-letter-zactima_en.pdf	**No orphan drugs**	10/30/09
Abilify,	aripiprazole	Aripiprazole	Otsuka Pharmaceutical Netherlands B.V.	Otsuka Pharmaceutical Netherlands B.V.	**Antipsychotics**	**N05AX12**	**06 Mental, behavioral or neurological developmental disorders**	ICD-11 Chapter 06	depressive episodes (severe, treatment-resistant)<br><br>Note:<br>Use after previously insufficient antidepressant treatment<br><br>already approved indications:<br>schizophrenia, bipolar disorders	**Receptors (Agonism/Antagonism) (N4.C)**	**/N4.C<br>//**	Receptors<br>(GPCR, Agonism/Antagonism) (N4.1.C)	**/N4.1.C<br>//**	**Post-authorisation**	6/4/04	11/17/09	• open questions from the EMA (a17)<br>• concerns regarding the manufacturer's responses/information (a1)<br>• doubts about efficacy<br>reason: flawed study design (lack of double-blinding in the long-term study) (a27.2)<br>• doubts about efficacy<br>reason: poor study quality (selection of participants) (unclear whether all included patients had treatment-resistant depression) (a27.1.7)	**/A17<br>/A1<br>/A27.2<br>/A27.1.7<br>//**	19/11/2009	**No authorisation, because of no positive benefit-risk ratio**	**EMA evaluation**	**Number of clinical studies 04**	**Participants over 2000**	**Placebo controlled clinical studies**		**Comparison with approved drugs**	**No special drug approval procedure**	**No consequences for patients in ongoing clinical trials or compassionate use programmes**	Ongoing clinical trials (at the time the application was withdrawn)	https://www.ema.europa.eu/en/documents/medicine-qa/questions-and-answers-withdrawal-application-change-marketing-authorisation-abilify-aripiprazole_en.pdf	https://www.ema.europa.eu/en/documents/other/withdrawal-letter-abilify_en.pdf	**No orphan drugs**	11/19/09
Mersarex,	iclaprim	Iclaprim	Arpida A/S	Arpida A/S	**antibacterial drugs**	**J01EA03**	**01 Certain infectious or parasitic diseases**	ICD-11 Chapter 01	complicated infections of the skin and soft tissue (use in adults)	**other proteins (Antagonism/Binding) (N5.A)**	**/N5.A<br>//**	other proteins<br>(bacterial proteins / bacterial protein synthesis, Antagonism)(N5.9.A)	**/N5.9.A<br>//**	**Initial authorisation**		10/21/09	• open questions from the EMA (a17)<br>• concerns regarding the manufacturer's responses/information (a1)<br>• doubts about equivalent/improved efficacy compared with the eu reference medicinal product (a25)<br>• doubts about assurance of patient safety<br>reason: side effects (e.g., prolongation of the qtc interval) (a21.1)<br>• concerns regarding recommended dosage (a6)<br>• doubts about efficacy<br>reason: other reasons (existing antibiotic resistance) (a27)	**/A17<br>/A1<br>/A25<br>/21.1<br>/A6<br>/A27.<br>//**	23/10/2009	**No authorisation, because of no positive benefit-risk ratio**	**EMA evaluation**	**Number of clinical studies 02**	**Participants 0500-0999**	**No placebo control**		**Comparison with approved drugs**	**No special drug approval procedure**	**No consequences for patients in ongoing clinical trials or compassionate use programmes**	Ongoing clinical trials unknown (at the time the application was withdrawn)	https://www.ema.europa.eu/en/documents/medicine-qa/questions-and-answers-withdrawal-marketing-authorisation-application-mersarex-iclaprim_en.pdf	https://www.ema.europa.eu/en/documents/withdrawal-letter/withdrawal-letter-mersarex_en.pdf	**No orphan drugs**	10/23/09
Zunrisa,	casopitant	Casopitant	Glaxo Group Ltd	GlaxoSmithKline (GSK) Group	**Antiemetics**	**A04AD13**	**23 External causes of morbidity or mortality**	ICD-11 Chapter 23	nausea and<br>vomiting (in chemotherapy and surgical interventions)<br><br>Note:<br>Use in combination with other drugs	**Receptors (Antagonism) (N4.A)**	**/N4.A<br>//**	Receptors<br>(GPCR, Antagonism) (N4.1.A)	**/N4.1.A<br>//**	**Initial authorisation**		9/25/09	• open questions from the EMA (a17)<br>• concerns regarding the manufacturer's responses/information (a1)<br>• doubts about efficacy<br>reason: poor study quality (selection of participants) (with regard to studies on nausea/vomiting in surgical procedures) (a27.1.7)<br>• doubts about efficacy<br>reason: lack of data to sufficiently demonstrate efficacy (with regard to use in patients receiving chemotherapeutic agents that cause moderate nausea and vomiting) (a27.3)	**/A17<br>/A1<br>/A27.1.7<br>/A27.3<br>//**	13/10/2009	**No authorisation, because of no positive benefit-risk ratio**	**Development strategy**	**Number of clinical studies unknown**	**Participants unknown**	**No placebo control**	**SD**	**Comparison with approved drugs**	**No special drug approval procedure**	**No consequences for patients in ongoing clinical trials or compassionate use programmes**	Ongoing clinical trials (at the time the application was withdrawn)	https://www.ema.europa.eu/en/documents/medicine-qa/questions-and-answers-withdrawal-marketing-authorisation-application-zunrisa-casopitant_en.pdf	https://www.ema.europa.eu/en/documents/withdrawal-letter/withdrawal-letter-zunrisa_en.pdf	**No orphan drugs**	10/13/09

Opaxio,	unknown	Paclitaxel-Poliglumex	CTI Life Sciences Limited	CTI Life Sciences Limited	**Cytostatics**	**L01CD03**	**02 Neoplasms**	ICD-11 Chapte	advanced non-small cell lung carcinoma (NSCLC) (first-line treatment, ECOG status 2)	**other proteins (Antagonism/Binding) (N5.A)**	**/N5.A //**	other proteins (Microtubules, Antagonism) (N5.8.A)	**/N5.8.A //**	**Initial authorisation**	9/21/09	• open questions from the EMA (a17)<br>• concerns regarding the manufacturer's responses/information (a1)<br>• doubts about efficacy<br>reason: lack of data to sufficiently demonstrate efficacy (a27.3)<br>• doubts about assurance of patient safety<br>reason: side effects (neuropathies and unexplained deaths) (a21.1)<br>• concerns regarding medicinal product quality (contaminants and/or concerns regarding sterilisation) (a3.3)<br>• open questions on bioavailability, bioequivalence, distribution and metabolism (for one or more topics) (a29)	**/A17<br>/A1<br>/A27.3<br>/A21.1<br>/A3.3<br>/A29<br>//**	23/09/2009	**No authorisation, because of no positive benefit-risk ratio**	**EMA evaluation**	**Number of clinical studies 01**	**Participants 0300-0499**	**No placebo control**		**Comparison with approved drugs**	**No special drug approval procedure**	**No consequences for patients in ongoing clinical trials or compassionate use programmes**	No ongoing clinical trials (at the time the application was withdrawn)	https://www.ema.europa.eu/en/documents/medicine-qa/questions-and-answers-withdrawal-marketing-authorisation-application-opaxio-paclitaxel-poliglumex_en.pdf	https://www.ema.europa.eu/en/documents/withdrawal-letter/withdrawal-letter-opaxio_en.pdf	**No orphan drugs**	9/23/09
Ramvocid,	oritavancin	Oritavancin Diphosphate	Targanta Netherlands B.V.	Targanta Netherlands B.V.	**antibacterial drugs**	**J01XA05**	**01 Certain infectious or parasitic diseases**	ICD-11 Chapter 01	complicated infections of the skin and soft tissues<br><br>Note:<br>Use for infection caused by Gram-positive bacteria	**other proteins (Antagonism/Binding) (N5.A)**	**/N5.A //**	other proteins (bacterial proteins / bacterial protein synthesis, Antagonism)(N5.9.A)	**/N5.9.A //**	**Initial authorisation**	8/20/09	• open questions from the EMA (a17)<br>• concerns regarding the manufacturer's responses/information (a1)<br>• concerns regarding medicinal product quality (contaminants and/or concerns regarding sterilisation) (a3.3)<br>• concerns regarding recommended dosage (a6)	**/A17<br>/A1<br>/A3.3<br>/A6<br>//**	25/08/2009	**No authorisation, because of no positive benefit-risk ratio**	**EMA evaluation**	**Number of clinical studies 01**	**Participants 1000-1999**	**No placebo control**		**Comparison with approved drugs**	**No special drug approval procedure**	**No consequences for patients in ongoing clinical trials or compassionate use programmes**	No ongoing clinical trials (at the time the application was withdrawn)	https://www.ema.europa.eu/en/documents/medicine-qa/questions-and-answers-withdrawal-marketing-authorisation-application-ramvocid-oritavancin_en.pdf	https://www.ema.europa.eu/en/documents/withdrawal-letter/withdrawal-letter-ramvocid_en.pdf	**No orphan drugs**	8/25/09
Bosatria,	mepolizumab	Mepolizumab	Glaxo Group Ltd	GlaxoSmithKline (GSK) Group	**Monoclonal antibodies**	**R03DX09**	**04 Diseases of the immune system**	ICD-11 Chapter 04	hypereosinophilic syndrome<br><br>Note:<br>the need for corticosteroids should also be reduced by treatment with bosatria	**Ligands (antagonism)(N9.A)**	**/N9.A //**	Ligands (Cytokines, Antagonism)(N9.2.A)	**/N9.2.A //**	**Initial authorisation**	7/28/09	• open questions from the EMA (a17)<br>• concerns regarding the manufacturer's responses/information (a1)<br>• doubts about efficacy<br>reason: lack of data to sufficiently demonstrate efficacy (a27.3)<br>• concerns regarding medicinal product quality (questionable method for quantifying the different forms of the active substance in the medicinal product) (a3)	**/A17<br>/A1<br>/A27.3<br>/A3.<br>//**	29/07/2009	**No authorisation, because of no positive benefit-risk ratio**	**EMA evaluation**	**Number of clinical studies 01**	**Participants 0075-0099**	**Placebo controlled clinical studies**		**Comparison with approved drugs**	**No special drug approval procedure**	**No consequences for patients in ongoing clinical trials or compassionate use programmes**	Ongoing clinical trials (at the time the application was withdrawn)	https://www.ema.europa.eu/en/documents/medicine-qa/questions-and-answers-withdrawal-marketing-authorisation-application-bosatria-mepolizumab_en.pdf	https://www.ema.europa.eu/en/documents/withdrawal-letter/withdrawal-letter-bosatria_en.pdf	**Orphan drugs**	7/29/09
Factive,	unknown	Gemifloxacin	Menarini International Operations Luxembourg S.A.	Menarini International Operations Luxembourg S.A.	**Antibacterial drugs**	**J01MA15**	**12 Respiratory diseases**	ICD-11 Chapter 12	pneumonia (community-acquired, mild to moderate), acute exacerbation of chronic bronchitis	**other proteins (Antagonism/Binding) (N5.A)**	**/N5.A //**	other proteins (bacterial proteins / bacterial protein synthesis, Antagonism)(N5.9.A)	**/N5.9.A //**	**Initial authorisation**	6/17/09	• open questions from the EMA (a17)<br>• concerns regarding the manufacturer's responses/information (a1)<br>• doubts about assurance of patient safety<br>reason: side effects (e.g., increased gonadotoxicity compared with other fluoroquinolones) (a21.1)<br>• doubts about efficacy<br>reason: lack of data to sufficiently demonstrate efficacy (for five-day treatment of patients with moderate community-acquired pneumonia) (a27.3)<br>• doubts about efficacy<br>reason: poor study quality (regarding the indication for treatment of chronic bronchitis) (a27.1)<br>• doubts about efficacy<br>reason: poor study quality or flawed study design (lack of comparison with placebo and/or comparator/reference medicinal products) (regarding the indication for treatment of chronic bronchitis) (a27.1.4)	**/A17<br>/A1<br>/A21.1<br>/A27.3<br>/A27.1<br>/A27.1.4<br>//**	23/06/2009	**No authorisation, because of no positive benefit-risk ratio**	**EMA evaluation**	**Number of clinical studies 07**	**Participants over 2000**	**No placebo control**	**SD**	**Comparison with approved drugs**	**No special drug approval procedure**	**No consequences for patients in ongoing clinical trials or compassionate use programmes**	No ongoing clinical trials (at the time the application was withdrawn)	https://www.ema.europa.eu/en/documents/medicine-qa/questions-and-answers-withdrawal-marketing-authorisation-application-factive-gemifloxacin_en.pdf	https://www.ema.europa.eu/en/documents/withdrawal-letter/withdrawal-letter-factive_en.pdf	**No orphan drugs**	6/23/09
Contusugene Ladenovec Gendux,	contusugene ladenovec	Contusugene ladenovec	Gendux Molecular Limited	Gendux Molecular Limited	**Other medicines**	**Unknown**	**02 Neoplasms**	ICD-11 Chapte	squamous cell carcinomas of the head and neck (recurrent or refractory)	**other (N6)**	**/N6.U //**	other (N6)	**/N6.U //**	**Initial authorisation**	6/12/09	• open questions from the EMA (a17)<br>• concerns regarding the manufacturer's responses/information (a1)<br>• doubts about benefit (a18)<br>• concerns regarding medicinal product quality (manufacturing process) (a3.1)<br>• concerns regarding medicinal product quality (contaminants and/or concerns regarding sterilisation) (a3.3)<br>• doubts about assurance of patient safety<br>reason: side effects (insufficient data regarding toxicity) (a21.1)<br>• open questions on bioavailability, bioequivalence, distribution and metabolism (for one or more topics) (a29)	**/A17<br>/A1<br>/A18<br>/A3.1<br>/A3.3<br>/A21.1<br>/A29<br>//**	23/07/2009	**No authorisation, because of no positive benefit-risk ratio**	**Marketing strategy**	**Number of clinical studies 01**	**Participants 0100-0149**	**No placebo control**		**Comparison with approved drugs**	**No special drug approval procedure**	**No consequences for patients in ongoing clinical trials or compassionate use programmes**	No ongoing clinical trials (at the time the application was withdrawn)	https://www.ema.europa.eu/en/documents/medicine-qa/questions-and-answers-withdrawal-marketing-authorisation-application-contusugene-ladenovec-gendux-contusugene-ladenovec_en.pdf	https://www.ema.europa.eu/en/documents/withdrawal-letter/withdrawal-letter-contusugene-ladenovec-gendux_en.pdf	**No orphan drugs**	7/23/09
Biferonex,	interferon beta-1a	Interferon beta-1a	BioPartners GmbH	BioPartners GmbH	**Interferons**	**L03AB07**	**08 Diseases of the nervous system**	ICD-11 Chapter 08	multiple sclerosis (relapsing-remitting)	**Receptors (Agonism) (N4.B)**	**/N4.B //**	Receptors (catalytic receptors, Agonism) (N4.3.B)	**/N4.3.B //**	**Initial authorisation**	5/28/09	• concerns regarding the manufacturer's responses/information (a1)<br>• doubts about efficacy<br>reason: lack of data to sufficiently demonstrate efficacy (literature data insufficient due to differences in active substance composition observed) (a27.3)<br>• doubts about efficacy<br>reason: poor study quality (results not clearly interpretable) (a27.1)	**/A1<br>/A27.3<br>/A27.1<br>//**	29/05/2009	**No authorisation, because of no positive benefit-risk ratio**	**EMA evaluation**	**Number of clinical studies 01**	**Participants 0300-0499**	**Placebo controlled clinical studies**	**SD**	**No comparison with approved drugs**	**No special drug approval procedure**	**No consequences for patients in ongoing clinical trials or compassionate use programmes**	No ongoing clinical trials (at the time the application was withdrawn)	https://www.ema.europa.eu/en/documents/medicine-qa/questions-and-answers-withdrawal-marketing-authorisation-application-biferonex-interferon-beta-1a_en.pdf	https://www.ema.europa.eu/en/documents/withdrawal-letter/withdrawal-letter-biferonex_en.pdf	**No orphan drugs**	5/29/09
Lunivia,	eszopiclone	Eszopiclone	Sepracor Ltd.	Sepracor Ltd.	**Z-drugs (non-benzodiazepine hypnotics)**	**N05CF04**	**07 Sleep-wake disorders**	ICD-11 Chapter 07	sleep disorders (difficulty falling asleep, nocturnal<br>or early awakening)	**Receptors (Agonism) (N4.B)**	**/N4.B //**	Receptors (Ligand-gated ion channels, Agonism) (N4.2.B)	**/N4.2.B //**	**Initial authorisation**	5/13/09	• not applicable (marketing authorisation was recommended) (a14.2)<br><br>comment:<br>eszopiclone was not considered a new active substance by chmp, therefore lunivia could not have benefited from ten years of market exclusivity	**/A14.2<br>//**	15/05/2009	**Authorisation recommended**	**Marketing strategy**	**Number of clinical studies 08**	**Participants over 2000**	**Placebo controlled clinical studies**		**No comparison with approved drugs**	**No special drug approval procedure**	**No consequences for patients in ongoing clinical trials or compassionate use programmes**	Ongoing clinical trials (at the time the application was withdrawn)	https://www.ema.europa.eu/en/documents/medicine-qa/questions-and-answers-withdrawal-marketing-authorisation-application-lunivia-eszopiclone_en.pdf	https://www.ema.europa.eu/en/documents/withdrawal-letter/withdrawal-letter-lunivia_en.pdf	**No orphan drugs**	5/15/09
Clopidogrel Teva Pharma,	clopidogrel	Clopidogrel	Teva Pharma B.V	Teva Pharma B.V	**Platelet aggregation inhibitors**	**B01AC04**	**X Extension codes (Anticoagulation)**	ICD-11 Chapter X	prevention of atherothrombotic events (in patients with myocardial infarction, ischemic stroke or proven peripheral arterial occlusive disease)	**Receptors (Antagonism) (N4.A)**	**/N4.A //**	Receptors (GPCR, Antagonism) (N4.1.A)	**/N4.1.A //**	**Initial authorisation**	4/22/09	• open questions from the EMA (a17)<br>• concerns regarding the manufacturer's responses/information (a1)<br>• doubts about equivalent/improved efficacy compared with the eu reference medicinal product (with regard to the results of the bioequivalence study) (a25)	**/A17<br>/A1<br>/A25<br>//**	23/04/2009	**No authorisation, because of no positive benefit-risk ratio**	**Marketing strategy**	**Number of clinical studies 0**	**Participants 0**	**No placebo control**	**SDG**	**Comparison with approved drugs**	**Drug application for generic medicines**	**No consequences for patients in ongoing clinical trials or compassionate use programmes**	No ongoing clinical trials (at the time the application was withdrawn)	https://www.ema.europa.eu/en/documents/medicine-qa/questions-and-answers-withdrawal-marketing-authorisation-application-clopidogrel-teva-pharma-clopidogrel_en.pdf	https://www.ema.europa.eu/en/documents/withdrawal-letter/withdrawal-letter-clopidogrel-teva-pharma_en.pdf	**No orphan drugs**	4/23/09
Ixempra,	ixabepilone	Ixabepilone	Bristol-Myers Squibb Pharma EEIG	Bristol-Myers Squibb Pharma EEIG	**Cytostatics**	**L01DC04**	**02 Neoplasms**	ICD-11 Chapte	breast cancer (locally advanced or metastatic)<br><br>Note:<br>Use in combination with capecitabine if the preceding cytostatic therapy was unsuccessful	**other proteins (Antagonism/Binding) (N5.A)**	**/N5.A //**	other proteins (Microtubules, Antagonism) (N5.8.A)	**/N5.8.A //**	**Initial authorisation**	3/18/09	• concerns regarding the manufacturer's responses/information (a1)<br>• doubts about assurance of patient safety<br>reason: side effects (a21.1)	**/A1<br>/A21.1<br>//**	19/03/2009	**No authorisation, because of no positive benefit-risk ratio**	**EMA evaluation**	**Number of clinical studies 03**	**Participants over 2000**	**No placebo control**		**Comparison with approved drugs**	**No special drug approval procedure**	**No consequences for patients in ongoing clinical trials or compassionate use programmes**	Ongoing clinical trials (at the time the application was withdrawn)	https://www.ema.europa.eu/en/documents/medicine-qa/questions-and-answers-withdrawal-marketing-authorisation-ixempra_en.pdf	https://www.ema.europa.eu/en/documents/withdrawal-letter/withdrawal-letter-ixempra_en.pdf	**No orphan drugs**	3/19/09
Cylatron,	peginterferon alfa-2b	Peginterferon alfa-2b	SP Europe	SP Europe	**Interferons**	**L03AB10**	**02 Neoplasms**	ICD-11 Chapte	melanoma (stage III)	**Receptors (Agonism) (N4.B)**	**/N4.B //**	Receptors (catalytic receptors, Agonism) (N4.3.B)	**/N4.3.B //**	**Initial authorisation**	3/11/09	• open questions from the EMA (a17)<br>• concerns regarding the manufacturer's responses/information (a1)<br>• doubts about assurance of patient safety<br>reason: side effects (fatigue, depression) (a21.1)<br>• doubts about efficacy<br>reason: lack of data to sufficiently demonstrate efficacy (with regard to overall survival) (a27.3)	**/A17<br>/A1<br>/A21.1<br>/A27.3<br>//**	16/03/2009	**No authorisation, because of no positive benefit-risk ratio**	**EMA evaluation**	**Number of clinical studies 01**	**Participants 1000-1999**	**No placebo control**		**No comparison with approved drugs**	**No special drug approval procedure**	**No consequences for patients in ongoing clinical trials or compassionate use programmes**	Ongoing clinical trials (at the time the application was withdrawn)	https://www.ema.europa.eu/en/documents/medicine-qa/questions-and-answers-withdrawal-marketing-application-cylatron_en.pdf	https://www.ema.europa.eu/en/documents/withdrawal-letter/withdrawal-letter-cylatron_en.pdf	**No orphan drugs**	3/16/09
Vorinostat MSD,	vorinostat	Vorinostat	Merck Sharp and Dohme Ltd.	Merck Sharp & Dohme	**Cytostatics**	**L01XH01**	**02 Neoplasms**	ICD-11 Chapte	advanced cutaneous T-cell lymphoma (CTCL)<br><br>Note:<br>Use under certain conditions	**Enzyme (Antagonism)(N1.A)**	**/N1.A //**	Enzyme (Hydrolases, Antagonism)(N1.3.A)	**/N1.3.A //**	**Initial authorisation**	2/13/09	• open questions from the EMA (a17)<br>• concerns regarding the manufacturer's responses/information (a1)<br>• doubts about efficacy<br>reason: flawed study design (missing data on safety and overall survival) (a27.2)<br>• doubts about efficacy<br>reason: poor study quality or flawed study design (lack of comparison with placebo and/or comparator/reference medicinal products) (a27.1.4)<br>• doubts about assurance of patient safety<br>reason: side effects (e.g., risk of thromboembolic events) (a21.1)	**/A17<br>/A1<br>/A27.2<br>/A27.1.4<br>/A21.1<br>//**	17/02/2009	**No authorisation, because of no positive benefit-risk ratio**	**EMA evaluation**	**Number of clinical studies 01**	**Participants 0050-0074**	**No placebo control**		**No comparison with approved drugs**	**No special drug approval procedure**	**No consequences for patients in ongoing clinical trials or compassionate use programmes**	Ongoing clinical trials (at the time the application was withdrawn)	https://www.ema.europa.eu/en/documents/medicine-qa/questions-and-answers-withdrawal-marketing-application-vorinostat-msd_en.pdf	https://www.ema.europa.eu/en/documents/withdrawal-letter/withdrawal-letter-vorinostat-msd_en.pdf	**Orphan drugs**	2/17/09
Stalevo,	levodopa, carbidopa, entacapone	Levodopa, Carbidopa, Entacapone	Orion Corporation	Orion Corporation	**Parkinson's therapeutics**	**N04BA03**	**08 Diseases of the nervous system**	ICD-11 Chapter 08	Parkinson's disease (adults in early stage of Parkinson's disease starting with levodopa treatment)<br><br>already approved indications:<br>Parkinson's disease (use in patients who are already treated with a combination of levodopa and a DOPA-decarboxylase inhibitor)	**Enzyme (Antagonism)(N1.A)**<br><br>**other (N6)**	**/N1.A<br>/N6.U<br>//**	other (N6)<br><br>Enzyme (Lyases, Antagonism) (N1.4.A)<br><br>Enzyme (Transferases, Antagonism)(N1.2.A)	**/N6.U<br>/N1.4.A<br>/N1.2.A<br>//**	**Post-authorisation**	3/6/09	• open questions from the EMA (a17)<br>• concerns regarding the manufacturer's responses/information (a1)<br>• doubts about efficacy<br>reason: lack of data to sufficiently demonstrate efficacy (a27.3)<br>• doubts about assurance of patient safety<br>reason: side effects (e.g., diarrhoea and weight loss) (a21.1)<br>• doubts about efficacy<br>reason: flawed study design (urine discoloration as a known side effect reveals treatment to patients, making simple blinding questionable) (a27.2)	**/A17<br>/A1<br>/A27.3<br>/A21.1<br>/A27.2<br>//**	10/03/2009	**No authorisation, because of no positive benefit-risk ratio**	**EMA evaluation**	**Number of clinical studies 01**	**Participants 0300-0499**	**No placebo control**		**Comparison with approved drugs**	**No special drug approval procedure**	**No consequences for patients in ongoing clinical trials or compassionate use programmes**	Ongoing clinical trials (at the time the application was withdrawn)	https://www.ema.europa.eu/en/documents/medicine-qa/questions-and-answers-withdrawal-application-change-marketing-authorisation-stalevo_en.pdf	https://www.ema.europa.eu/en/documents/other/withdrawal-letter-stalevo_en.pdf	**No orphan drugs**	3/10/09

Advexin,	contusugene ladenovec	Contusugene ladenovec	Gendux Molecular Limited	Gendux Molecular Limited	**Other medicines**	**Unknown**	**02 Neoplasms**	ICD-11 Chapte	Li-Fraumeni tumor in Li-Fraumeni syndrome	**other (N6)**	**/N6.U //**	other (N6)	**/N6.U //**	**Initial authorisation**	12/17/08	• open questions from the EMA (a17)<br>• concerns regarding the manufacturer's responses/information (a1)<br>• doubts about efficacy<br>reason: lack of data to sufficiently demonstrate efficacy (a27.3)<br>• concerns regarding medicinal product quality (manufacturing process) (a3.1)<br>• open questions on bioavailability, bioequivalence, distribution and metabolism (for one or more topics) (a29)<br>• doubts about assurance of patient safety (a21)<br>• concerns regarding the form of application (a2)	**/A17 /A1 /A27.3 /A3.1 /A29 /A21. /A2 //**	19/12/2009	**No authorisation, because of no positive benefit-risk ratio**	**Marketing strategy**	**Number of clinical studies 01**	**Participants 0001-0024**	**No placebo control**	**SD**	**No comparison with approved drugs**	**No special drug approval procedure**	**Consequences for patients in ongoing clinical trials or compassionate use programmes unknown**	Ongoing clinical trials unknown (at the time the application was withdrawn)	https://www.ema.europa.eu/en/documents/medicine-qa/questions-and-answers-withdrawal-marketing-application-advexin_en.pdf	https://www.ema.europa.eu/en/documents/withdrawal-letter/withdrawal-letter-advexin_en.pdf	**Orphan drugs**	12/19/09
Invega,	paliperidone	Paliperidone	Janssen-Cilag International NV	Janssen Pharmaceutical Companies of Johnson & Johnson	**Antipsychotics**	**N05AX13**	**06 Mental, behavioral or neurological developmental disorders**	ICD-11 Chapter 06	acute manic episodes in connection with bipolar I disorder<br><br>already approved indications: schizophrenia	**Receptors (Antagonism) (N4.A)**	**/N4.A //**	Receptors (GPCR, Antagonism) (N4.1.A)	**/N4.1.A //**	**Post-authorisation**	12/15/08	• not applicable (documents have not yet been assessed or recommendations not yet published) (a14.1)<br><br>comment:<br>documents were not fully assessed at the time the application was withdrawn.	**/A14.1 //**	17/12/2008	**None (documents have not yet been evaluated)**	**Suspected EMA evaluation**	**Number of clinical studies 03**	**Participants 1000-1999**	**Placebo controlled clinical studies**		**Comparison with approved drugs**	**No special drug approval procedure**	**No consequences for patients in ongoing clinical trials or compassionate use programmes**	Ongoing clinical trials (at the time the application was withdrawn)	https://www.ema.europa.eu/en/documents/medicine-qa/questions-and-answers-withdrawal-application-change-marketing-authorisation-invega_en.pdf	https://www.ema.europa.eu/en/documents/other/withdrawal-letter-invega_en.pdf	**No orphan drugs**	12/17/08
Theraloc,	nimotuzumab	Nimotuzumab	Oncoscience AG	Oncoscience AG	**Monoclonal antibodies**	**Unknown**	**02 Neoplasms**	ICD-11 Chapte	glioma (high-grade, treatment for adolescents and children from three years)<br><br>Note:<br>Use in relapsed or treatment-resistant glioma	**Receptors (Antagonism) (N4.A)**	**/N4.A //**	Receptors (catalytic receptors, Antagonism) (N4.3.A)	**/N4.3.A //**	**Initial authorisation**	12/1/08	• open questions from the EMA (a17)<br>• concerns regarding the manufacturer's responses/information (a1)<br>• concerns regarding medicinal product quality (manufacturing process) (a3.1)<br>• open questions on bioavailability, bioequivalence, distribution and metabolism (for one or more topics) (a29)<br>• doubts about efficacy<br>reason: lack of data to sufficiently demonstrate efficacy (a27.3)<br>• doubts about assurance of patient safety<br>reason: side effects (a21.1)<br>• doubts about efficacy<br>reason: poor study quality (selection of participants) (a27.1.7)	**/A17 /A1 /A3.1 /A29 /A27.3 /A21.1 /A27.1.7 //**	4/12/2008	**No authorisation, because of no positive benefit-risk ratio**	**EMA evaluation**	**Number of clinical studies 01**	**Participants 0025-0049**	**No placebo control**		**No comparison with approved drugs**	**No special drug approval procedure**	**No consequences for patients in ongoing clinical trials or compassionate use programmes**	Ongoing clinical trials (at the time the application was withdrawn)	https://www.ema.europa.eu/en/documents/medicine-qa/questions-and-answers-withdrawal-marketing-application-theraloc_en.pdf	https://www.ema.europa.eu/en/documents/withdrawal-letter/withdrawal-letter-theraloc_en.pdf	**Orphan drugs**	12/4/08
Viagra,	sildenafil	Sildenafil	Upjohn EESV	Upjohn EESV	**PDE-5 inhibitors**	**G04BE03**	**17 Diseases related to sexual health**	ICD-11 Chapter 17	erectile dysfunction (application for exemption from prescription requirement)<br><br>already approved indications: erectile dysfunction (medication is prescription-only)	**Enzyme (Antagonism)(N1.A)**	**/N1.A //**	Enzyme (Hydrolases, Antagonism)(N1.3.A)	**/N1.3.A //**	**Post-authorisation**	11/19/08	• open questions from the EMA (a17)<br>• concerns regarding the manufacturer's responses/information (a1)<br>• doubts about assurance of patient safety (e.g., side effects, possible delay in diagnosis of underlying diseases, and potential misuse of the medicinal product) (a21)	**/A17 /A1 /A21. //**	20/11/2008	**No authorisation, because of no positive benefit-risk ratio**	**EMA evaluation**	**Number of clinical studies 0**	**Participants 0**	**No placebo control**	**SD**	**No comparison with approved drugs**	**No special drug approval procedure**	**No consequences for patients in ongoing clinical trials or compassionate use programmes**	No ongoing clinical trials (at the time the application was withdrawn)	https://www.ema.europa.eu/en/documents/medicine-qa/questions-and-answers-withdrawal-application-change-marketing-authorisation-viagra_en.pdf	https://www.ema.europa.eu/en/documents/other/withdrawal-letter-viagra_en.pdf	**No orphan drugs**	11/20/08
Vekacia,	ciclosporin	Cyclosporine	Novagali Pharma S.A.	Novagali Pharma S.A.	**Immunosuppressants (no monoclonal antibodies)**	**S01XA18**	**09 Diseases of the visual system**	ICD-11 Chapter 09	vernal conjunctivitis	**Enzyme (Antagonism)(N1.A)**	**/N1.A //**	Enzyme (Isomerases, Antagonism) (N1.5.A)	**/N1.5.A //**	**Initial authorisation**	11/14/08	• open questions from the EMA (a17)<br>• concerns regarding the manufacturer's responses/information (a1)<br>• doubts about assurance of patient safety<br>reason: lack of data and/or inadequate long-term follow-up (a21.3)<br>• doubts about efficacy<br>reason: lack of data to sufficiently demonstrate efficacy (a27.3)<br>• doubts about efficacy<br>reason: poor study quality (data analysis) (a27.1.3)<br>• doubts about efficacy<br>reason: poor study quality (selection of participants) (a27.1.7)<br>• doubts about efficacy<br>reason: poor study quality (conduct) (measurement of symptoms) (a27.1.6)	**/A17 /A1 /A21.3 /A27.3 /A27.1.3 /A27.1.7 /A27.1.6 //**	18/11/2008	**No authorisation, because of no positive benefit-risk ratio**	**EMA evaluation**	**Number of clinical studies 01**	**Participants 0100-0149**	**Placebo controlled clinical studies**	**SD**	**No comparison with approved drugs**	**No special drug approval procedure**	**No consequences for patients in ongoing clinical trials or compassionate use programmes**	No ongoing clinical trials (at the time the application was withdrawn)	https://www.ema.europa.eu/en/documents/medicine-qa/questions-and-answers-withdrawal-marketing-application-vekacia_en.pdf	https://www.ema.europa.eu/en/documents/withdrawal-letter/withdrawal-letter-vekacia_en.pdf	**Orphan drugs**	11/18/08
Taxotere,	docetaxel	Docetaxel	Sanofi Mature IP	Sanofi	**Cytostatics**	**L01CD02**	**02 Neoplasms**	ICD-11 Chapte	breast cancer (resectable, HER2 positive)<br><br>Note:<br>Use in combination with trastuzumab, after a<br>treatment with doxorubicin and cyclophosphamide or use in combination with trastuzumab and carboplatin<br><br>already approved indications: breast cancer (advanced, metastatic or resectable), non-small cell lung carcinoma (NSCLC) (advanced or metastatic), prostate carcinoma, adenocarcinoma of the stomach (metastatic), advanced head or neck cancer	**other proteins (Antagonism/Binding) (N5.A)**	**/N5.A //**	other proteins (Microtubules, Antagonism) (N5.8.A)	**/N5.8.A //**	**Post-authorisation**	11/14/08	• concerns regarding the manufacturer's responses/information (a1)<br>• doubts about efficacy<br>reason: lack of data to sufficiently demonstrate efficacy (a27.3)<br>• doubts about efficacy<br>reason: flawed study design (a27.2)	**/A1 /A27.3 /A27.2 //**	17/11/2008	**No authorisation, because of no positive benefit-risk ratio**	**EMA evaluation**	**Number of clinical studies 01**	**Participants over 2000**	**No placebo control**		**Comparison with approved drugs**	**No special drug approval procedure**	**No consequences for patients in ongoing clinical trials or compassionate use programmes**	Ongoing clinical trials (at the time the application was withdrawn)	https://www.ema.europa.eu/en/documents/medicine-qa/questions-and-answers-withdrawal-application-change-marketing-authorisation-taxoteredocetaxel-winthrop_en.pdf	https://www.ema.europa.eu/en/documents/other/withdrawal-letter-taxotere_en.pdf	**No orphan drugs**	11/17/08
Docetaxel Zentiva (previously Docetaxel Winthrop),	docetaxel	Docetaxel	Zentiva, k.s.	Zentiva, k.s.	**Cytostatics**	**L01CD02**	**02 Neoplasms**	ICD-11 Chapte	breast cancer (resectable, HER2 positive)<br><br>Note:<br>Use in combination with trastuzumab, after a<br>treatment with doxorubicin and cyclophosphamide or use in combination with trastuzumab and carboplatin<br><br>already approved indications: breast cancer (advanced, metastatic or resectable), non-small cell lung carcinoma (NSCLC) (advanced or metastatic), prostate carcinoma, adenocarcinoma of the stomach (metastatic), advanced head or neck cancer	**other proteins (Antagonism/Binding) (N5.A)**	**/N5.A //**	other proteins (Microtubules, Antagonism) (N5.8.A)	**/N5.8.A //**	**Post-authorisation**	11/14/08	• concerns regarding the manufacturer's responses/information (a1)<br>• doubts about efficacy<br>reason: lack of data to sufficiently demonstrate efficacy (a27.3)<br>• doubts about efficacy<br>reason: flawed study design (a27.2)	**/A1 /A27.3 /A27.2 //**	17/11/2008	**No authorisation, because of no positive benefit-risk ratio**	**EMA evaluation**	**Number of clinical studies 01**	**Participants over 2000**	**No placebo control**		**Comparison with approved drugs**	**No special drug approval procedure**	**No consequences for patients in ongoing clinical trials or compassionate use programmes**	Ongoing clinical trials (at the time the application was withdrawn)	https://www.ema.europa.eu/en/documents/medicine-qa/questions-and-answers-withdrawal-application-change-marketing-authorisation-taxoteredocetaxel-winthrop_en.pdf	https://www.ema.europa.eu/en/documents/other/withdrawal-letter-docetaxel-winthrop_en.pdf	**No orphan drugs**	11/17/08
Vibativ,	telavancin	Telavancin	Astellas Pharma Europe B.V.	Astellas Pharma Europe B. V.	**Antibacterial drugs**	**J01XA03**	**01 Certain infectious or parasitic diseases**	ICD-11 Chapter 01	complicated skin and soft tissue infections (in known or suspected infections by Gram-positive pathogens, including MRSA)	**other proteins (Antagonism/Binding) (N5.A)**	**/N5.A //**	other proteins (bacterial proteins / bacterial protein synthesis, Antagonism)(N5.9.A)	**/N5.9.A //**	**Initial authorisation**	10/20/08	• open questions from the EMA (a17)<br>• concerns regarding the manufacturer's responses/information (a1)<br>• doubts about equivalent/improved efficacy compared with the eu reference medicinal product (a25)<br>• doubts about assurance of patient safety<br>reason: side effects (possible prolongation of qt time) (a21.1)<br>• concerns regarding medicinal product quality (manufacturing process) (a3.1)<br>• concerns regarding medicinal product quality (contaminants and/or concerns regarding sterilisation) (a3.3)	**/A17 /A1 /A25 /A21.1 /A3.1 /A3.3 //**	24/10/2008	**No authorisation, because of no positive benefit-risk ratio**	**EMA evaluation**	**Number of clinical studies 02**	**Participants over 2000**	**No placebo control**		**Comparison with approved drugs**	**No special drug approval procedure**	**No consequences for patients in ongoing clinical trials or compassionate use programmes**	No ongoing clinical trials (at the time the application was withdrawn)	https://www.ema.europa.eu/en/documents/medicine-qa/questions-and-answers-withdrawal-marketing-application-vibativ_en.pdf	https://www.ema.europa.eu/en/documents/withdrawal-letter/withdrawal-letter-vibativ_en.pdf	**No orphan drugs**	10/24/08
Ellefore,	desvenlafaxine	Desvenlafaxine	Wyeth Europa Ltd	Wyeth	**Serotonin-norepinephrine reuptake inhibitors (SNRI)**	**N06AX23**	**06 Mental, behavioral or neurological developmental disorders**	ICD-11 Chapter 06	major depression	**Transporter (Antagonism)(N3.A)**	**/N3.A //**	Transporter (SLC-Transporters, Antagonism)(N3.1.A)	**/N3.1.A //**	**Initial authorisation**	10/13/08	• concerns regarding the manufacturer's responses/information (a1)<br>• doubts about assurance of patient safety<br>reason: lack of data and/or inadequate long-term follow-up (a21.3)<br>• doubts about efficacy<br>reason: lack of data to sufficiently demonstrate efficacy (a27.3)<br>• doubts about equivalent/improved efficacy compared with the eu reference medicinal product (venlafaxine) (a25)	**/A1 /A21.3 /A27.3 /A25 //**	15/10/2008	**No authorisation, because of no positive benefit-risk ratio**	**Marketing strategy**	**Number of clinical studies 09**	**Participants over 2000**	**Placebo controlled clinical studies**		**No comparison with approved drugs**	**No special drug approval procedure**	**No consequences for patients in ongoing clinical trials or compassionate use programmes**	Ongoing clinical trials (at the time the application was withdrawn)	https://www.ema.europa.eu/en/documents/medicine-qa/questions-and-answers-withdrawal-marketing-application-ellefore_en.pdf	https://www.ema.europa.eu/en/documents/withdrawal-letter/withdrawal-letter-ellefore_en.pdf	**No orphan drugs**	10/15/08

Lacosamide Pain UCB Pharma,	lacosamide	Lacosamide	UCB Pharma S.A.	UCB Pharma S.A.	**Other medicines**	**N03AX18**	**08 Diseases of the nervous system**	ICD-11 Chapter 08	diabetic neuropathic pain (diabetic peripheral neuropathy)	**Ion channels (Antagonism) (N2.A)**	**/N2.A<br>//**	Ion channels (Antagonism) (N2.A)	**/N2.A<br>//**	**Initial authorisation**	9/25/08	• open questions from the EMA (a17)<br>• concerns regarding the manufacturer's responses/information (a1)<br>• doubts about efficacy<br>reason: lack of data to sufficiently demonstrate efficacy (a27.3)<br>• doubts about assurance of patient safety<br>reason: side effects (possible effects on heart and cns) (a21.1)	**/A17<br>/A1<br>/A27.3<br>/A21.1<br>//**	26/09/2008	**No authorisation, because of no positive benefit-risk ratio**	**EMA evaluation**	**Number of clinical studies 04**	**Participants 1000-1999**	**Placebo controlled clinical studies**		**No comparison with approved drugs**	**No special drug approval procedure**	**No consequences for patients in ongoing clinical trials or compassionate use programmes**	Ongoing clinical trials (at the time the application was withdrawn)	https://www.ema.europa.eu/en/documents/medicine-qa/questions-and-answers-withdrawal-marketing-application-lacosamide-pain-ucb-pharma_en.pdf	https://www.ema.europa.eu/en/documents/withdrawal-letter/withdrawal-letter-lacosamide-pain-ucb-pharma_en.pdf	**No orphan drugs**	9/26/08
Ramelteon,	ramelteon	Ramelteon	Takeda Global Research and Development Centre (Europe)	Takeda	**Other medicines**	**N05CH02**	**07 Sleep-wake disorders**	ICD-11 Chapter 07	primary insomnia (difficulty falling asleep and staying asleep as well as poor sleep quality)	**Receptors (Agonism) (N4.B)**	**/N4.B<br>//**	Receptors<br>(GPCR, Agonism) (N4.1.B)	**/N4.1.B<br>//**	**Initial authorisation**	9/19/08	• concerns regarding the manufacturer's responses/information (a1)<br>• doubts about efficacy<br>reason: lack of data to sufficiently demonstrate efficacy (a27.3)<br>• doubts about efficacy<br>reason: flawed study design (efficacy assessed only by measuring time to fall asleep) (a27.2)<br>• doubts about assurance of patient safety<br>reason: lack of data and/or inadequate long-term follow-up (a21.3)	**/A1<br>/A27.3<br>/A27.2<br>/A21.3<br>//**	25/09/2008	**No authorisation, because of no positive benefit-risk ratio**	**Development strategy**	**Number of clinical studies 03**	**Participants 2000**	**Placebo controlled clinical studies**	**SD**	**No comparison with approved drugs**	**No special drug approval procedure**	**No consequences for patients in ongoing clinical trials or compassionate use programmes**	No ongoing clinical trials (at the time the application was withdrawn)	https://www.ema.europa.eu/en/documents/medicine-qa/questions-and-answers-withdrawal-marketing-application-ramelteon_en.pdf	https://www.ema.europa.eu/en/documents/withdrawal-letter/withdrawal-letter-ramelteon_en.pdf	**No orphan drugs**	9/25/08
Exulett,	dalbavancin	Dalbavancin	Pfizer Limited	Pfizer	**Antibacterial drugs**	**J01XA04**	**01 Certain infectious or parasitic diseases**	ICD-11 Chapter 01	complicated skin and soft tissue infections (in known or suspected infections by Gram-positive pathogens, including MRSA)	**other proteins (Antagonism/Binding) (N5.A)**	**/N5.A<br>//**	other proteins<br>(bacterial proteins / bacterial protein synthesis, Antagonism)(N5.9.A)	**/N5.9.A<br>//**	**Initial authorisation**	9/9/08	• open questions from the EMA (a17)<br>• concerns regarding the manufacturer's responses/information (a1)<br>• doubts about efficacy<br>reason: lack of data to sufficiently demonstrate efficacy (a27.3)<br>• concerns regarding medicinal product quality (manufacturing process) (a3.1)<br>• doubts about efficacy<br>reason: poor study conduct (a27.1.6)<br>• doubts about efficacy<br>reason: patients in the study not compatible with the target indication (partly not complicated infections) (a27.4)	**/A17<br>/A1<br>/A27.3<br>/A3.1<br>/A27.1.6<br>/A27.4<br>//**	10/09/2008	**No authorisation, because of no positive benefit-risk ratio**	**Development strategy**	**Number of clinical studies 01**	**Participants 0500-0999**	**No placebo control**		**Comparison with approved drugs**	**No special drug approval procedure**	**No consequences for patients in ongoing clinical trials or compassionate use programmes**	Ongoing clinical trials (at the time the application was withdrawn)	https://www.ema.europa.eu/en/documents/medicine-qa/questions-and-answers-withdrawal-marketing-application-exulett_en.pdf	https://www.ema.europa.eu/en/documents/withdrawal-letter/withdrawal-letter-exulett_en.pdf	**No orphan drugs**	9/10/08
Orplatna,	satraplatin	Satraplatin	Pharmion Ltd.	Pharmion Ltd.	**Cytostatics**	**L01XA04**	**02 Neoplasms**	ICD-11 Chapte	prostate carcinoma (metastatic, hormone-refractory)<br><br>Note:<br>Use under certain conditions (e.g., after unsuccessful first-line therapy)	**other (N6)**	**/N6.U<br>//**	other (N6)	**/N6.U<br>//**	**Initial authorisation**	8/1/08	• open questions from the EMA (a17)<br>• concerns regarding the manufacturer's responses/information (a1)<br>• doubts about efficacy<br>reason: lack of data to sufficiently demonstrate efficacy (a27.3)<br>• doubts about efficacy<br>reason: flawed study design ("combined method" for measuring efficacy regarding progression-free survival is questionable) (a27.2)<br>• doubts about assurance of patient safety<br>reason: side effects (especially in first-line treatment with docetaxel) (a21.1)	**/A17<br>/A1<br>/A27.3<br>/A27.2<br>/A21.1<br>//**	5/08/2008	**No authorisation, because of no positive benefit-risk ratio**	**EMA evaluation**	**Number of clinical studies 01**	**Participants 0500-0999**	**Placebo controlled clinical studies**		**Comparison with approved drugs**	**No special drug approval procedure**	**No consequences for patients in ongoing clinical trials or compassionate use programmes**	Ongoing clinical trials (at the time the application was withdrawn)	https://www.ema.europa.eu/en/documents/medicine-qa/questions-and-answers-withdrawal-marketing-application-orplatna_en.pdf	https://www.ema.europa.eu/en/documents/withdrawal-letter/withdrawal-letter-orplatna_en.pdf	**No orphan drugs**	8/5/08
Diractin,	ketoprofen	Ketoprofen	IDEA AG	IDEA AG	**Non-steroidal anti-inflammatory drugs (NSAIDs)**	**M02AA10**	**15 Diseases of the musculoskeletal system or connective tissue**	ICD-11 Chapter 15	osteoarthritis (osteoarthritis)	**Enzyme (Antagonism)(N1.A)**	**/N1.A<br>//**	Enzyme<br>(Oxidoreductases, Antagonism)(N1.1.A)	**/N1.1.A<br>//**	**Initial authorisation**	7/23/08	• open questions from the EMA (a17)<br>• concerns regarding the manufacturer's responses/information (a1)<br>• doubts about efficacy<br>reason: lack of data to sufficiently demonstrate efficacy (a27.3)	**/A17<br>/A1<br>/A27.3<br>//**	24/07/2008	**No authorisation, because of no positive benefit-risk ratio**	**EMA evaluation**	**Number of clinical studies 01**	**Participants 0500-0999**	**Placebo controlled clinical studies**		**No comparison with approved drugs**	**No special drug approval procedure**	**No consequences for patients in ongoing clinical trials or compassionate use programmes**	Ongoing clinical trials (at the time the application was withdrawn)	https://www.ema.europa.eu/en/documents/medicine-qa/questions-and-answers-withdrawal-marketing-application-diractin_en.pdf	https://www.ema.europa.eu/en/documents/withdrawal-letter/withdrawal-letter-diractin_en.pdf	**No orphan drugs**	7/24/08
Spanidin,	gusperimus	Gusperimus	Euro Nippon Kayaku GmbH	Euro Nippon Kayaku GmbH	**Immunosuppressants (no monoclonal antibodies)**	**L04AA19**	**04 Diseases of the immune system**	ICD-11 Chapter 04	Wegener's granulomatosis (clinically refractory)<br><br>Note:<br>Use under certain conditions (e.g., after unsuccessful first treatment)	**other (N6)**	**/N6.U<br>//**	other (N6)	**/N6.U<br>//**	**Initial authorisation**	6/17/08	• open questions from the EMA (a17)<br>• concerns regarding the manufacturer's responses/information (a1)<br>• doubts about efficacy<br>reason: flawed study design (a27.2)<br>• doubts about efficacy<br>reason: lack of data to sufficiently demonstrate efficacy (a27.3)<br>• doubts about efficacy<br>reason: patients in the study not compatible with the target indication (partly no refractory disease) (a27.4)<br>• doubts about efficacy<br>reason: poor study conduct (a27.1.6)<br>• doubts about efficacy<br>reason: poor study quality or flawed study design (lack of comparison with placebo and/or comparator/reference medicinal products) (a27.1.4)	**/A17<br>/A1<br>/A27.3<br>/A27.2<br>/A27.4<br>/A27.1.6<br>/A27.1.4<br>//**	19/06/2008	**No authorisation, because of no positive benefit-risk ratio**	**EMA evaluation**	**Number of clinical studies 01**	**Participants 0025-0049**	**No placebo control**		**No comparison with approved drugs**	**No special drug approval procedure**	**No consequences for patients in ongoing clinical trials or compassionate use programmes**	Ongoing clinical trials (at the time the application was withdrawn)	https://www.ema.europa.eu/en/documents/medicine-qa/questions-and-answers-withdrawal-marketing-application-spanidin_en.pdf	https://www.ema.europa.eu/en/documents/withdrawal-letter/withdrawal-letter-spanidin_en.pdf	**Orphan drugs**	6/19/08
Aflunov,	unknown	pre-pandemic influenza vaccine (H5N1) (surface antigen, inactivated, adjuvanted) (A/VietNam/1194/2004)	Novartis Vaccines and Diagnostics S.r.l.	Novartis	**Other medicines**	**J07BB**	**X Extension codes (viral vaccines)**	ICD-11 Chapter X	influenza A virus<br><br>Note:<br>pandemic vaccination protection, strain (type) H5N1	**other (N6)**	**/N6.U<br>//**	other (N6)	**/N6.U<br>//**	**Initial authorisation**	6/13/08	• open questions from the EMA (a17)<br>• concerns regarding the manufacturer's responses/information (a1)<br>• doubts about compliance with good clinical practice (gcp) (a30)<br>• doubts about efficacy<br>reason: lack of data to sufficiently demonstrate efficacy (a27.3)<br>• doubts about efficacy<br>reason: poor study conduct (a27.1.6)	**/A17<br>/A1<br>/A30<br>/A27.3<br>/A27.1.6<br>//**	16/06/2008	**No authorisation, because of no positive benefit-risk ratio**	**EMA evaluation**	**Number of clinical studies 01**	**Participants over 2000**	**No placebo control**		**Comparison with approved drugs**	**No special drug approval procedure**	**No consequences for patients in ongoing clinical trials or compassionate use programmes**	Ongoing clinical trials (at the time the application was withdrawn)	https://www.ema.europa.eu/en/documents/medicine-qa/questions-and-answers-withdrawal-marketing-application-aflunov_en.pdf	https://www.ema.europa.eu/en/documents/withdrawal-letter/withdrawal-letter-aflunov_en.pdf	**No orphan drugs**	6/16/08
Lenalidomide Celgene Europe,	lenalidomide	Lenalidomide	Celgene Europe Limited	Celgene	**Immunomodulators**	**L04AX04**	**02 Neoplasms**	ICD-11 Chapte	anemia in myelodysplastic syndrome<br><br>Note:<br>Use under certain conditions (e.g., partial deletion of chromosome number 5)	**Enzyme (Regulation)(N1.U)**<br><br>**Ligands (Regulation) (N9.U)**<br><br>**other proteins (Regulation)(N5.U)**	**/N1.U<br>/N9.U<br>/N5.U<br>//**	Enzyme<br>(Regulation)(N1)<br><br>Ligands (Regulation) (N9)<br><br>other proteins (Regulation)(N5)	**/N1.U<br>/N9.U<br>/N5.U<br>//**	**Initial authorisation**	5/30/08	• concerns regarding the manufacturer's responses/information (a1)<br>• doubts about efficacy<br>reason: poor study conduct (a27.1.6)<br>• doubts about assurance of patient safety<br>reason: lack of data and/or inadequate long-term follow-up (a21.3)<br>• doubts about efficacy<br>reason: poor study quality or flawed study design (lack of comparison with placebo and/or comparator/reference medicinal products) (a27.1.4)	**/A1<br>/A27.1.6<br>/A21.3<br>/A27.1.4<br>//**	4/06/2008	**No authorisation, because of no positive benefit-risk ratio**	**EMA evaluation**	**Number of clinical studies 01**	**Participants 0100-0149**	**No placebo control**		**No comparison with approved drugs**	**No special drug approval procedure**	**No consequences for patients in ongoing clinical trials or compassionate use programmes**	Ongoing clinical trials (at the time the application was withdrawn)	https://www.ema.europa.eu/en/documents/medicine-qa/questions-and-answers-withdrawal-marketing-application-lenalidomide-celgene-europe_en.pdf	https://www.ema.europa.eu/en/documents/withdrawal-letter/withdrawal-letter-lenalidomide-celgene-europe_en.pdf	**Orphan drugs**	6/4/08
Aquilda,	satavaptan	Satavaptan	Sanofi-Aventis	Sanofi	**Diuretics**	**Unknown**	**05 Endocrine diseases, nutritional or metabolic diseases**	ICD-11 Chapter 05	hyponatremia (euvolemic or hypervolemic)	**Receptors (Antagonism) (N4.A)**	**/N4.A<br>//**	Receptors<br>(GPCR, Antagonism) (N4.1.A)	**/N4.1.A<br>//**	**Initial authorisation**	5/23/08	• concerns regarding the manufacturer's responses/information (a1)<br>• doubts about assurance of patient safety<br>reason: lack of data and/or inadequate long-term follow-up (a21.3)<br>• doubts about efficacy<br>reason: lack of data to sufficiently demonstrate efficacy (a27.3)<br>• doubts about efficacy<br>reason: flawed study design (a27.2)<br>• doubts about assurance of patient safety<br>reason: side effects (e.g., heart) (a21.1)<br>• doubts about efficacy<br>reason: poor study quality (small number of participants) (a27.1.5)	**/A1<br>/A21.3<br>/A27.3<br>/A27.2<br>/A21.1<br>/A27.1.5<br>//**	28/05/2008	**No authorisation, because of no positive benefit-risk ratio**	**EMA evaluation**	**Number of clinical studies 05**	**Participants 0500-0999**	**Placebo controlled clinical studies**		**No comparison with approved drugs**	**No special drug approval procedure**	**No consequences for patients in ongoing clinical trials or compassionate use programmes**	Ongoing clinical trials (at the time the application was withdrawn)	https://www.ema.europa.eu/en/documents/medicine-qa/questions-and-answers-withdrawal-marketing-application-aquilda_en.pdf	https://www.ema.europa.eu/en/documents/withdrawal-letter/withdrawal-letter-aquilda_en.pdf	**No orphan drugs**	5/28/08
DuoPlavin,	clopidogrel/acetylsalicylic acid	Clopidogrel/acetylsalicylic acid	Sanofi Pharma Bristol-Myers Squibb	Sanofi Pharma Bristol-Myers Squibb	**Platelet aggregation inhibitors**	**B01AC06, B01AC0**	**X Extension codes (Anticoagulation)**	ICD-11 Chapter X	prevention of atherothrombotic events<br><br>Note:<br>Use under certain conditions, when clopidogrel and acetylsalicylic acid have already been taken separately.	**Enzyme (Antagonism)(N1.A)**<br><br>**Receptors (Antagonism)(N4.A)**	**/N1.A<br>/N4.A<br>//**	Receptors<br>(GPCR, Antagonism) (N4.1.A)<br><br>Enzyme<br>(Oxidoreductases, Antagonism)(N1.1.A)	**/N4.1.A<br>/N1.1.A<br>//**	**Initial authorisation**	5/23/08	• open questions from the EMA (a17)<br>• concerns regarding the manufacturer's responses/information (a1)<br>• open questions on bioavailability, bioequivalence, distribution and metabolism (for one or more topics) (a29)<br>• doubts about efficacy<br>reason: flawed study design (methods in bioequivalence study) (a27.2)	**/A17<br>/A1<br>/A29<br>/A27.2<br>//**	27/05/2008	**Authorisation recommended**	**EMA evaluation**	**Number of clinical studies 0**	**Participants 0**	**No placebo control**	**SD**	**Comparison with approved drugs**	**No special drug approval procedure**	**No consequences for patients in ongoing clinical trials or compassionate use programmes**	Ongoing clinical trials (at the time the application was withdrawn)	https://www.ema.europa.eu/en/documents/medicine-qa/questions-and-answers-withdrawal-marketing-application-duoplavin_en.pdf	https://www.ema.europa.eu/en/documents/withdrawal-letter/withdrawal-letter-duoplavin_en.pdf	**No orphan drugs**	5/27/08
DuoCover,	clopidogrel/acetylsalicylic acid	Clopidogrel/acetylsalicylic acid	Bristol-Myers Squibb Pharma EEIG	Bristol-Myers Squibb Pharma EEIG	**Platelet aggregation inhibitors**	**B01AC06, B01AC0**	**X Extension codes (Anticoagulation)**	ICD-11 Chapter X	prevention of atherothrombotic events<br><br>Note:<br>Use under certain conditions, when clopidogrel and acetylsalicylic acid have already been taken separately.	**Enzyme (Antagonism)(N1.A)**<br><br>**Receptors (Antagonism)(N4.A)**	**/N1.A<br>/N4.A<br>//**	Receptors<br>(GPCR, Antagonism) (N4.1.A)<br><br>Enzyme<br>(Oxidoreductases, Antagonism)(N1.1.A)	**/N4.1.A<br>/N1.1.A<br>//**	**Initial authorisation**	5/23/08	• open questions from the EMA (a17)<br>• concerns regarding the manufacturer's responses/information (a1)<br>• open questions on bioavailability, bioequivalence, distribution and metabolism (for one or more topics) (a29)<br>• doubts about efficacy<br>reason: flawed study design (methods in bioequivalence study) (a27.2)	**/A17<br>/A1<br>/A29<br>/A27.2<br>//**	27/05/2008	**Authorisation recommended**	**EMA evaluation**	**Number of clinical studies 0**	**Participants 0**	**No placebo control**	**SD**	**Comparison with approved drugs**	**No special drug approval procedure**	**No consequences for patients in ongoing clinical trials or compassionate use programmes**	Ongoing clinical trials (at the time the application was withdrawn)	https://www.ema.europa.eu/en/documents/medicine-qa/questions-and-answers-withdrawal-marketing-application-duocover_en.pdf	https://www.ema.europa.eu/en/documents/withdrawal-letter/withdrawal-letter-duocover_en.pdf	**No orphan drugs**	5/27/08

orBec,	beclomethasone dipropionate	Beclomethasone dipropionate	DOR BIOPHARMA UK Ltd	DOR BIOPHARMA UK Ltd	**Immunosuppressants (no monoclonal antibodies)**	**Unknown**	**04 Diseases of the immune system**	ICD-11 Chapter 04	graft vs host disease (gastrointestinal)	**Receptors (Agonism) (N4.B)**	**/N4.B //**	Receptors (nuclear receptors, Agonism) (N4.4.1.B)	**/N4.4.B //**	**Initial authorisation**		5/22/08	• open questions from the EMA (a17)<br>• concerns regarding the manufacturer's responses/information (a1)<br>• doubts about efficacy<br>reason: lack of data to sufficiently demonstrate efficacy (a27.3)<br>• doubts about efficacy<br>reason: poor study quality (selection of participants) (a27.1.7)<br>• open questions on bioavailability, bioequivalence, distribution and metabolism (for one or more topics) (a29)	**/A17 /A1 /A27.3 /A27.1.7 /A29 //**	23/05/2008	**No authorisation, because of no positive benefit-risk ratio**	**EMA evaluation**	**Number of clinical studies 01**	**Participants 0100-0149**	**Placebo controlled clinical studies**	**SD**	**No comparison with approved drugs**	**No special drug approval procedure**	**No consequences for patients in ongoing clinical trials or compassionate use programmes**	Ongoing clinical trials (at the time the application was withdrawn)	https://www.ema.europa.eu/en/documents/medicine-qa/questions-and-answers-withdrawal-marketing-application-orbec_en.pdf	https://www.ema.europa.eu/en/documents/withdrawal-letter/withdrawal-letter-orbec_en.pdf	**Orphan drugs**	5/23/08
Tygacil,	tigecycline	Tigecycline	Pfizer Europe MA EEIG	Pfizer	**antibacterial drugs**	**J01AA12**	**12 Respiratory diseases**	ICD-11 Chapter 12	pneumonia (community-acquired)<br><br>already approved indications: complicated infections of the skin and soft tissues, complicated infections of the abdomen	**other proteins (Antagonism/Binding) (N5.A)**	**/N5.A //**	other proteins (bacterial proteins / bacterial protein synthesis, Antagonism)(N5.9.A)	**/N5.9.A //**	**Post-authorisation**	4/24/06	4/22/08	• open questions from the EMA (a17)<br>• concerns regarding the manufacturer's responses/information (a1)<br>• doubts about assurance of patient safety<br>reason: side effects (a21.1)<br>• doubts about efficacy<br>reason: patients in the study not compatible with the target indication (a27.4)<br>• doubts about efficacy<br>reason: other reasons (concerns about the number of deaths, sufficient efficacy questionable) (a27)	**/A17 /A1 /A21.1 /A27.4 /A27. //**	24/04/2008	**No authorisation, because of no positive benefit-risk ratio**	**EMA evaluation**	**Number of clinical studies 02**	**Participants 0500-0999**	**No placebo control**	**SD**	**Comparison with approved drugs**	**No special drug approval procedure**	**No consequences for patients in ongoing clinical trials or compassionate use programmes**	Ongoing clinical trials (at the time the application was withdrawn)	https://www.ema.europa.eu/en/documents/medicine-qa/questions-and-answers-withdrawal-application-change-marketing-authorisation-tygacil_en.pdf	https://www.ema.europa.eu/en/documents/other/withdrawal-letter-tygacil_en.pdf	**No orphan drugs**	4/24/08
Evoltra,	clofarabine	Clofarabine	Sanofi B.V.	Sanofi	**Cytostatics**	**L01BB06**	**02 Neoplasms/**<br><br>**X Extension codes (Leukemias)**	ICD-11 Chapter 02<br><br>ICD-11 Chapter X	acute myeloid leukemia (AML) (from age 65, use under special conditions)<br><br>already approved indications: acute lymphoblastic leukemia in children (ALL) (use after at least two failed treatment attempts or a recurrence and no prospect of other treatment success)	**Enzyme (Antagonism)(N1.A)**	**/N1.A //**	Enzyme (Oxidoreductases, Antagonism)(N1.1.A)	**/N1.1.A //**	**Post-authorisation**	5/29/06	3/18/08	• open questions from the EMA (a17)<br>• concerns regarding the manufacturer's responses/information (a1)<br>• doubts about benefit (a18)<br>• doubts about efficacy<br>reason: poor study quality or flawed study design (lack of comparison with placebo and/or comparator/reference medicinal products) (a27.1.4)<br>• doubts about efficacy<br>reason: patients in the study not compatible with the target indication (a27.4)<br>• doubts about efficacy<br>reason: lack of data to sufficiently demonstrate efficacy (a27.3)<br>• doubts about assurance of patient safety<br>reason: side effects (kidney) (a21.1)	**/A17 /A1 /A18 /A27.1.4 /A27.4 /A27.3 /A21.1 //**	19/03/2008	**No authorisation, because of no positive benefit-risk ratio**	**EMA evaluation**	**Number of clinical studies 01**	**Participants 0050-0074**	**No placebo control**		**No comparison with approved drugs**	**No special drug approval procedure**	**No consequences for patients in ongoing clinical trials or compassionate use programmes**	Ongoing clinical trials (at the time the application was withdrawn)	https://www.ema.europa.eu/en/documents/medicine-qa/questions-and-answers-withdrawal-change-marketing-authorisation-evoltra_en.pdf	https://www.ema.europa.eu/en/documents/press-release/bioenvision-ltd-withdraws-its-application-extension-indication-evoltra-clofarabine_en.pdf	**Orphan drugs**	3/19/08
Kiacta,	eprodisate sodium	Eprodisat Sodium	Neurochem Luxco II SARL	Neurochem Luxco II SARL	**Other medicines**	**Unknown**	**05 Endocrine diseases, nutritional or metabolic diseases**	ICD-11 Chapter 05	amyloidosis (AA-amyloidosis)	**other proteins (N5.U)**	**/N5.U //**	other proteins (N5)	**/N5.U //**	**Initial authorisation**		3/13/08	• concerns regarding the manufacturer's responses/information (a1)<br>• doubts about efficacy<br>reason: poor study quality (data analysis) (a27.1.3)<br>• doubts about efficacy<br>reason: lack of data to sufficiently demonstrate efficacy (a27.3)	**/A1 /A27.1.3 /A27.3 //**	17/03/2008	**No authorisation, because of no positive benefit-risk ratio**	**EMA evaluation**	**Number of clinical studies 01**	**Participants 0150-0199**	**Placebo controlled clinical studies**		**No comparison with approved drugs**	**No special drug approval procedure**	**No consequences for patients in ongoing clinical trials or compassionate use programmes**	Ongoing clinical trials (at the time the application was withdrawn)	https://www.ema.europa.eu/en/documents/medicine-qa/questions-and-answers-withdrawal-marketing-application-kiacta_en.pdf	https://www.ema.europa.eu/en/documents/withdrawal-letter/withdrawal-letter-kiacta_en.pdf	**Orphan drugs**	3/17/08
Pristiqs,	desvenlafaxine	Desvenlafaxine	Wyeth Europa Ltd	Wyeth	**Serotonin-norepinephrine reuptake inhibitors (SNRI)**	**N06AX23**	**16 Diseases of the urogenital system**	ICD-11 Chapter 16	hot flashes associated with menopause	**Transporter (Antagonism)(N3.A)**	**/N3.A //**	Transporter (SLC-Transporters, Antagonism)(N3.1.A)	**/N3.1.A //**	**Initial authorisation**		3/10/08	• open questions from the EMA (a17)<br>• concerns regarding the manufacturer's responses/information (a1)<br>• doubts about efficacy<br>reason: lack of data to sufficiently demonstrate efficacy (a27.3)<br>• doubts about assurance of patient safety<br>reason: adverse reactions (a21.1)	**/A17 /A1 /A27.3 /A21.1 //**	12/03/2008	**No authorisation, because of no positive benefit-risk ratio**	**EMA evaluation**	**Number of clinical studies 03**	**Participants 1000-1999**	**Placebo controlled clinical studies**		**Comparison with approved drugs**	**No special drug approval procedure**	**No consequences for patients in ongoing clinical trials or compassionate use programmes**	No ongoing clinical trials (at the time the application was withdrawn)	https://www.ema.europa.eu/en/documents/medicine-qa/questions-and-answers-withdrawal-marketing-application-pristiqs_en.pdf	https://www.ema.europa.eu/en/documents/withdrawal-letter/withdrawal-letter-pristiqs_en.pdf	**No orphan drugs**	3/12/08
Zavesca,	miglustat	Miglustat	Janssen-Cilag International NV	Janssen Pharmaceutical Companies of Johnson & Johnson	**Other medicines**	**A16AX06**	**08 Diseases of the nervous system**	ICD-11 Chapter 08	neurological manifestations in patients with Niemann-Pick disease type C<br><br>already approved indications: Gaucher's disease type I (mild to moderate)	**Enzyme (Antagonism)(N1.A)**	**/N1.A //**	Enzyme (Transferases, Antagonism)(N1.2.A)	**/N1.2.A //**	**Post-authorisation**	11/20/02	2/19/08	• concerns regarding the manufacturer's responses/information (a1)<br>• doubts about efficacy<br>reason: lack of data to sufficiently demonstrate efficacy (a27.3)<br>• doubts about assurance of patient safety<br>reason: adverse reactions (stomach, intestines, weight loss and thrombocytopenia) (a21.1)<br>• doubts about equivalent/improved efficacy compared with the eu reference medicinal product (a25)<br>• doubts about efficacy<br>reason: inadequate study design (assessment of eye movements to evaluate efficacy) (a27.2)	**/A1 /A27.3 /A21.1 /A25 /A27.2 //**	25/02/2008	**No authorisation, because of no positive benefit-risk ratio**	**EMA evaluation**	**Number of clinical studies 01**	**Participants 0025-0049**	**No placebo control**	**SD**	**Comparison with approved drugs**	**No special drug approval procedure**	**No consequences for patients in ongoing clinical trials or compassionate use programmes**	Ongoing clinical trials (at the time the application was withdrawn)	https://www.ema.europa.eu/en/documents/medicine-qa/questions-and-answers-withdrawal-application-change-marketing-authorisation-zavesca_en.pdf	https://www.ema.europa.eu/en/documents/other/withdrawal-letter-zavesca_en.pdf	**Orphan drugs**	2/25/08
NutropinAq,	somatropin	Somatropin	Ipsen Pharma	Ipsen Pharma	**Other medicines**	**H01AC01**	**05 Endocrine diseases, nutritional or metabolic diseases**<br><br>**21 Symptoms, signs or clinical findings not elsewhere classified (short stature)**	ICD-11 Chapter 05<br><br>ICD-11 Chapter 21	severe idiopathic growth disorders in children (long-term treatment)<br><br>already approved indications: growth disorders (due to growth hormone deficiency), growth disorders (in Turner syndrome), growth disorders (in chronic kidney insufficiency), adults with growth hormone deficiency (as replacement therapy)	**Receptors (Agonism) (N4.B)**	**/N4.B //**	Receptors (catalytic receptors, Agonism) (N4.3.B)		**Post-authorisation**	2/15/01	1/24/08	• concerns regarding the manufacturer's responses/information (a1)<br>• doubts about assurance of patient safety<br>reason: adverse reactions (risk of tumours and diabetes) (a21.1)<br>• doubts about benefit (benefit too marginal) (a18)<br>• doubts about efficacy<br>reason: lack of data to sufficiently demonstrate efficacy (a27.3)	**/A1 /A21.1 /A27.3 /A18 //**	28/01/2008	**No extension of authorisation, because of no positive benefit-risk ratio**	**EMA evaluation**	**Number of clinical studies 01**	**Participants 0100-0149**	**No placebo control**		**No comparison with approved drugs**	**No special drug approval procedure**	**No consequences for patients in ongoing clinical trials or compassionate use programmes**	No ongoing clinical trials (at the time the application was withdrawn)	https://www.ema.europa.eu/en/documents/medicine-qa/questions-and-answers-withdrawal-application-change-marketing-authorisation-nutropinaq_en.pdf	https://www.ema.europa.eu/en/documents/other/withdrawal-letter-nutropinaq_en.pdf	**No orphan drugs**	1/28/08
Insulin Human 30/70 Mix Marvel,	insulin human	Insulin human	Marvel Life Sciences Ltd.	Marvel Life Sciences Ltd.	**Human insulin**	**A10AB01**	**05 Endocrine diseas**	ICD-11 Chapter 05	diabetes mellitus (including newly diagnosed diabetes in pregnant women)	**other (N6)**<br><br>**Receptors (Agonism) (N4.B)**	**/N4.B /N6.U //**	other (N6)<br><br>Receptors (catalytic receptors, Activation)(N4.3.B)	**/N6.U /N4.3.B //**	**Initial authorisation**		12/20/07	• open questions from the EMA (a17)<br>• concerns regarding the manufacturer's responses/information (a1)<br>• doubts about equivalent/improved efficacy compared with the eu reference medicinal product (with respect to the bioequivalence study and the pivotal study) (a25)<br>• concerns regarding medicinal product quality (manufacturing process) (a3.1)	**/A17 /A1 /A25 /A3.1 //**	16/01/2008	**No authorisation, because of no positive benefit-risk ratio**	**EMA evaluation**	**Number of clinical studies 01**	**Participants 0500-0999**	**No placebo control**	**SDB**	**Comparison with approved drugs**	**Drug application for biosimilars**	**No consequences for patients in ongoing clinical trials or compassionate use programmes**	No ongoing clinical trials (at the time the application was withdrawn)	https://www.ema.europa.eu/en/documents/medicine-qa/questions-and-answers-withdrawal-marketing-authorisation-application-insulin-human-rapid-marvel-insulin-human-long-marvel-and-insulin-human-3070-mix-marvel_en.pdf	https://www.ema.europa.eu/en/documents/withdrawal-letter/withdrawal-letter-insulin-human-rapid-marvel-insulin-human-long-marvel-insulin-human-30-70-mix-marvel_en.pdf	**No orphan drugs**	1/16/08
Insulin Human Rapid Marvel,	insulin human	Insulin human	Marvel Life Sciences Ltd.	Marvel Life Sciences Ltd.	**Human insulin**	**A10AB01**	**05 Endocrine diseas**	ICD-11 Chapter 05	diabetes mellitus (including newly diagnosed diabetes in pregnant women)	**other (N6)**<br><br>**Receptors (Agonism) (N4.B)**	**/N4.B /N6.U //**	other (N6)<br><br>Receptors (catalytic receptors, Activation)(N4.3.B)	**/N6.U /N4.3.B //**	**Initial authorisation**		12/20/07	• open questions from the EMA (a17)<br>• concerns regarding the manufacturer's responses/information (a1)<br>• doubts about equivalent/improved efficacy compared with the eu reference medicinal product (with respect to the bioequivalence study and the pivotal study) (a25)<br>• concerns regarding medicinal product quality (manufacturing process) (a3.1)	**/A17 /A1 /A25 /A3.1 //**	16/01/2008	**No authorisation, because of no positive benefit-risk ratio**	**EMA evaluation**	**Number of clinical studies 01**	**Participants 0500-0999**	**No placebo control**	**SDB**	**Comparison with approved drugs**	**Drug application for biosimilars**	**No consequences for patients in ongoing clinical trials or compassionate use programmes**	No ongoing clinical trials (at the time the application was withdrawn)	https://www.ema.europa.eu/en/documents/medicine-qa/questions-and-answers-withdrawal-marketing-authorisation-application-insulin-human-rapid-marvel-insulin-human-long-marvel-and-insulin-human-3070-mix-marvel_en.pdf	https://www.ema.europa.eu/en/documents/withdrawal-letter/withdrawal-letter-insulin-human-rapid-marvel-insulin-human-long-marvel-insulin-human-30-70-mix-marvel_en.pdf	**No orphan drugs**	1/16/08
Insulin Human Long Marvel,	insulin human	Insulin human	Marvel Life Sciences Ltd.	Marvel Life Sciences Ltd.	**Human insulin**	**A10AB01**	**05 Endocrine diseas**	ICD-11 Chapter 05	diabetes mellitus (including newly diagnosed diabetes in pregnant women)	**other (N6)**<br><br>**Receptors (Agonism) (N4.B)**	**/N4.B /N6.U //**	other (N6)<br><br>Receptors (catalytic receptors, Activation)(N4.3.B)	**/N6.U /N4.3.B //**	**Initial authorisation**		12/20/07	• open questions from the EMA (a17)<br>• concerns regarding the manufacturer's responses/information (a1)<br>• doubts about equivalent/improved efficacy compared with the eu reference medicinal product (with respect to the bioequivalence study and the pivotal study) (a25)<br>• concerns regarding medicinal product quality (manufacturing process) (a3.1)	**/A17 /A1 /A25 /A3.1 //**	16/01/2008	**No authorisation, because of no positive benefit-risk ratio**	**EMA evaluation**	**Number of clinical studies 01**	**Participants 0500-0999**	**No placebo control**	**SDB**	**Comparison with approved drugs**	**Drug application for biosimilars**	**No consequences for patients in ongoing clinical trials or compassionate use programmes**	No ongoing clinical trials (at the time the application was withdrawn)	https://www.ema.europa.eu/en/documents/medicine-qa/questions-and-answers-withdrawal-marketing-authorisation-application-insulin-human-rapid-marvel-insulin-human-long-marvel-and-insulin-human-3070-mix-marvel_en.pdf	https://www.ema.europa.eu/en/documents/withdrawal-letter/withdrawal-letter-insulin-human-rapid-marvel-insulin-human-long-marvel-insulin-human-30-70-mix-marvel_en.pdf	**No orphan drugs**	1/16/08
Sinerem,	unknown	Stabilized superparamagnetic iron oxide nanoparticles with dextran and sodium citrate	Guerbet	Guerbet	**Other medicines**	**Unknown**	**X Extension codes (Medicines used in diagnostic tests, not elsewhere classified)**<br><br>**02 Neoplasms**	ICD-11 Chapter 02<br><br>ICD-11 Chapter X	magnetic resonance imaging (contrast agent for visualizing lymph nodes to assess the spreading of primary tumors in the lower abdomen, e.g., prostate, bladder or uterus)	**other (N6)**	**/N6.U //**	other (N6)	**/N6.U //**	**Initial authorisation**		12/13/07	• open questions from the EMA (a17)<br>• concerns regarding the manufacturer's responses/information (a1)<br>• doubts about efficacy<br>reason: lack of data to sufficiently demonstrate efficacy (a27.3)<br>• doubts about benefit (a18)	**/A17 /A1 /A27.3 /A18 //**	14/12/2007	**No authorisation, because of no positive benefit-risk ratio**	**EMA evaluation**	**Number of clinical studies 01**	**Participants 0200-0299**	**No placebo control**		**Comparison with approved drugs**	**No special drug approval procedure**	**No consequences for patients in ongoing clinical trials or compassionate use programmes**	Ongoing clinical trials (at the time the application was withdrawn)	https://www.ema.europa.eu/en/documents/medicine-qa/questions-and-answers-withdrawal-marketing-application-sinerem_en.pdf	https://www.ema.europa.eu/en/documents/withdrawal-letter/withdrawal-letter-sinerem_en.pdf	**No orphan drugs**	12/14/07
Zometa,	zoledronic acid	Zoledronic acid	Phoenix Labs Unlimited Company (Erweiterung der Zulassung durch Novartis Europharm Ltd. beantragt)	Phoenix Labs Unlimited Company (Erweiterung der Zulassung durch Novartis Europharm Ltd. beantragt)	**Bisphosphonates**	**M05BA08**	**15 Diseases of the musculoskeletal system or connective tissue**	ICD-11 Chapter 15	prevention of fractures and bone loss in postmenopausal women with early-stage breast cancer (when treated with aromatase inhibitors)<br><br>already approved indications: prevention of fractures in cancer patients, treatment of tumor-induced hypercalcemia	**Enzyme (Antagonism)(N1.A)**	**/N1.A //**	Enzyme (Transferases, Antagonism)(N1.2.A)	**/N1.2.A //**	**Post-authorisation**	3/20/01	11/15/07	• open questions from the EMA (a17)<br>• concerns regarding the manufacturer's responses/information (a1)<br>• doubts about efficacy<br>reason: inadequate study design (fractures were not a direct endpoint) (a27.2)<br>• concerns regarding the recommended dose (a6)<br>• doubts about efficacy<br>reason: lack of data to sufficiently demonstrate efficacy (a27.3)	**/A17 /A1 /A27.2 /A27.3 /A6 //**	20/11/2007	**No extension of authorisation, because of no positive benefit-risk ratio**	**EMA evaluation**	**Number of clinical studies 02**	**Participants 1000-1999**	**No placebo control**		**No comparison with approved drugs**	**No special drug approval procedure**	**No consequences for patients in ongoing clinical trials or compassionate use programmes**	Ongoing clinical trials (at the time the application was withdrawn)	https://www.ema.europa.eu/en/documents/medicine-qa/questions-and-answers-withdrawal-application-change-marketing-authorisation-zometa_en.pdf	https://www.ema.europa.eu/en/documents/other/withdrawal-letter-zometa_en.pdf	**No orphan drugs**	11/20/07

Gastromotal,	1-13 C-caprylic acid	1-13 C-Caprylic acid	INFAI, Institut für biomedizinische Analytik NMR-Imaging GmbH	INFAI, Institut für biomedizinische Analytik NMR-Imaging GmbH	**Other medicines**	**Unknown**	**X Extension codes (Medicines used in diagnostic tests, not elsewhere classified)**<br><br>**13 Diseases of the digestive system**	ICD-11 Chapter X<br><br>ICD-11 Chapter 13	in vivo diagnosis of the half-life of solid-phase gastric emptying in gastric motility disorders	**Transporter (N3.U)**	**/N3.U<br>//**	Transporter (SLC-Transporters)(N3.1)	**/N3.1.U<br>//**	**Initial authorisation**		11/5/07	• concerns regarding the manufacturer's responses/information (a1)<br>• doubts about efficacy<br>reason: lack of data to sufficiently demonstrate efficacy (a27.3)<br>• doubts about equivalent/improved efficacy compared with the eu reference medicinal product (standard test 99m tc scintigraphy) (a25)<br>• doubts about benefit (a18)	**/A1<br>/A27.3<br>/A25<br>/A18<br>//**	16/11/2007	**No authorisation, because of no positive benefit-risk ratio**	**EMA evaluation**	**Number of clinical studies 02**	**Participants 0300-0499**	**No placebo control**		**Comparison with approved drugs**	**No special drug approval procedure**	**No consequences for patients in ongoing clinical trials or compassionate use programmes**	Ongoing clinical trials (at the time the application was withdrawn)	https://www.ema.europa.eu/en/documents/medicine-qa/questions-and-answers-withdrawal-marketing-application-gastromotal_en.pdf	https://www.ema.europa.eu/en/documents/withdrawal-letter/withdrawal-letter-gastromotal_en.pdf	**No orphan drugs**	11/16/07
Garenoxacin mesylate,	garenoxacin mesylate	Garenoxacin	Schering-Plough Europe	Schering-Plough Europe	**antibacterial drugs**	**J01MA19**	**01 Certain infectious or parasitic diseases**<br><br>**12 Respiratory diseases**	ICD-11 Chapter 01<br><br>ICD-11 Chapter 12	acute bacterial exacerbation of chronic bronchitis, acute bacterial sinusitis, community-acquired pneumonia, infections of the skin and subcutaneous tissue (including foot infections in patients<br>with diabetes), complicated infections of the abdomen, (including postoperative infections and acute pelvic infections)	**other proteins (Antagonism/Binding) (N5.A)**	**/N5.A<br>//**	other proteins (bacterial proteins / bacterial protein synthesis, Antagonism)(N5.9.A)	**/N5.9.A<br>//**	**Initial authorisation**		7/25/07	• open questions from the EMA (a17)<br>• concerns regarding the manufacturer's responses/information (a1)<br>• doubts about efficacy<br>reason: lack of data to sufficiently demonstrate efficacy (with respect to part of the infections/requested indications) (a27.3)<br>• doubts about assurance of patient safety<br>reason: adverse reactions (e.g. hypotension, effects on blood glucose) (a21.1)	**/A17<br>/A1<br>/A27.3<br>/A21.1<br>//**	26/07/2007	**No authorisation, because of no positive benefit-risk ratio**	**EMA evaluation**	**Number of clinical studies 19**	**Participants over 2000**	**No placebo control**		**Comparison with approved drugs**	**No special drug approval procedure**	**No consequences for patients in ongoing clinical trials or compassionate use programmes**	Ongoing clinical trials (at the time the application was withdrawn)	https://www.ema.europa.eu/en/documents/medicine-qa/questions-and-answers-withdrawal-marketing-application-garenoxacin-mesylate_en.pdf	https://www.ema.europa.eu/en/documents/withdrawal-letter/withdrawal-letter-garenoxacin-mesylate_en.pdf	**No orphan drugs**	7/26/07
Retisert,	fluocinolone acetonide	Fluocinolone acetonide	Bausch Lomb Ireland	Bausch Lomb Ireland	**Immunosuppressants (no monoclonal antibodies)**	**S01BA15**	**09 Diseases of the visual system**	ICD-11 Chapter 09	chronic non-infectious uveitis (in the posterior eye section)	**Enzyme (Antagonism)(N1.A)**<br><br>**Receptors (Agonism)(N4.B)**	**/N1.A<br>/N4.B<br>//**	Receptors (nuclear receptors, Agonism) (N4.4.B)<br><br>Enzyme (Hydrolases, Antagonism)(N1.3.A)	**/N4.4.B<br>/N1.3.A<br>//**	**Initial authorisation**		7/16/07	• open questions from the EMA (a17)<br>• concerns regarding the manufacturer's responses/information (a1)<br>• doubts about equivalent/improved efficacy compared with the eu reference medicinal product (a25)<br>• doubts about benefit (a18)<br>• doubts about efficacy<br>reason: lack of data to sufficiently demonstrate efficacy (a27.3)<br>• doubts about efficacy<br>reason: inadequate study design (primary efficacy indicator questionable) (a27.2)<br>• doubts about assurance of patient safety<br>reason: adverse reactions (eye pain, cataract, intraocular pressure) (a21.1)<br>• concerns regarding medicinal product quality (a3)	**/A17<br>/A1<br>/A25<br>/A27.3<br>/A18<br>/A27.2<br>/A21.1<br>/A3.<br>//**	23/07/2007	**No authorisation, because of no positive benefit-risk ratio**	**EMA evaluation**	**Number of clinical studies 01**	**Participants 0100-0149**	**No placebo control**		**Comparison with approved drugs**	**No special drug approval procedure**	**No consequences for patients in ongoing clinical trials or compassionate use programmes**	No ongoing clinical trials (at the time the application was withdrawn)	https://www.ema.europa.eu/en/documents/medicine-qa/questions-and-answers-withdrawal-marketing-application-retisert_en.pdf	https://www.ema.europa.eu/en/documents/withdrawal-letter/withdrawal-letter-retisert_en.pdf	**Orphan drugs**	7/23/07
Cerepro,	unknown	adenoviral herpes simplex virus thymidine kinase gene	Ark Therapeutics	Ark Therapeutics	**Other medicines**	**Unknown**	**02 Neoplasms**	ICD-11 Chapte	high-grade gliomas (use if surgery is possible and in combination with ganciclovir sodium)	**other (N6)**	**/N6.U<br>//**	other (N6)	**/N6.U<br>//**	**Initial authorisation**		7/13/07	• concerns regarding the manufacturer's responses/information (a1)<br>• doubts about efficacy<br>reason: poor study quality (small number of participants) (a27.1.5)<br>• doubts about benefit (a18)<br>• doubts about efficacy<br>reason: lack of data to sufficiently demonstrate efficacy (a27.3)<br>• doubts about assurance of patient safety<br>reason: lack of data and/or inadequate long-term follow-up (a21.3)<br>• doubts about efficacy<br>reason: poor study quality (conduct) (a27.1.6)	**/A1<br>/A27.1.5<br>/A18<br>/A27.3<br>/A21.3<br>/A27.1.6<br>//**	20/07/2007	**No authorisation, because of no positive benefit-risk ratio**	**EMA evaluation**	**Number of clinical studies 01**	**Participants 0025-0049**	**No placebo control**		**Comparison with approved drugs**	**No special drug approval procedure**	**No consequences for patients in ongoing clinical trials or compassionate use programmes**	Ongoing clinical trials (at the time the application was withdrawn)	https://www.ema.europa.eu/en/documents/medicine-qa/questions-and-answers-withdrawal-marketing-application-cerepro_en.pdf	https://www.ema.europa.eu/en/documents/withdrawal-letter/withdrawal-letter-cerepro_en.pdf	**Orphan drugs**	7/20/07
Voraxaze,	glucarpidase	Glucarpidase	Protherics PLC	Protherics PLC	**Other medicines**	**V03AF09**	**23 External causes of morbidity or mortality**	ICD-11 Chapter 23	prevention/treatment of methotrexate toxicity<br>(adjuvant treatment in patients who have already received the drug methotrexate to prevent or treat the toxic effects of methotrexate)	**other (N6)**	**/N6.U<br>//**	other (N6)	**/N6.U<br>//**	**Initial authorisation**		5/21/07	• open questions from the EMA (a17)<br>• concerns regarding the manufacturer's responses/information (a1)<br>• concerns regarding medicinal product quality (manufacturing process) (a3.1)<br>• concerns regarding medicinal product quality (contaminants and/or concerns regarding sterilisation) (concerns regarding assurance of purity) (a3.3)<br>• doubts about assurance of patient safety<br>reason: interactions with other medicinal products (folinic acid) (a21.2)	**/A17<br>/A1<br>/A3.1<br>/A3.3<br>/A21.2<br>//**	29/05/2007	**No authorisation, because of no positive benefit-risk ratio**	**EMA evaluation**	**Number of clinical studies 02**	**Participants 0200-0299**	**No placebo control**		**No comparison with approved drugs**	**No special drug approval procedure**	**No consequences for patients in ongoing clinical trials or compassionate use programmes**	Ongoing clinical trials (at the time the application was withdrawn)	https://www.ema.europa.eu/en/documents/medicine-qa/questions-and-answers-withdrawal-marketing-application-voraxaze_en.pdf	https://www.ema.europa.eu/en/documents/withdrawal-letter/withdrawal-letter-voraxaze_en.pdf	**Orphan drugs**	5/29/07
Vitragan,	hyaluronidase (ovine)	Hyaluronidase (from sheep)	ISTA Pharma Limited	ISTA Pharma Limited	**Other medicines**	**B06AA03**	**09 Diseases of the visual system**	ICD-11 Chapter 09	vitreous hemorrhages	**other (N6)**	**/N6.U<br>//**	other (N6)	**/N6.U<br>//**	**Initial authorisation**		4/25/07	• open questions from the EMA (a17)<br>• concerns regarding the manufacturer's responses/information (a1)<br>• doubts about benefit (a18)<br>• doubts about efficacy<br>reason: lack of data to sufficiently demonstrate efficacy (a27.3)<br>• doubts about assurance of patient safety<br>reason: lack of data and/or inadequate long-term follow-up (a21.3)<br>• doubts about equivalent/improved efficacy compared with the eu reference medicinal product (comparison with other treatment options) (a25)<br>• concerns regarding medicinal product quality (consistency between production batches) (a3)	**/A17<br>/A1<br>/A18<br>/A27.3<br>/A21.3<br>/A25<br>/A3.<br>//**	30/04/2007	**No authorisation, because of no positive benefit-risk ratio**	**EMA evaluation**	**Number of clinical studies 02**	**Participants 1000-1999**	**Placebo controlled clinical studies**		**No comparison with approved drugs**	**No special drug approval procedure**	**No consequences for patients in ongoing clinical trials or compassionate use programmes**	No ongoing clinical trials (at the time the application was withdrawn)	https://www.ema.europa.eu/en/documents/medicine-qa/questions-and-answers-withdrawal-marketing-application-vitragan_en.pdf	https://www.ema.europa.eu/en/documents/withdrawal-letter/withdrawal-letter-vitragan_en.pdf	**No orphan drugs**	4/30/07
Iplex,	mecasermin rinfabate	Mecasermin Rinfabat	Insmed Europe Ltd	Insmed	**Other medicines**	**H01AC05**	**05 Endocrine diseases, nutritional or metabolic diseases**	ICD-11 Chapter 05	short statured children<br>(primary growth hormone resistance or patients with<br>growth hormone gene deletion who have developed neutralizing antibodies against growth hormone)	**Receptors (Agonism) (N4.B)**	**/N4.B<br>//**	Receptors (catalytic receptors, Agonism) (N4.3.B)	**/N4.3.B<br>//**	**Initial authorisation**		3/26/07	• open questions from the EMA (a17)<br>• concerns regarding the manufacturer's responses/information (a1)<br>• concerns regarding medicinal product quality (manufacturing process) (a3.1)<br>• doubts about efficacy<br>reason: lack of data to sufficiently demonstrate efficacy (a27.3)<br>• doubts about efficacy<br>reason: poor study quality (selection of participants) (a27.1.7)	**/A17<br>/A1<br>/A3.1<br>/A27.3<br>/A27.1.7<br>//**	26/03/2007	**No authorisation, because of no positive benefit-risk ratio**	**Marketing strategy**	**Number of clinical studies 01**	**Participants 0025-0049**	**No placebo control**		**No comparison with approved drugs**	**No special drug approval procedure**	**No consequences for patients in ongoing clinical trials or compassionate use programmes**	Ongoing clinical trials (at the time the application was withdrawn)	https://www.ema.europa.eu/en/documents/medicine-qa/questions-and-answers-withdrawal-marketing-application-iplex_en.pdf	https://www.ema.europa.eu/en/documents/withdrawal-letter/withdrawal-letter-iplex_en.pdf	**Orphan drugs**	3/26/07
Arxxant,	ruboxistaurin	Ruboxistaurin	Eli Lilly Nederland B.V.	Eli Lilly Nederland B.V.	**Other medicines**	**Unknown**	**09 Diseases of the visual system**	ICD-11 Chapter 09	diabetic retinopathy (in adults with moderate to<br>severe non-proliferative retinopathy)	**Enzyme (Antagonism)(N1.A)**	**/N1.A<br>//**	Enzyme (Transferases, Antagonism)(N1.2.A)	**/N1.2.A<br>//**	**Initial authorisation**		3/13/07	• open questions from the EMA (a17)<br>• concerns regarding the manufacturer's responses/information (a1)<br>• doubts about efficacy<br>reason: lack of data to sufficiently demonstrate efficacy (a27.3)<br>• doubts about assurance of patient safety<br>reason: adverse reactions (cardiac arrhythmias) (a21.1)	**/A17<br>/A1<br>/A27.3<br>/A21.1<br>//**	15/03/2007	**No authorisation, because of no positive benefit-risk ratio**	**EMA evaluation**	**Number of clinical studies 01**	**Participants 0500-0999**	**Placebo controlled clinical studies**		**No comparison with approved drugs**	**No special drug approval procedure**	**No consequences for patients in ongoing clinical trials or compassionate use programmes**	Ongoing clinical trials (at the time the application was withdrawn)	https://www.ema.europa.eu/en/documents/medicine-qa/questions-and-answers-withdrawal-marketing-application-arxxant_en.pdf	https://www.ema.europa.eu/en/documents/withdrawal-letter/withdrawal-letter-arxxant_en.pdf	**No orphan drugs**	3/15/07
Synordia,	fenofibrate/metformin hydrochloride	Fenofibrate/ Metformin Hydrochloride	Fournier Laboratories Ireland Ltd.	Fournier Laboratories Ireland Ltd.	**Other antidiabetics**	**C10AB05, A10BA0**	**05 Endocrine diseases, nutritional or metabolic diseases**	ICD-11 Chapter 05	diabetes mellitus (type 2)<br><br>Note:<br>Use in patients who are already taking fenofibrate and metformin separately	**other (N6)**<br><br>**Receptors (Agonism) (N4.B)**	**/N4.B<br>/N6.U<br>//**	(Transferases, Antagonism)(N1.2.A)	**/N4.4.B<br>/N6.U<br>//**	**Initial authorisation**		12/7/06	• not present (documents have not yet been reviewed or recommendations not yet published) (a14.1)<br><br>note:<br>documents had not been fully reviewed at the time of withdrawal of the marketing authorisation application.	**/A14.1<br>//**	8/12/2006	**None (documents have not yet been evaluated)**	**Suspected EMA evaluation**	**Number of clinical studies 02**	**Participants 1000-1999**	**No placebo control**		**Comparison with approved drugs**	**No special drug approval procedure**	**No consequences for patients in ongoing clinical trials or compassionate use programmes**	Ongoing clinical trials (at the time the application was withdrawn)	https://www.ema.europa.eu/en/documents/medicine-qa/questions-and-answers-withdrawal-marketing-application-synordia_en.pdf	https://www.ema.europa.eu/en/documents/withdrawal-letter/withdrawal-letter-synordia_en.pdf	**No orphan drugs**	12/8/06
Glivec,	imatinib	Imatinib	Novartis Europharm Limited	Novartis	**Kinase inhibitors**	**L01EA01**	**02 Neoplasms**	ICD-11 Chapte	systemic mastocytosis (in adults)<br><br><br>already approved indications:<br>chronic myeloid leukemia (CML), acute lymphoblastic leukemia (ALL), myelodysplastic or myeloproliferative diseases (MD/MPD), advanced hypereosinophilic syndrome (HES), chronic eosinophilic leukemia (CEL), gastrointestinal stromal tumors (GIST), dermatofibrosarcoma protuberans (DFSP)	**Enzyme (Antagonism)(N1.A)**	**/N1.A<br>//**	Enzyme (Transferases, Antagonism)(N1.2.A)	**/N1.2.A<br>//**	**Post-authorisation**	11/7/01	10/18/06	• open questions from the EMA (a17)<br>• concerns regarding the manufacturer's responses/information (a1)<br>• doubts about efficacy<br>reason: lack of data to sufficiently demonstrate efficacy (a27.3)<br>• doubts about efficacy<br>reason: poor study quality (small number of participants) (a27.1.5)	**/A17<br>/A1<br>/A27.3<br>/A27.1.5<br>//**	23/10/2006	**No extension of authorisation, because of no positive benefit-risk ratio**	**EMA evaluation**	**Number of clinical studies 01**	**Participants 0001-0024**	**No placebo control**	**SD**	**No comparison with approved drugs**	**No special drug approval procedure**	**No consequences for patients in ongoing clinical trials or compassionate use programmes**	Ongoing clinical trials (at the time the application was withdrawn)	https://www.ema.europa.eu/en/documents/medicine-qa/questions-and-answers-withdrawal-application-change-marketing-authorisation-glivec_en.pdf	https://www.ema.europa.eu/en/documents/other/withdrawal-letter-glivec_en.pdf	**Orphan drugs**	10/23/06
Prandin,	repaglinide	Repaglinide	Novo Nordisk A/S	Novo Nordisk A/S	**Other antidiabetics**	**A10BX02**	**05 Endocrine diseases, nutritional or metabolic diseases**	ICD-11 Chapter 05	diabetes mellitus, type 2 (in combination with thiazolidinediones (e.g., rosiglitazone or pioglitazone) when blood sugar levels could not be controlled with a thiazolidinedione alone)<br><br>already approved indications:<br>diabetes mellitus, type 2<br><br>Note:<br>Use under certain conditions	**Transporter (Antagonism) (N3.A)**	**/N3.A<br>//**	Transporter (Antagonism) (N3.A)	**/N3.A<br>//**	**Post-authorisation**	1/29/01	10/12/06	• open questions from the EMA (a17)<br>• concerns regarding the manufacturer's responses/information (a1)<br>• doubts about efficacy<br>reason: patients in the study were not compatible with the target indication (a27.4)<br>• doubts about efficacy<br>reason: inadequate study design (no comparison with an approved therapy consisting of a combination of a thiazolidinedione and a sulfonylurea) (a27.2)<br>• doubts about efficacy<br>reason: lack of data to sufficiently demonstrate efficacy (a27.3)	**/A17<br>/A1<br>/A27.4<br>/A27.2<br>/A27.3<br>//**	13/10/2006	**No extension of authorisation, because of no positive benefit-risk ratio**	**EMA evaluation**	**Number of clinical studies 02**	**Participants 0300-0499**	**No placebo control**		**Comparison with approved drugs**	**No special drug approval procedure**	**No consequences for patients in ongoing clinical trials or compassionate use programmes**	Ongoing clinical trials (at the time the application was withdrawn)	https://www.ema.europa.eu/en/documents/medicine-qa/questions-and-answers-withdrawal-application-change-marketing-authorisation-novonormprandin_en.pdf	https://www.ema.europa.eu/en/documents/other/withdrawal-letter-novonormprandin_en.pdf	**No orphan drugs**	10/13/06

NovoNorm,	repaglinide	Repaglinide	Novo Nordisk A/S	Novo Nordisk A/S	**Other antidiabetics**	**A10BX02**	**05 Endocrine diseases, nutritional or metabolic diseases**	ICD-11 Chapter 05	diabetes mellitus, type 2 (in combination with thiazolidinediones (e.g., rosiglitazone or pioglitazone) when blood sugar levels could not be controlled with a thiazolidinedione alone)<br><br>already approved indications:<br>diabetes mellitus, type 2<br><br>Note:<br>Use under certain conditions	**Transporter (Antagonism) (N3.A)**	**/N3.A //**	Transporter (Antagonism) (N3.A)	**/N3.A //**	**Post-authorisation**	8/16/98	10/12/06	• open questions from the EMA (a17)<br>• concerns regarding the manufacturer's responses/information (a1)<br>• doubts about efficacy<br>reason: patients in the study were not compatible with the target indication (a27.4)<br>• doubts about efficacy<br>reason: inadequate study design (no comparison with an approved therapy consisting of a combination of a thiazolidinedione and a sulfonylurea) (a27.2)<br>• doubts about efficacy<br>reason: lack of data to sufficiently demonstrate efficacy (a27.3)	**/A17 /A1 /A27.4 /A27.2 /A27.3 //**	13/10/2006	**No extension of authorisation, because of no positive benefit-risk ratio**	**EMA evaluation**	**Number of clinical studies 02**	**Participants 0300-0499**	**No placebo control**		**Comparison with approved drugs**	**No special drug approval procedure**	**No consequences for patients in ongoing clinical trials or compassionate use programmes**	Ongoing clinical trials (at the time the application was withdrawn)	https://www.ema.europa.eu/en/documents/medicine-qa/questions-and-answers-withdrawal-application-change-marketing-authorisation-novonormprandin_en.pdf	https://www.ema.europa.eu/en/documents/other/withdrawal-letter-novonormprandin_en.pdf	**No orphan drugs**	10/13/06
Riquent,	abetimus	Abetimus	La Jolla Limited	La Jolla Limited	**Immunosuppressants (no monoclonal antibodies)**	**L04AA22**	**04 Diseases of the immune system**	ICD-11 Chapter 04	lupus nephritis	**other (N6)**	**/N6.U //**	other (N6)	**/N6.U //**	**Initial authorisation**		10/13/06	• not present (documents have not yet been reviewed or recommendations not yet published) (a14.1)<br><br>note:<br>documents had not been fully reviewed at the time of withdrawal of the marketing authorisation application.	**/A14.1 //**	13/10/2006	**None (documents have not yet been evaluated)**	**Development strategy other reasons**	**Number of clinical studies 02**	**Participants 0500-0999**	**Placebo controlled clinical studies**		**No comparison with approved drugs**	**No special drug approval procedure**	**No consequences for patients in ongoing clinical trials or compassionate use programmes**	Ongoing clinical trials (at the time the application was withdrawn)	https://www.ema.europa.eu/en/documents/medicine-qa/questions-and-answers-withdrawal-marketing-application-riquent_en.pdf	https://www.ema.europa.eu/en/documents/withdrawal-letter/withdrawal-letter-riquent_en.pdf	**Orphan drugs**	10/13/06
Multaq,	dronedarone	Dronedarone	Sanofi-Aventis	Sanofi	**Antiarrhythmic agent**	**C01BD07**	**11 Diseases of the circulatory system**	ICD-11 Chapter 11	atrial fibrillation, atrial flutter	**Ion channels (Antagonism) (N2.A)**	**/N2.A //**	Ion channels (Antagonism) (N2.A)	**/N2.A //**	**Initial authorisation**		9/6/06	• open questions from the EMA (a17)<br>• concerns regarding the manufacturer's responses/information (a1)<br>• doubts about efficacy<br>reason: poor study quality or inadequate study design (lack of comparison with placebo and/or comparator/reference medicinal products) (a27.1.4)<br>• doubts about equivalent/improved efficacy compared with the eu reference medicinal product (a25)<br>• doubts about assurance of patient safety<br>reason: adverse reactions (a21.1)<br>• doubts about assurance of patient safety<br>reason: interactions with other medicinal products (a21.2)	**/A17 /A1 /A27.1.4 /A25 /A21.1 /A21.2 //**	8/09/2006	**No authorisation, because of no positive benefit-risk ratio**	**EMA evaluation**	**Number of clinical studies 03**	**Participants 1000-1999**	**Placebo controlled clinical studies**		**No comparison with approved drugs**	**No special drug approval procedure**	**No consequences for patients in ongoing clinical trials or compassionate use programmes**	Ongoing clinical trials (at the time the application was withdrawn)	https://www.ema.europa.eu/en/documents/medicine-qa/questions-and-answers-withdrawal-marketing-application-multaq_en.pdf	https://www.ema.europa.eu/en/documents/withdrawal-letter/withdrawal-letter-multaq_en.pdf	**No orphan drugs**	9/8/06
DepoCyte,	cytarabine	Cytarabine	Pacira Limited/ Skye Pharma PLC	Pacira Limited/ Skye Pharma PLC	**Cytostatics**	**L01BC01**	**08 Diseases of the nervous system**	ICD-11 Chapter 08	meningeosis neoplastica<br><br>already approved indications:<br>lymphatic meningitis	**Enzyme (Antagonism)(N1.A)**	**/N1.A //**	Enzyme (Antagonism) (N1.A)	**/N1.A //**	**Post-authorisation**	7/11/01	6/28/06	• concerns regarding the manufacturer's responses/information (a1)<br>• doubts about efficacy<br>reason: poor study quality (data analysis) (a27.1.3)<br>• doubts about equivalent/improved efficacy compared with the eu reference medicinal product (a25)	**/A1 /A27.1.3 /A25 //**	30/06/2006	**No authorisation, because of no positive benefit-risk ratio**	**EMA evaluation**	**Number of clinical studies 02**	**Participants 0150-0199**	**No placebo control**		**Comparison with approved drugs**	**No special drug approval procedure**	**No consequences for patients in ongoing clinical trials or compassionate use programmes**	Ongoing clinical trials (at the time the application was withdrawn)	https://www.ema.europa.eu/en/documents/medicine-qa/questions-and-answers-withdrawal-application-change-marketing-authorisation-depocyte_en.pdf	https://www.ema.europa.eu/en/documents/other/withdrawal-letter-depocyte_en.pdf	**No orphan drugs**	6/30/06
Surfaxin,	unknown	Sinapultid, Dipalmitoylphosphatidylcholine, Palmitoyl-Oleoyl-Phosphatidylglycerol and Palmitic acid.	Pharm Research Associates (UK) Limited	Pharm Research Associates (UK) Limited	**Other medicines**	**Unknown**	**19 Certain diseases originating in the perinatal period**	ICD-11 Chapter 19	respiratory distress syndrome (ANS) in premature infants (prevention and treatment)	**other (N6)**	**/N6.U //**	other (N6)	**/N6.U //**	**Initial authorisation**		6/7/06	• open questions from the EMA (a17)<br>• concerns regarding the manufacturer's responses/information (a1)<br>• doubts about efficacy<br>reason: lack of data to sufficiently demonstrate efficacy (a27.3)<br>• concerns regarding medicinal product quality (manufacturing process) (a3.1)<br>• doubts about equivalent/improved efficacy compared with the eu reference medicinal product (a25)<br>• doubts about efficacy<br>reason: inadequate study design (a27.2)	**/A17 /A1 /A3.1 /A27.3 /A25 /A27.2 //**	8/06/2006	**No authorisation, because of no positive benefit-risk ratio**	**Manufacturing issues other reasons**	**Number of clinical studies 02**	**Participants 1000-1999**	**No placebo control**		**Comparison with approved drugs**	**No special drug approval procedure**	**No consequences for patients in ongoing clinical trials or compassionate use programmes**	No ongoing clinical trials (at the time the application was withdrawn)	https://www.ema.europa.eu/en/documents/medicine-qa/questions-and-answers-withdrawal-marketing-application-surfaxin_en.pdf	https://www.ema.europa.eu/en/documents/withdrawal-letter/withdrawal-letter-surfaxin_en.pdf	**Orphan drugs**	6/8/06
Scintimun	besilesomab	Besilesomab with Sodium Pertechnetate (99mTc)	CIS bio international	CIS bio international	**Monoclonal antibodies**	**Unknown**	**X Extension codes (Medicines used in diagnostic tests, not elsewhere classified)**<br><br>**01 Certain infectious or parasitic diseases**<br><br>**02 Neoplasms**	ICD-11 Chapter 01<br><br>ICD-11 Chapter 02<br><br>ICD-11 Chapter X	localization of infectious or inflammatory lesions and detection of metastases in the bone marrow with imaging diagnostic procedures	**other (N6)**	**/N6.U //**	other (N6)	**/N6.U //**	**Initial authorisation**		5/17/06	• open questions from the EMA (a17)<br>• concerns regarding the manufacturer's responses/information (a1)<br>• doubts about efficacy<br>reason: inadequate study design (a27.2)<br>• doubts about efficacy<br>reason: lack of data to sufficiently demonstrate efficacy (a27.3)	**/A17 /A1 /A27.2 /A27.3 //**	22/05/2006	**No authorisation, because of no positive benefit-risk ratio**	**EMA evaluation**	**Number of clinical studies 07**	**Participants 1000-1999**	**No placebo control**	**SD**	**Comparison with approved drugs unknown**	**No special drug approval procedure**	**No consequences for patients in ongoing clinical trials or compassionate use programmes**	Ongoing clinical trials (at the time the application was withdrawn)	https://www.ema.europa.eu/en/documents/medicine-qa/questions-and-answers-withdrawal-marketing-application-scintimun_en.pdf	https://www.ema.europa.eu/en/documents/withdrawal-letter/withdrawal-letter-scintimun_en.pdf	**No orphan drugs**	5/22/06
NovoSeven,	eptacog alfa (activated)	Eptacog alfa (activated)	Novo Nordisk A/S	Novo Nordisk A/S	**Other medicines**	**B02BD08**	**08 Diseases of the nervous system**	ICD-11 Chapter 08	intracerebral hemorrhage (in adult patients)<br><br>already approved indications:<br>hemophilia A, hemophilia B, factor VII deficiency, Glanzmann-Naegeli thrombastenia	**Enzyme (Antagonism)(N1.A)**	**/N1.A //**	Enzyme (Hydrolases, Activation)(N1.3.B)	**/N1.3.B //**	**Post-authorisation**	2/23/96	4/3/06	• concerns regarding the manufacturer's responses/information (a1)<br>• doubts about efficacy<br>reason: lack of data to sufficiently demonstrate efficacy (a27.3)<br>• doubts about assurance of patient safety<br>reason: adverse reactions (thromboembolic events) (a21.1)<br>• doubts about efficacy<br>reason: poor study quality (small number of participants) (a27.1.5)<br>• concerns regarding the recommended dose (a6)	**/A1 /A27.3 /A21.1 /A27.1.5 /A6 //**	6/04/2006	**No authorisation, because of no positive benefit-risk ratio**	**EMA evaluation**	**Number of clinical studies 03**	**Participants 0300-0499**	**Placebo controlled clinical studies**		**Comparison with approved drugs unknown**	**No special drug approval procedure**	**No consequences for patients in ongoing clinical trials or compassionate use programmes**	Ongoing clinical trials (at the time the application was withdrawn)	https://www.ema.europa.eu/en/documents/medicine-qa/questions-and-answers-withdrawal-application-change-marketing-authorisation-novoseven_en.pdf	https://www.ema.europa.eu/en/documents/other/withdrawal-letter-novoseven_en.pdf	**No orphan drugs**	4/6/06
Retaane,	anecortave	Anecortave	Alcon Laboratories (U.K.) Limited.	Alcon Laboratories (U.K.) Limited.	**Other medicines**	**S01LA02**	**09 Diseases of the visual system**	ICD-11 Chapter 09	age-related macular degeneration (AMD) (wet form with subfoveal choroidal neovascularization (CNV))	**other (N6)**	**/N6.U //**	other (N6)	**/N6.U //**	**Initial authorisation**		2/28/06	• open questions from the EMA (a17)<br>• concerns regarding the manufacturer's responses/information (a1)<br>• doubts about equivalent/improved efficacy compared with the eu reference medicinal product (results worse than standard treatment) (a25)<br>• doubts about efficacy<br>reason: lack of data to sufficiently demonstrate efficacy (due to high dropout rate in clinical study) (a27.3)	**/A17 /A1 /A25 /A27.3 //**	2/03/2006	**No authorisation, because of no positive benefit-risk ratio**	**Marketing strategy Development strategy**	**Number of clinical studies 02**	**Participants 0500-0999**	**Placebo controlled clinical studies**		**Comparison with approved drugs**	**No special drug approval procedure**	**No consequences for patients in ongoing clinical trials or compassionate use programmes**	Ongoing clinical trials (at the time the application was withdrawn)	https://www.ema.europa.eu/en/documents/medicine-qa/questions-and-answers-withdrawal-marketing-application-retaane_en.pdf	https://www.ema.europa.eu/en/documents/withdrawal-letter/withdrawal-letter-retaane_en.pdf	**No orphan drugs**	3/2/06
Opatanol,	olopatadine	Olopatadine Hydrochloride	Novartis Europharm Limited/Alcon Laboratories (U.K.) Limited.	Novartis Europharm Limited/Alcon Laboratories (U.K.) Limited.	**Antihistamines**	**S01GX09**	**12 Respiratory diseases**	ICD-11 Chapter 12	seasonal and perennial (all-year) rhinitis<br><br>already approved indications:<br>seasonal allergic conjunctivitis	**Receptors (Antagonism) (N4.A)**<br><br>**other (N6)**	**/N4.A /N6.U //**	Receptors (GPCR, Antagonism) (N4.1.A)<br><br>other (N6)	**/N4.1.A /N6.U //**	**Post-authorisation**	5/16/02	2/14/06	• open questions from the EMA (a17)<br>• concerns regarding the manufacturer's responses/information (a1)<br>• doubts about efficacy<br>reason: poor study quality or inadequate study design (lack of comparison with placebo and/or comparator/reference medicinal products) (a27.1.4)<br>• concerns regarding medicinal product quality (contaminants and/or concerns regarding sterilisation) (possible contamination) (a3.3)	**/A17 /A1 /A27.1.4 /A3.3 //**	23/02/2006	**No authorisation, because of no positive benefit-risk ratio**	**Marketing strategy**	**Number of clinical studies unknown**	**Participants unknown**	**Placebo controlled clinical studies**		**No comparison with approved drugs**	**No special drug approval procedure**	**No consequences for patients in ongoing clinical trials or compassionate use programmes**	No ongoing clinical trials (at the time the application was withdrawn)	https://www.ema.europa.eu/en/documents/medicine-qa/questions-and-answers-withdrawal-application-change-marketing-authorisation-opatanol_en.pdf	https://www.ema.europa.eu/en/documents/other/withdrawal-letter-opatanol_en.pdf	**No orphan drugs**	2/23/06
Ximelagatran 36 mg film coated tablets,	ximelagatran	Ximelagatran	AstraZeneca	AstraZeneca	**Anticoagulants**	**B01AE05**	**X Extension codes (Anticoagulation)**	ICD-11 Chapter X	prevention of strokes and other complications (due to excessive coagulability) in atrial fibrillation	**Enzyme (Antagonism)(N1.A)**	**/N1.A //**	Enzyme (Hydrolases, Antagonism)(N1.3.A)	**/N1.3.A //**	**Initial authorisation**		2/14/06	• not present (documents have not yet been reviewed or recommendations not yet published) (a14.1)<br><br>note:<br>documents had not been fully reviewed at the time of withdrawal of the marketing authorisation application.	**/A14.1 //**	16/02/2006	**None (documents have not yet been evaluated)**	**Other reasons**	**Number of clinical studies unknown**	**Participants unknown**	**No placebo control**		**Comparison with approved drugs**	**No special drug approval procedure**	**Consequences for patients in ongoing clinical trials or compassionate use programmes**	No ongoing clinical trials (at the time the application was withdrawn)	https://www.ema.europa.eu/en/documents/medicine-qa/questions-and-answers-withdrawal-marketing-application-ximelagatran-astrazeneca-36-mg-film-coated-tablets_en.pdf	https://www.ema.europa.eu/en/documents/withdrawal-letter/withdrawal-letter-ximelagatran-36-mg-film-coated-tablets_en.pdf	**No orphan drugs**	2/16/06
Orathecin,	rubitecan	Rubitecan	EuroGen Pharmaceuticals Ltd	EuroGen Pharmaceuticals Ltd	**Cytostatics**	**Unknown**	**02 Neoplasms**	ICD-11 Chapter 02	pancreatic carcinoma (advanced or metastatic)	**Enzyme (Antagonism)(N1.A)**	**/N1.A //**	Enzyme (Isomerases, Antagonism) (N1.5.A)	**/N1.5.A //**	**Initial authorisation**		1/19/06	• concerns regarding the manufacturer's responses/information (a1)<br>• doubts about assurance of patient safety<br>reason: adverse reactions (a21.1)<br>• doubts about efficacy<br>reason: lack of data to sufficiently demonstrate efficacy (study results show no benefit in terms of overall survival and quality of life) (a27.3)	**/A1 /A21.1 /A27.3 //**	23/01/2006	**No authorisation, because of no positive benefit-risk ratio**	**EMA evaluation**	**Number of clinical studies 02**	**Participants 0500-0999**	**No placebo control**		**Comparison with approved drugs**	**No special drug approval procedure**	**No consequences for patients in ongoing clinical trials or compassionate use programmes**	Ongoing clinical trials (at the time the application was withdrawn)	https://www.ema.europa.eu/en/documents/medicine-qa/questions-and-answers-withdrawal-marketing-application-orathecin_en.pdf	https://www.ema.europa.eu/en/documents/withdrawal-letter/withdrawal-letter-orathecin_en.pdf	**Orphan drugs**	1/23/06
